# Supplementary material for: Differentiated glioma cell-derived fibromodulin activates integrin-dependent Notch signaling in endothelial cells to promote tumor angiogenesis and growth
Source: eLife. 2022 Jun 1;11:e78972. doi: 10.7554/eLife.78972 (PMC9259034; doi:10.7554/eLife.78972)
Supplement: Figure 1—source data 5. [file elife-78972-fig1-data5.zip › Figure 1-Source data E,F,H,I/Blots E,F,H,I.pptx]

## Slide 1
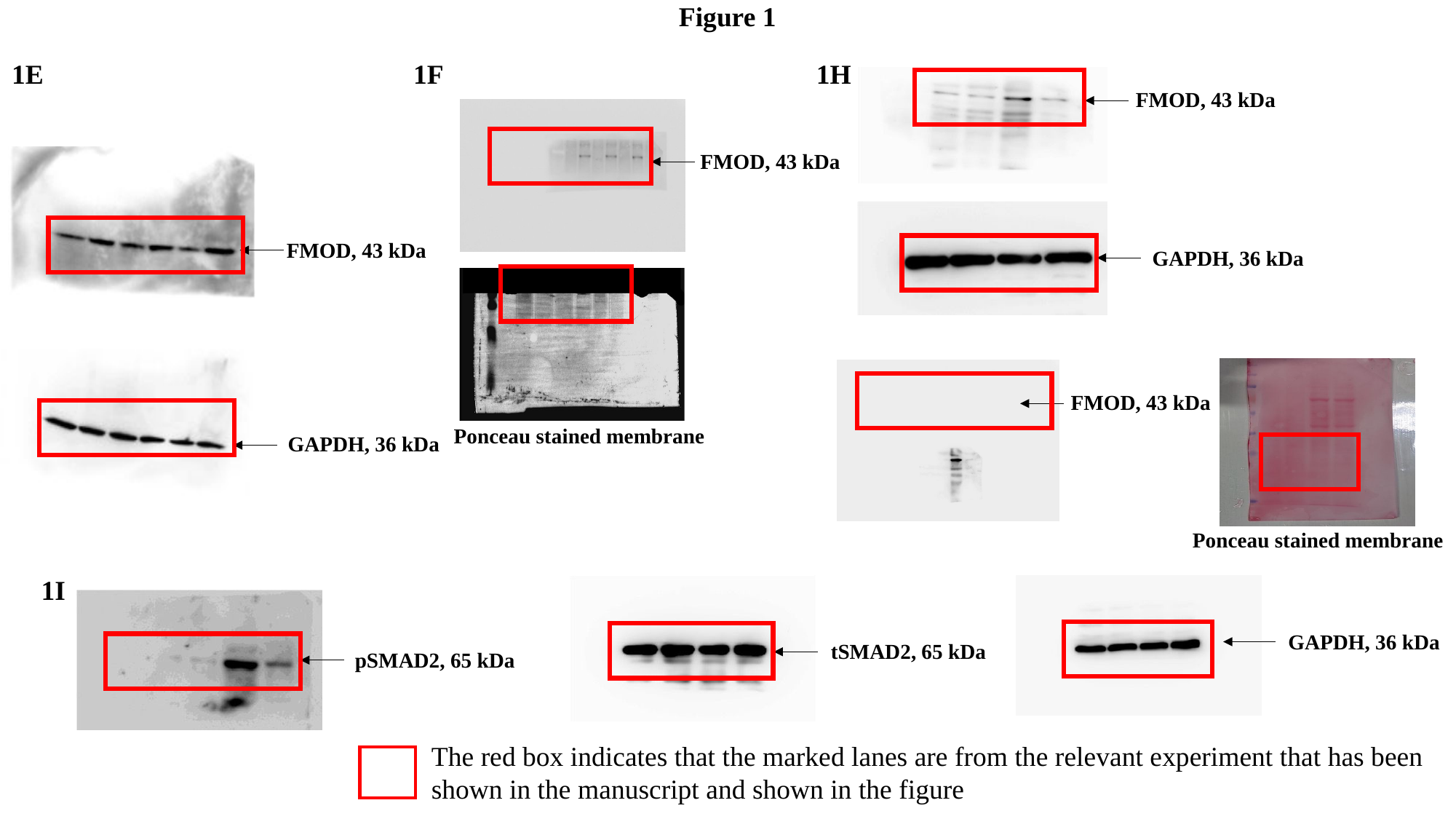

Figure 1
1E
1F
1H
FMOD, 43 kDa
FMOD, 43 kDa
FMOD, 43 kDa
GAPDH, 36 kDa
FMOD, 43 kDa
Ponceau stained membrane
GAPDH, 36 kDa
Ponceau stained membrane
1I
GAPDH, 36 kDa
tSMAD2, 65 kDa
pSMAD2, 65 kDa
The red box indicates that the marked lanes are from the relevant experiment that has been shown in the manuscript and shown in the figure
