# Supplementary figures and images for: Differentiated glioma cell-derived fibromodulin activates integrin-dependent Notch signaling in endothelial cells to promote tumor angiogenesis and growth
Source: eLife. 2022 Jun 1;11:e78972. doi: 10.7554/eLife.78972 (PMC9259034; doi:10.7554/eLife.78972)

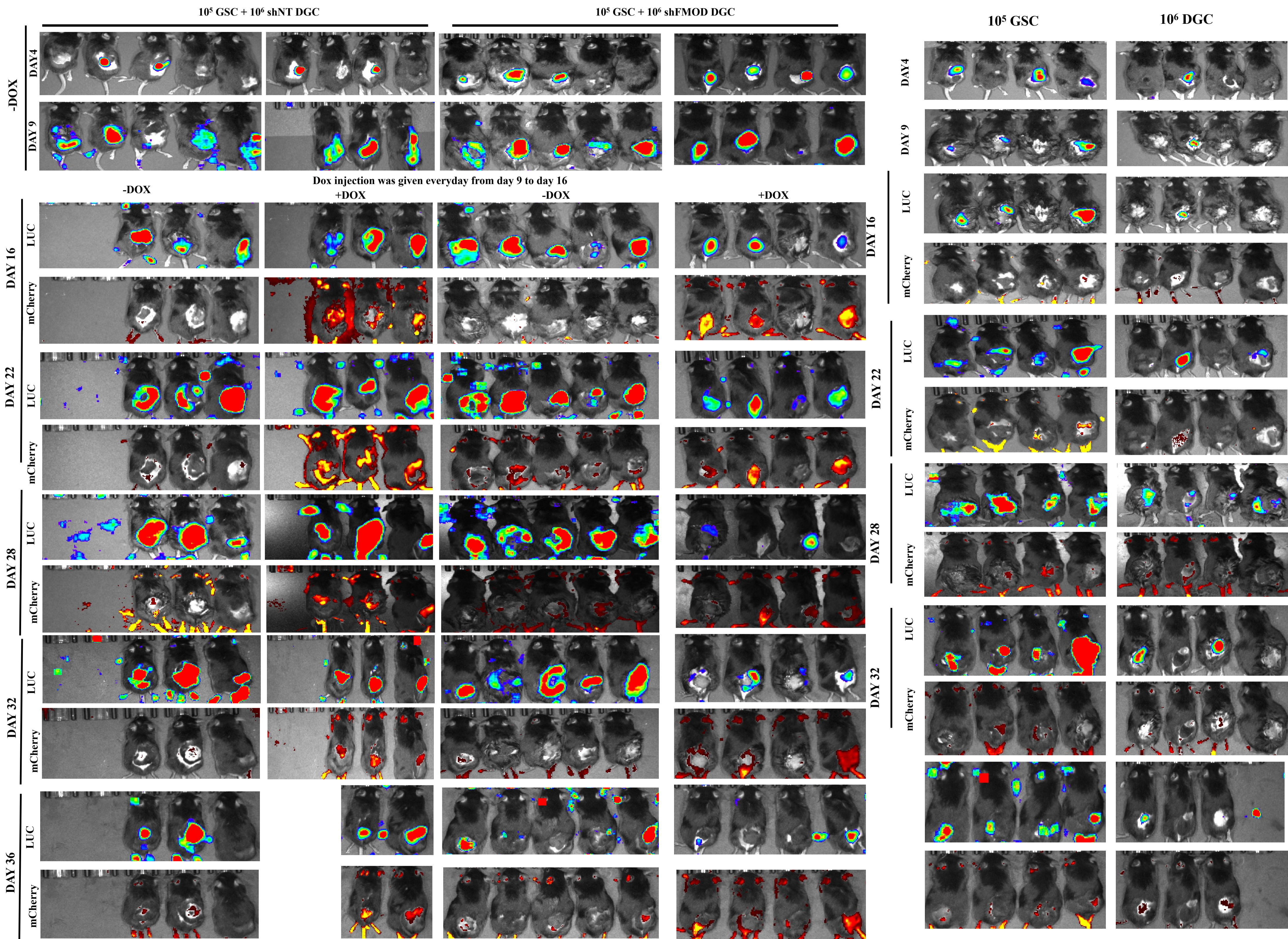

Supplement: Figure 2—source data 1. [file elife-78972-fig2-data1.zip › Figure 2-Source data C/SUBCUTANEOUS SSG 23.12.2020.pdf]

## Supplementary Figure 6

6A

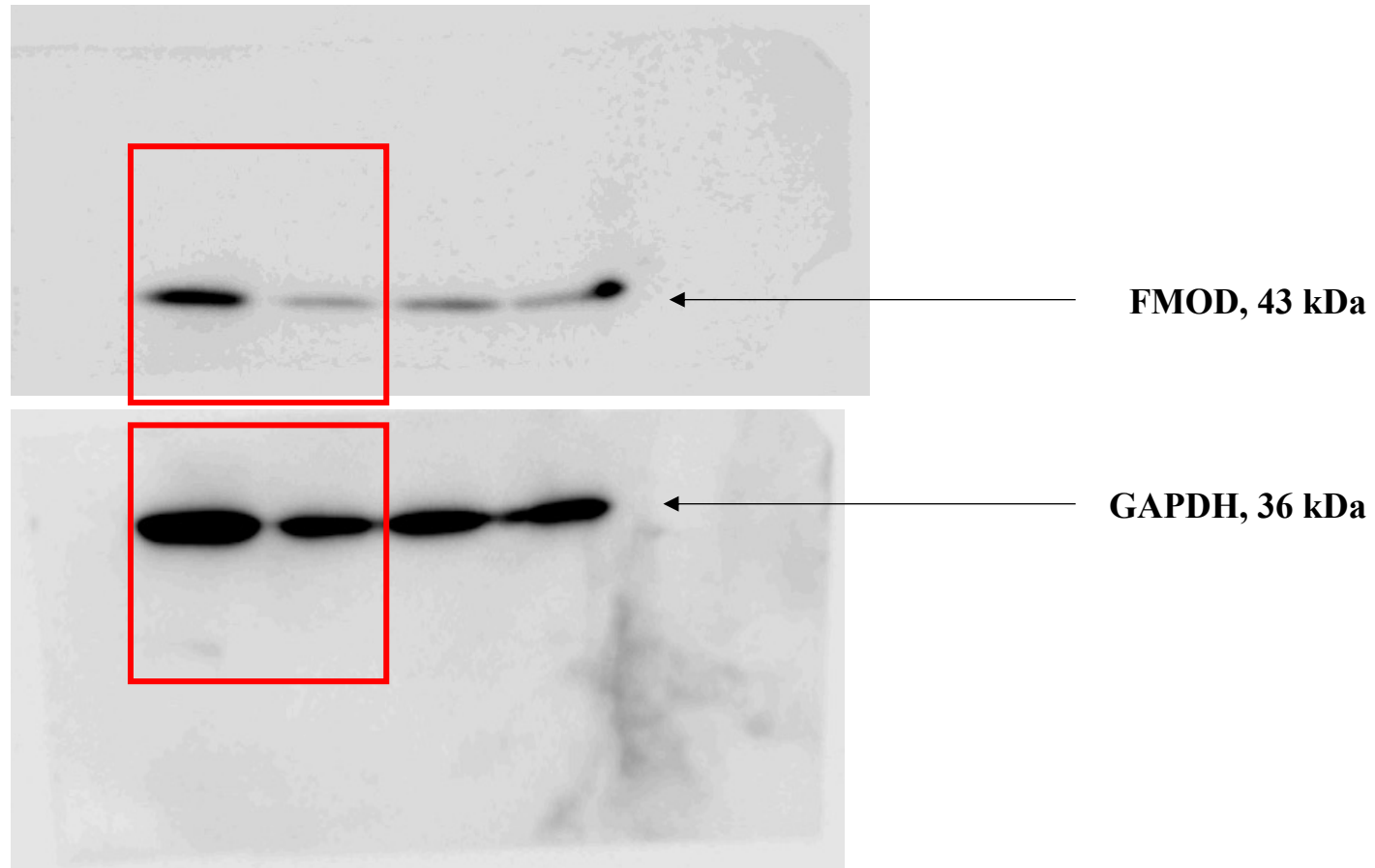

Supplement: Figure 2—figure supplement 1—source data 1. [file elife-78972-fig2-figsupp1-data1.zip › Figure 2-Figure Supplement 1-Source Data/Figure 2-Figure supplement 1 Source Data A/BLOT FOR PANEL A.pdf]

**MGG8-GSC/  
shNT**

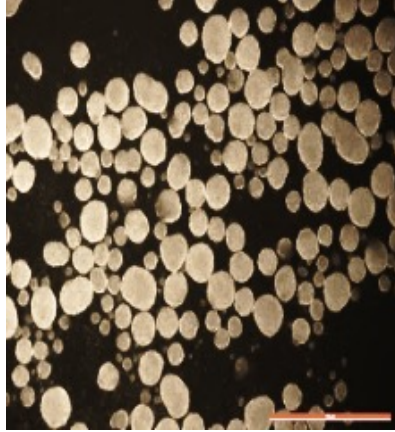

**MGG8-GSC/  
shFMOD**

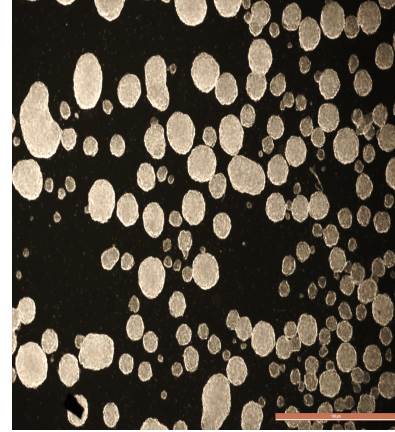

**MGG8-DGC/  
shNT**

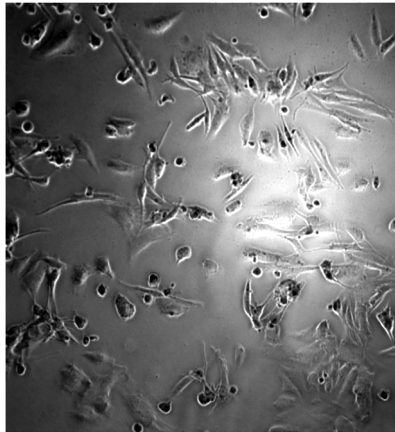

**MGG8-DGC/  
shFMOD**

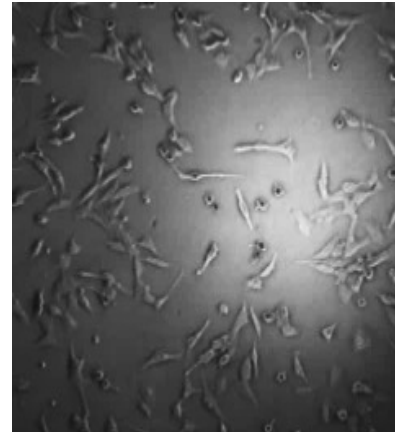

Supplement: Figure 2—figure supplement 1—source data 1. [file elife-78972-fig2-figsupp1-data1.zip › Figure 2-Figure Supplement 1-Source Data/Figure 2-Figure supplement 1 Source Data D/IMAGES FOR PANEL D.pdf]

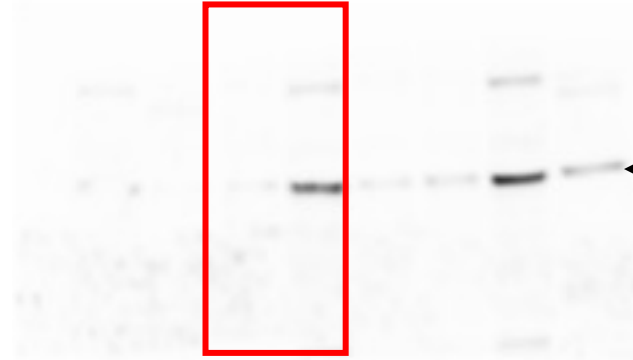

← **FMOD, 43 kDa**

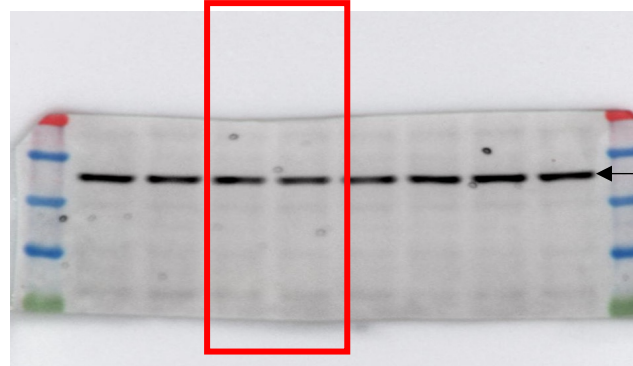

← **Tubulin, 50kDa**

Supplement: Figure 2—figure supplement 2—source data 1. [file elife-78972-fig2-figsupp2-data1.zip › Figure 2-Figure Supplement 2-Source Data/Figure 2-Figure supplement 2 Source Data B/BLOTS FOR PANEL B.pdf]

## Supplementary Figure 7

7C

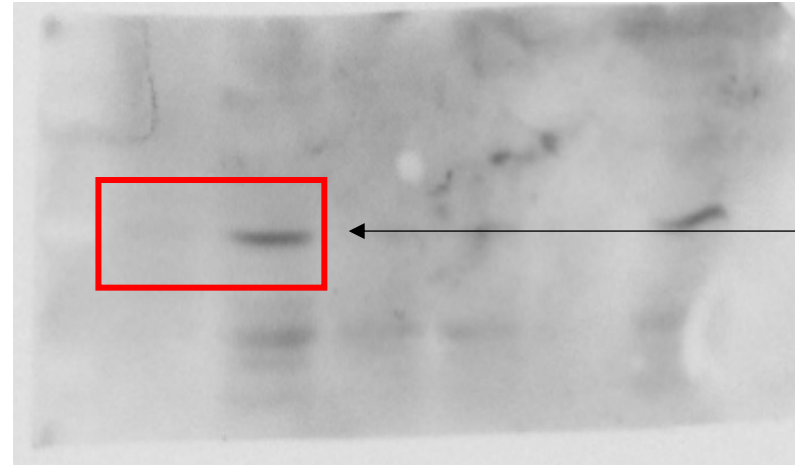

FMOD, 43 kDa

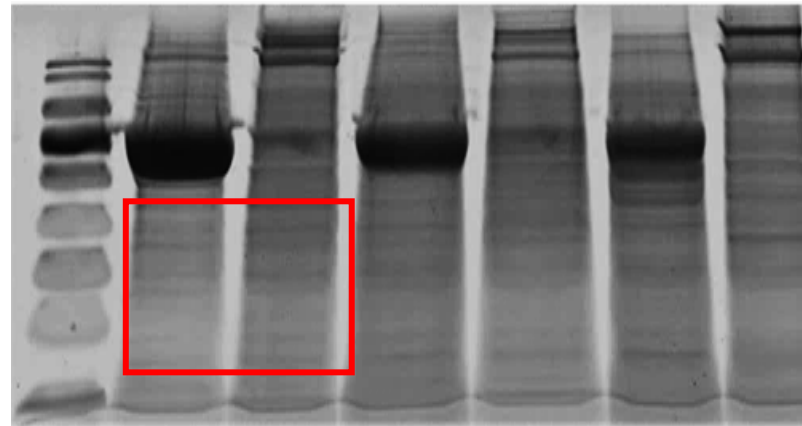

Ponceau stained membrane

Supplement: Figure 2—figure supplement 2—source data 1. [file elife-78972-fig2-figsupp2-data1.zip › Figure 2-Figure Supplement 2-Source Data/Figure 2-Figure supplement 2 Source Data C/BLOTS FOR PANEL C.pdf]

**7E**

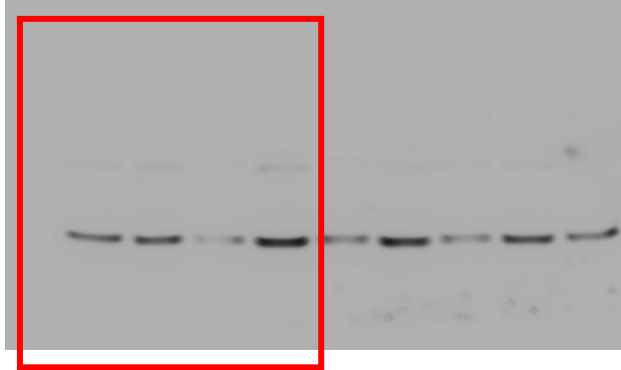

← **FMOD, 43 kDa**

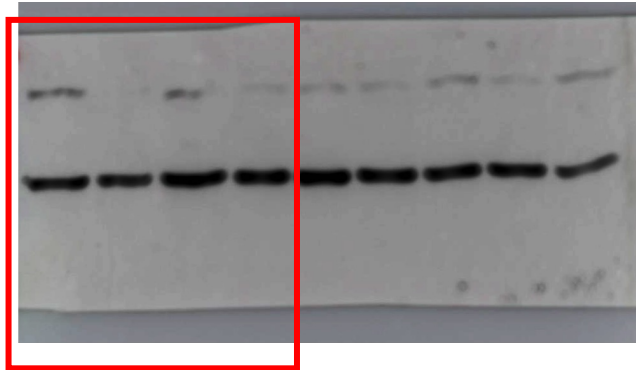

← **Tubulin, 50kDa**

Supplement: Figure 2—figure supplement 2—source data 1. [file elife-78972-fig2-figsupp2-data1.zip › Figure 2-Figure Supplement 2-Source Data/Figure 2-Figure supplement 2 Source Data E/BLOTS FOR PANEL E.pdf]

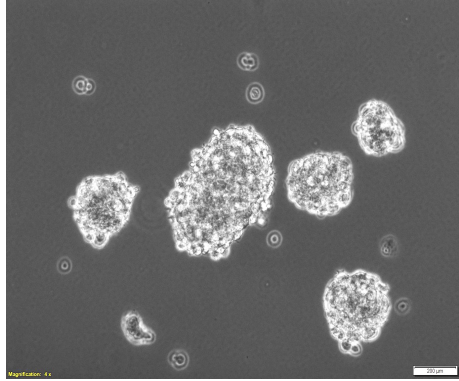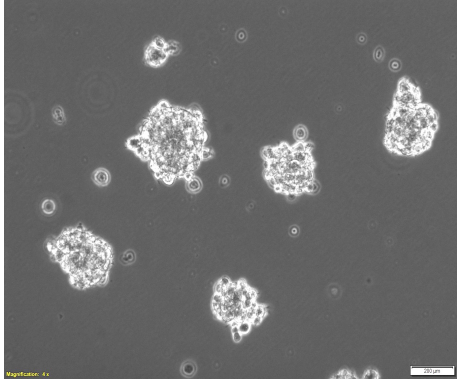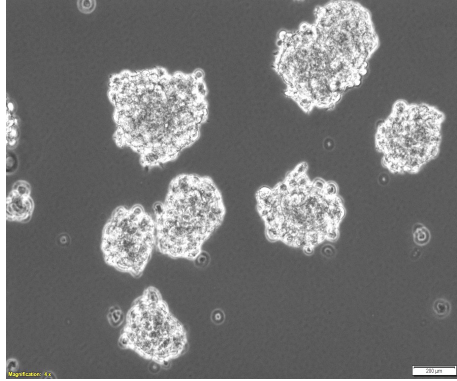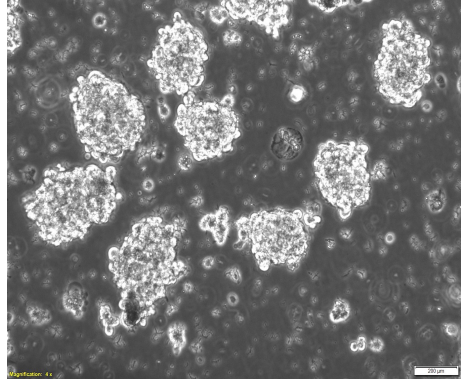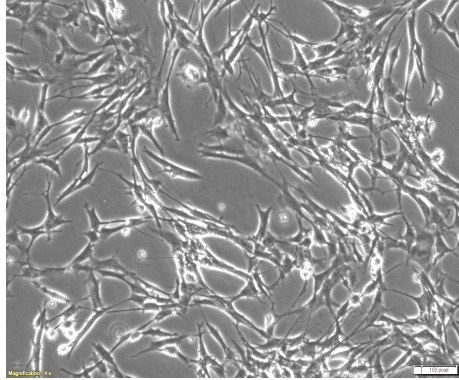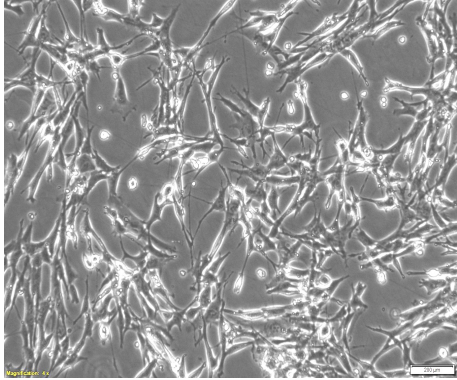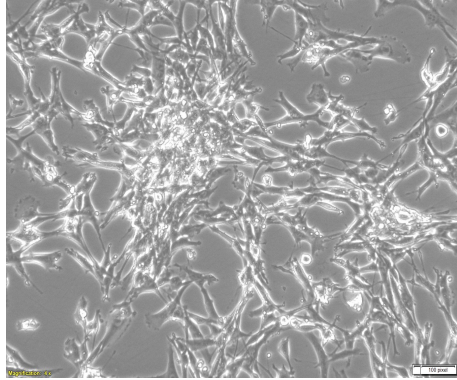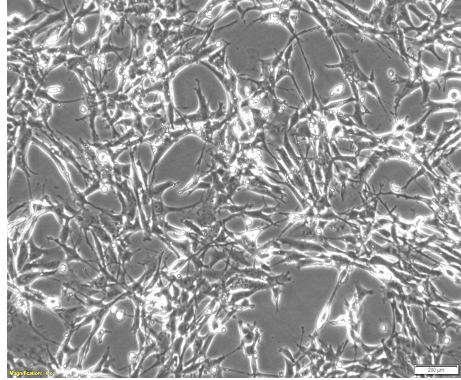

Supplement: Figure 2—figure supplement 3—source data 1. [file elife-78972-fig2-figsupp3-data1.zip › Figure 2-Figure Supplement 3-Source Data/Figure 2-Figure supplement 3 Source Data C/IMAGES FOR PANEL C.pdf]

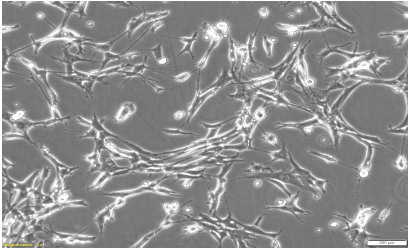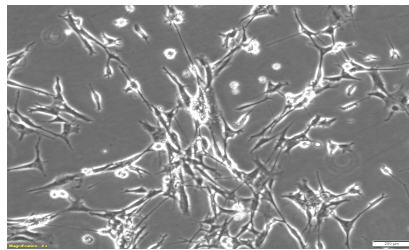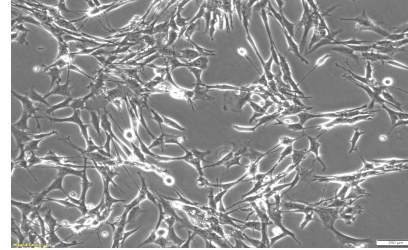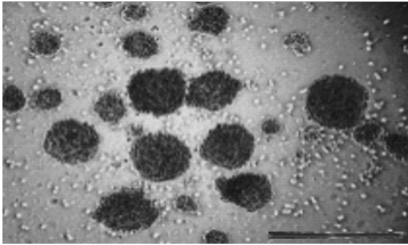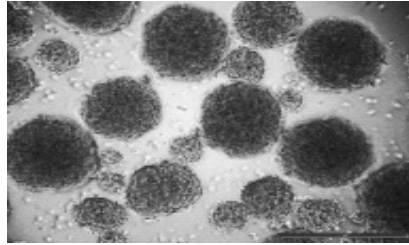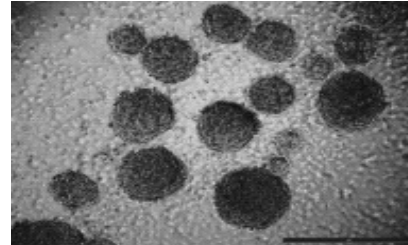

Supplement: Figure 2—figure supplement 4—source data 1. [file elife-78972-fig2-figsupp4-data1.zip › Figure 2-Figure Supplement 4-Source Data/Figure 2-Figure supplement 4 Source Data A/IMAGES FOR PANEL A.pdf]

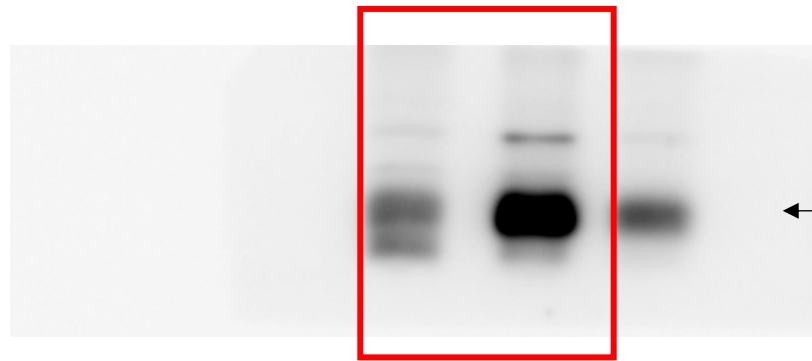

← **FMOD, 43 kDa**

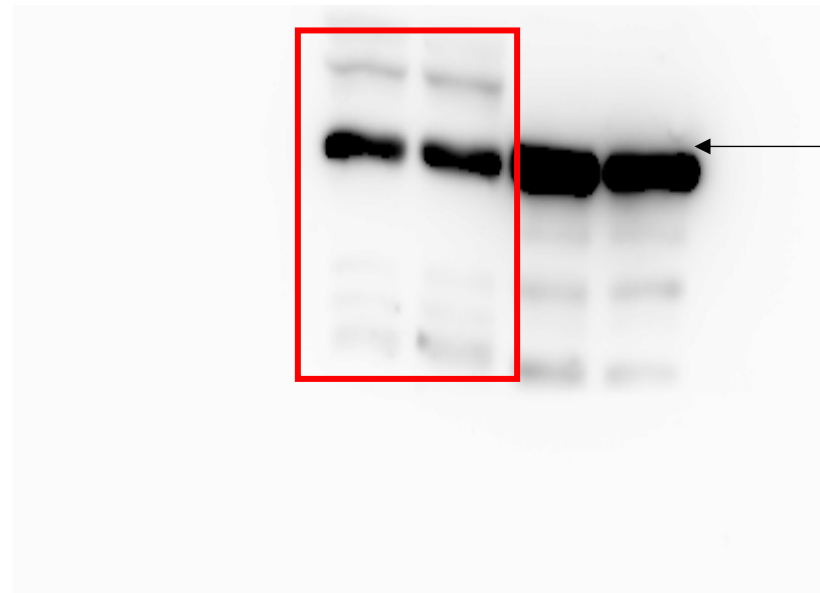

← **Tubulin, 50kDa**

Supplement: Figure 2—figure supplement 5—source data 1. [file elife-78972-fig2-figsupp5-data1.zip › Figure 2-Figure Supplement 5-Source Data/Figure 2-Figure supplement 5 Source Data D/BLOTS FOR PANEL D.pdf]

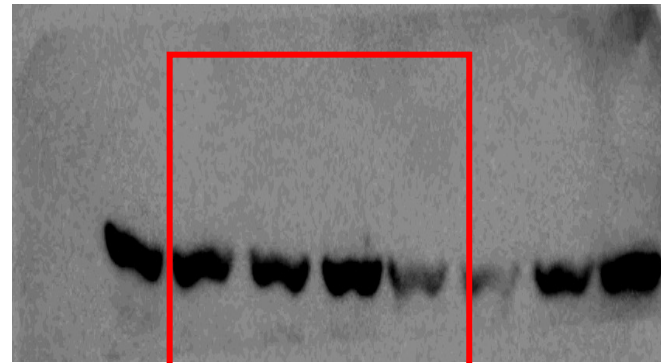

← **FMOD, 43 kDa**

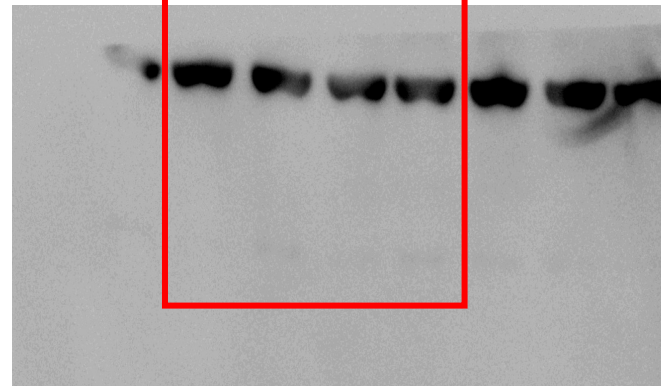

← **Tubulin, 50kDa**

Supplement: Figure 2—figure supplement 5—source data 1. [file elife-78972-fig2-figsupp5-data1.zip › Figure 2-Figure Supplement 5-Source Data/Figure 2-Figure supplement 5 Source Data F/BLOTS FOR PANEL F.pdf]

**DBT-Luc-DGC/miRNT (Dox+)**

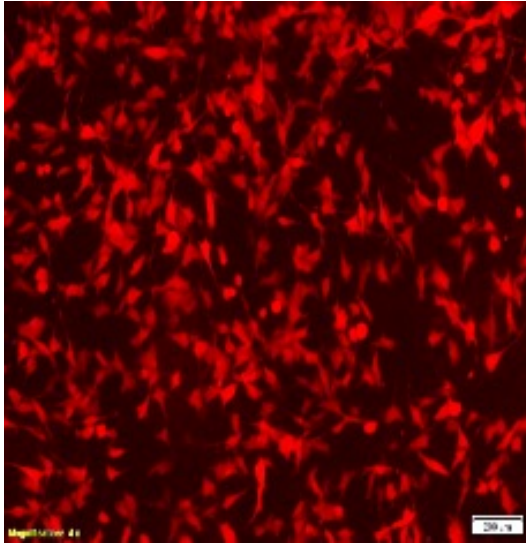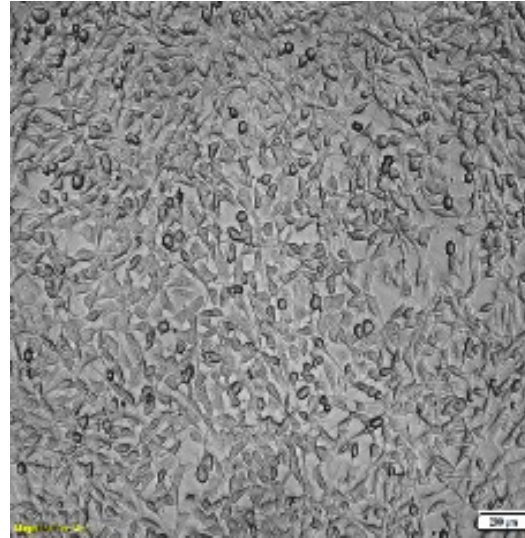

**DBT-Luc-DGC/miRFMOD (Dox+)**

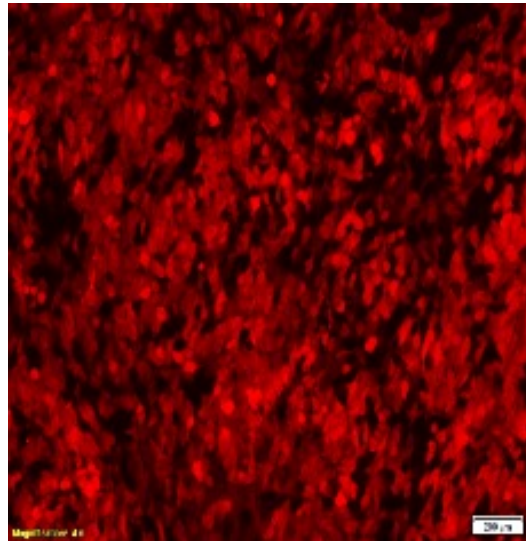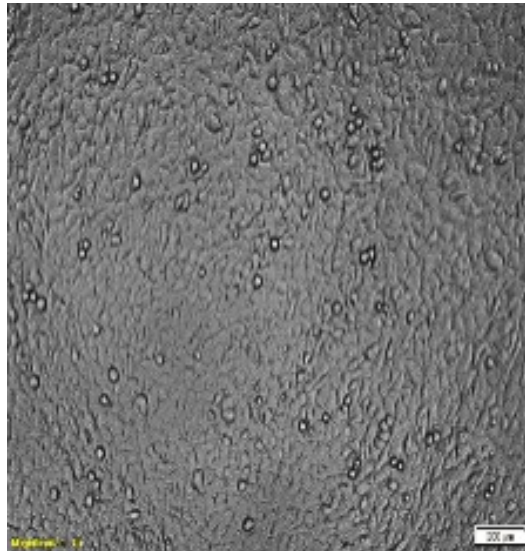

Supplement: Figure 2—figure supplement 5—source data 1. [file elife-78972-fig2-figsupp5-data1.zip › Figure 2-Figure Supplement 5-Source Data/Figure 2-Figure supplement 5 Source Data G/IMAGES FOR PANEL G.pdf]

## Supplementary Figure 11

Gel pic

11A

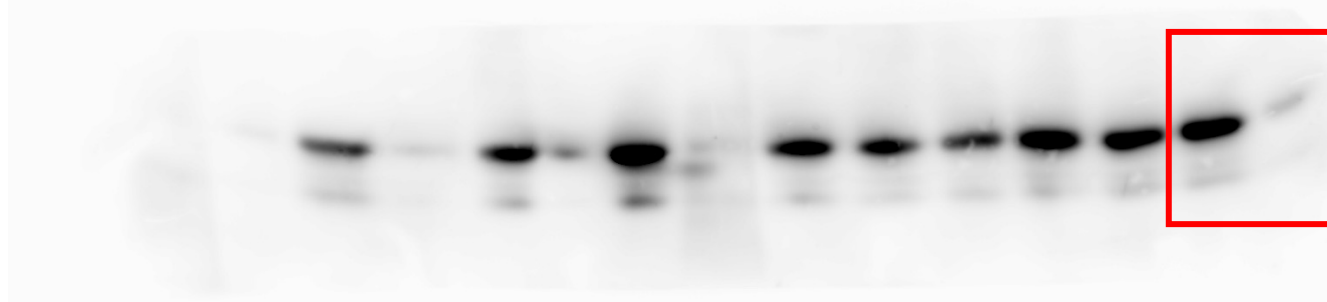

FMOD, 43 kDa

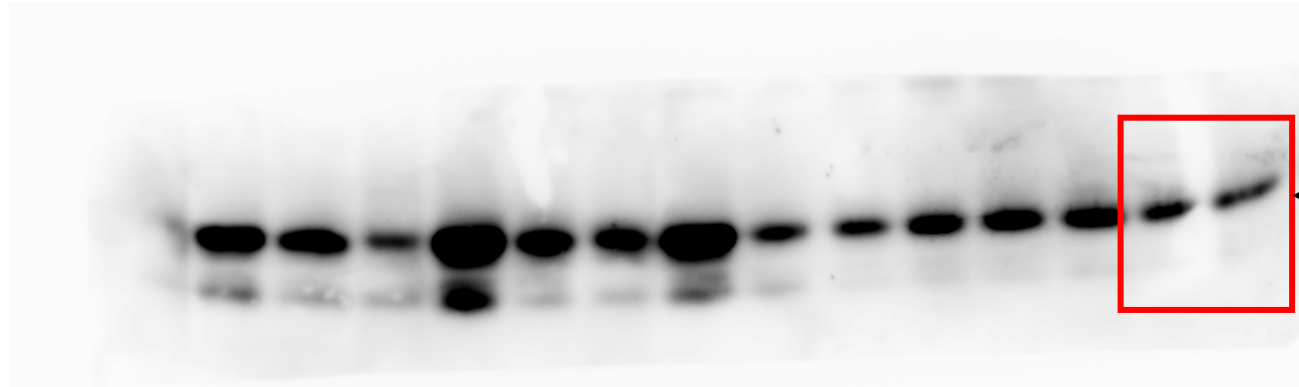

GAPDH, 36 kDa

Supplement: Figure 2—figure supplement 6—source data 1. [file elife-78972-fig2-figsupp6-data1.zip › Figure 2-Figure Supplement 6-Source Data/Figure 2-Figure supplement 6 Source Data A/BLOTS FOR PANEL A.pdf]

**U251-DGC/  
shNT**

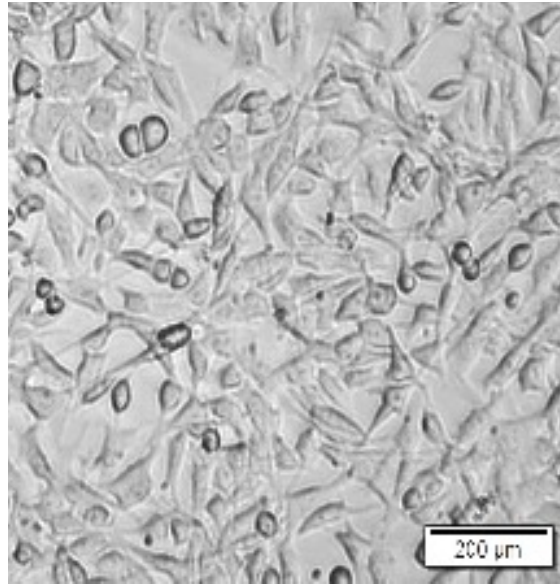

**U251-DGC/  
shFMOD**

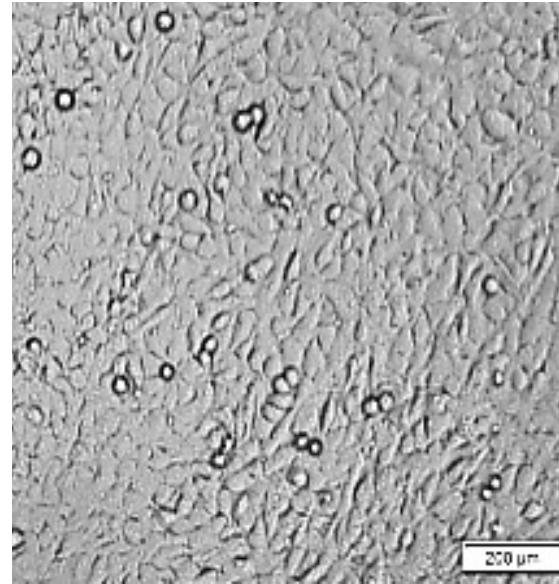

**U251-GSC/  
shNT**

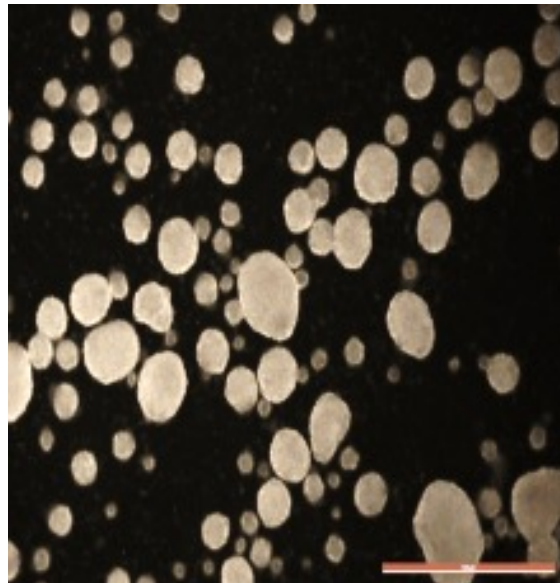

**U251-GSC/  
shFMOD**

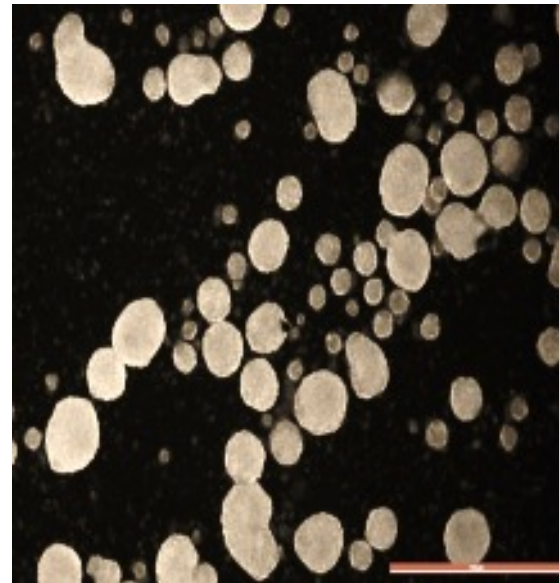

Supplement: Figure 2—figure supplement 6—source data 1. [file elife-78972-fig2-figsupp6-data1.zip › Figure 2-Figure Supplement 6-Source Data/Figure 2-Figure supplement 6 Source Data B/IMAGES FOR PANEL B.pdf]

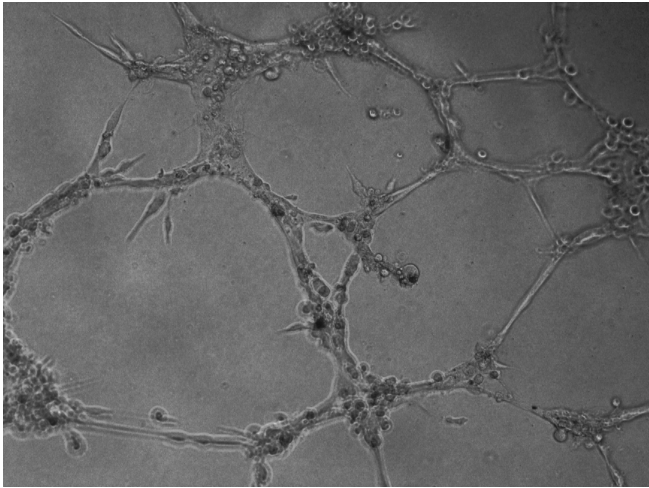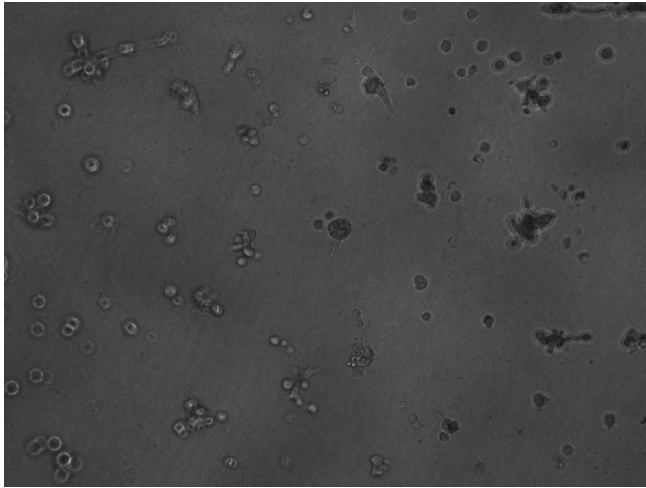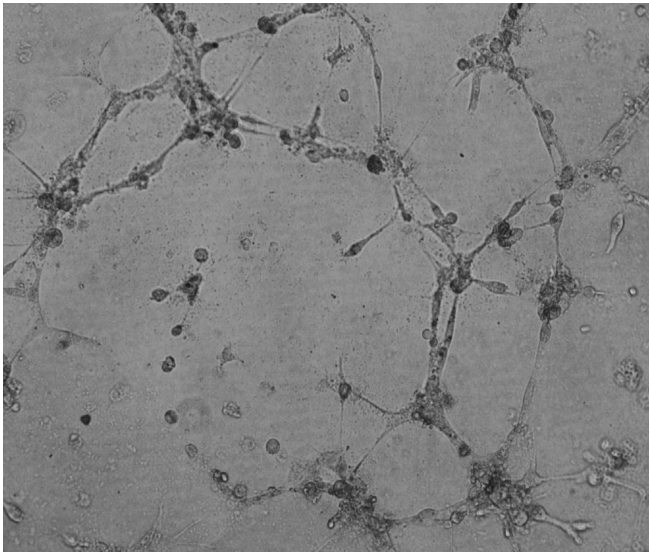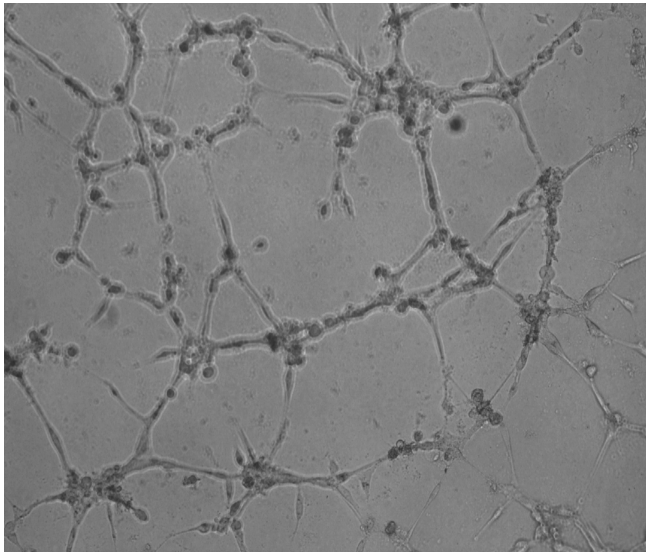

Supplement: Figure 3—source data 1. [file elife-78972-fig3-data1.zip › Figure 3-Source data A/IMAGES FOR PANEL A.pdf]

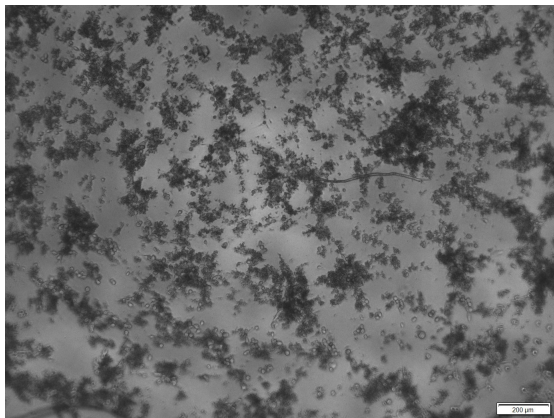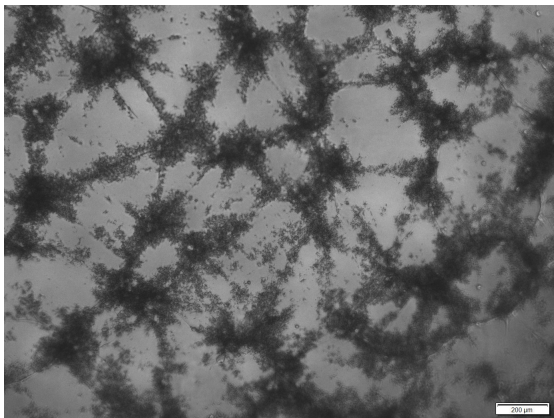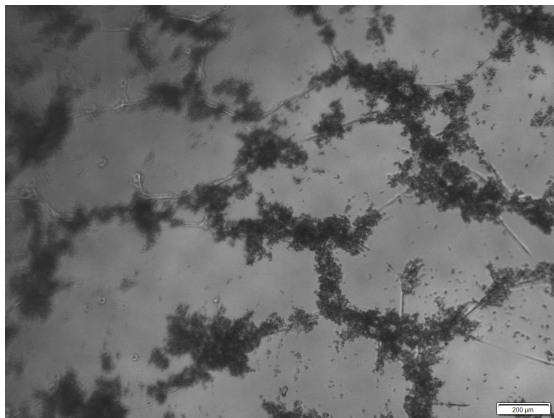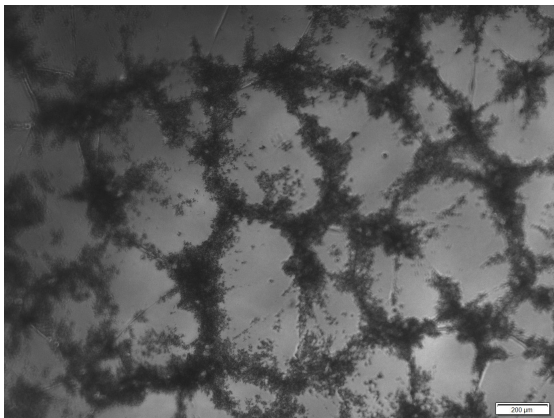

Supplement: Figure 3—source data 5. [file elife-78972-fig3-data5.zip › Figure 3-Source data E/IMAGES FOR PANEL E.pdf]

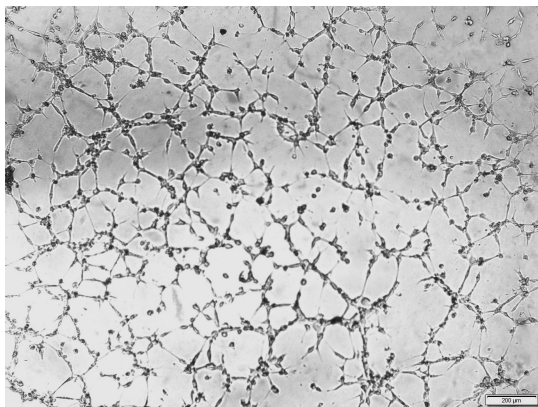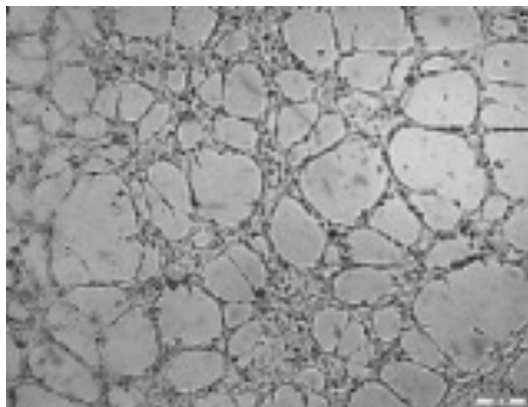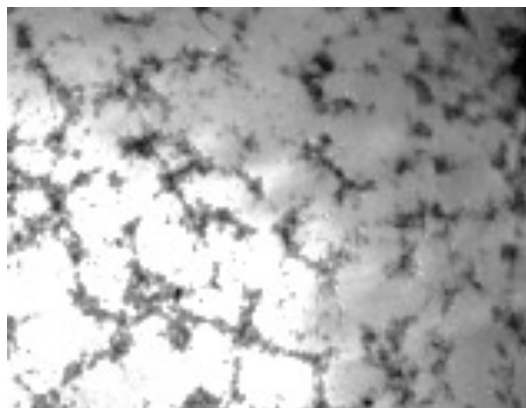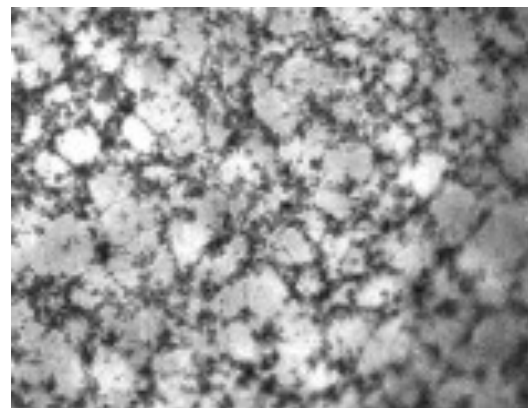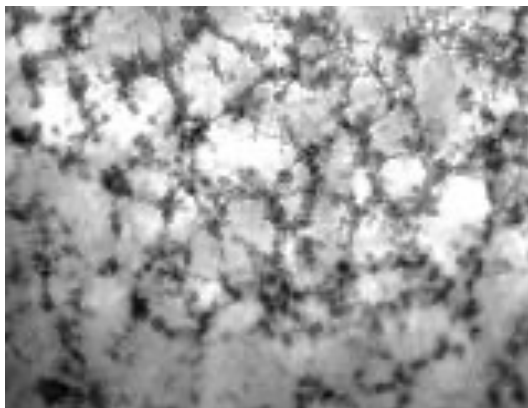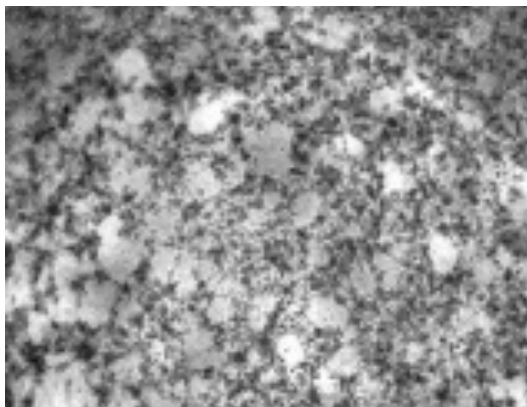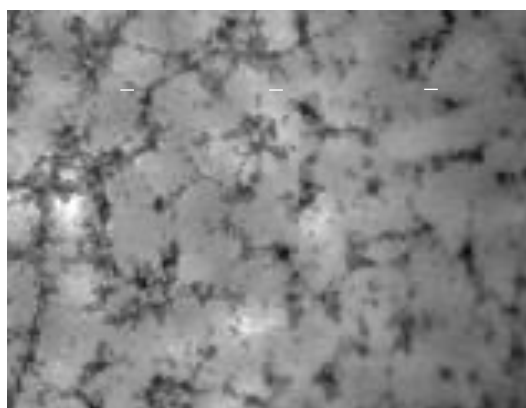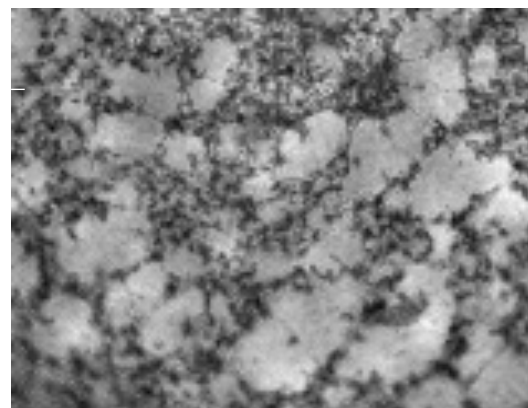

Supplement: Figure 3—source data 9. [file elife-78972-fig3-data9.zip › Figure 3-Source data I/IMAGES FOR PANEL I.pdf]

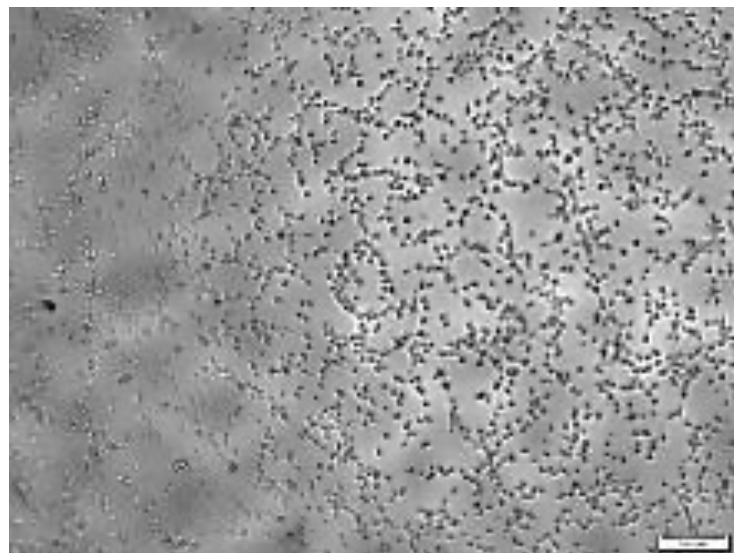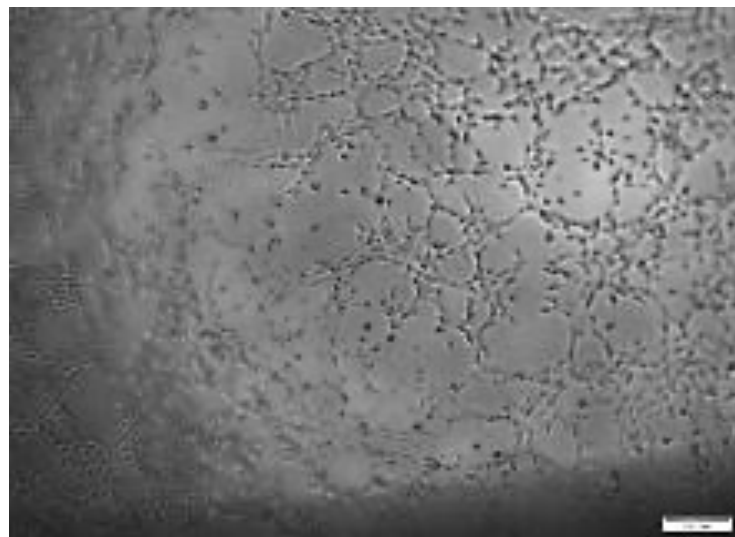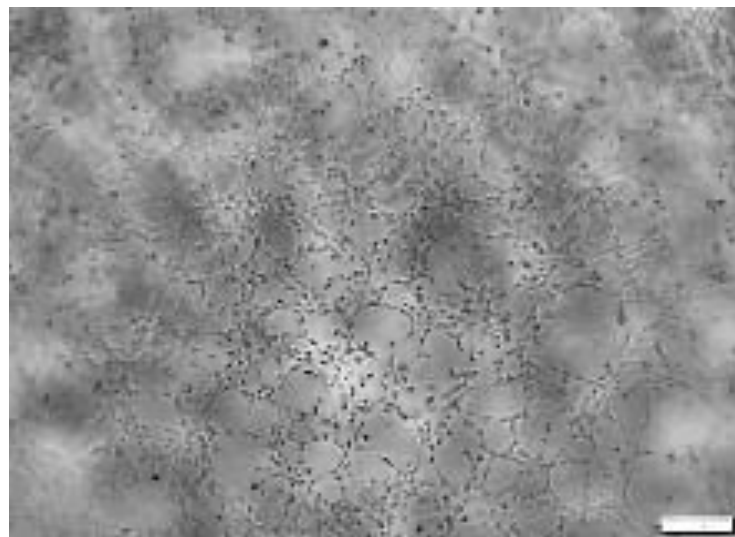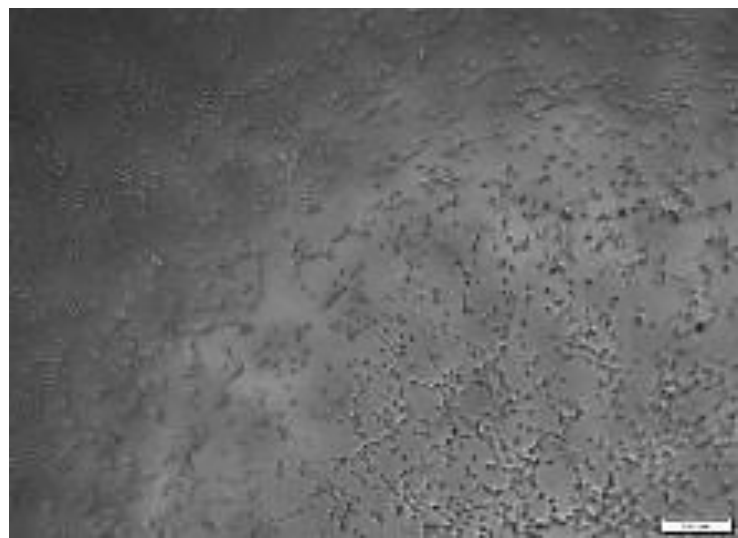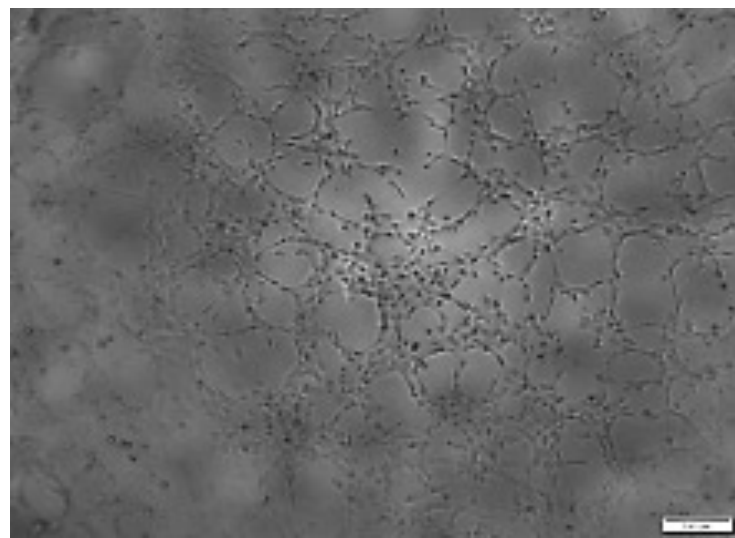

Supplement: Figure 3—figure supplement 1—source data 1. [file elife-78972-fig3-figsupp1-data1.zip › Figure 3-Figure Supplement 1 Source Data/Figure 3-Figure supplement 1 Source Data E/IMAGES FOR PANEL E.pdf]

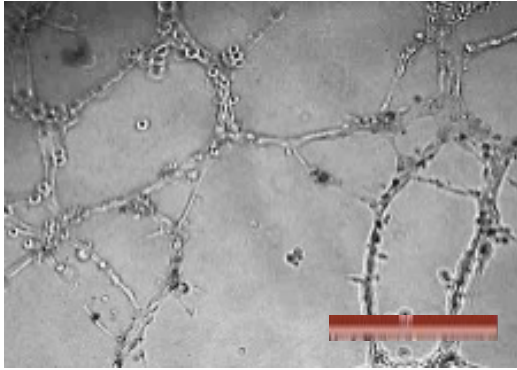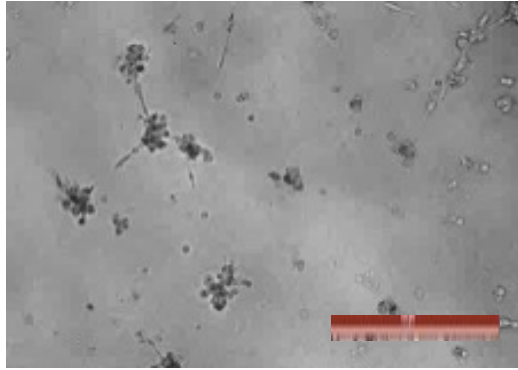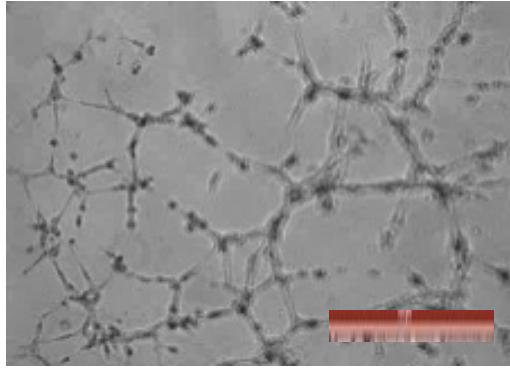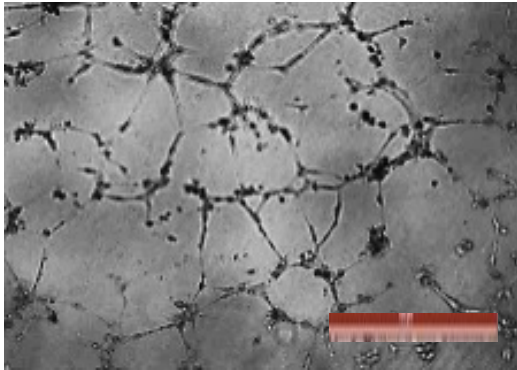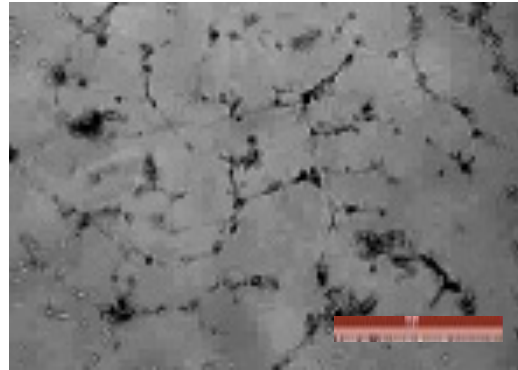

Supplement: Figure 3—figure supplement 1—source data 1. [file elife-78972-fig3-figsupp1-data1.zip › Figure 3-Figure Supplement 1 Source Data/Figure 3-Figure supplement 1 Source Data A/IMAGES FOR PANEL A.pdf]

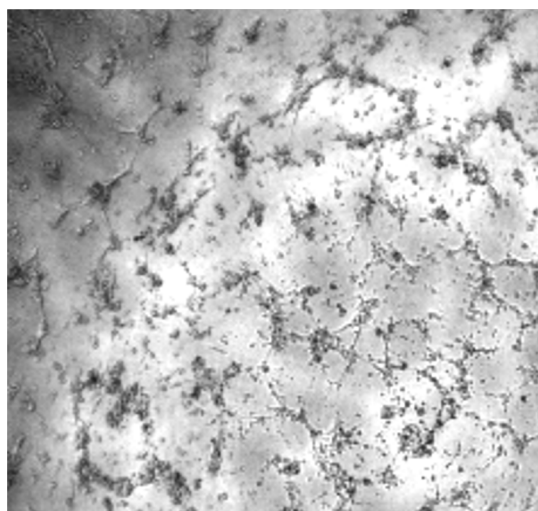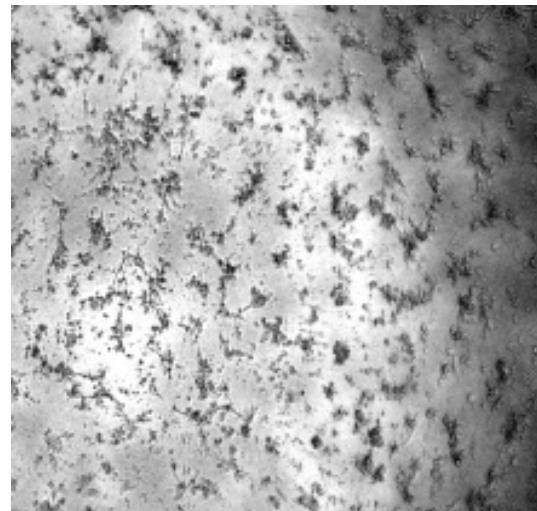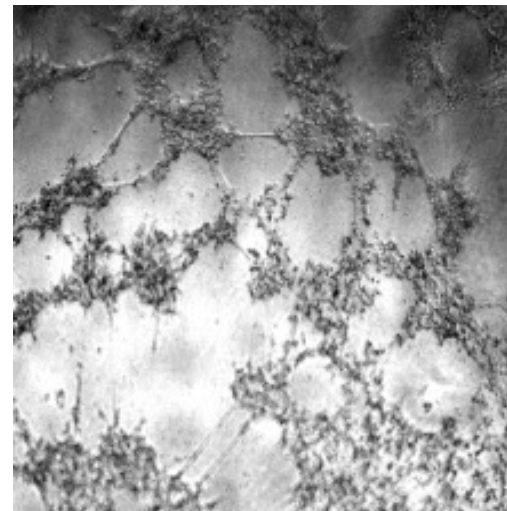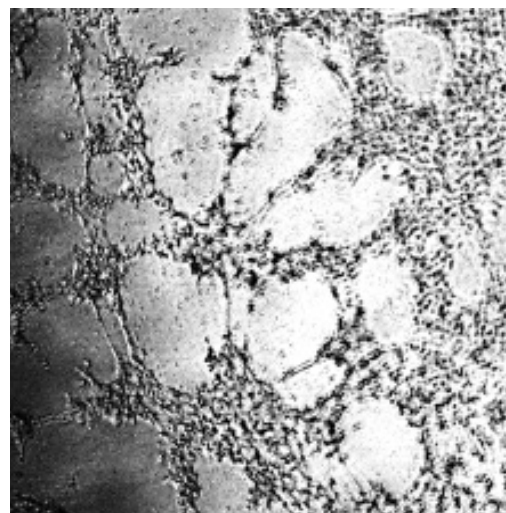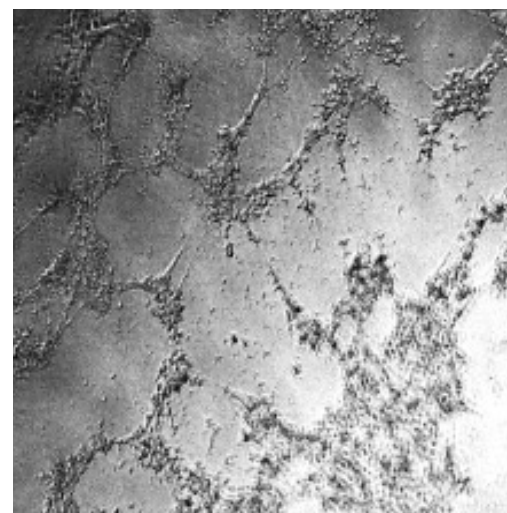

Supplement: Figure 3—figure supplement 1—source data 1. [file elife-78972-fig3-figsupp1-data1.zip › Figure 3-Figure Supplement 1 Source Data/Figure 3-Figure supplement 1 Source Data B/IMAGES FOR PANEL B.pdf]

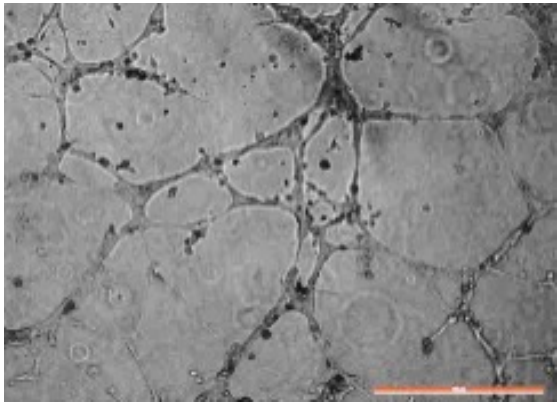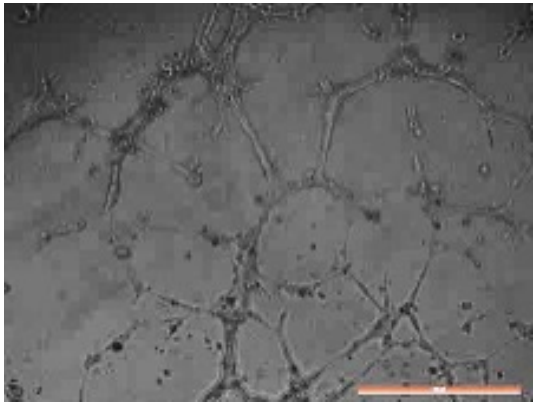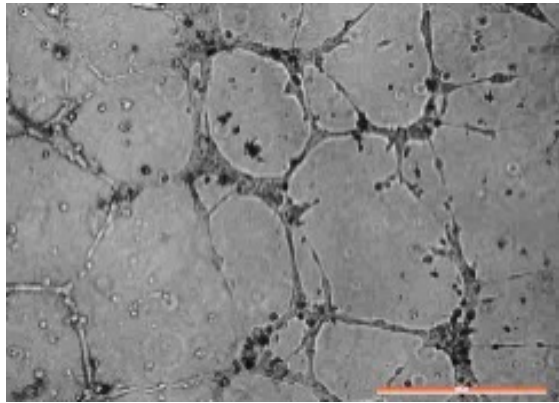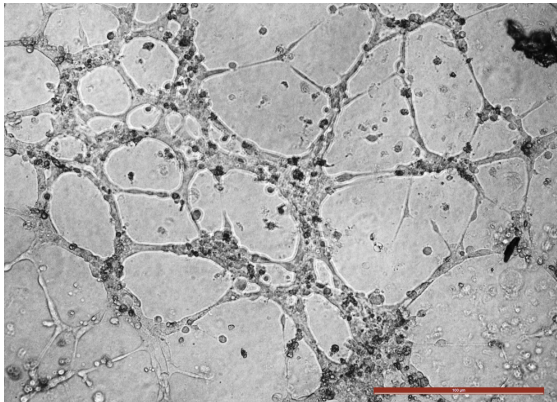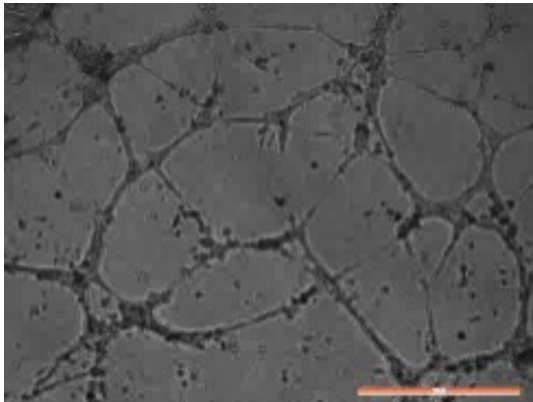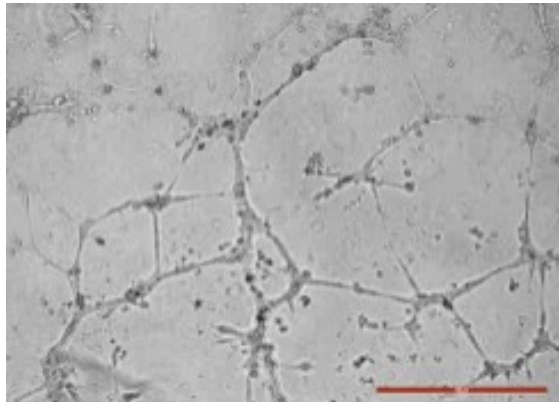

Supplement: Figure 3—figure supplement 1—source data 1. [file elife-78972-fig3-figsupp1-data1.zip › Figure 3-Figure Supplement 1 Source Data/Figure 3-Figure supplement 1 Source Data C/IMAGES FOR PANEL C.pdf]

**A**

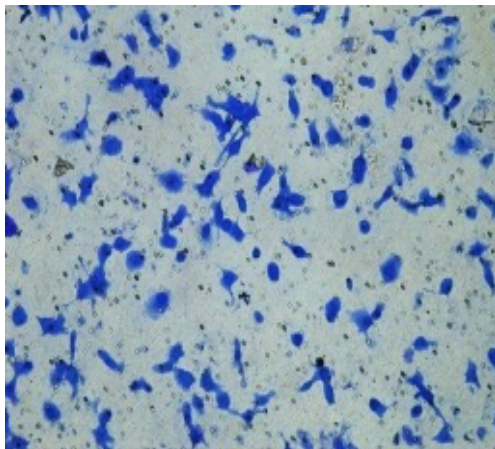

**B**

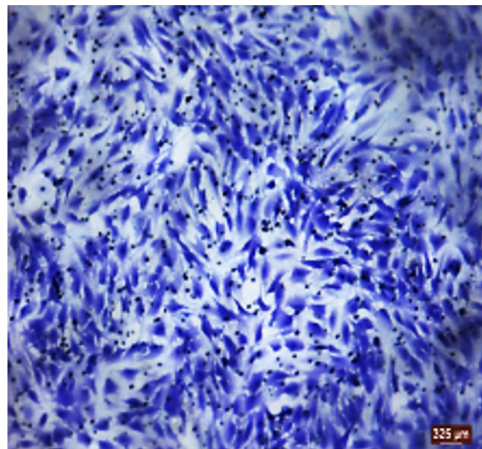

**C**

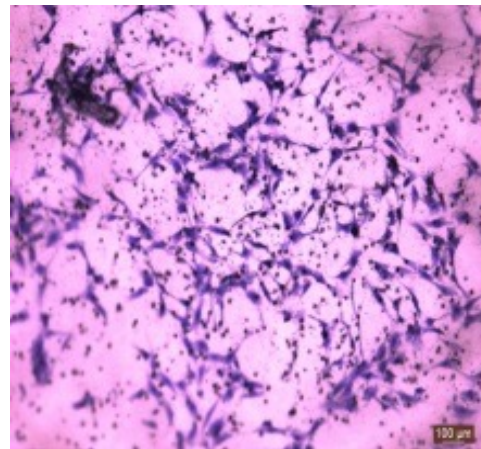

**D**

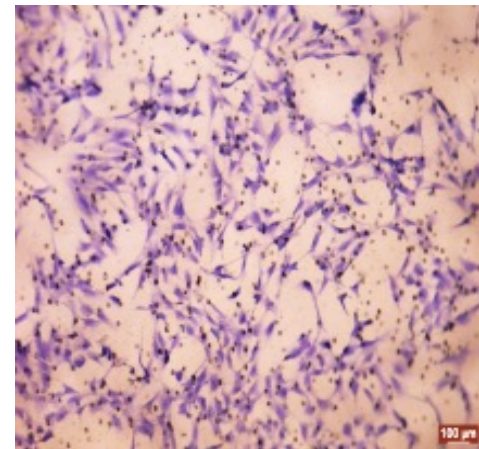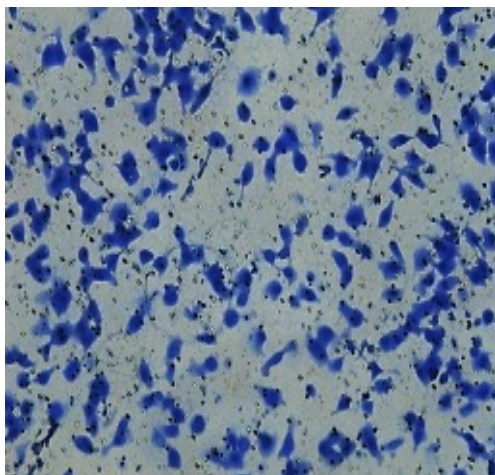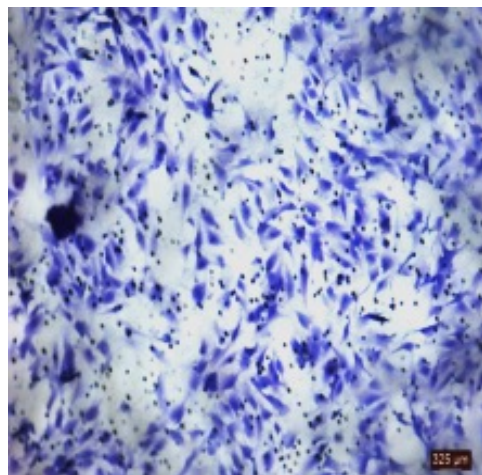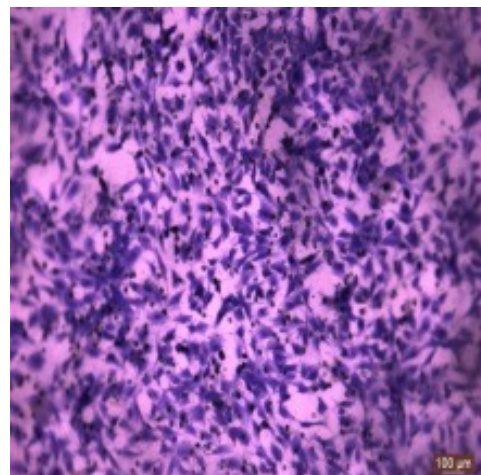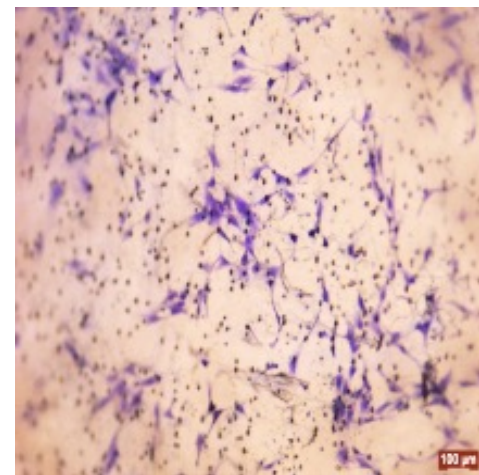

Supplement: Figure 3—figure supplement 2—source data 1. [file elife-78972-fig3-figsupp2-data1.zip › Figure 3-Figure Supplement 2 Source Data/Figure 3-figure supplement 2-Source Data A,B,C,D/IMAGES FOR PANELS A,B,C,D.pdf]

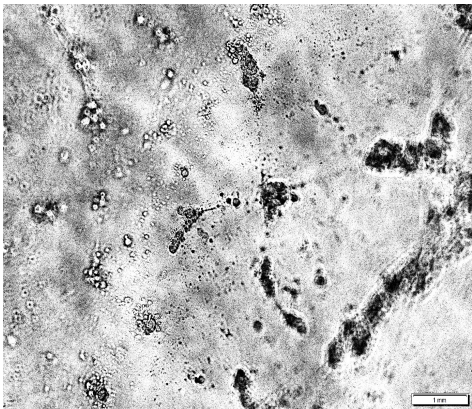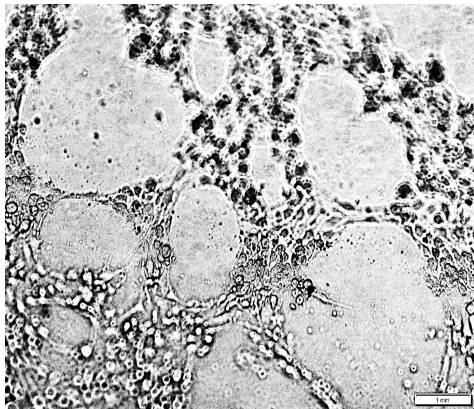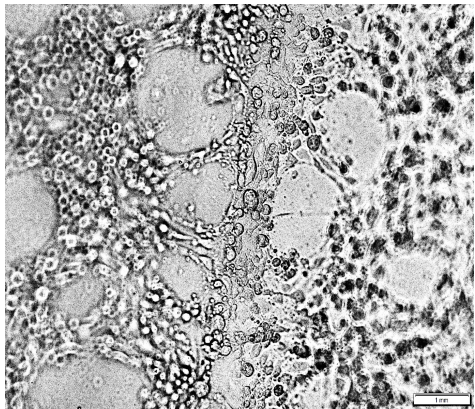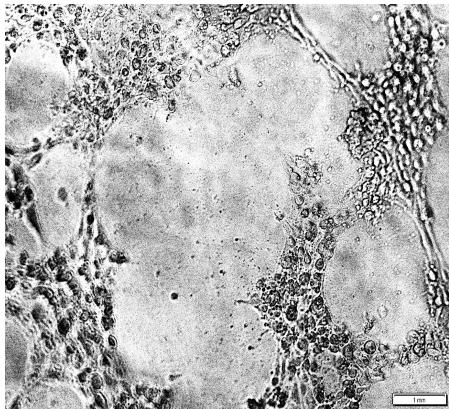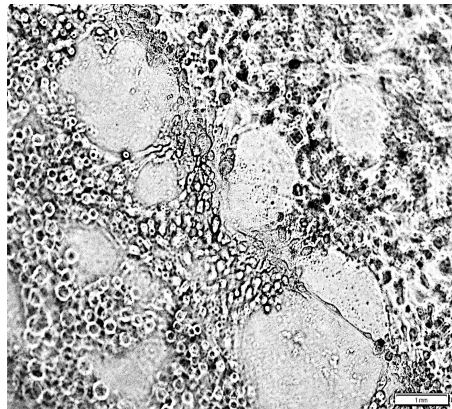

Supplement: Figure 3—figure supplement 2—source data 1. [file elife-78972-fig3-figsupp2-data1.zip › Figure 3-Figure Supplement 2 Source Data/Figure 3-figure supplement 2-Source Data F/IMAGES FOR PANEL F.pdf]

**B**

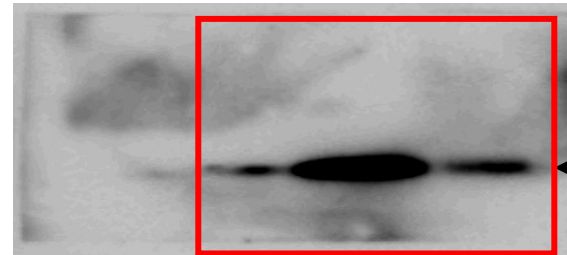

← **FMOD, 43 kDa**

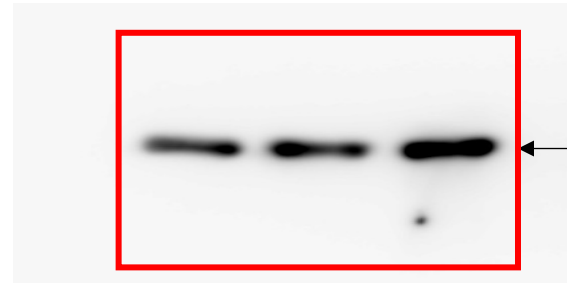

← **GAPDH, 36 kDa**

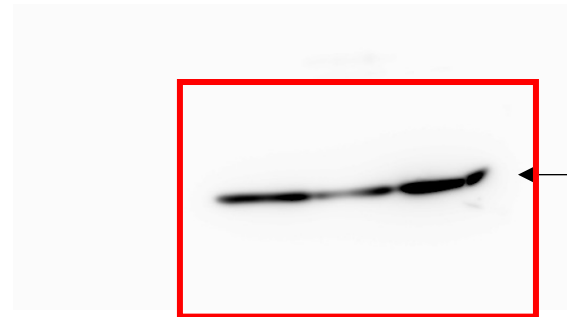

← **CD31, 135 kDa**

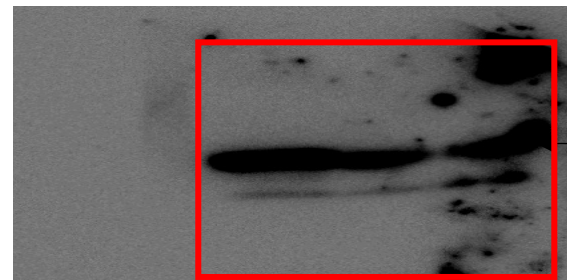

← **GAPDH, 36 kDa**

Supplement: Figure 3—figure supplement 3—source data 1. [file elife-78972-fig3-figsupp3-data1.zip › Figure 3-Figure Supplement 3 Source Data/Figure 3-figure supplement 3-Source Data B/BLOTS FOR PANEL B.pdf]

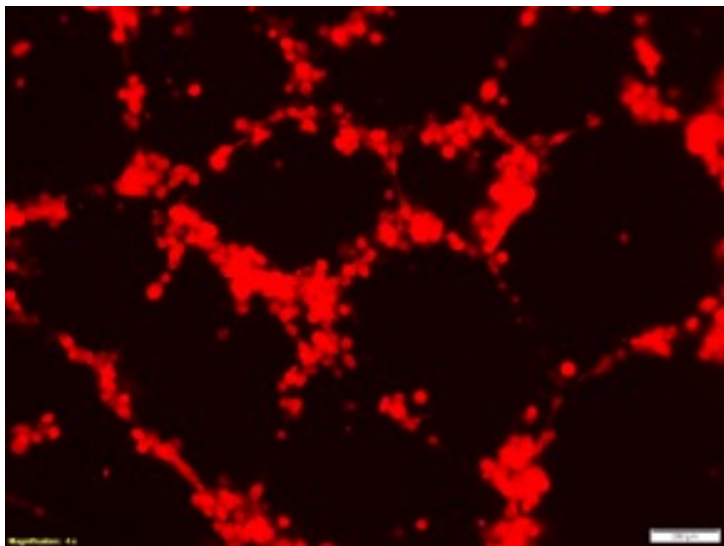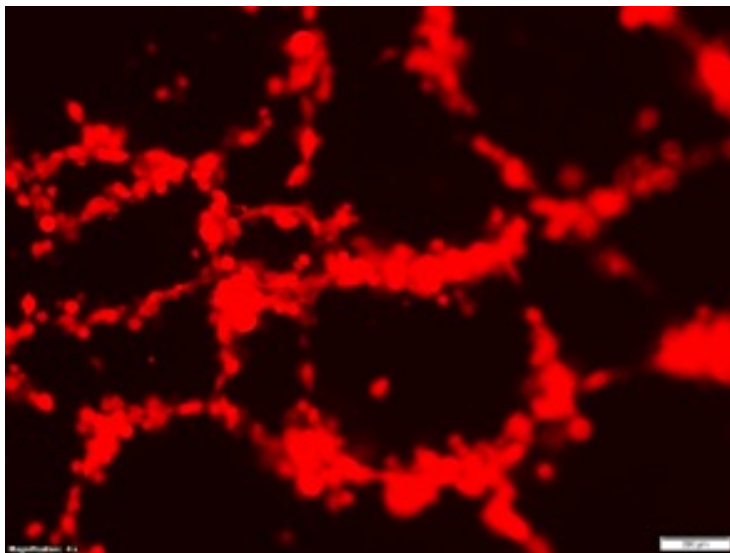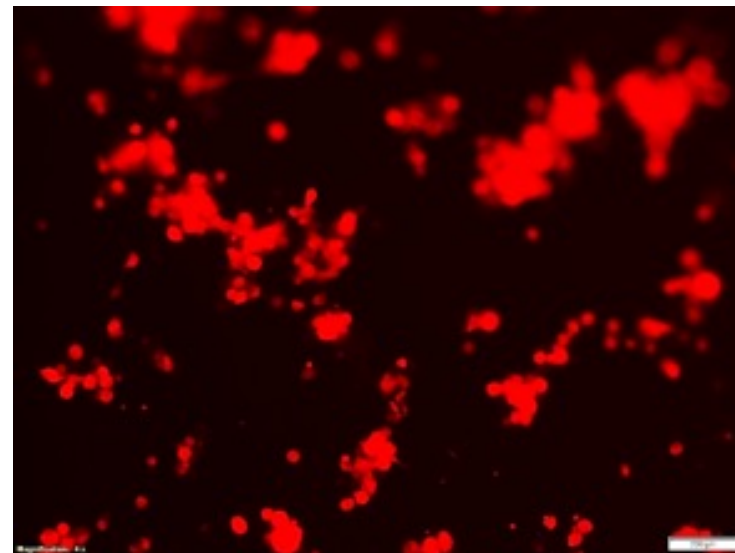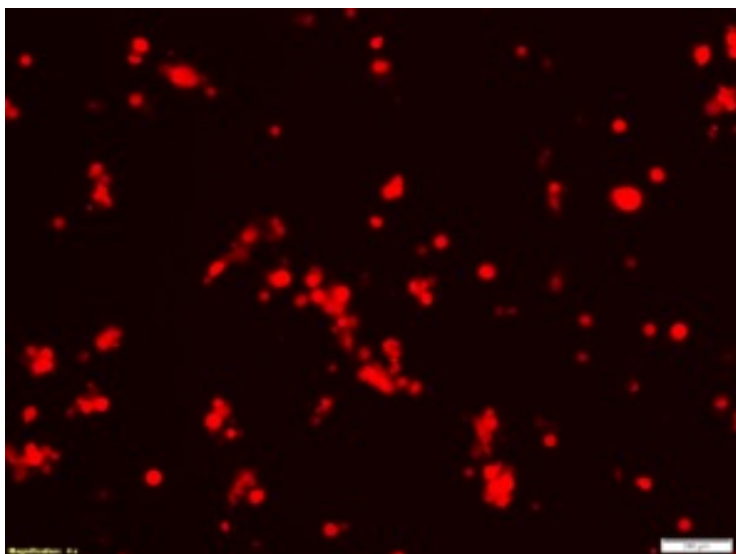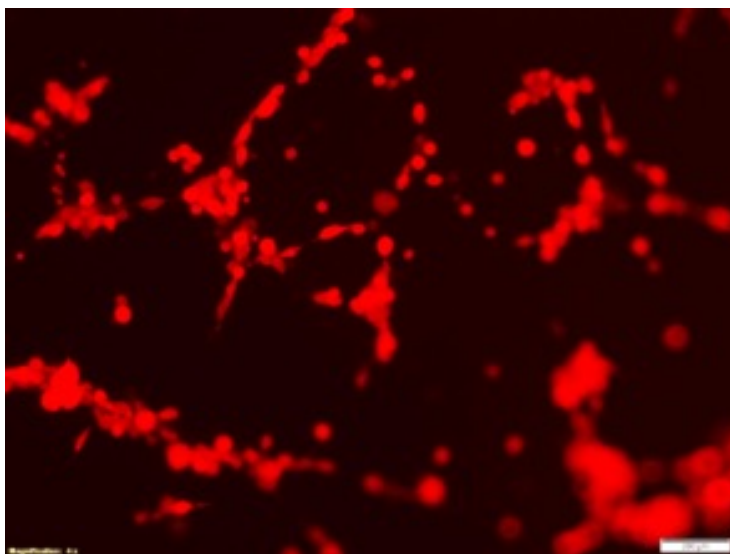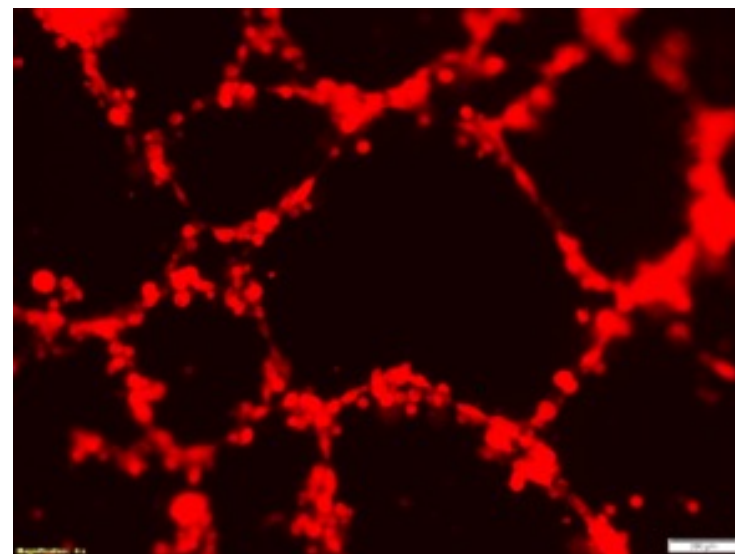

Supplement: Figure 3—figure supplement 3—source data 1. [file elife-78972-fig3-figsupp3-data1.zip › Figure 3-Figure Supplement 3 Source Data/Figure 3-figure supplement 3-Source Data C/IMAGES FOR PANEL C.pdf]

**Figure 4**

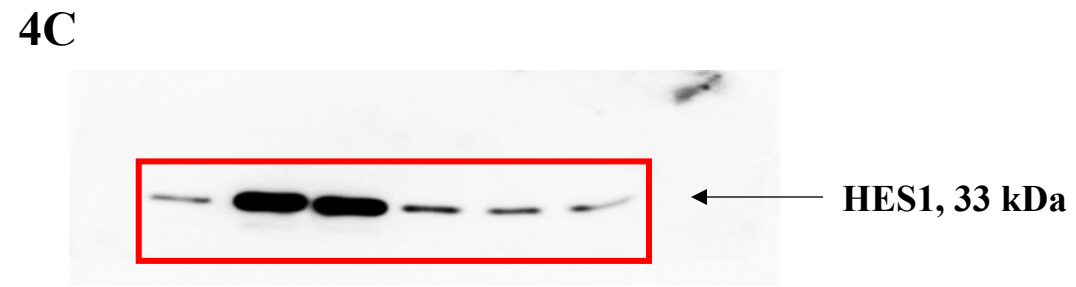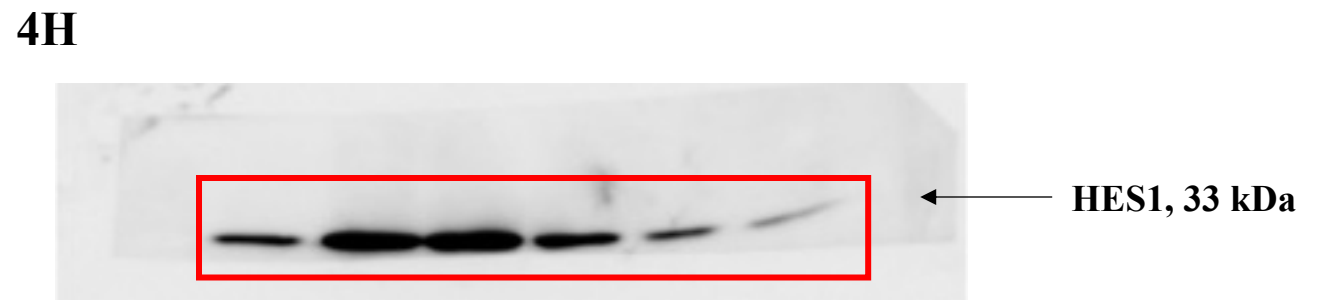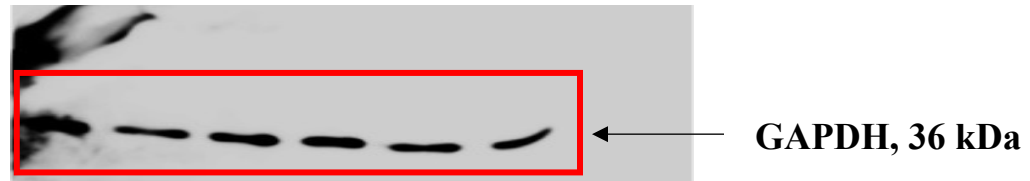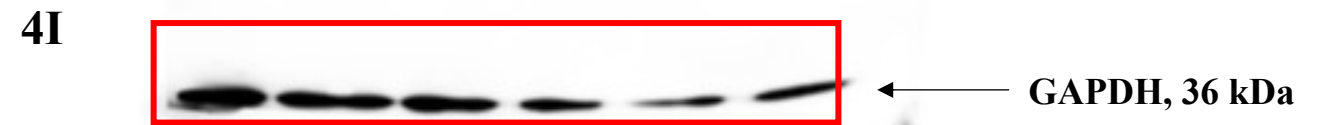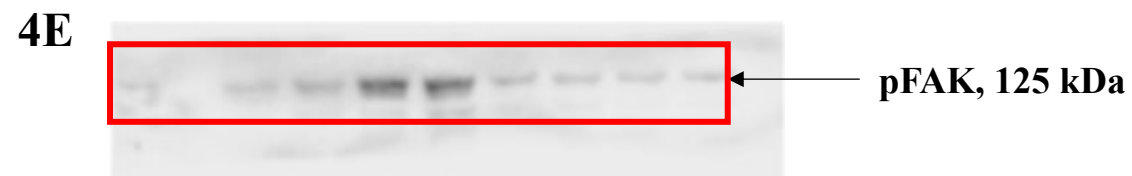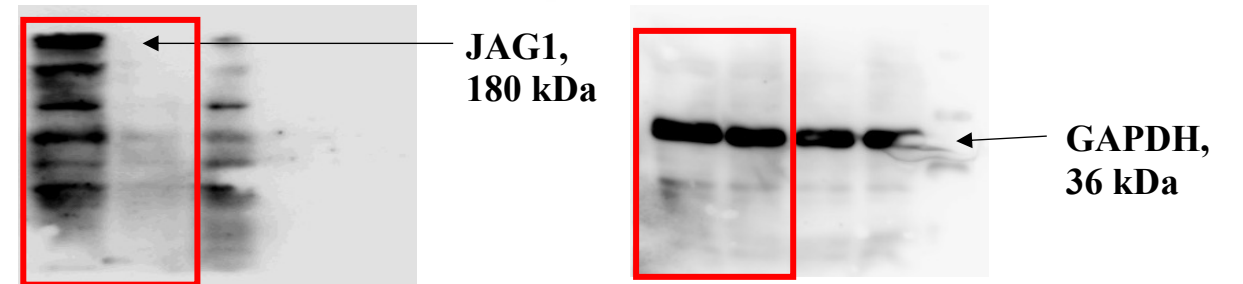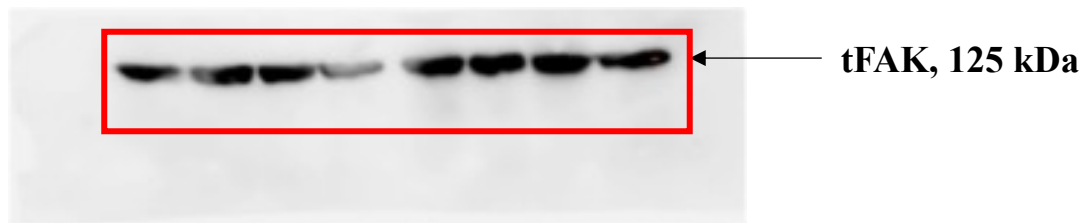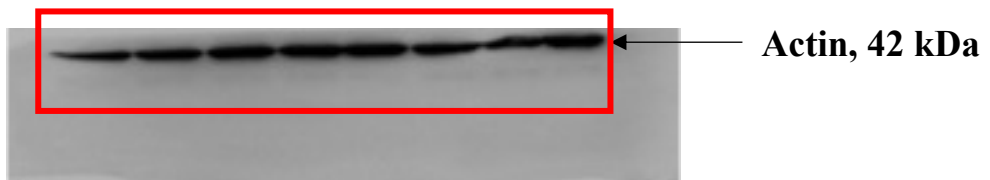

Supplement: Figure 4—source data 3. [file elife-78972-fig4-data3.zip › Figure 4-Source data C,E,H,I/BLOTS FOR C,E,H,I.pdf]

**Figure 4**

**4K**

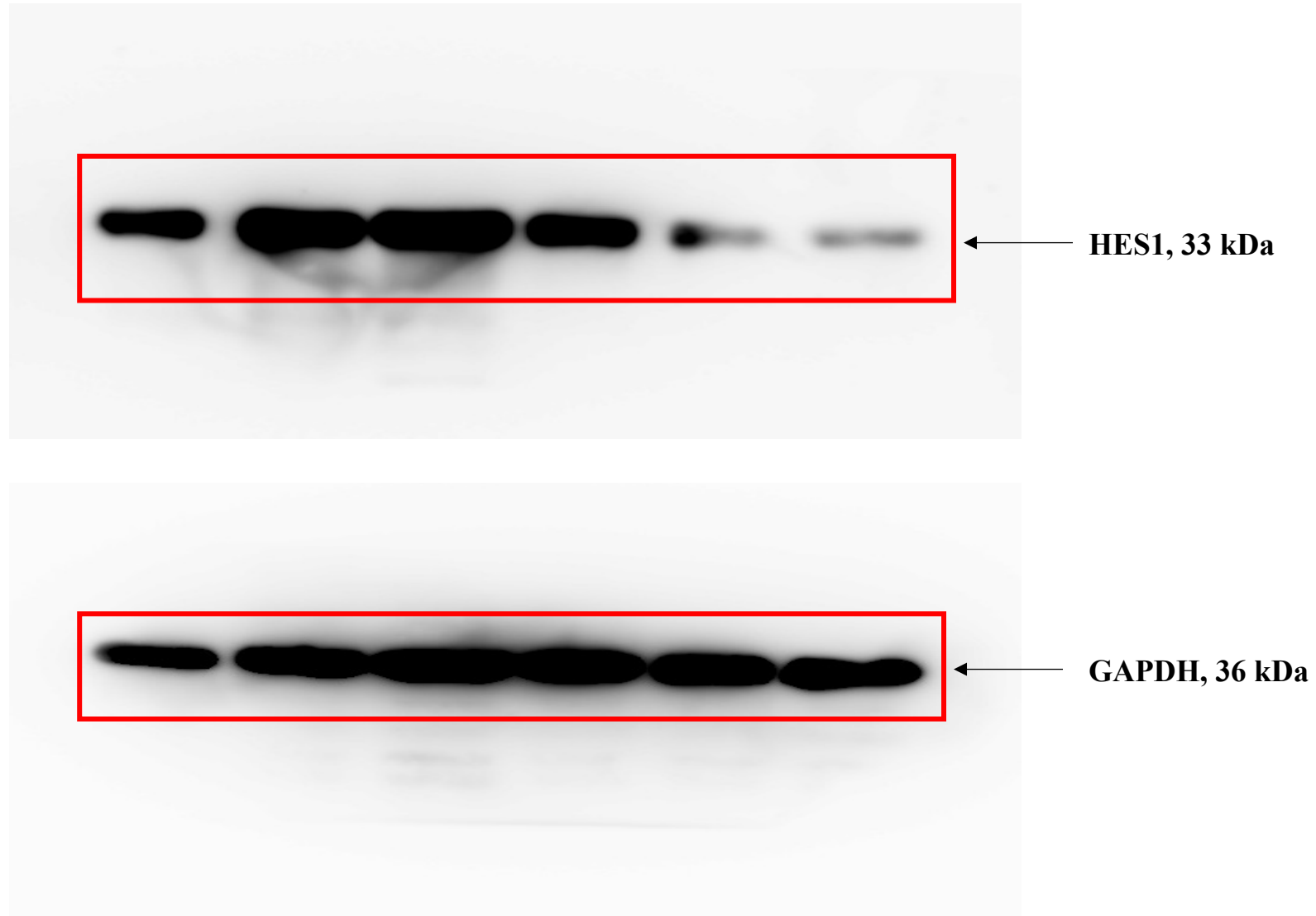

Supplement: Figure 4—source data 8. [file elife-78972-fig4-data8.zip › Figure 4-Source data K/BLOTS FOR PANEL K.pdf]

**Supplementary Figure 16**

**16 C**

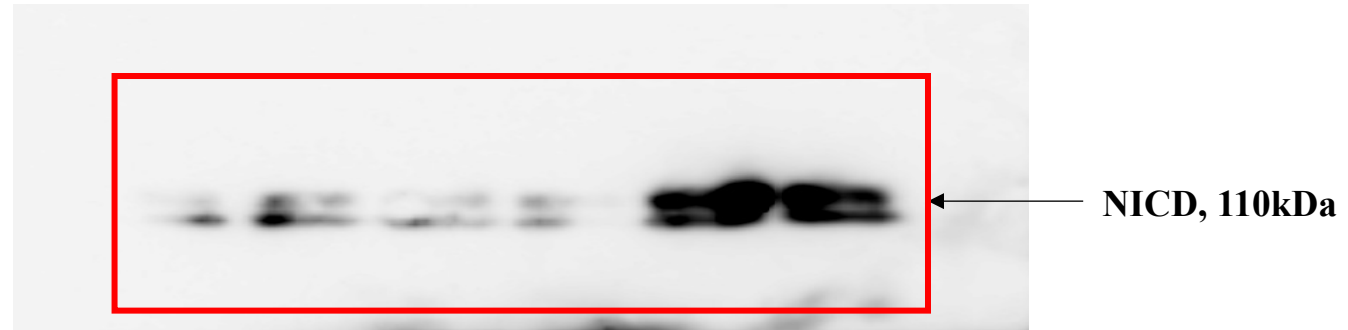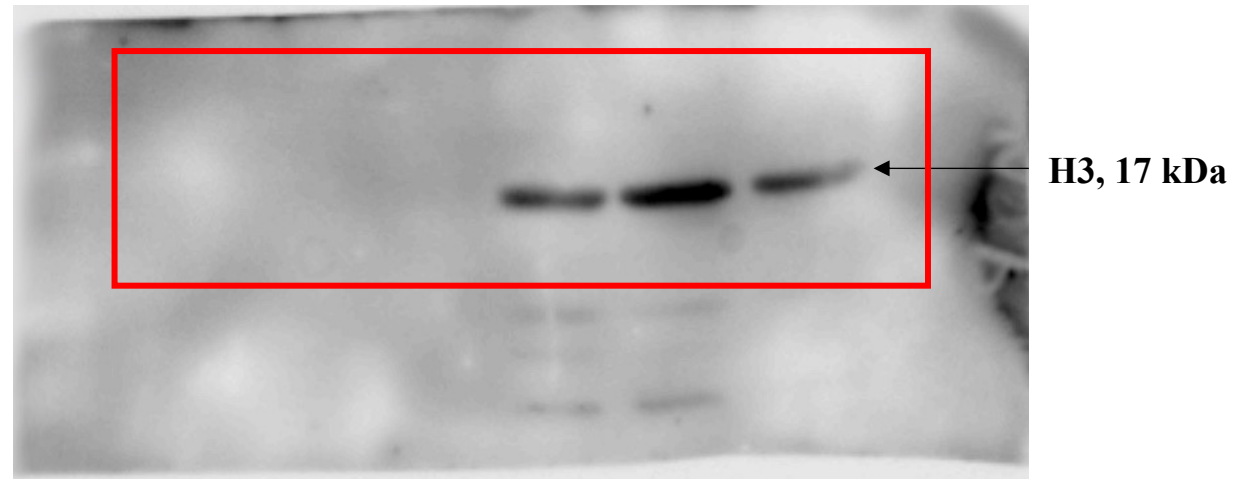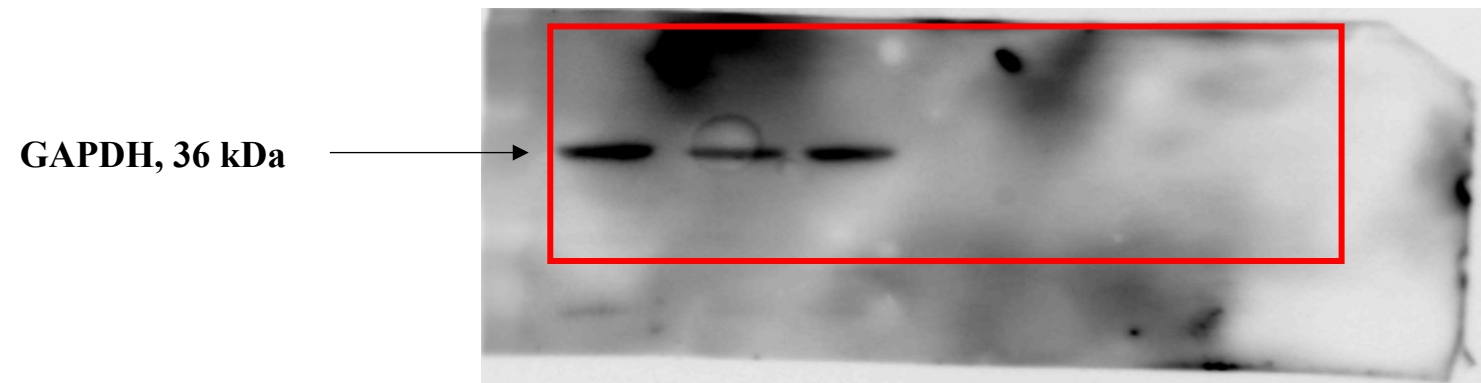

Supplement: Figure 4—figure supplement 1—source data 1. [file elife-78972-fig4-figsupp1-data1.zip › Figure 4-Figure Supplement 1-Source Data/Figure 4-Figure Supplement 1-Source Data C/BLOTS FOR PANEL C.pdf]

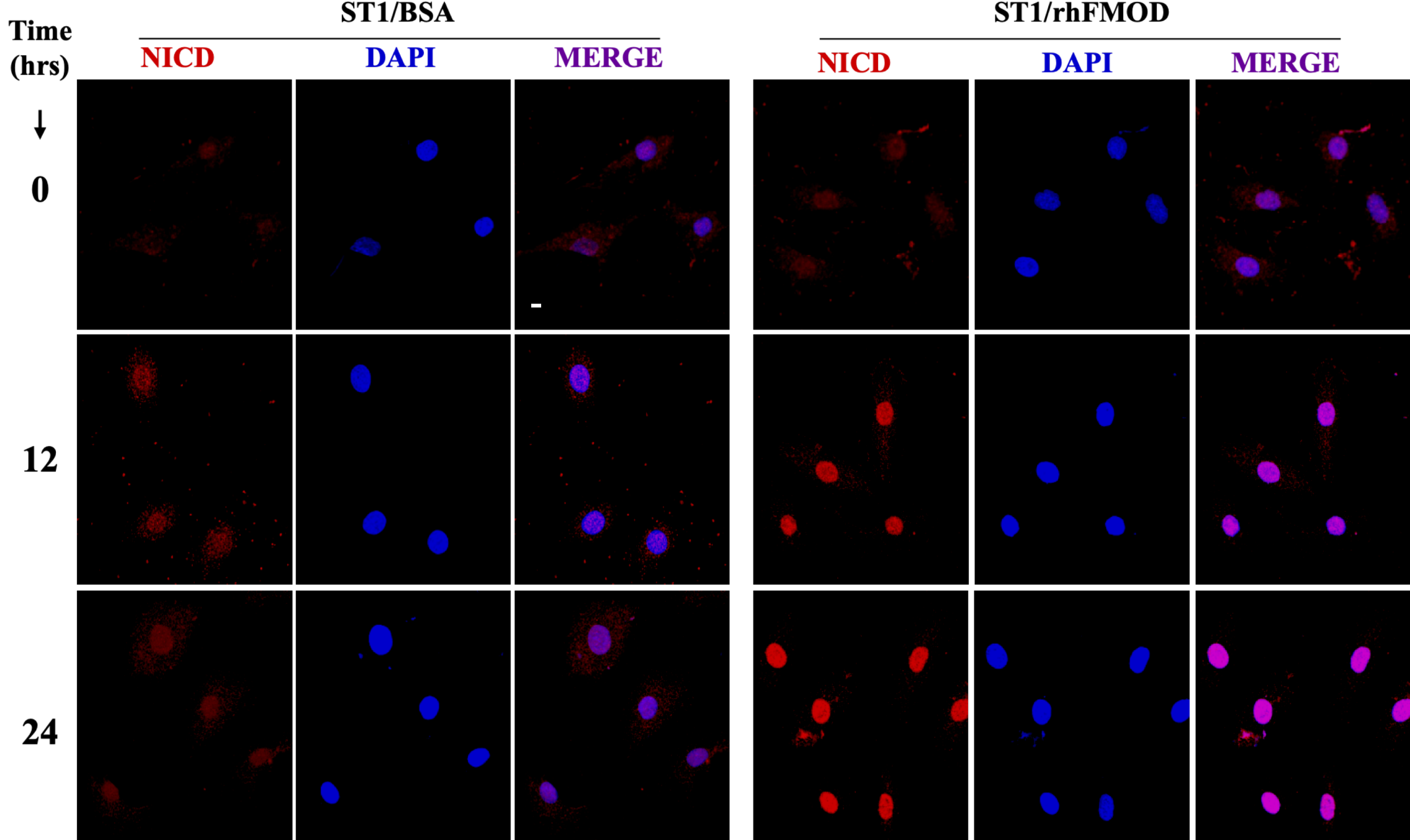

Supplement: Figure 4—figure supplement 1—source data 1. [file elife-78972-fig4-figsupp1-data1.zip › Figure 4-Figure Supplement 1-Source Data/Figure 4-Figure Supplement 1-Source Data D/IMAGES FOR PANEL D.pdf]

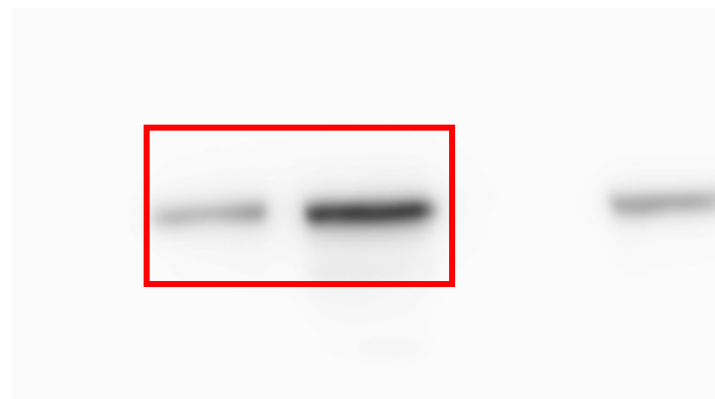

← **NICD, 110 kDa**

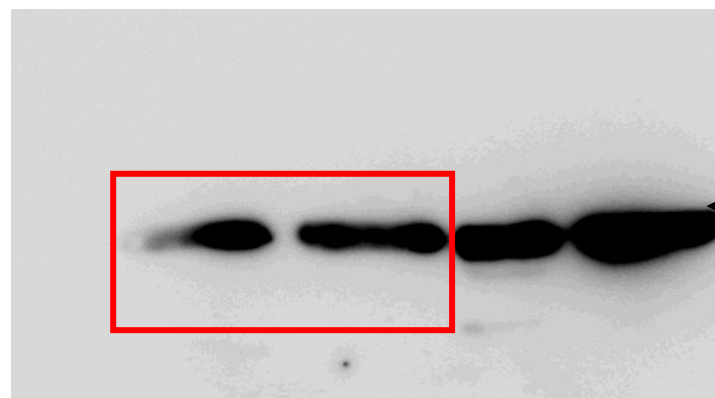

← **GAPDH, 36 kDa**

Supplement: Figure 4—figure supplement 2—source data 1. [file elife-78972-fig4-figsupp2-data1.zip › Figure 4-Figure Supplement 2-Source Data/Figure 4-Figure Supplement 2-Source Data A/BLOTS FOR PANEL A.pdf]

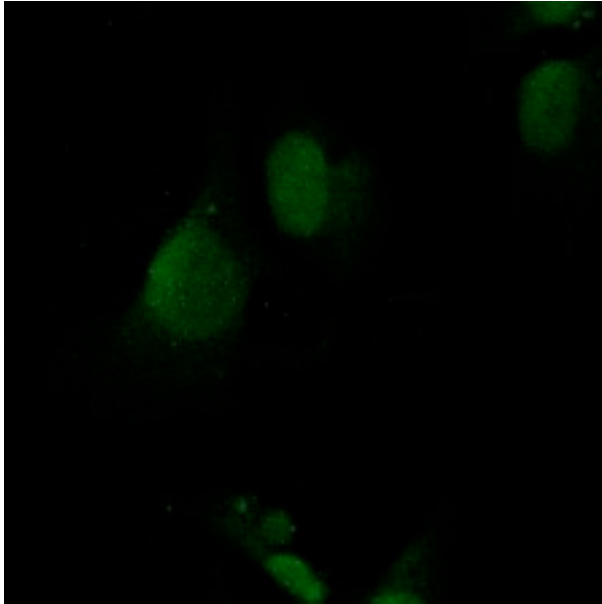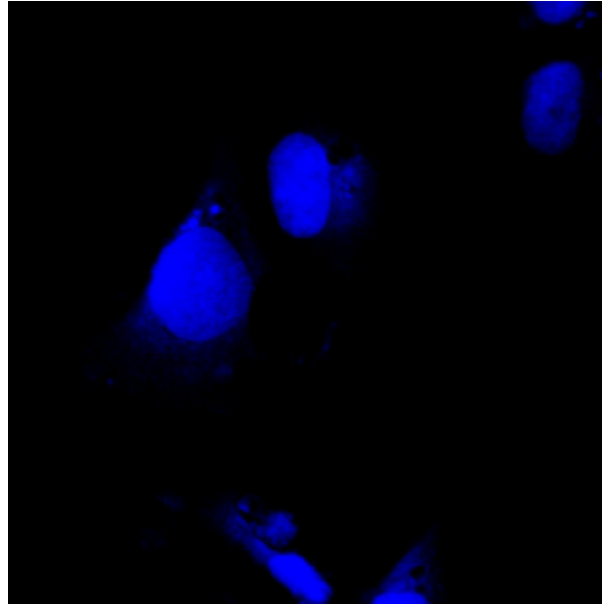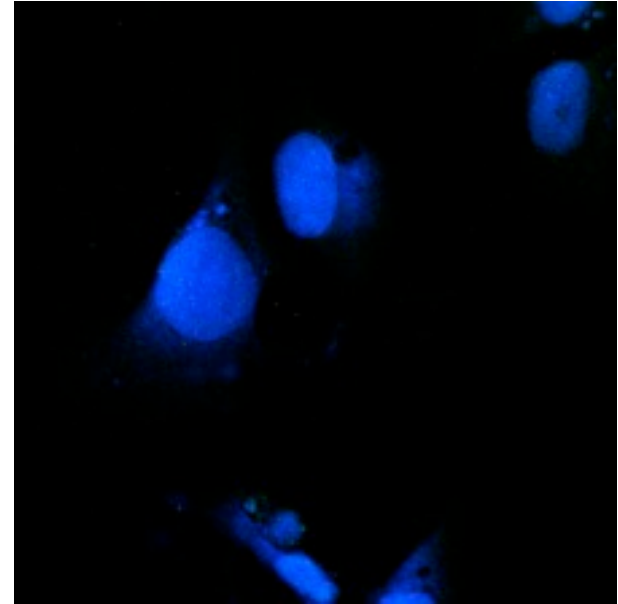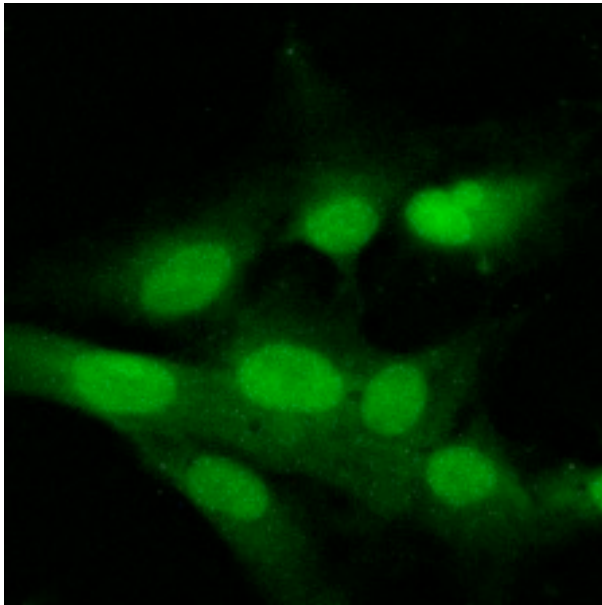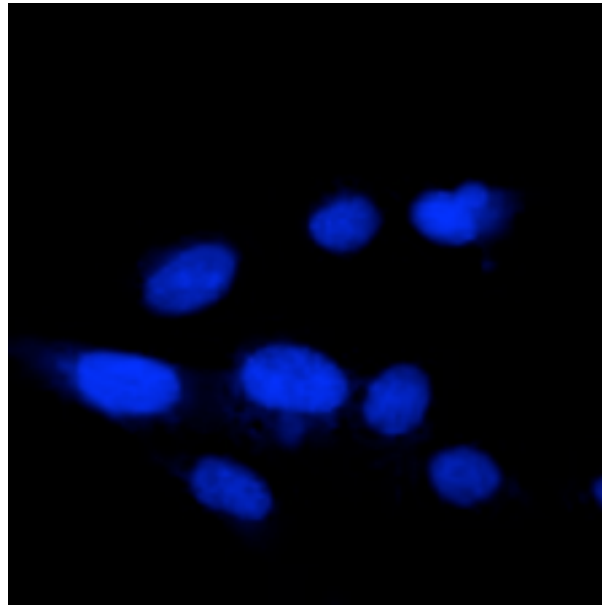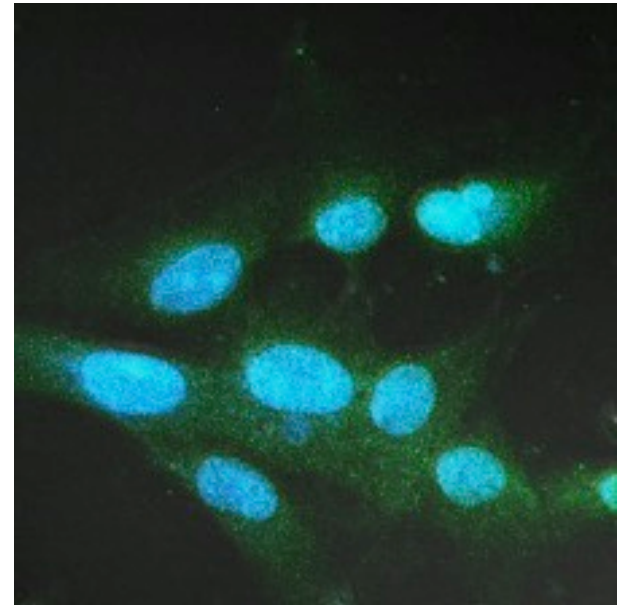

Supplement: Figure 4—figure supplement 2—source data 1. [file elife-78972-fig4-figsupp2-data1.zip › Figure 4-Figure Supplement 2-Source Data/Figure 4-Figure Supplement 2-Source Data B/IMAGES FOR PANEL B.pdf]

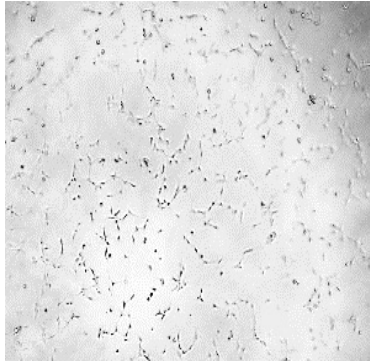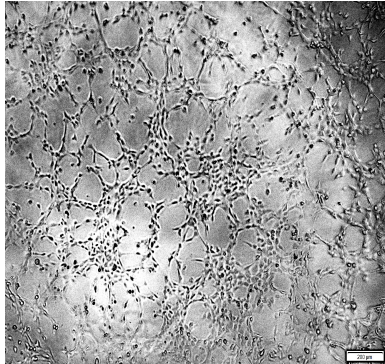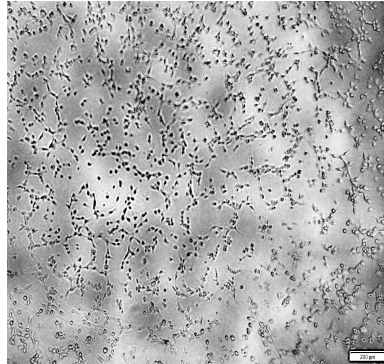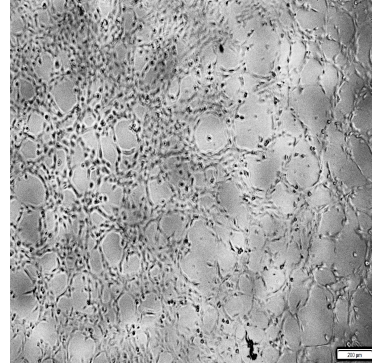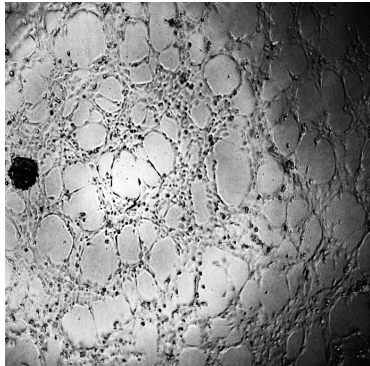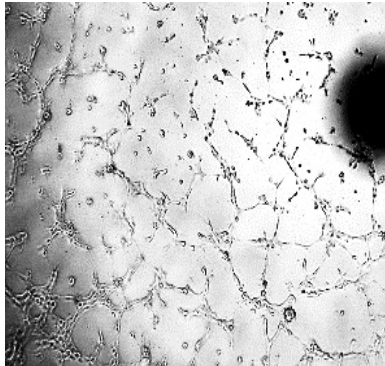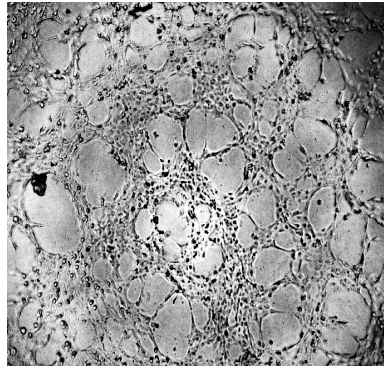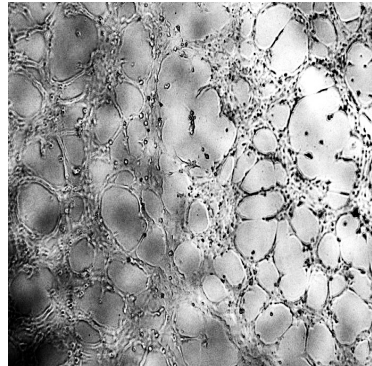

Supplement: Figure 4—figure supplement 2—source data 1. [file elife-78972-fig4-figsupp2-data1.zip › Figure 4-Figure Supplement 2-Source Data/Figure 4-Figure Supplement 2-Source Data C/IMAGES FOR PANEL C.pdf]

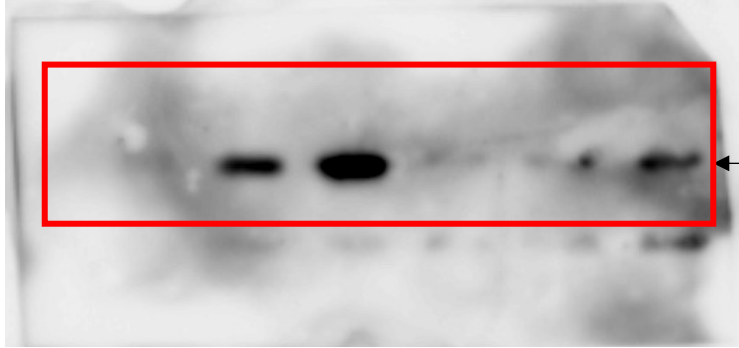

← **HES1, 33 kDa**

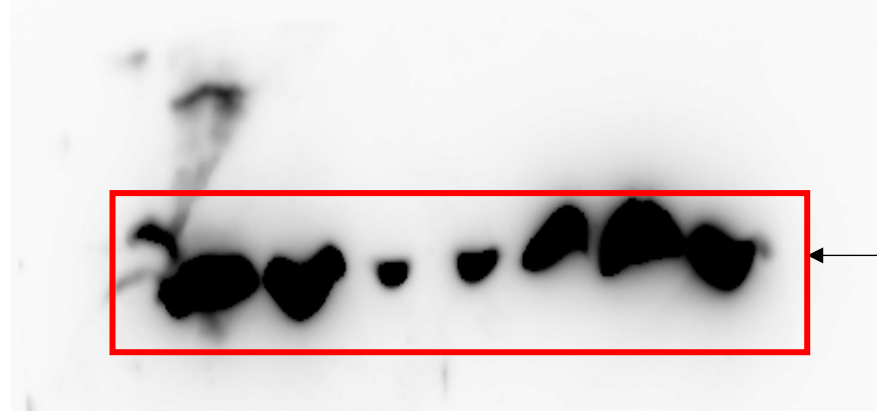

← **HES1, 33 kDa**

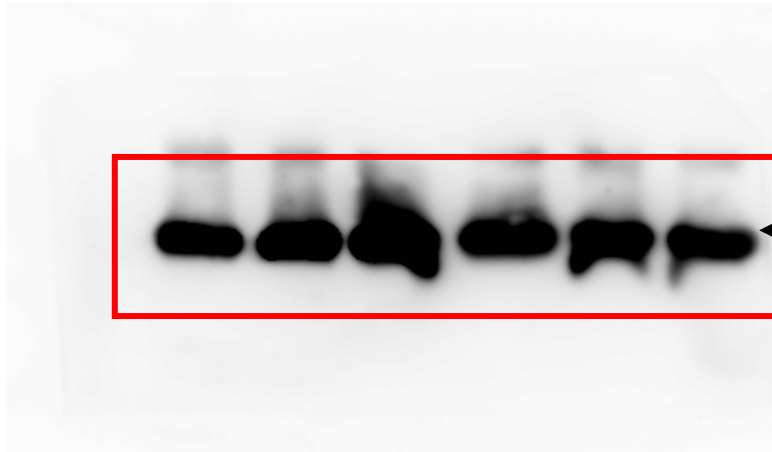

← **GAPDH,  
36 kDa**

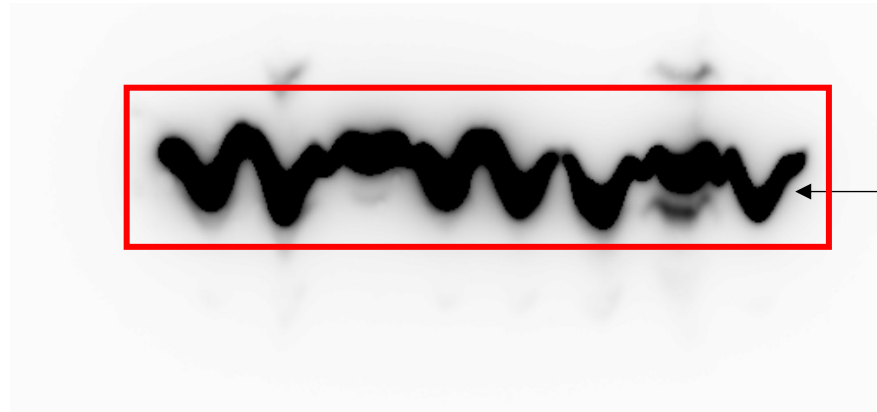

← **GAPDH, 36 kDa**

Supplement: Figure 4—figure supplement 4—source data 1. [file elife-78972-fig4-figsupp4-data1.zip › Figure 4-Figure Supplement 4-Source Data/Figure 4-Figure Supplement 4-Source Data H/RAW BLOTS.pdf]

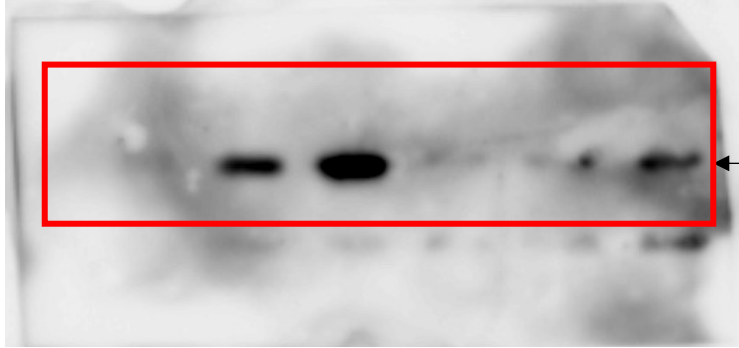

← **HES1, 33 kDa**

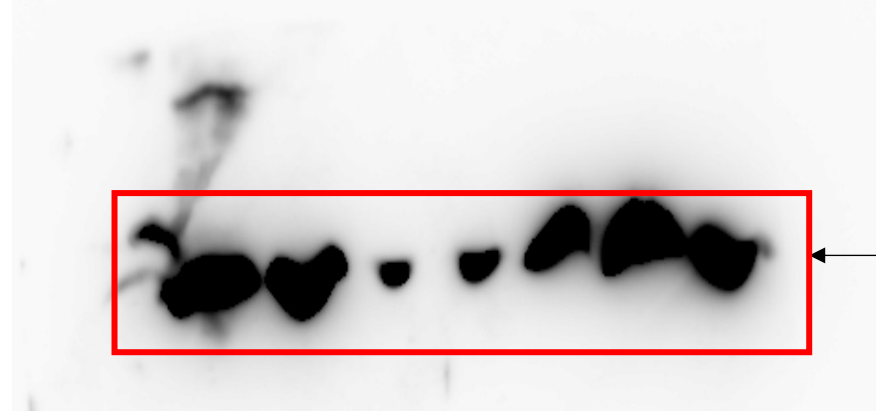

← **HES1, 33 kDa**

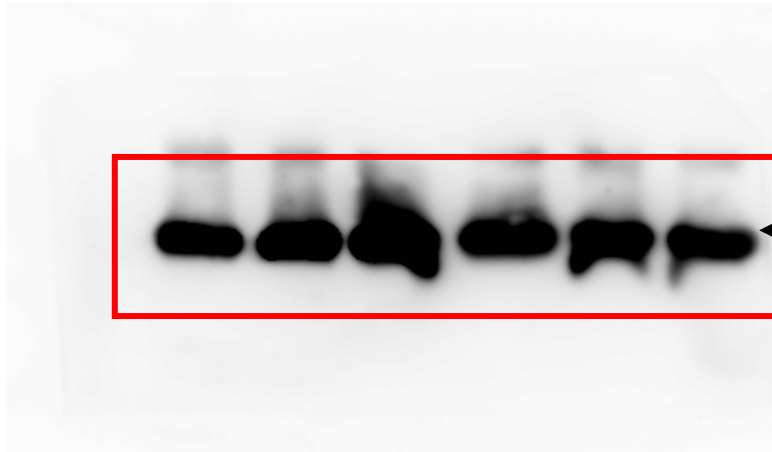

← **GAPDH,  
36 kDa**

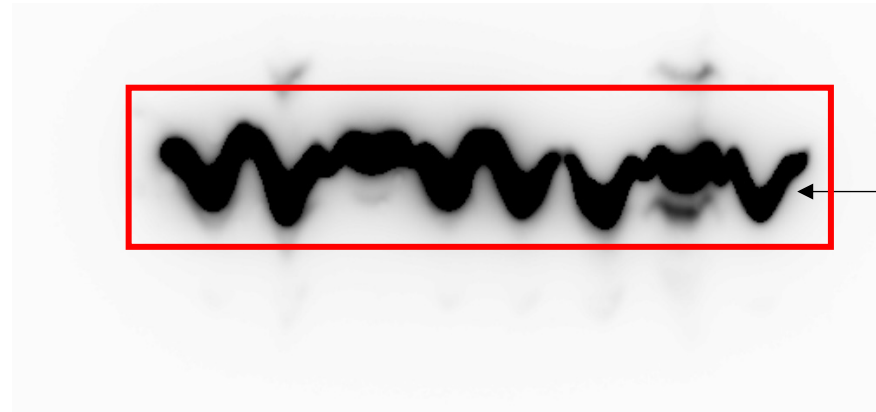

← **GAPDH, 36 kDa**

Supplement: Figure 4—figure supplement 4—source data 1. [file elife-78972-fig4-figsupp4-data1.zip › Figure 4-Figure Supplement 4-Source Data/Figure 4-Figure Supplement 4-Source Data/RAW BLOTS.pdf]

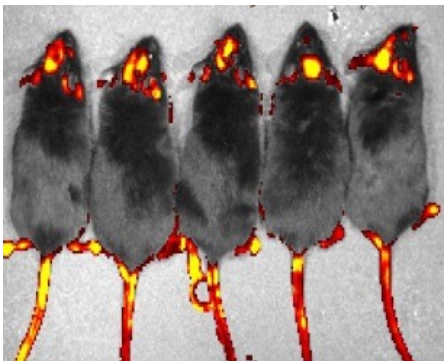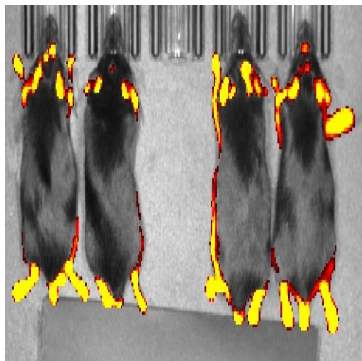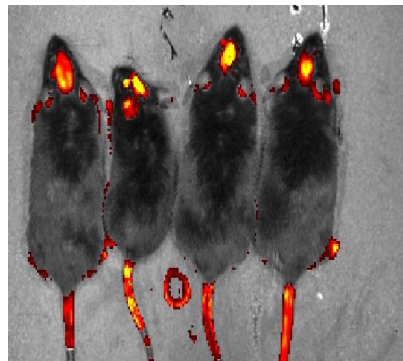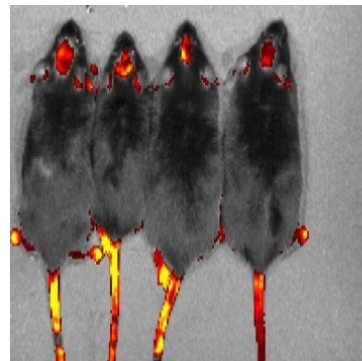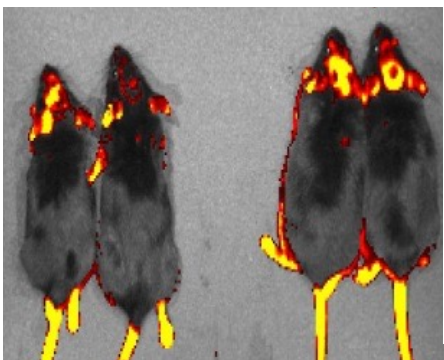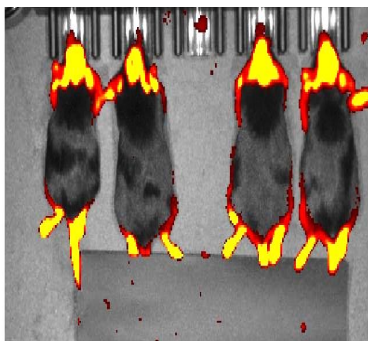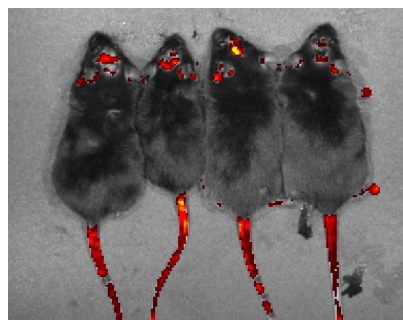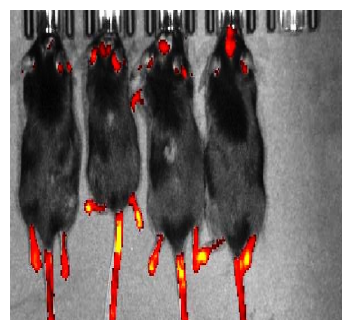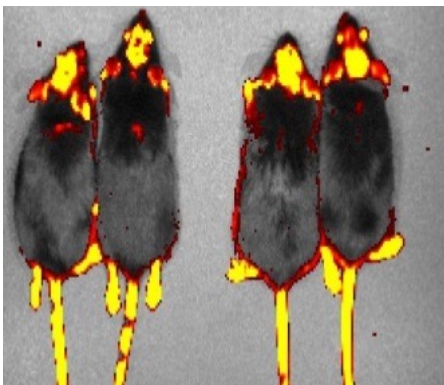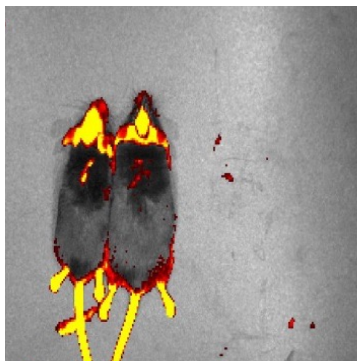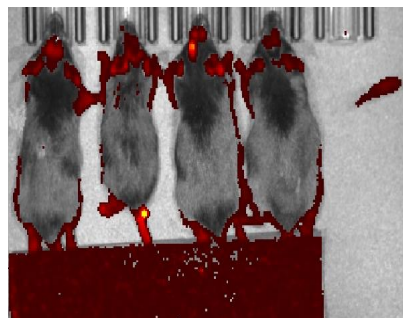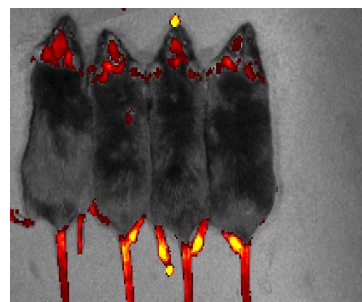

Supplement: Figure 5—source data 1. [file elife-78972-fig5-data1.zip › Figure 5-Source data B/IMAGES FOR PANEL B.pdf]

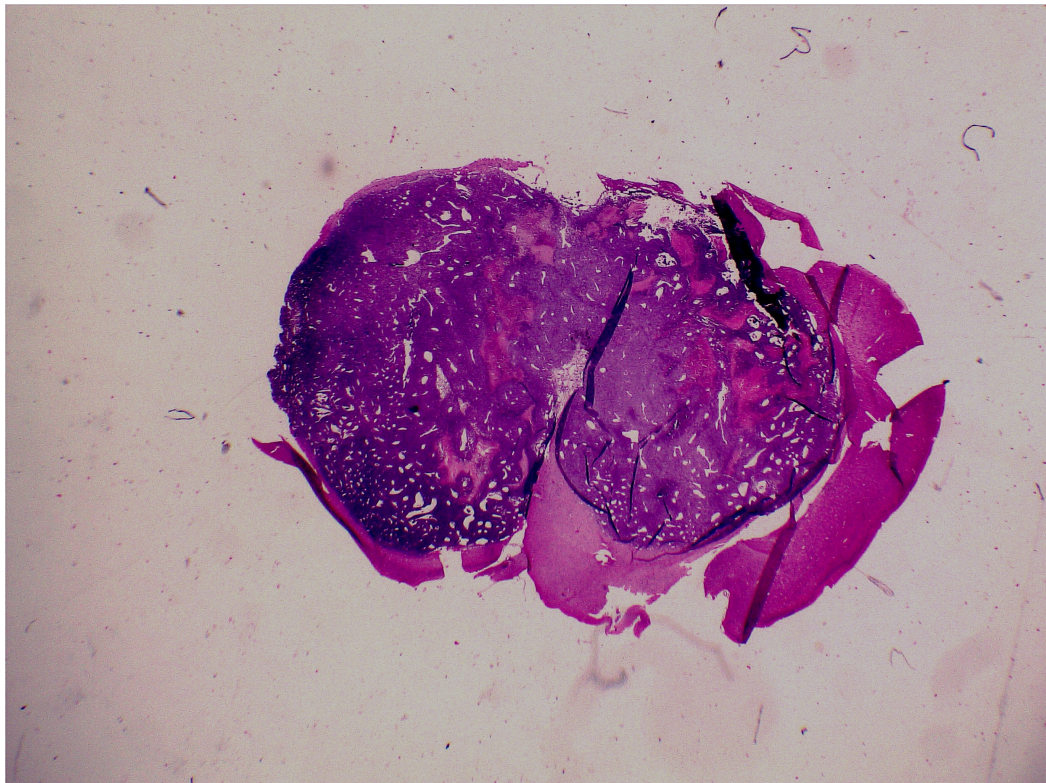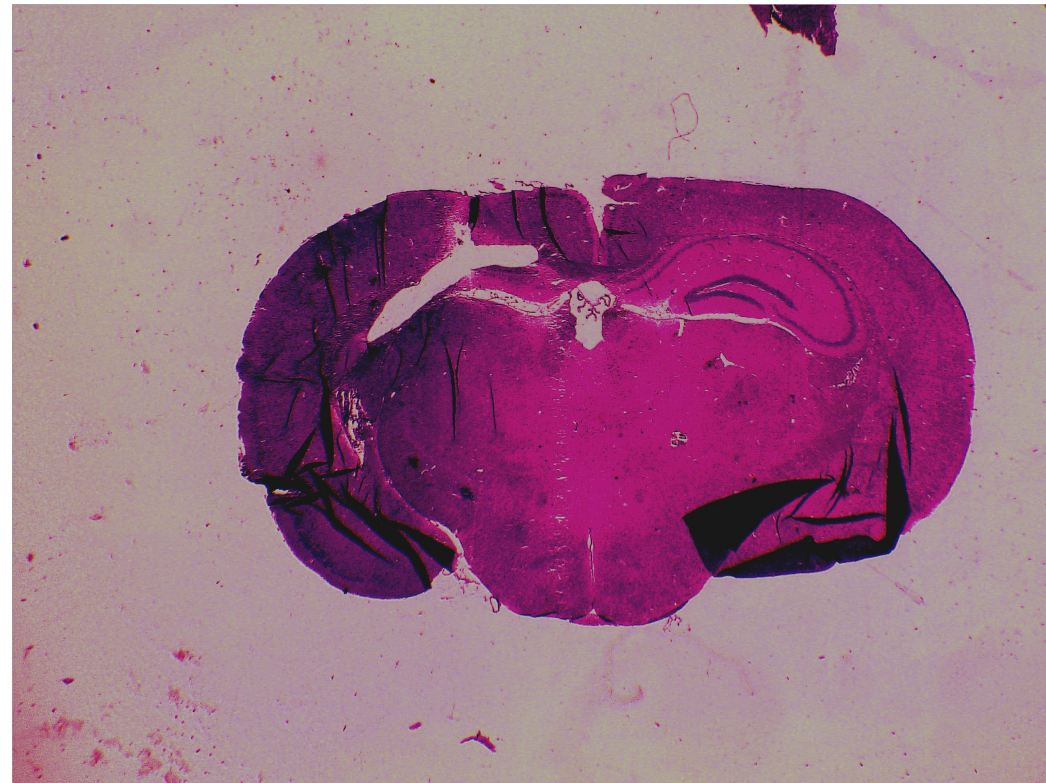

Supplement: Figure 5—source data 4. [file elife-78972-fig5-data4.zip › Figure 5-Source data E/RAW IMAGES FOR PANEL E.pdf]

AGR53-GSC/miRNT (Dox+)

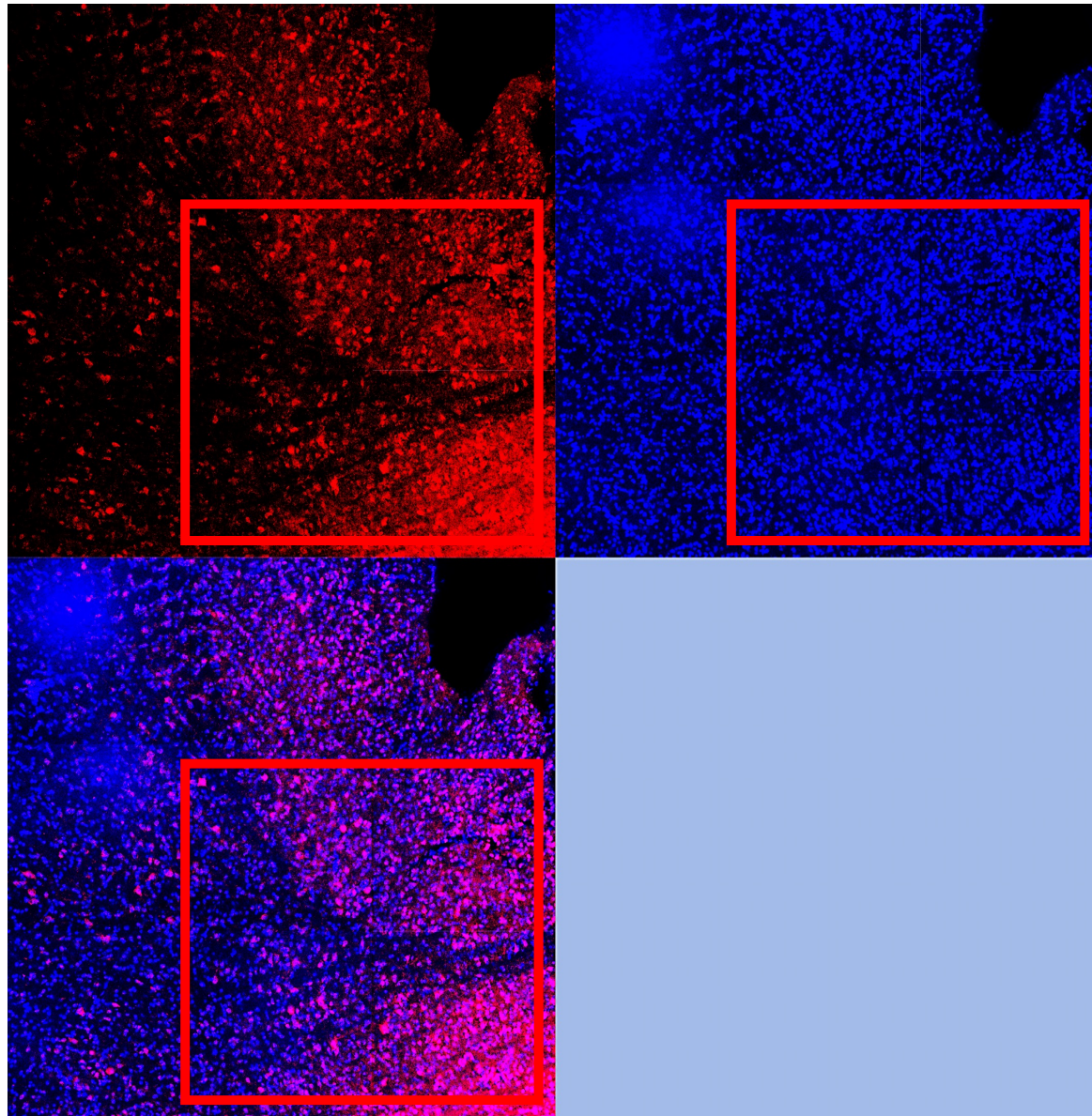

AGR53-GSC/miRFMOD (Dox+)

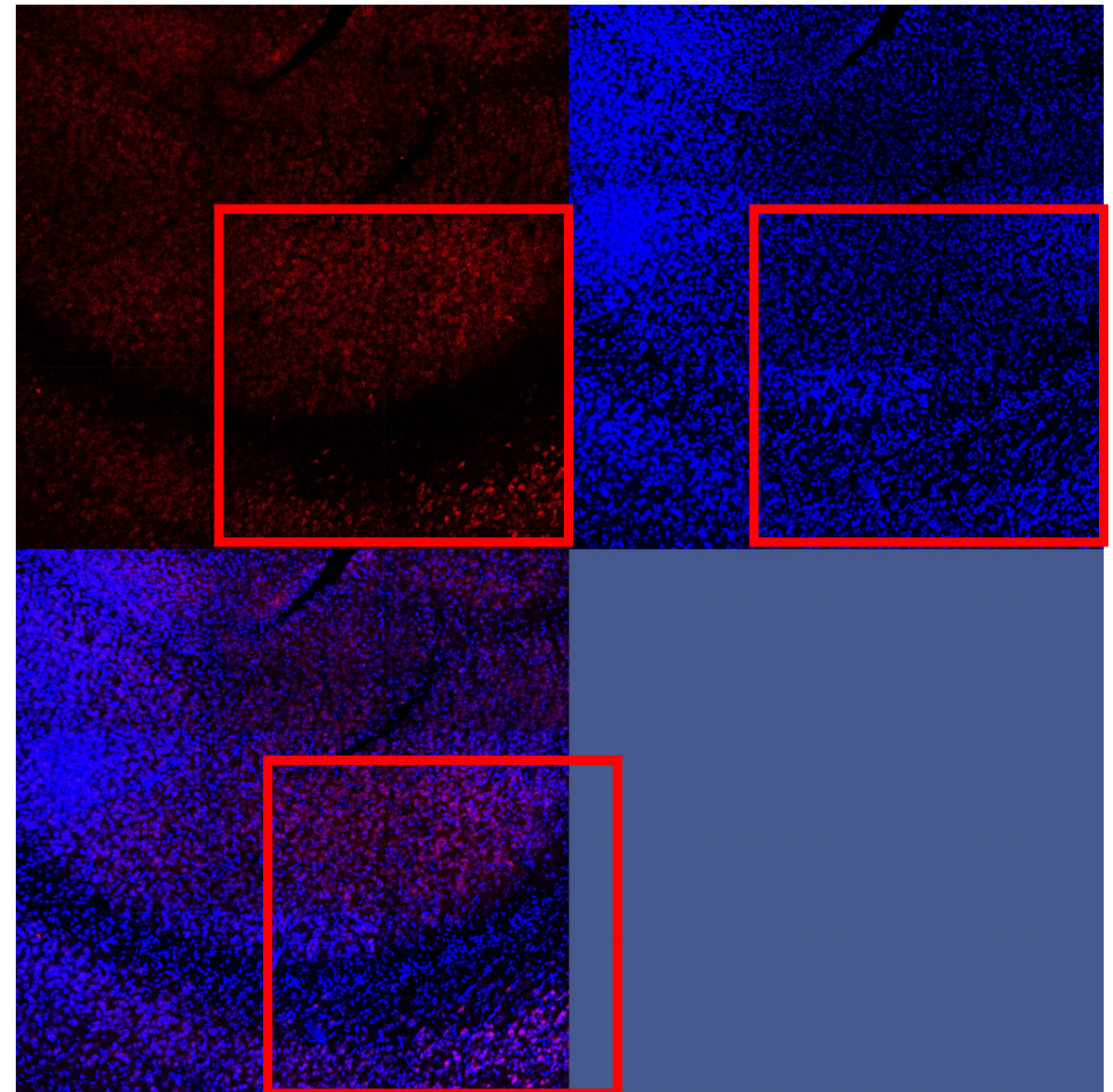

Supplement: Figure 5—source data 5. [file elife-78972-fig5-data5.zip › Figure 5-Source data F/RAW IMAGES FOR PANEL F.pdf]

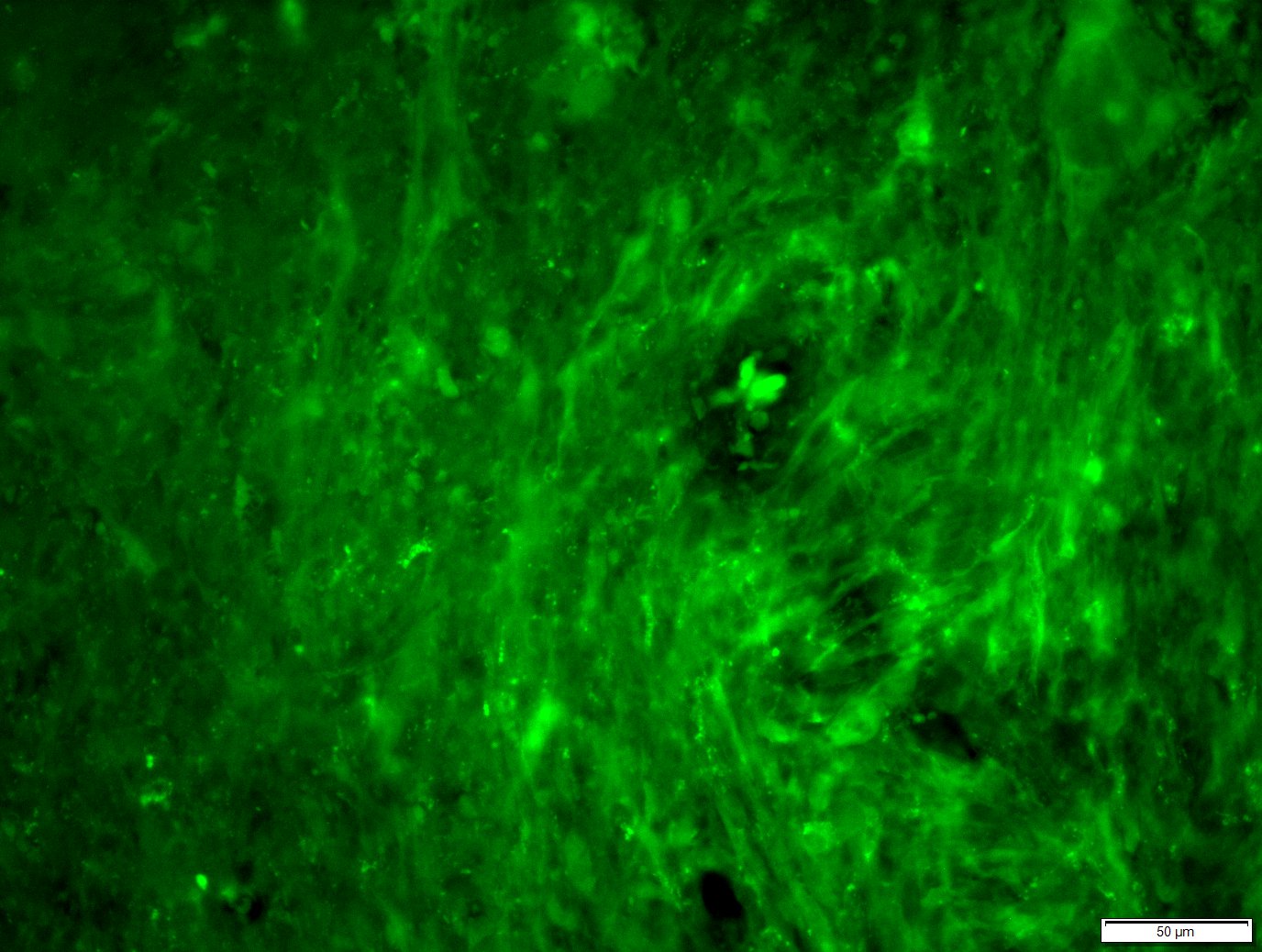

Supplement: Figure 5—source data 6. [file elife-78972-fig5-data6.zip › Figure 5-Source data G/RAW IMAGES FOR LEFT PANEL OF G/Image_578.jpg]

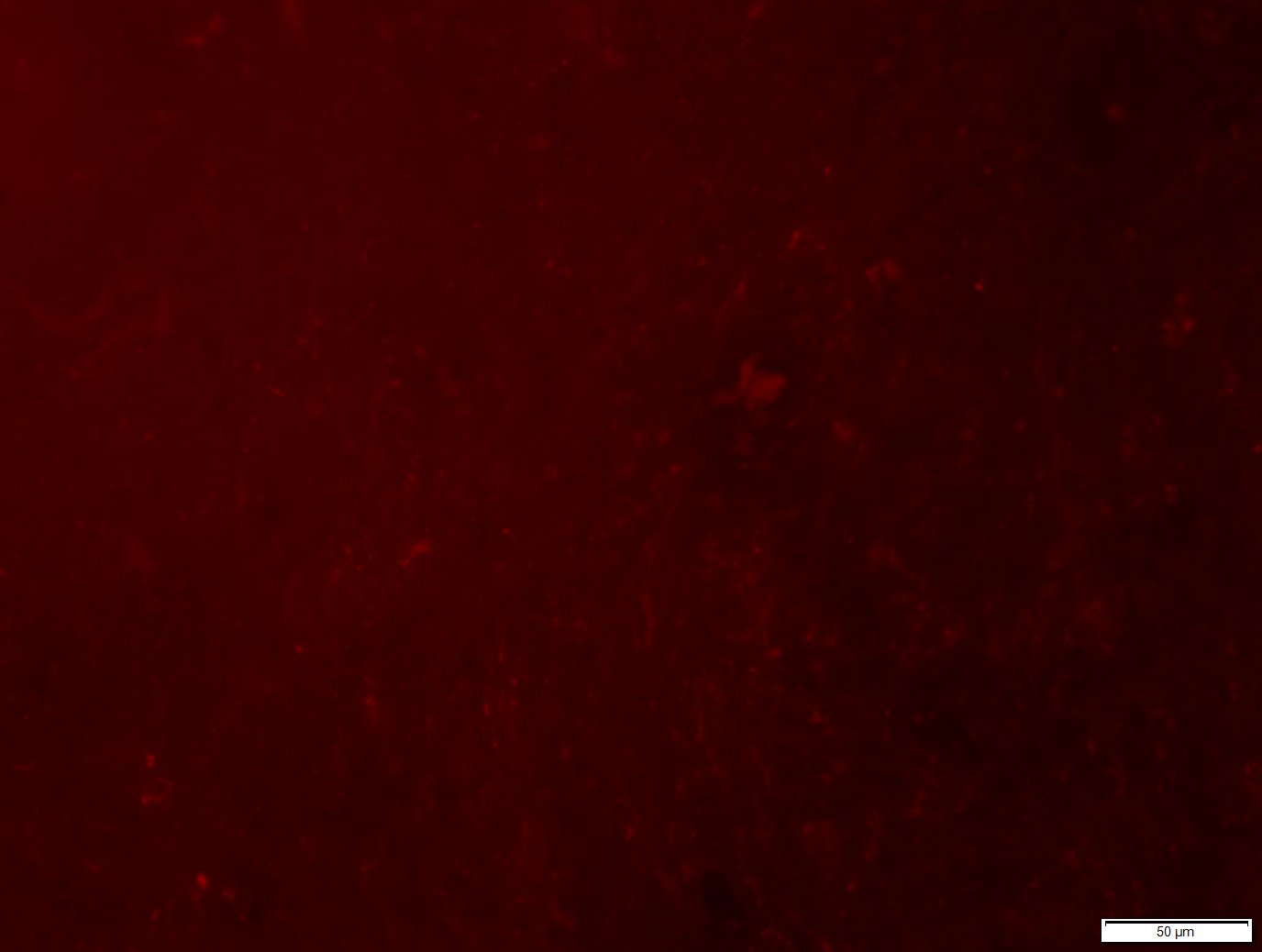

Supplement: Figure 5—source data 6. [file elife-78972-fig5-data6.zip › Figure 5-Source data G/RAW IMAGES FOR LEFT PANEL OF G/Image_579.jpg]

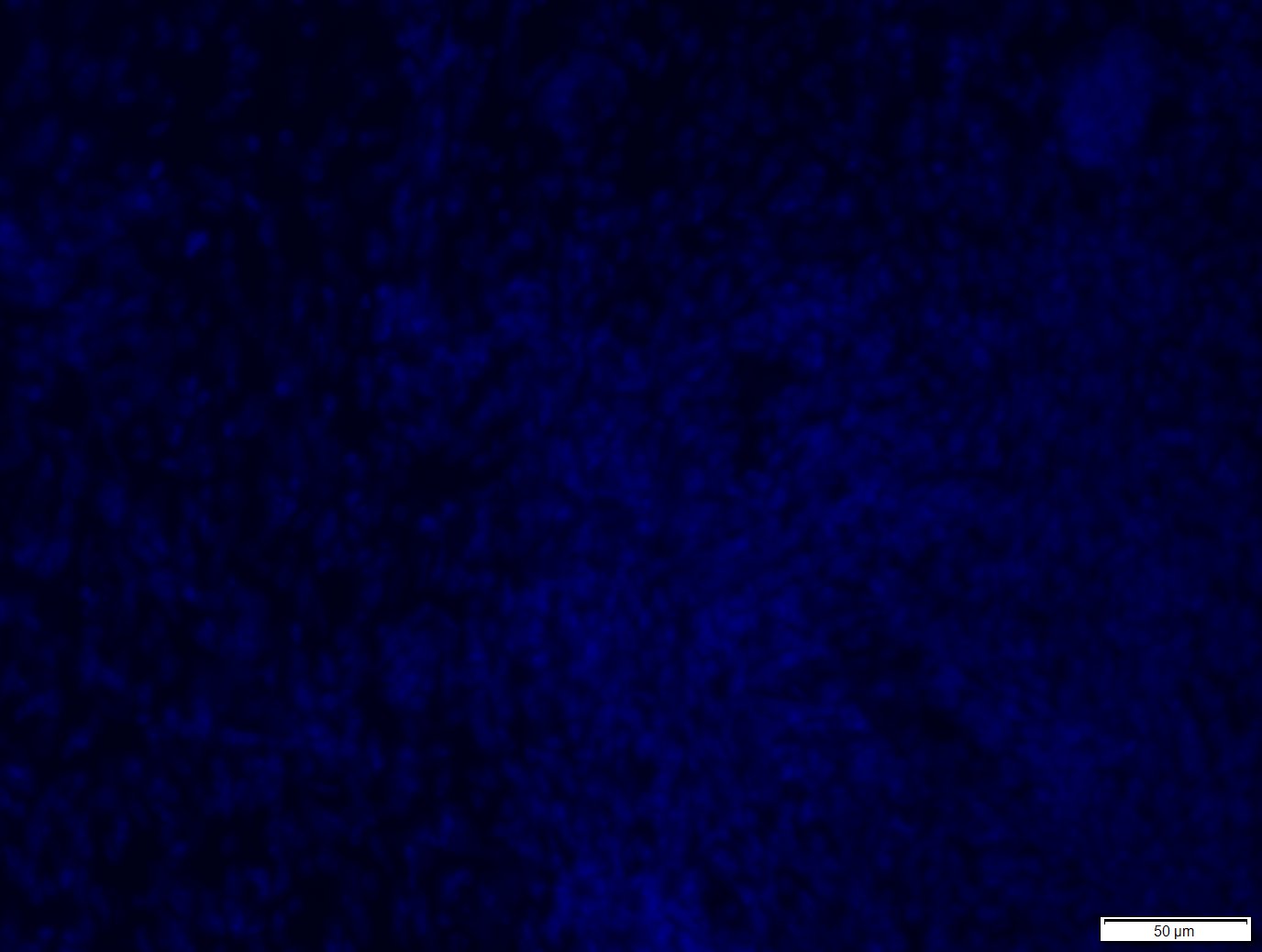

Supplement: Figure 5—source data 6. [file elife-78972-fig5-data6.zip › Figure 5-Source data G/RAW IMAGES FOR LEFT PANEL OF G/Image_580.jpg]

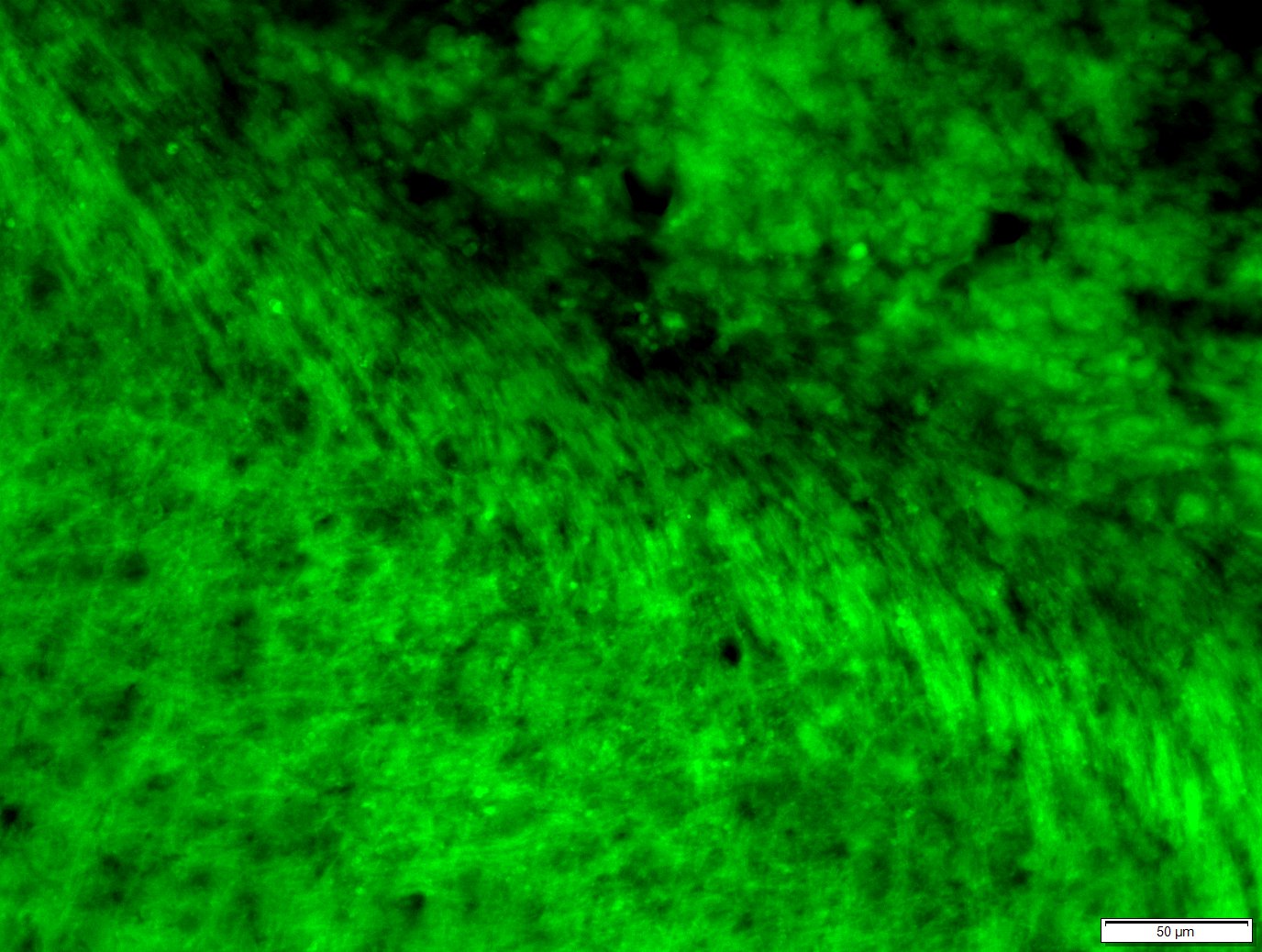

Supplement: Figure 5—source data 6. [file elife-78972-fig5-data6.zip › Figure 5-Source data G/RAW IMAGES FOR LEFT PANEL OF G/Image_584.jpg]

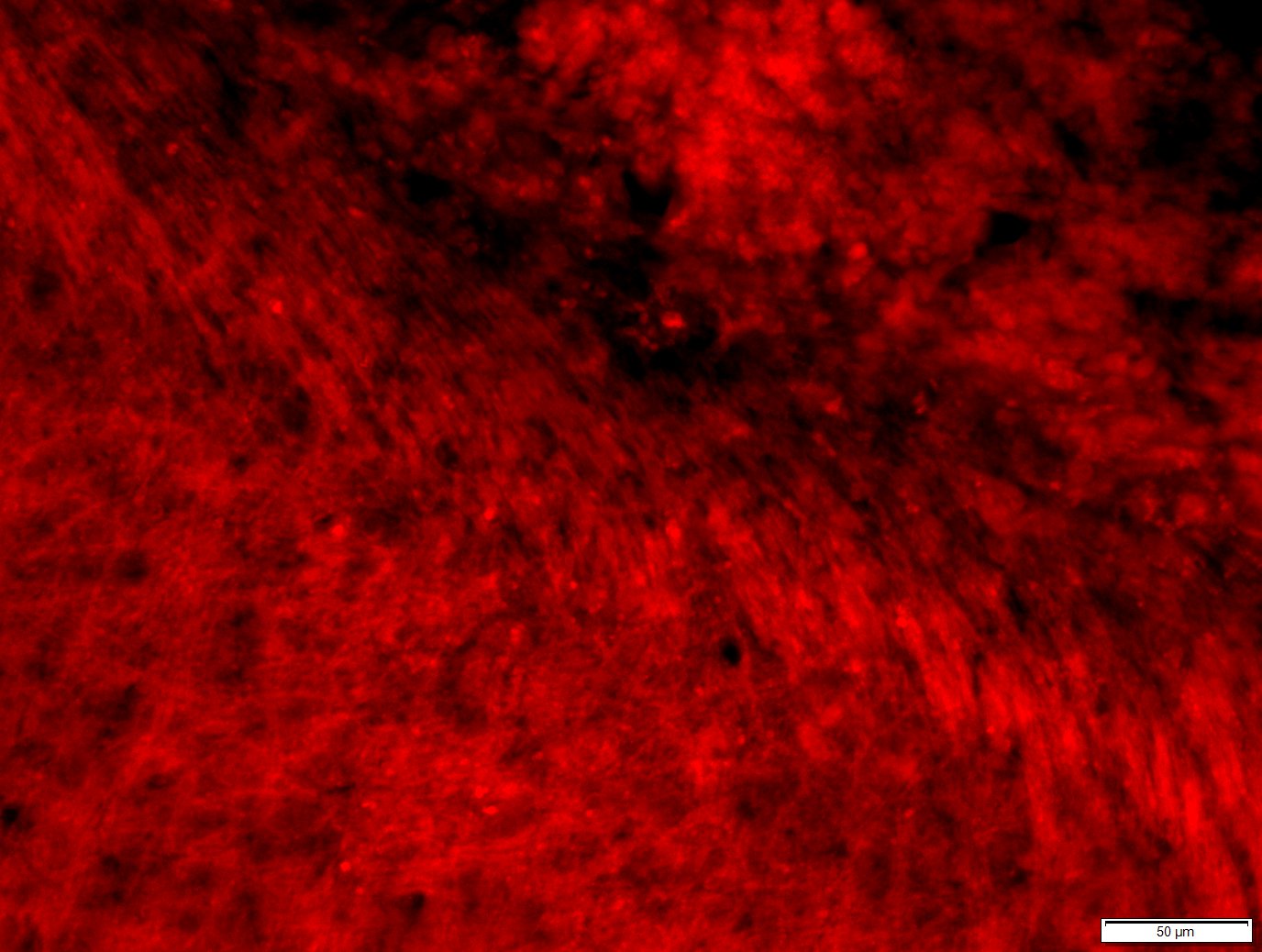

Supplement: Figure 5—source data 6. [file elife-78972-fig5-data6.zip › Figure 5-Source data G/RAW IMAGES FOR LEFT PANEL OF G/Image_585.jpg]

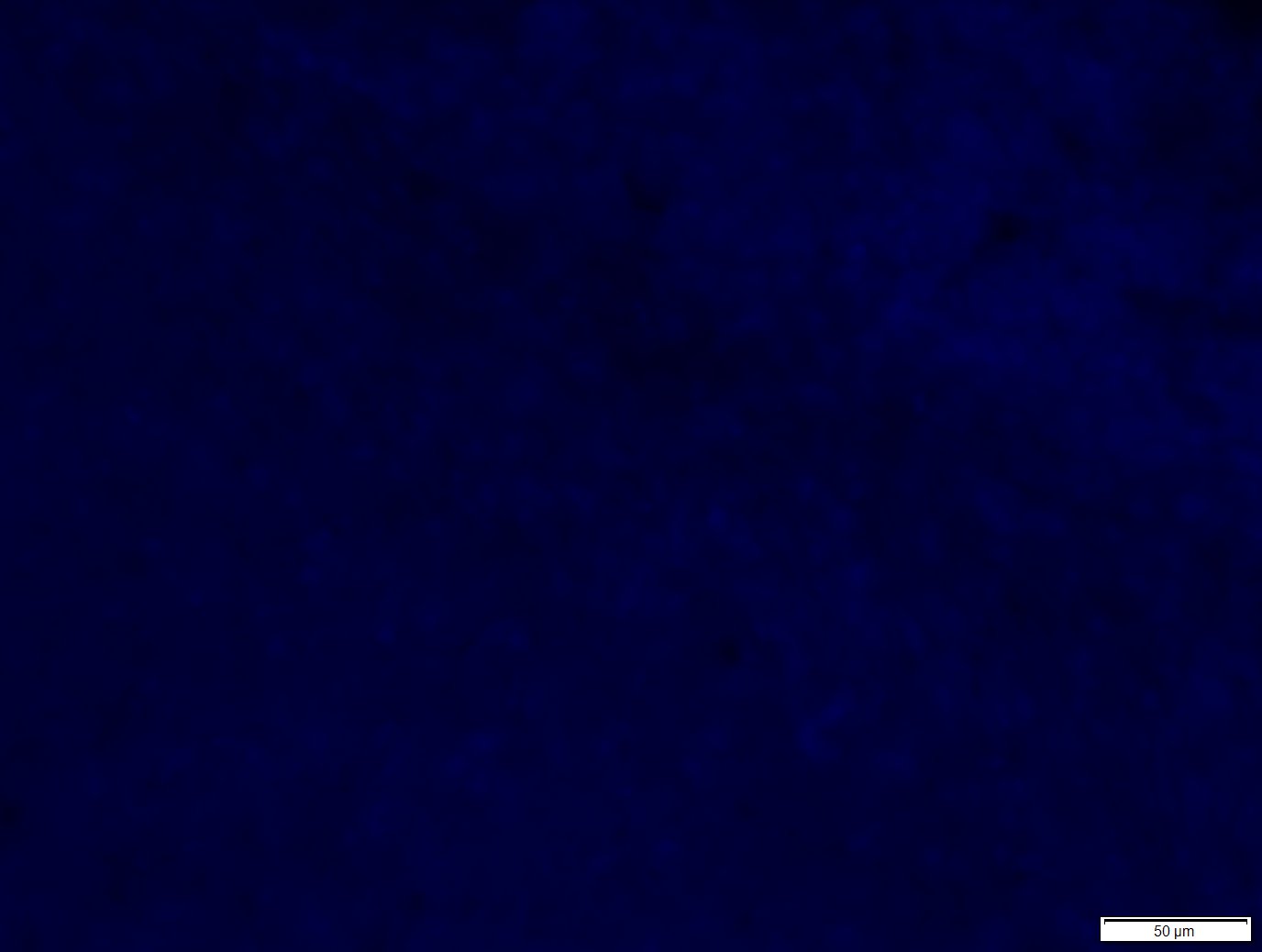

Supplement: Figure 5—source data 6. [file elife-78972-fig5-data6.zip › Figure 5-Source data G/RAW IMAGES FOR LEFT PANEL OF G/Image_586.jpg]

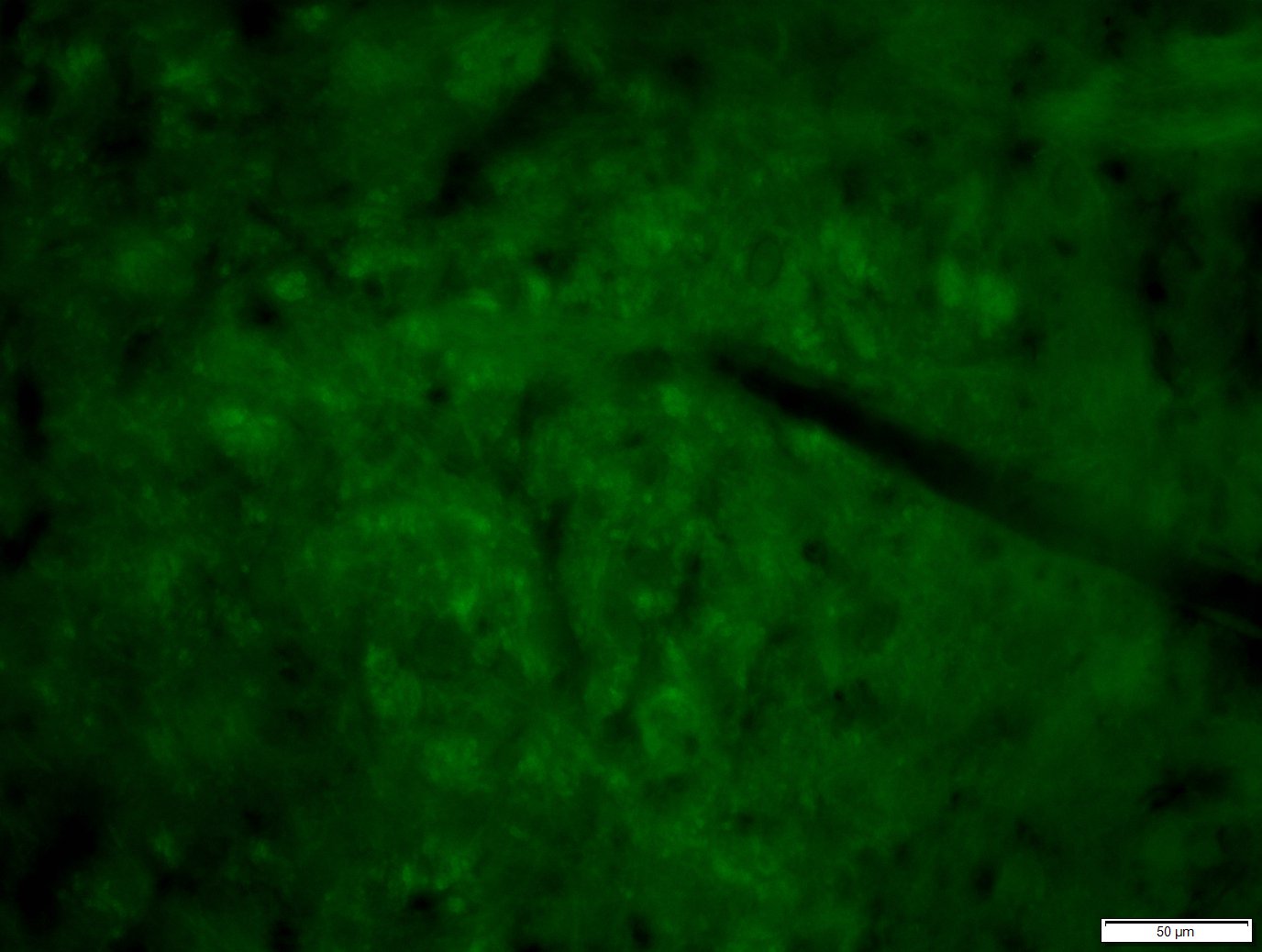

Supplement: Figure 5—source data 6. [file elife-78972-fig5-data6.zip › Figure 5-Source data G/RAW IMAGES FOR LEFT PANEL OF G/Image_622.jpg]

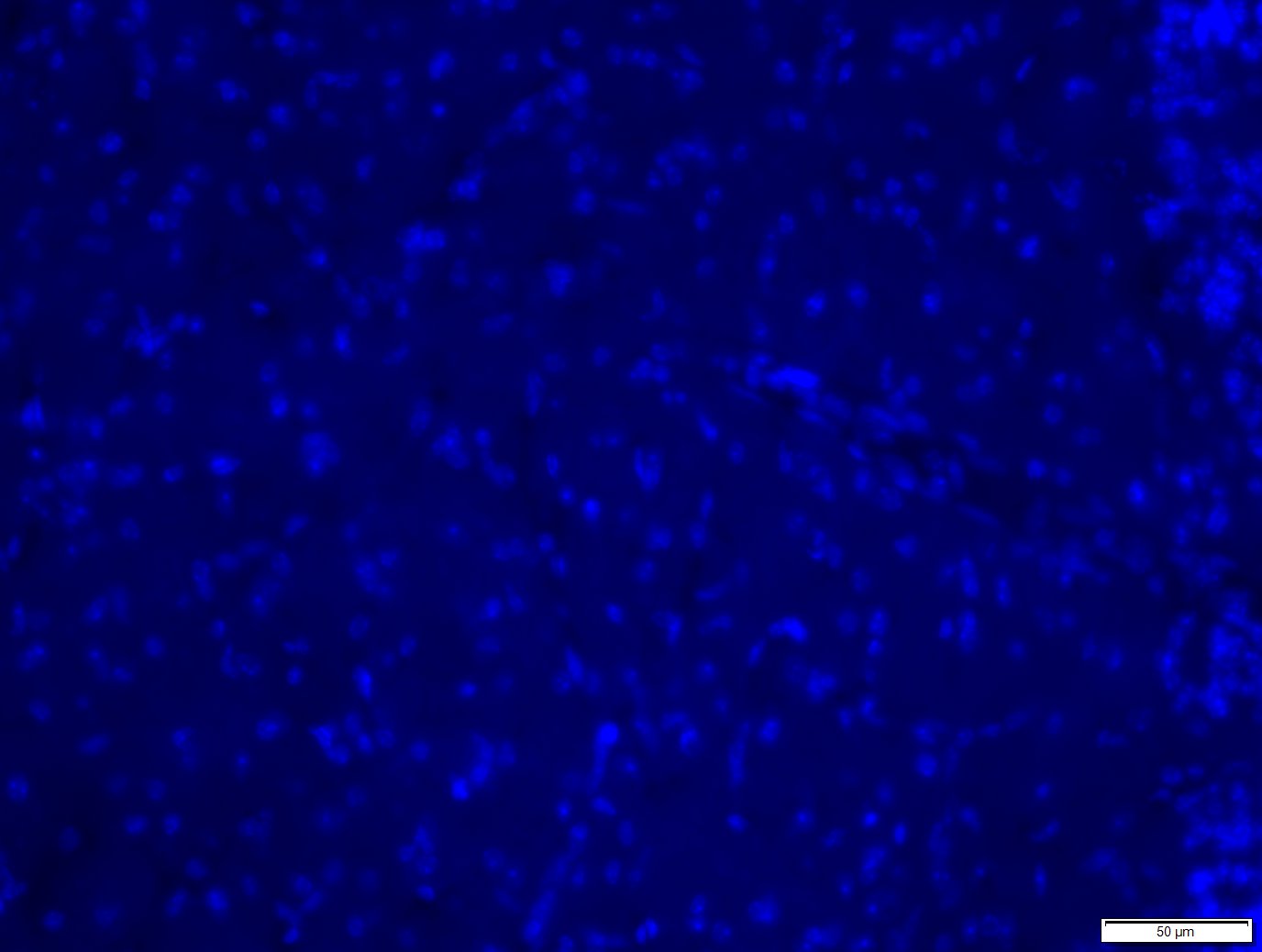

Supplement: Figure 5—source data 6. [file elife-78972-fig5-data6.zip › Figure 5-Source data G/RAW IMAGES FOR LEFT PANEL OF G/Image_624.jpg]

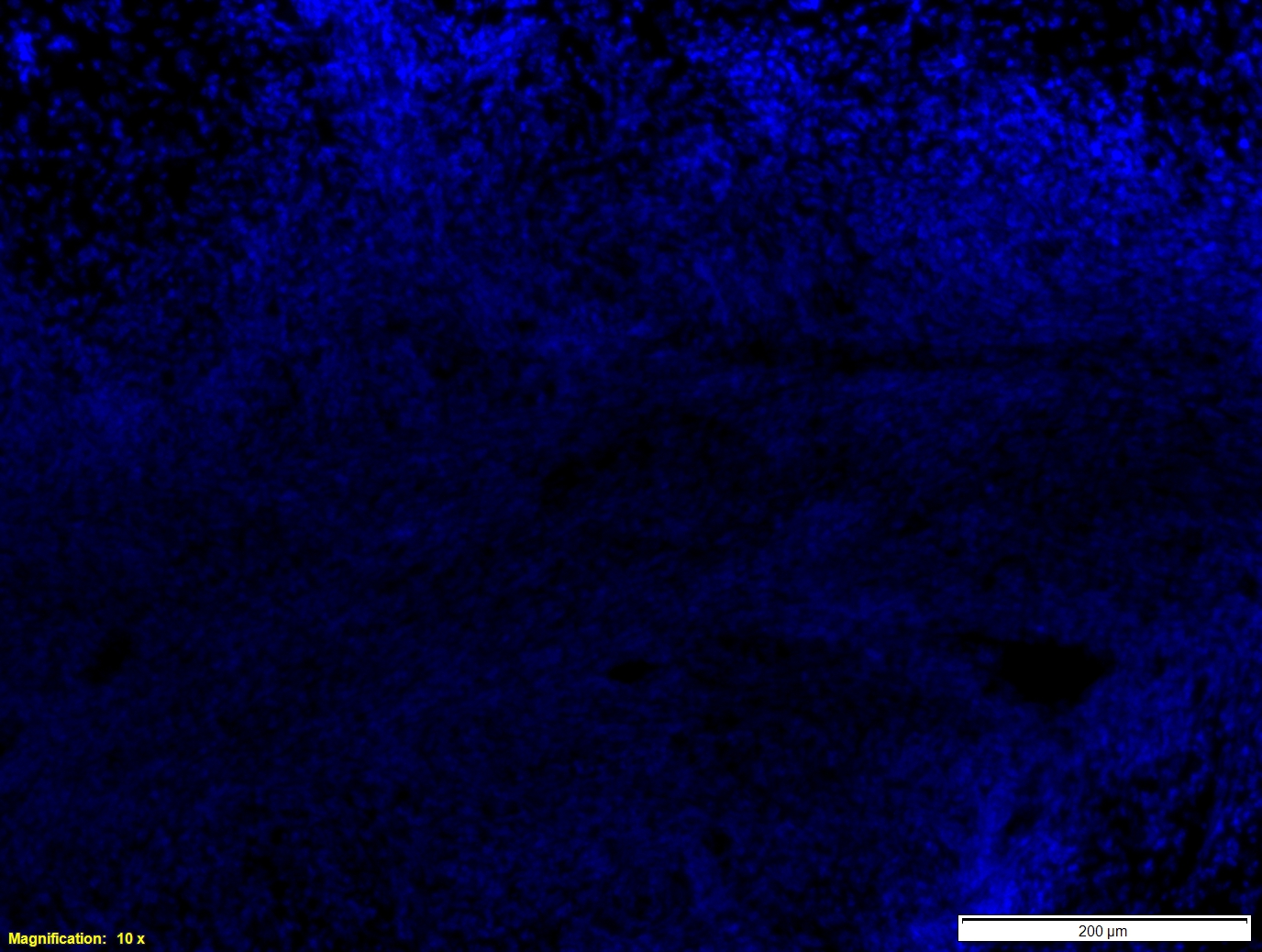

Supplement: Figure 5—source data 6. [file elife-78972-fig5-data6.zip › Figure 5-Source data G/RAW IMAGES FOR RIGHT PANEL OF G/dapi.jpg]

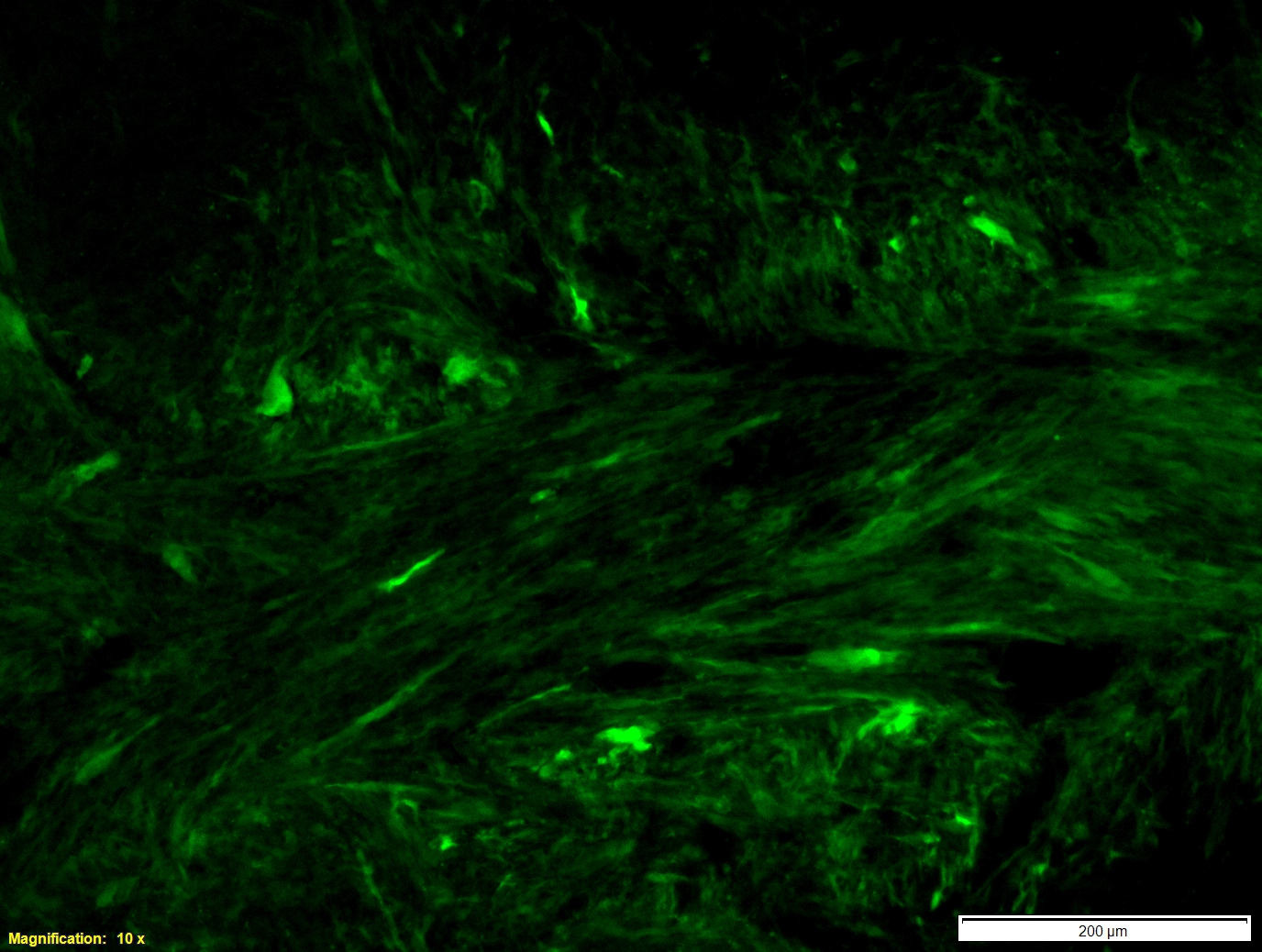

Supplement: Figure 5—source data 6. [file elife-78972-fig5-data6.zip › Figure 5-Source data G/RAW IMAGES FOR RIGHT PANEL OF G/gfp 10x.jpg]

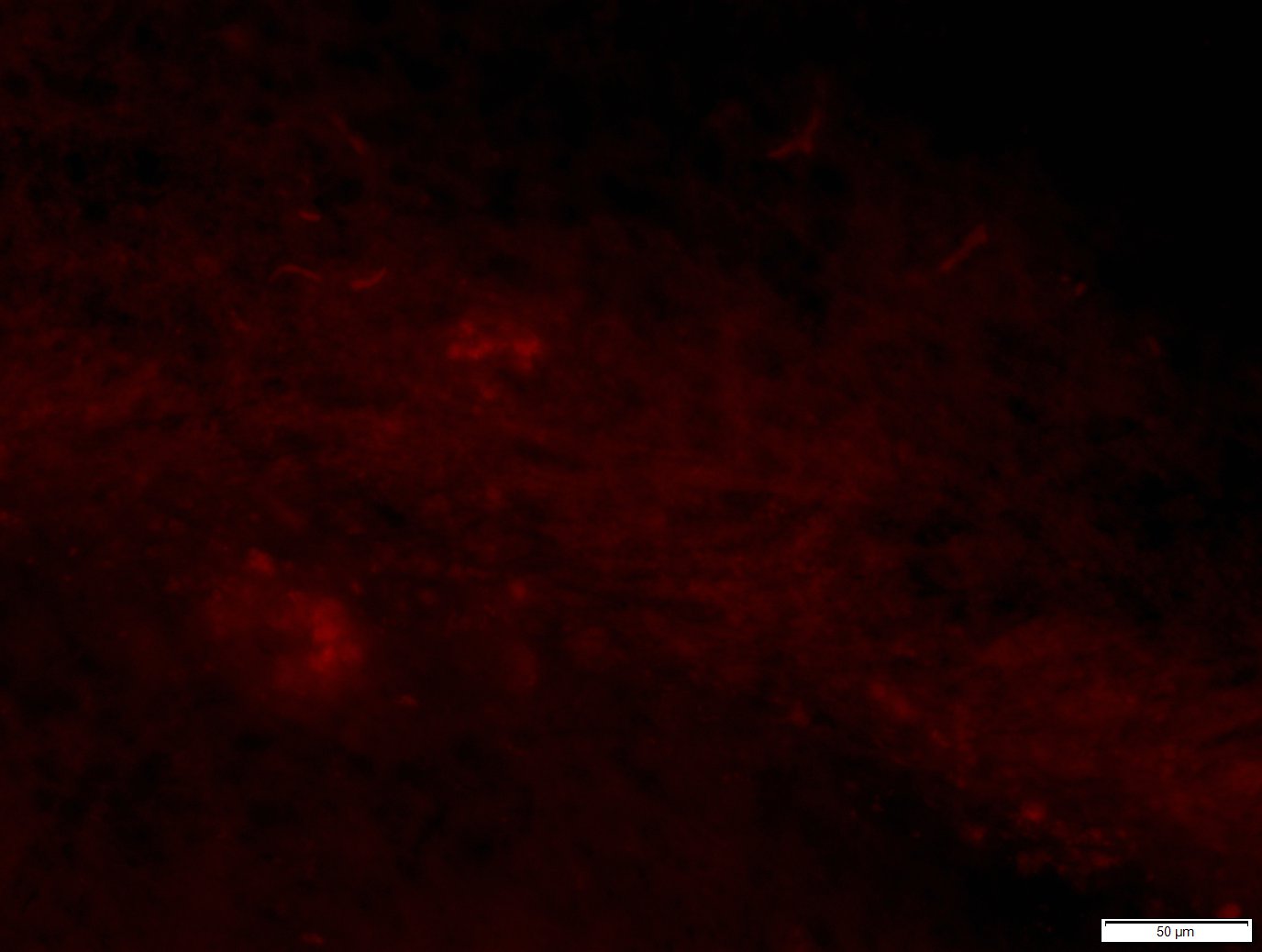

Supplement: Figure 5—source data 6. [file elife-78972-fig5-data6.zip › Figure 5-Source data G/RAW IMAGES FOR RIGHT PANEL OF G/Image_599.jpg]

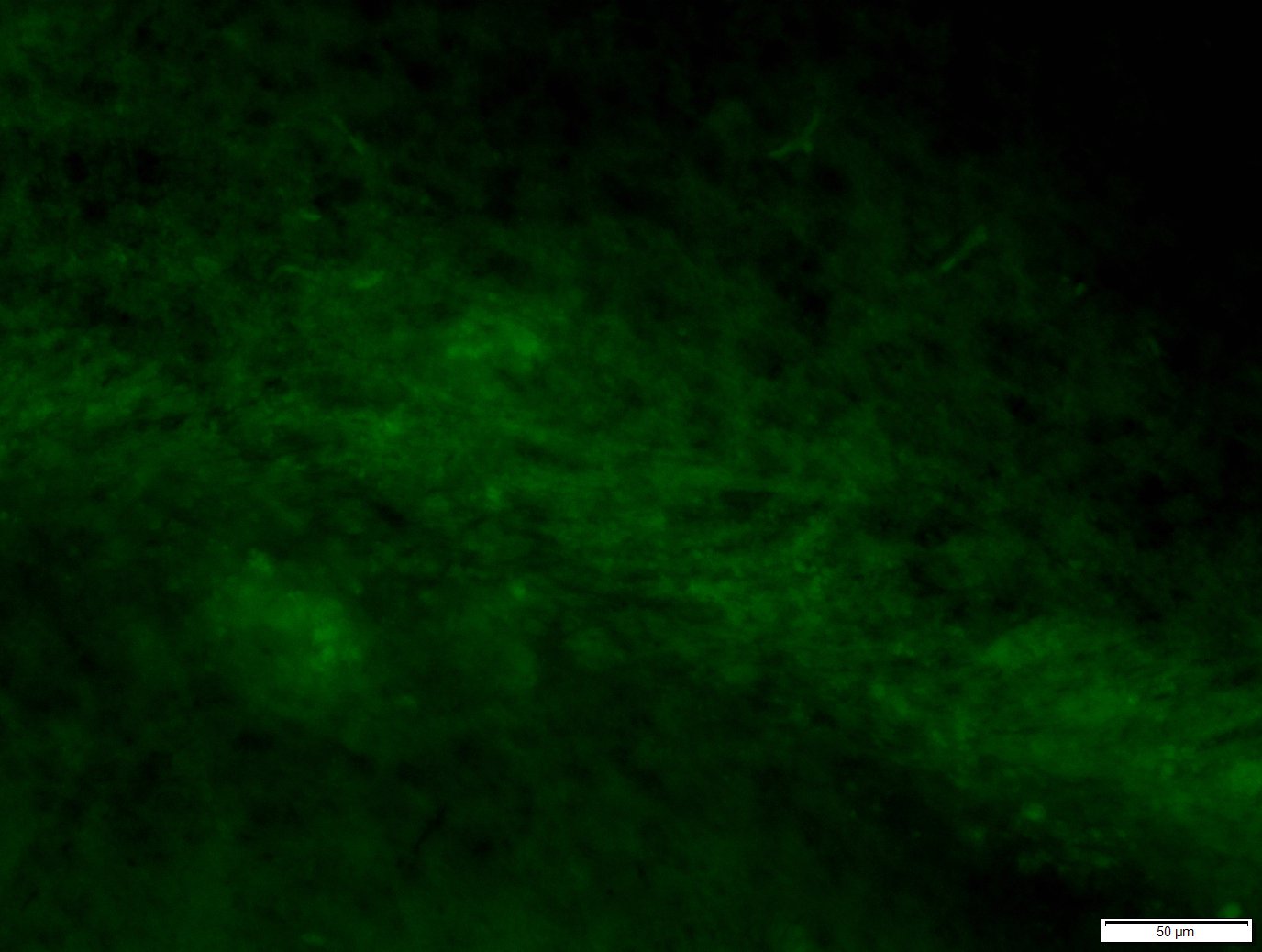

Supplement: Figure 5—source data 6. [file elife-78972-fig5-data6.zip › Figure 5-Source data G/RAW IMAGES FOR RIGHT PANEL OF G/Image_600.jpg]

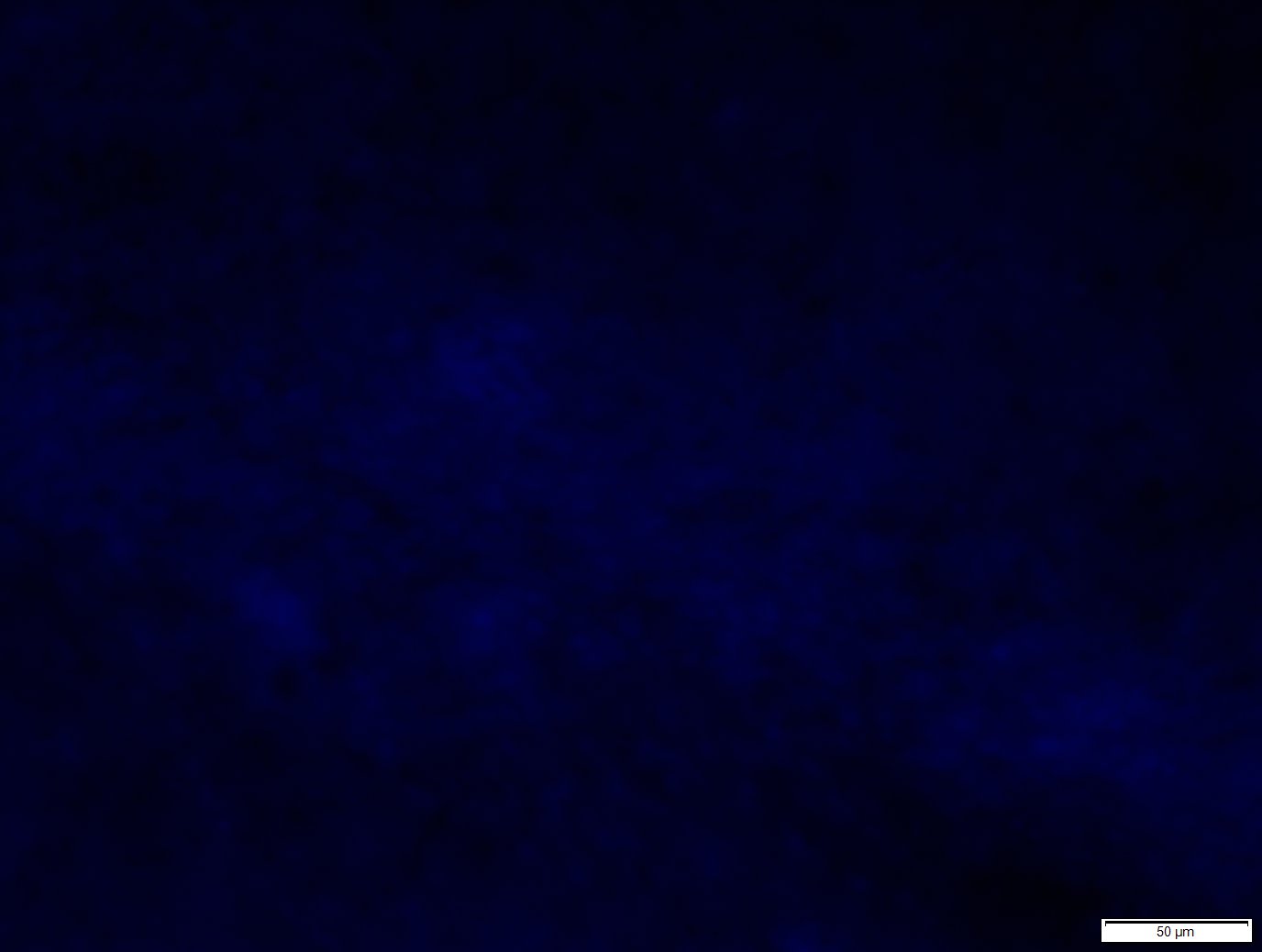

Supplement: Figure 5—source data 6. [file elife-78972-fig5-data6.zip › Figure 5-Source data G/RAW IMAGES FOR RIGHT PANEL OF G/Image_601.jpg]

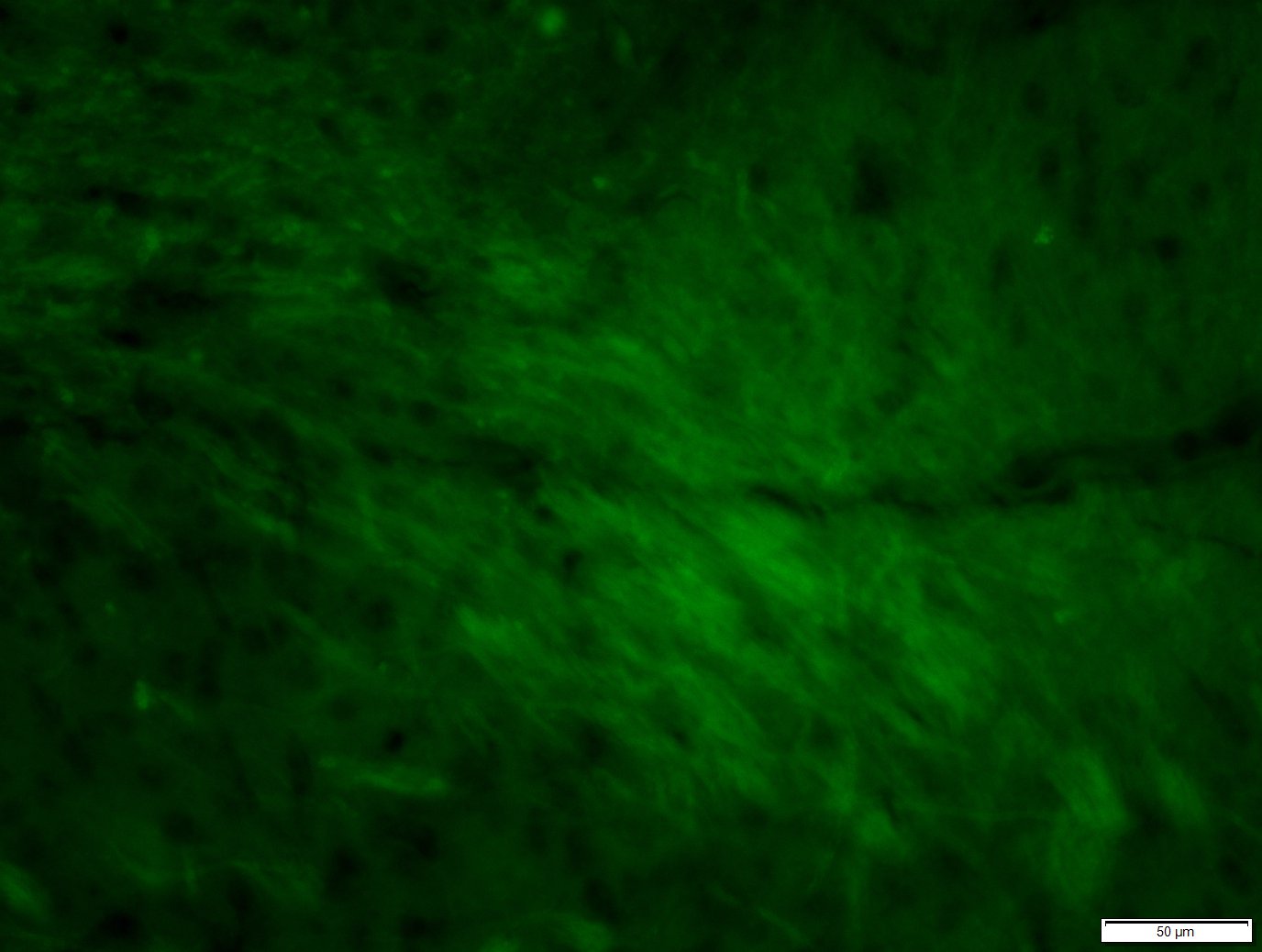

Supplement: Figure 5—source data 6. [file elife-78972-fig5-data6.zip › Figure 5-Source data G/RAW IMAGES FOR RIGHT PANEL OF G/Image_619.jpg]

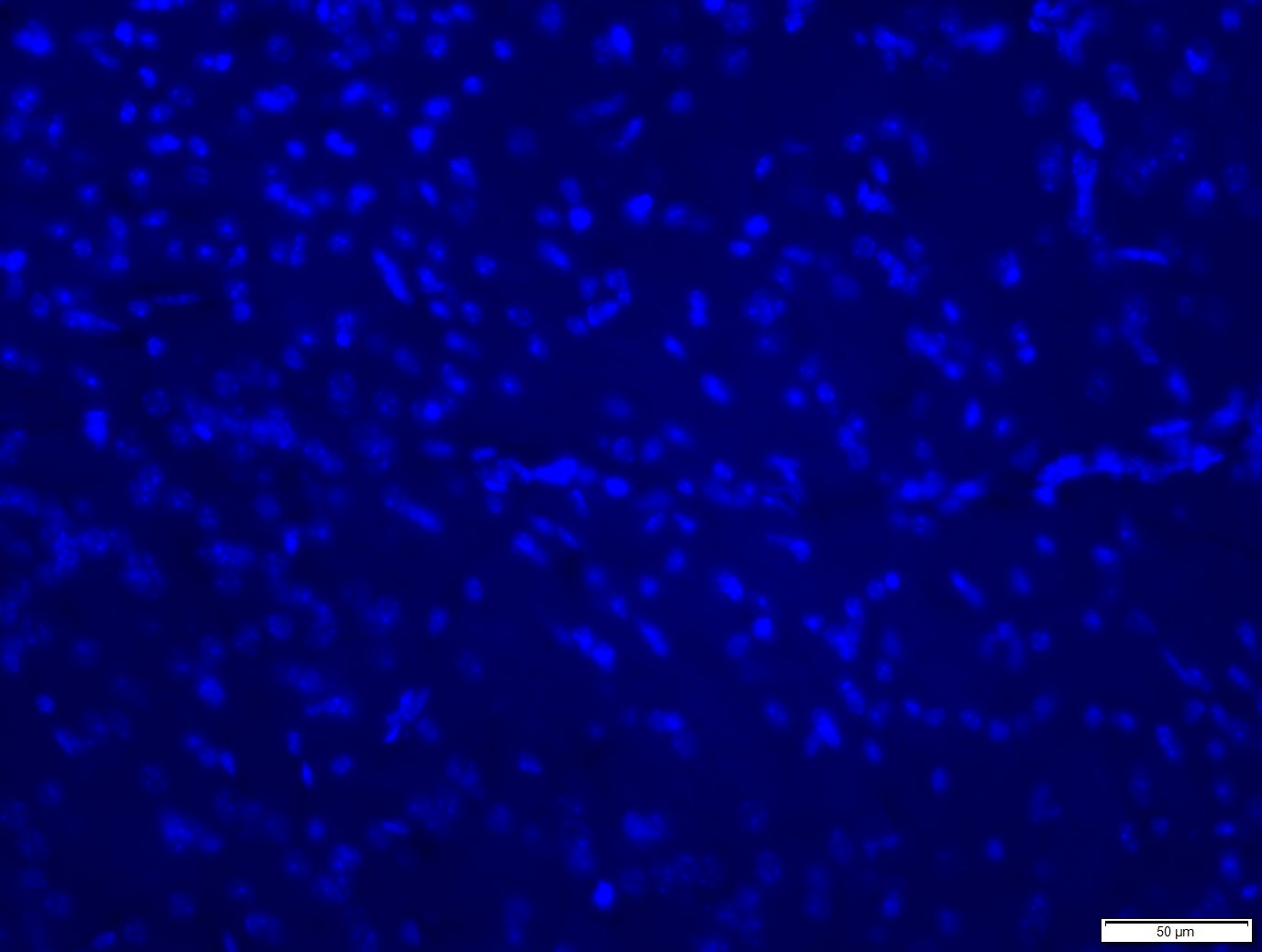

Supplement: Figure 5—source data 6. [file elife-78972-fig5-data6.zip › Figure 5-Source data G/RAW IMAGES FOR RIGHT PANEL OF G/Image_621.jpg]

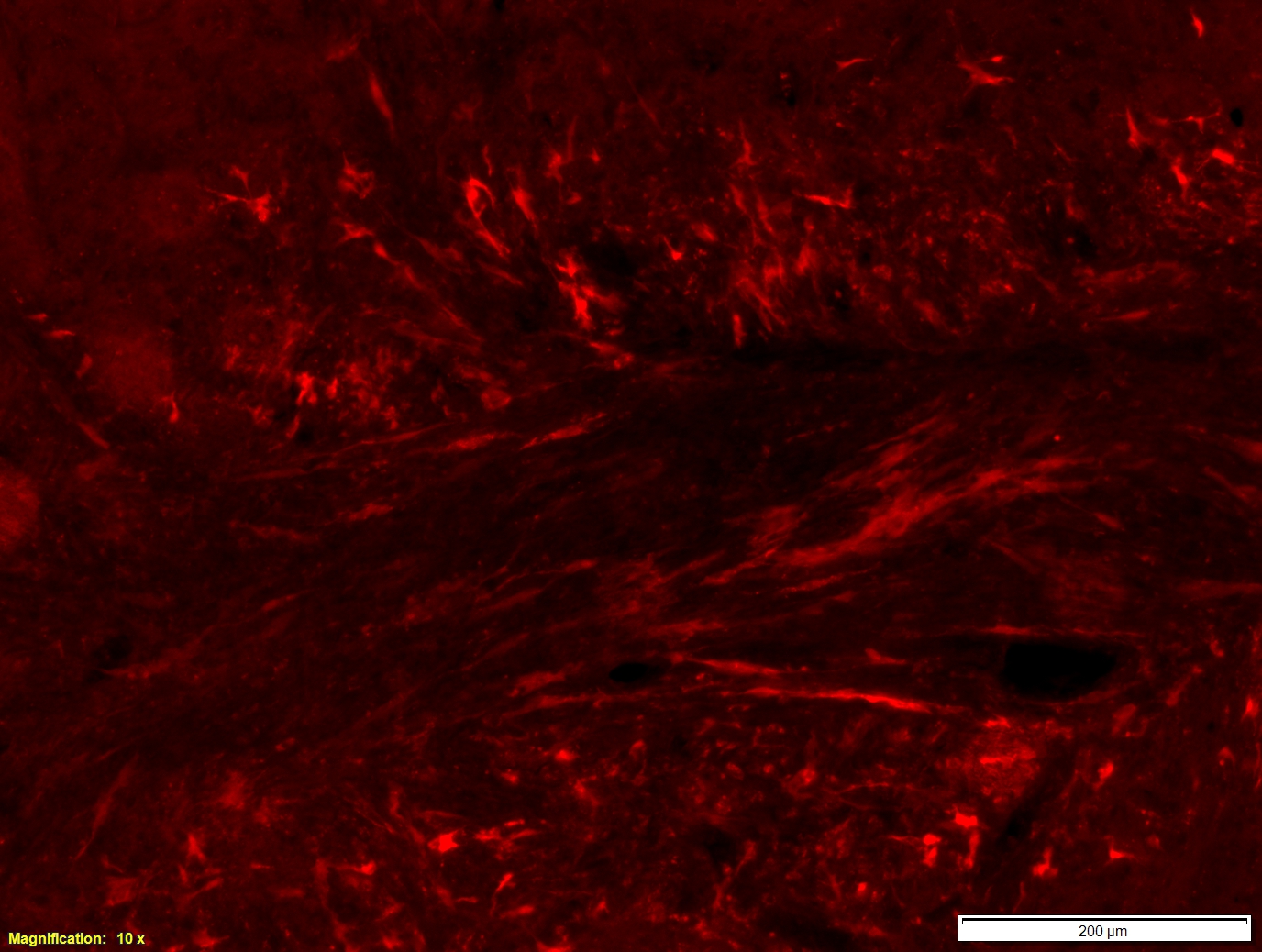

Supplement: Figure 5—source data 6. [file elife-78972-fig5-data6.zip › Figure 5-Source data G/RAW IMAGES FOR RIGHT PANEL OF G/mCherry 10x.jpg]

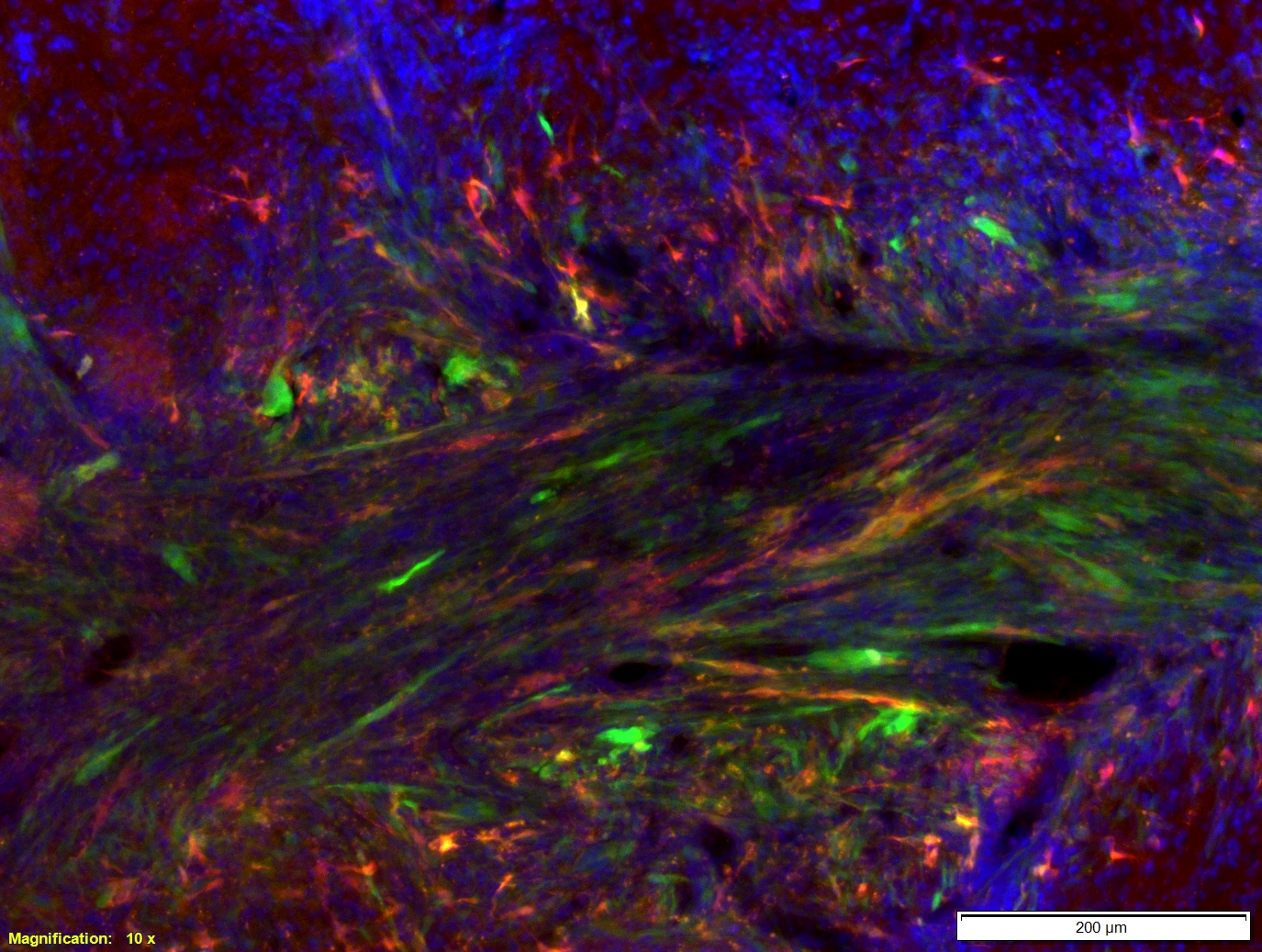

Supplement: Figure 5—source data 6. [file elife-78972-fig5-data6.zip › Figure 5-Source data G/RAW IMAGES FOR RIGHT PANEL OF G/merge 10x.jpg]

DAY1

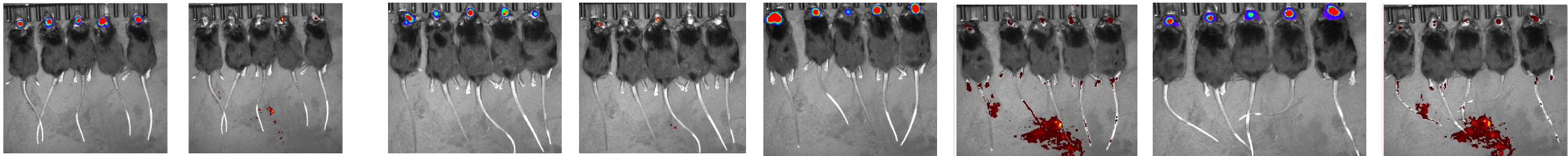

DAY2

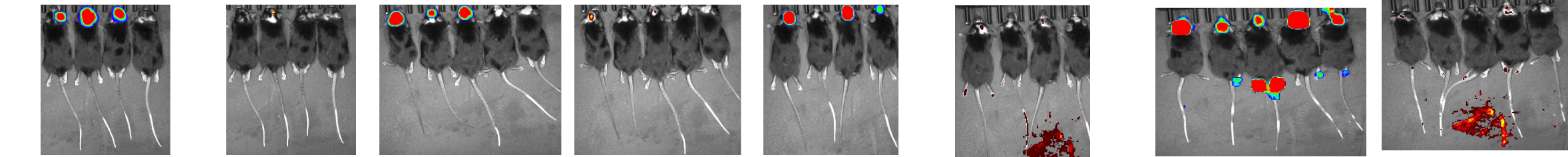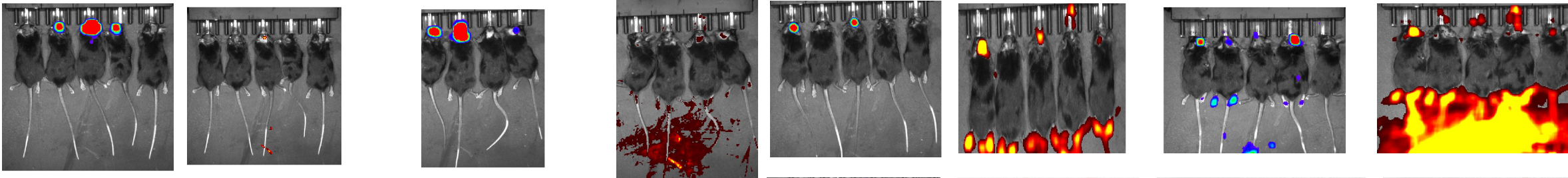

DAY4

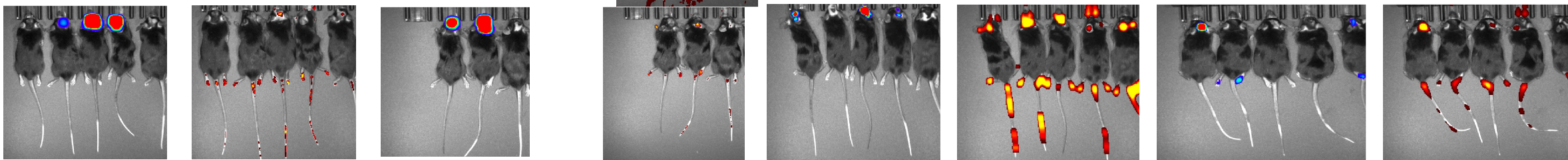

DAY5

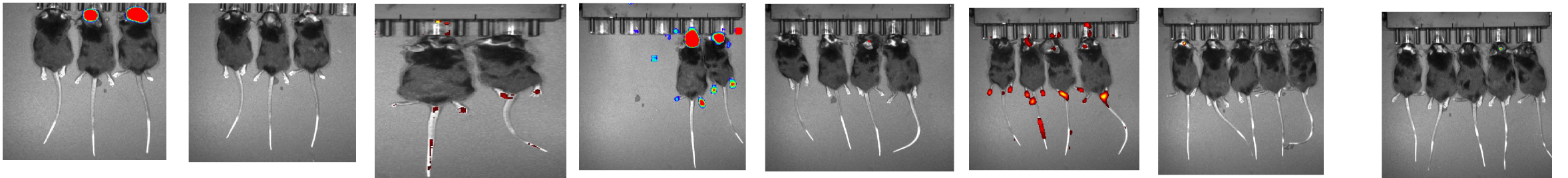

Supplement: Figure 5—figure supplement 1—source data 1. [file elife-78972-fig5-figsupp1-data1.zip › Figure 5-Figure Supplement 1-Source Data/Figure 5-Figure Supplement 1-Source Data A/in vivo imaging images.pdf]

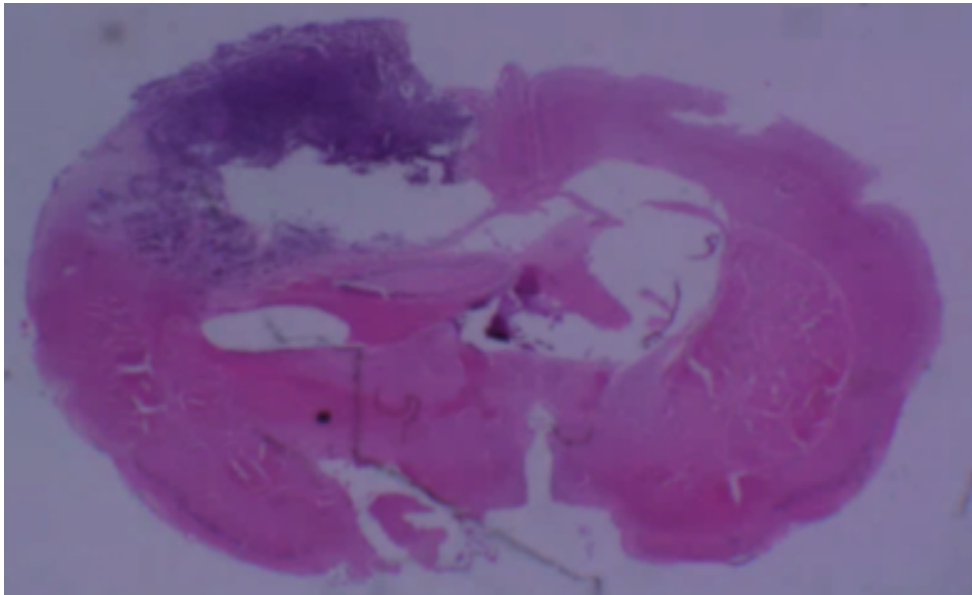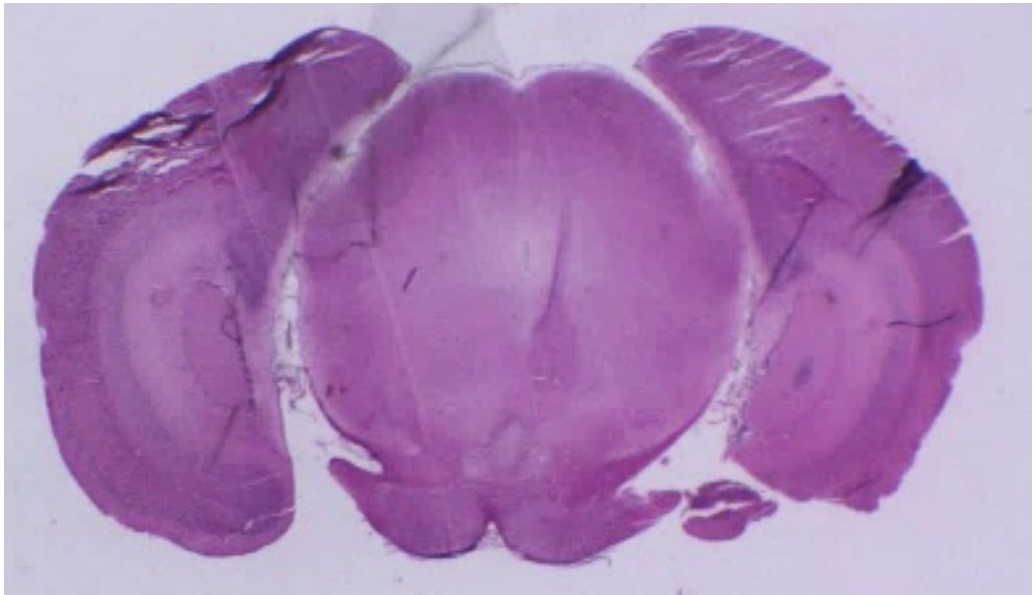

Supplement: Figure 5—figure supplement 1—source data 1. [file elife-78972-fig5-figsupp1-data1.zip › Figure 5-Figure Supplement 1-Source Data/Figure 5-Figure Supplement 1-Source Data B/HnE brain images.pdf]

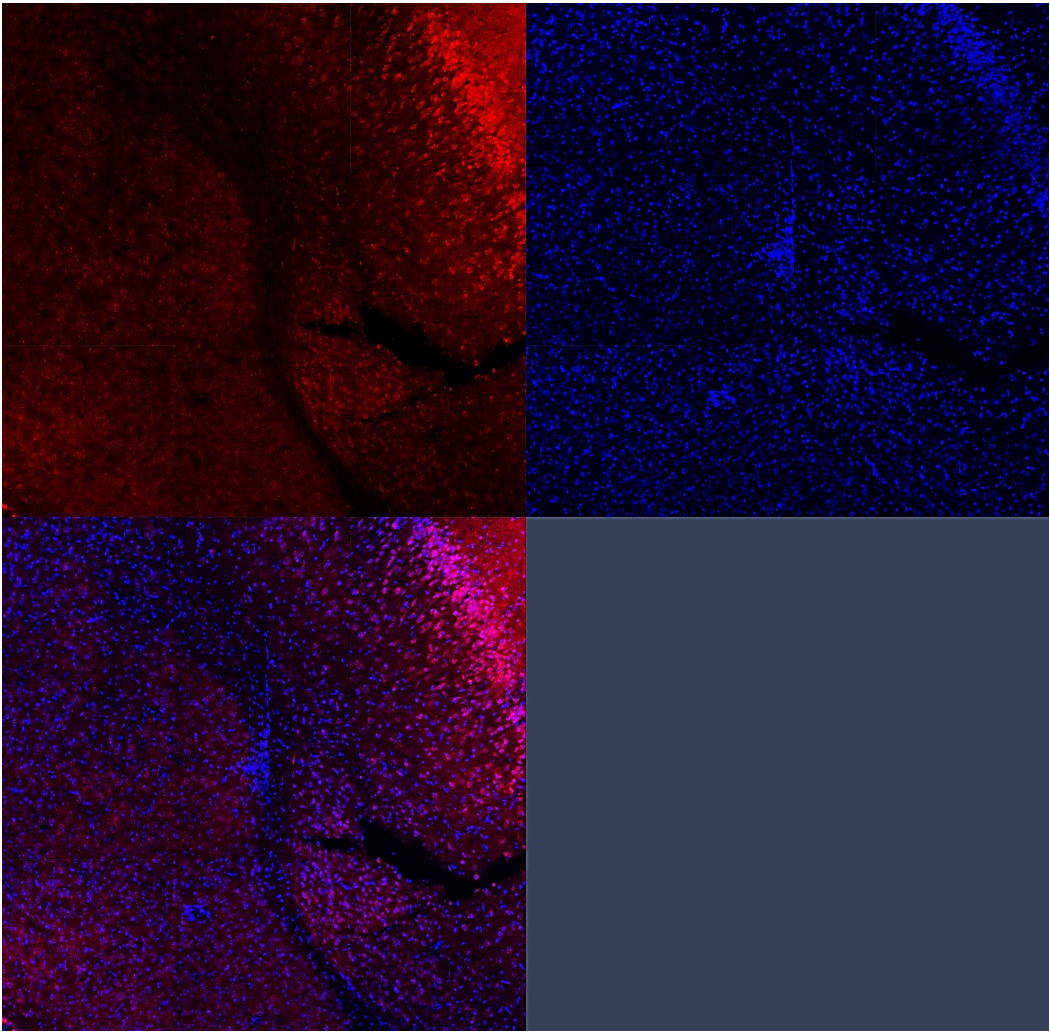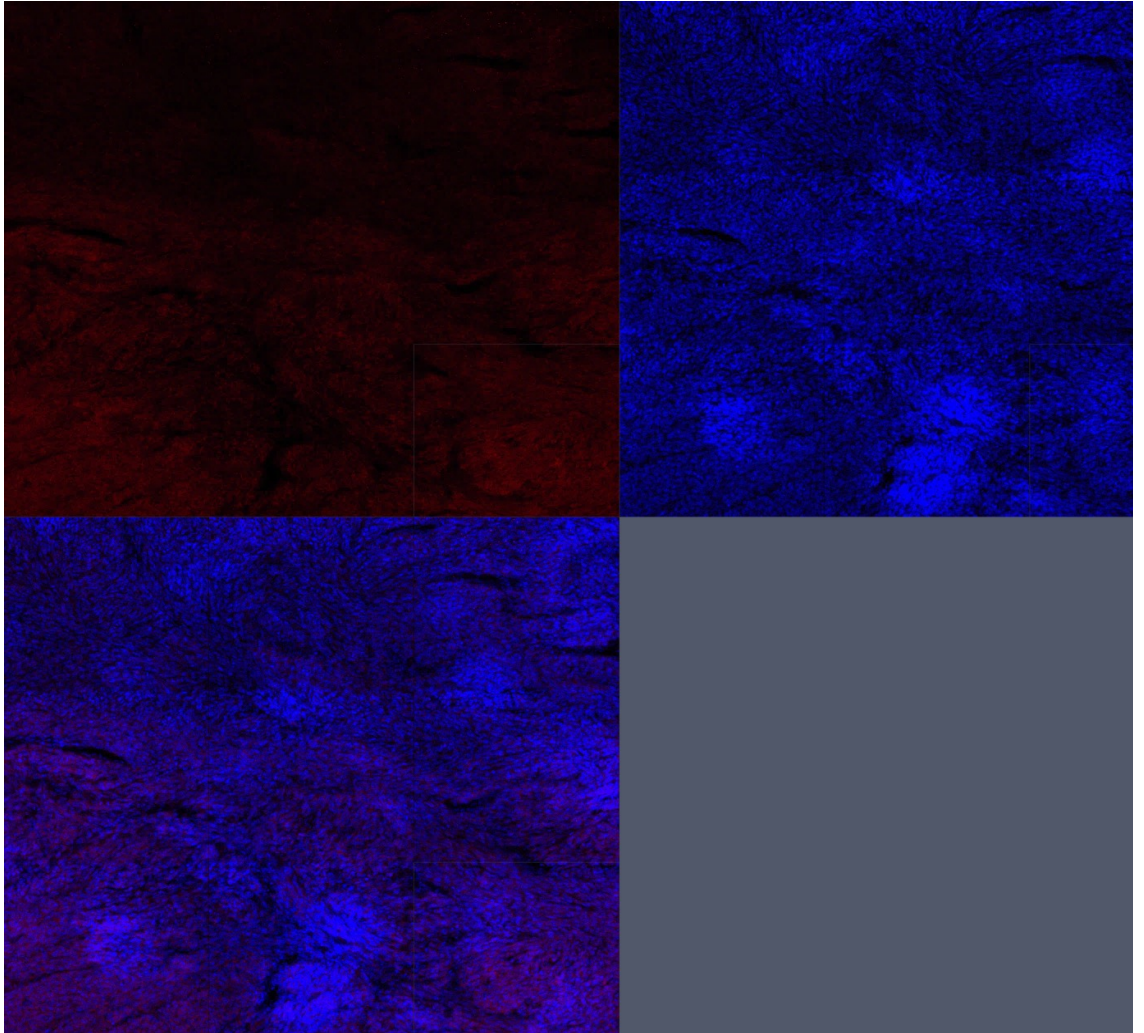

Supplement: Figure 5—figure supplement 1—source data 1. [file elife-78972-fig5-figsupp1-data1.zip › Figure 5-Figure Supplement 1-Source Data/Figure 5-Figure Supplement 1-Source Data D/confocal images for panel d.pdf]

## IgG GSC

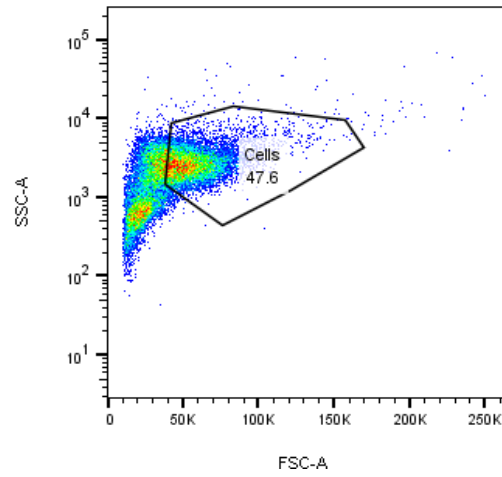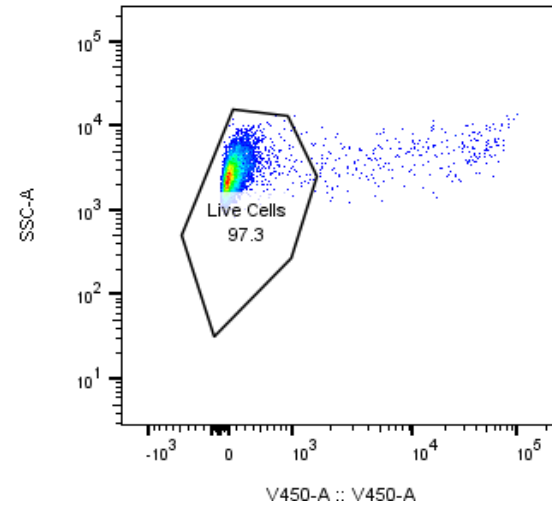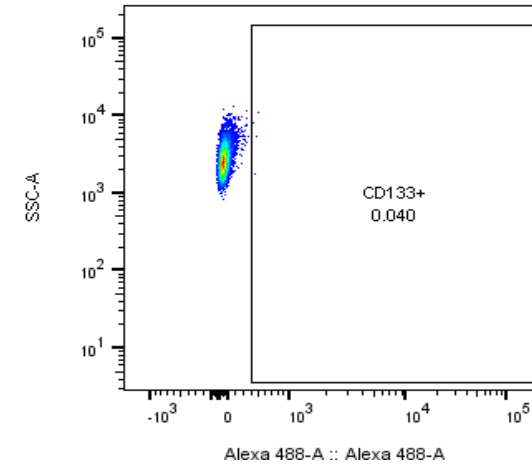

## CD133 GSC

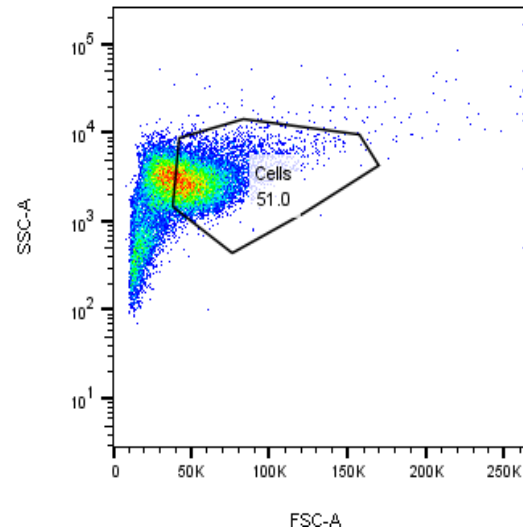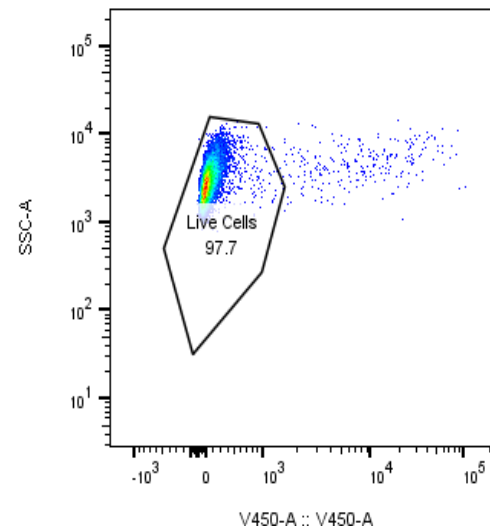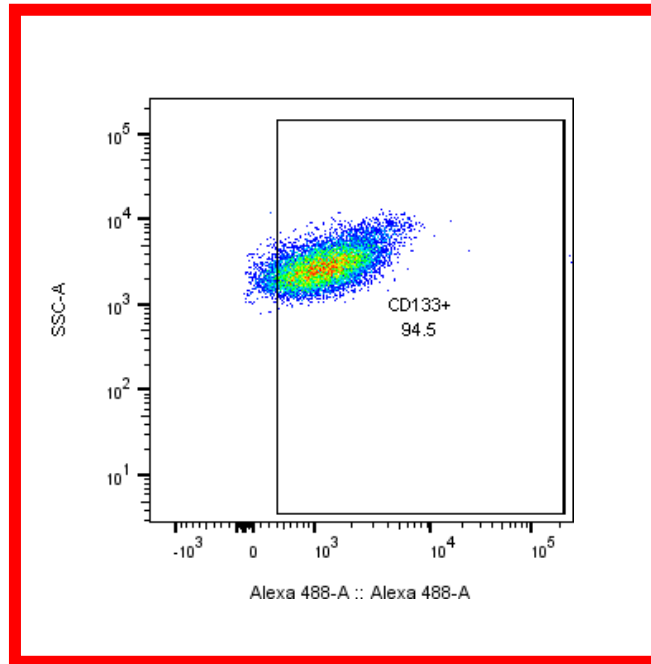

## IgG DGC

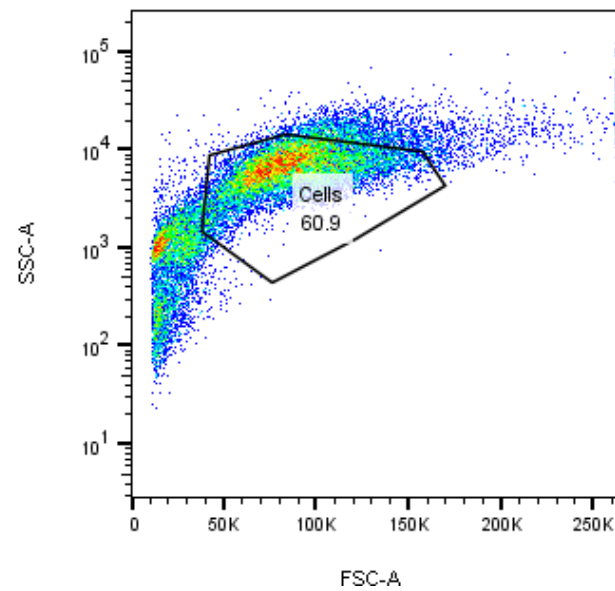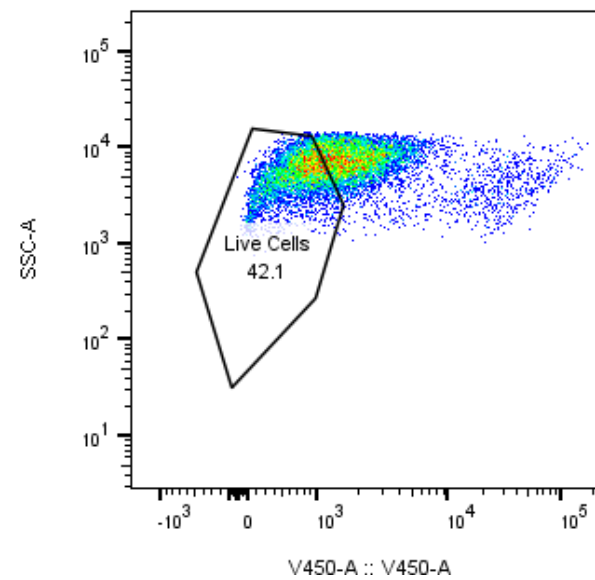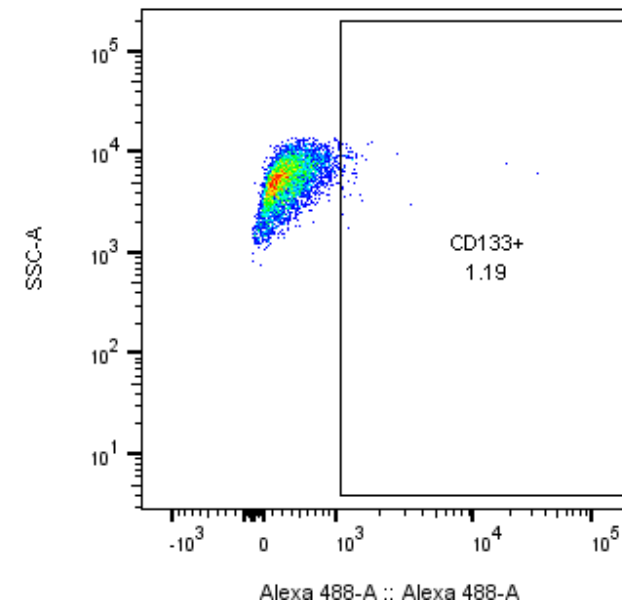

## CD133 DGC

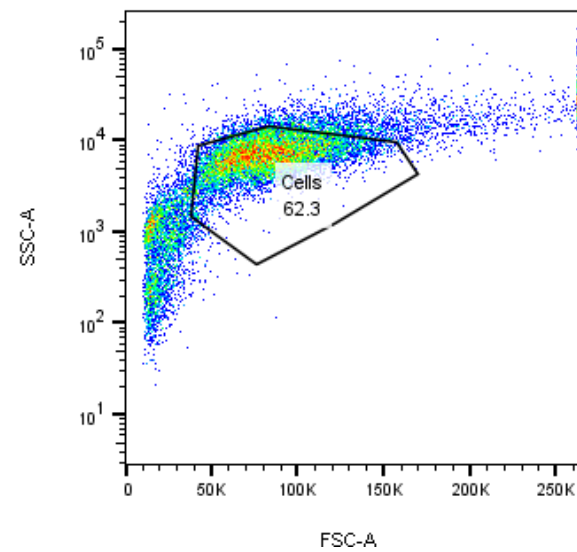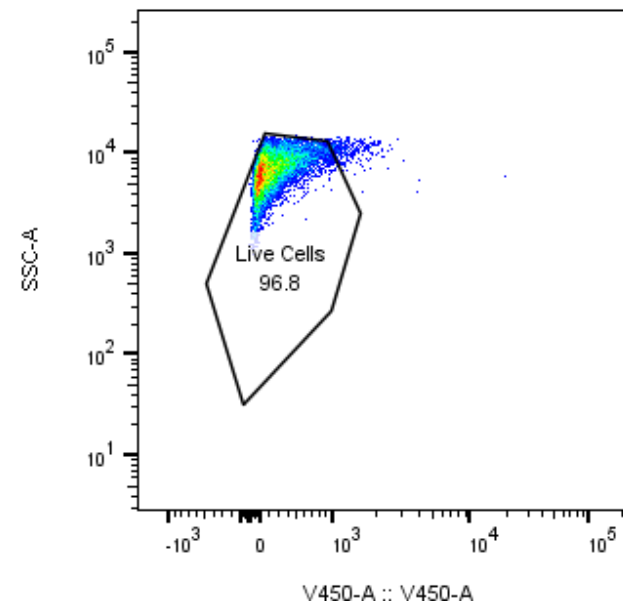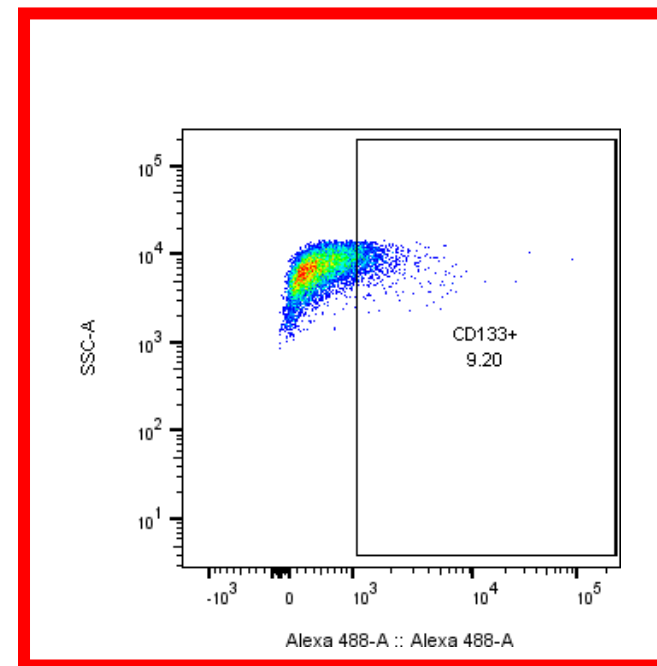

Supplement: Figure 6—source data 1. [file elife-78972-fig6-data1.zip › Figure 6-Source data A/ALL ANALYSIS PLOTS FOR PANEL A.pdf]

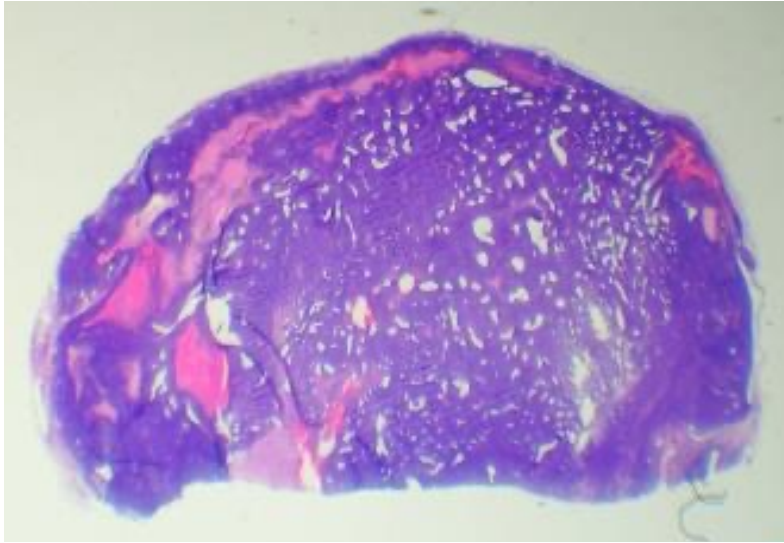

**MGG8-GSC/shNT**

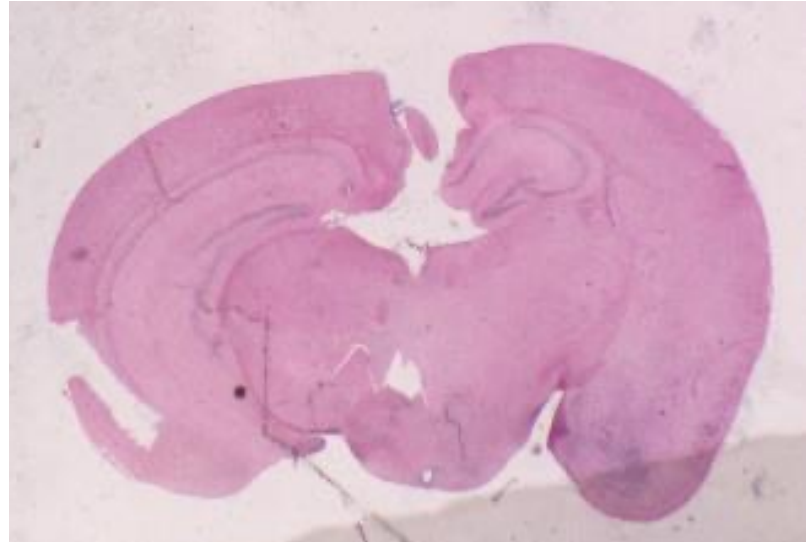

**MGG8-GSC/shFMOD**

Supplement: Figure 6—source data 3. [file elife-78972-fig6-data3.zip › Figure 6-Source data C/IMAGES FOR PANEL C.pdf]

MGG8-GSC/shNT

**FMOD**

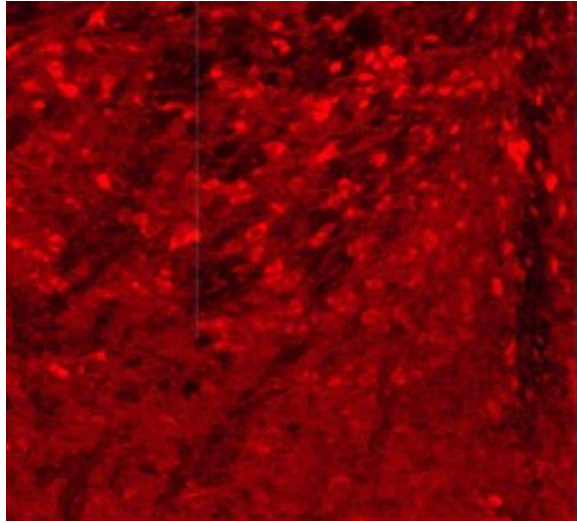

**H33342**

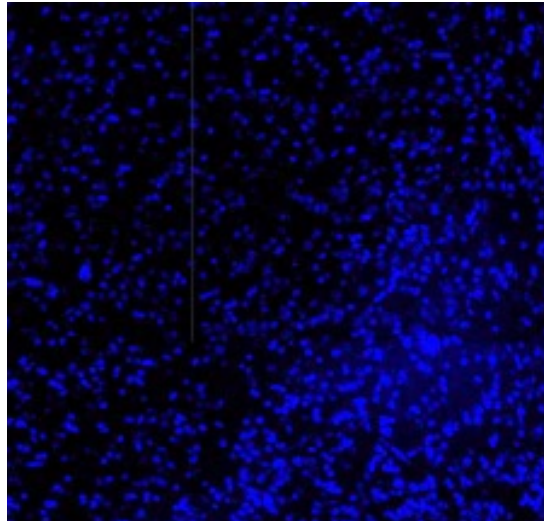

**MERGE**

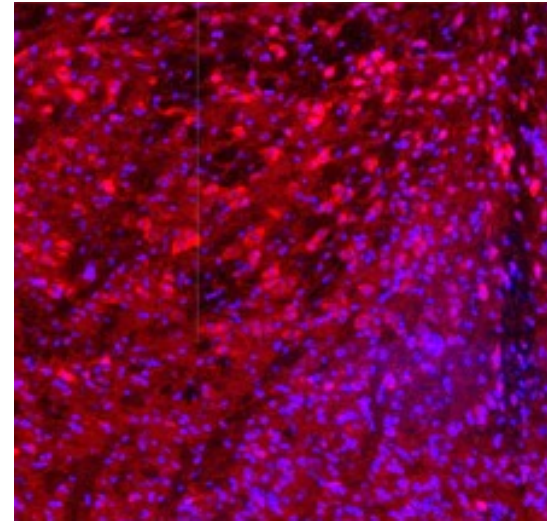

MGG8-GSC/shFMOD

**FMOD**

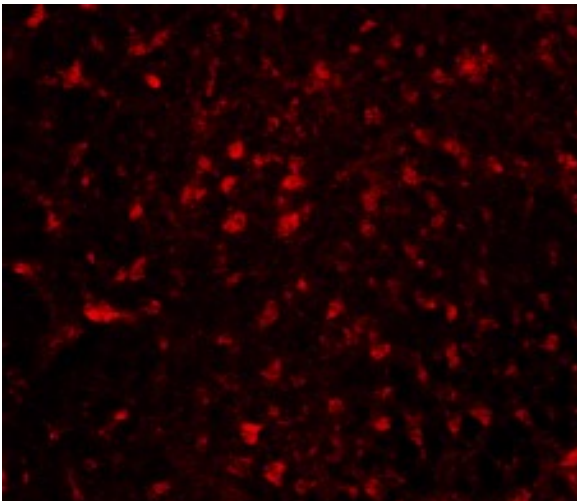

**H33342**

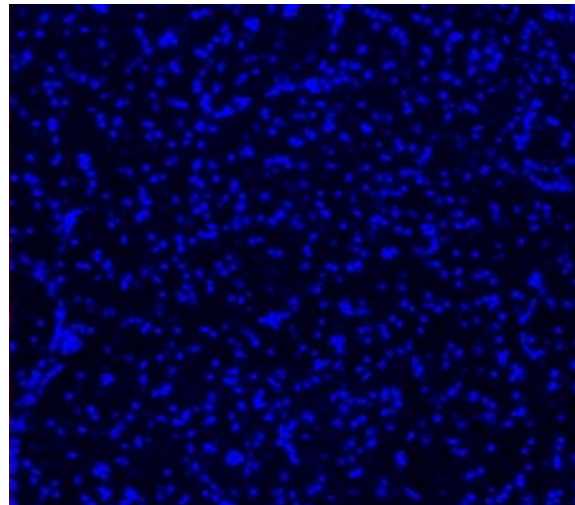

**MERGE**

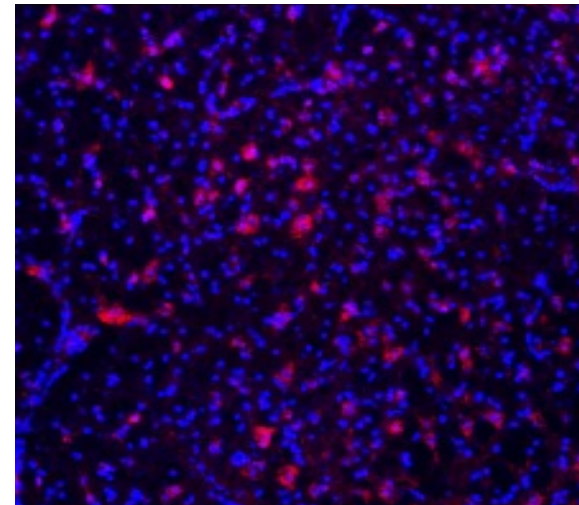

Supplement: Figure 6—source data 4. [file elife-78972-fig6-data4.zip › Figure 6-Source data D/IMAGES OR PANEL D.pdf]

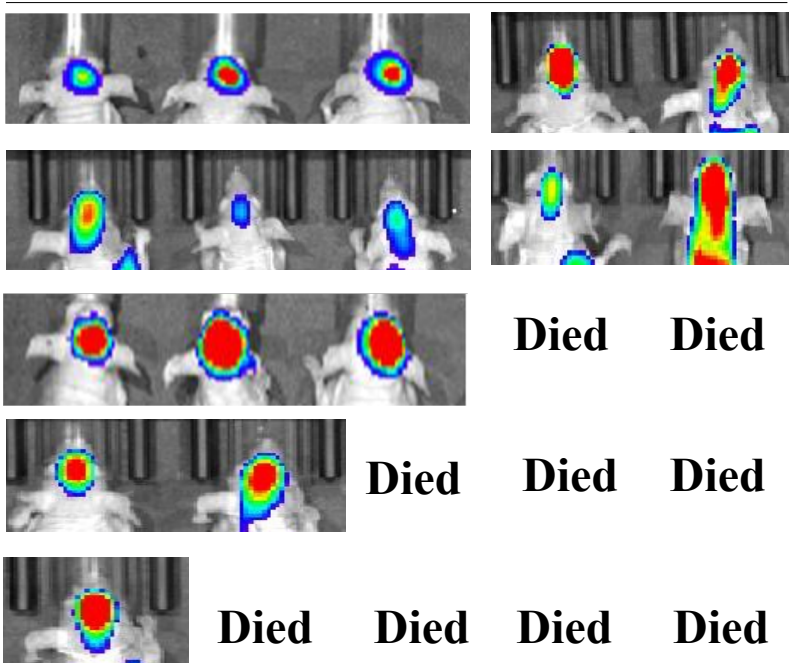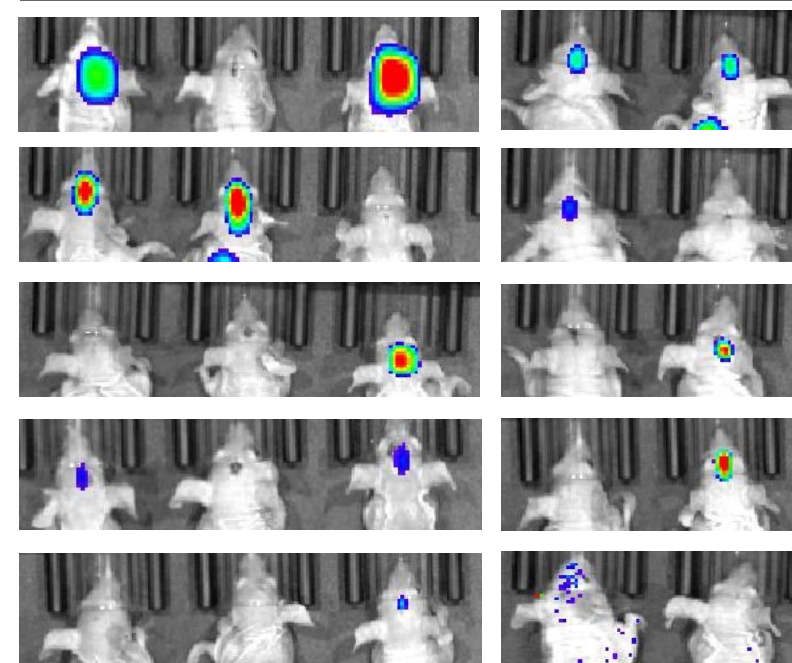

Supplement: Figure 6—figure supplement 1—source data 1. [file elife-78972-fig6-figsupp1-data1.zip › Figure 6-Figure Supplement 1-Source data/Figure 6-Figure Supplement 1-Source Data A/in vivo images for panel a.pdf]

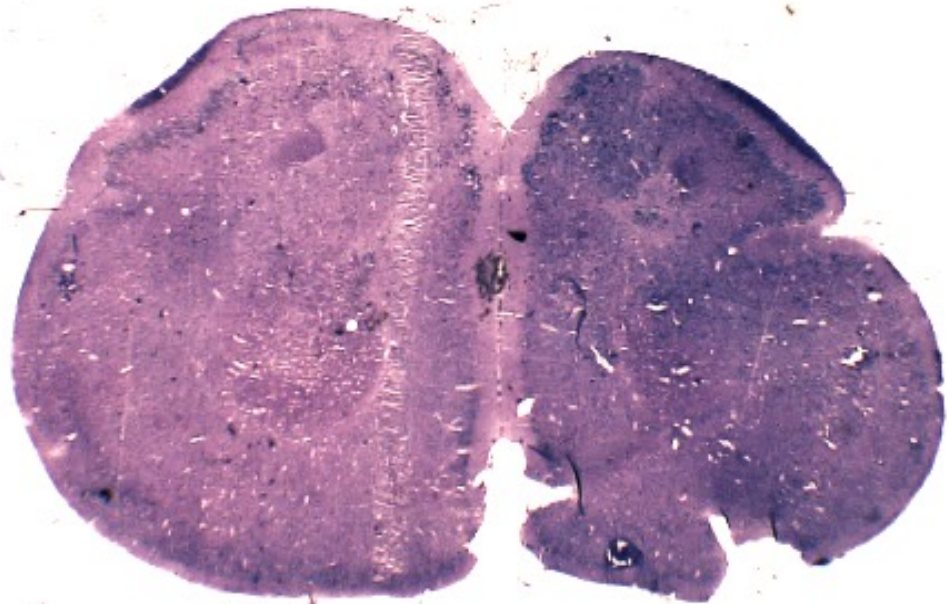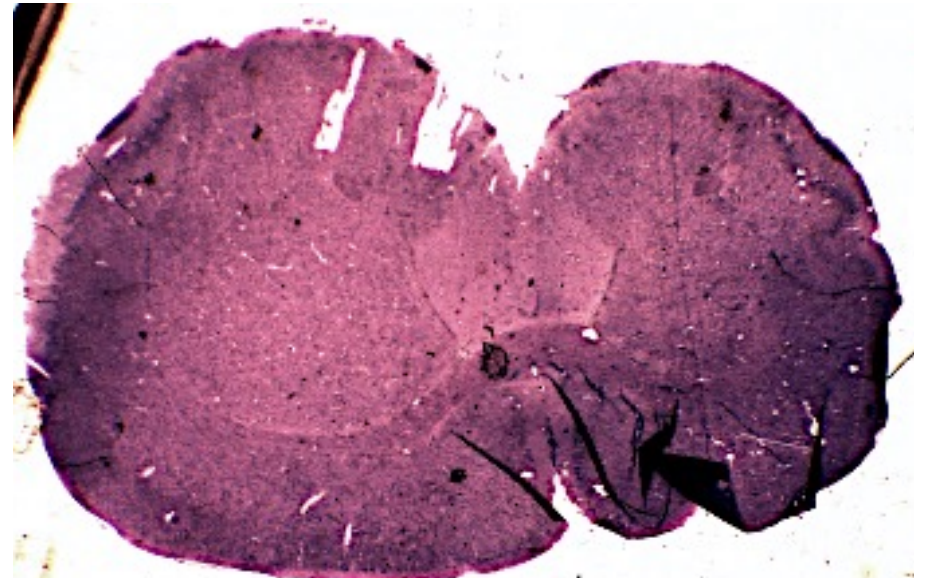

Supplement: Figure 6—figure supplement 1—source data 1. [file elife-78972-fig6-figsupp1-data1.zip › Figure 6-Figure Supplement 1-Source data/Figure 6-Figure Supplement 1-Source Data D/hne images for panel d.pdf]

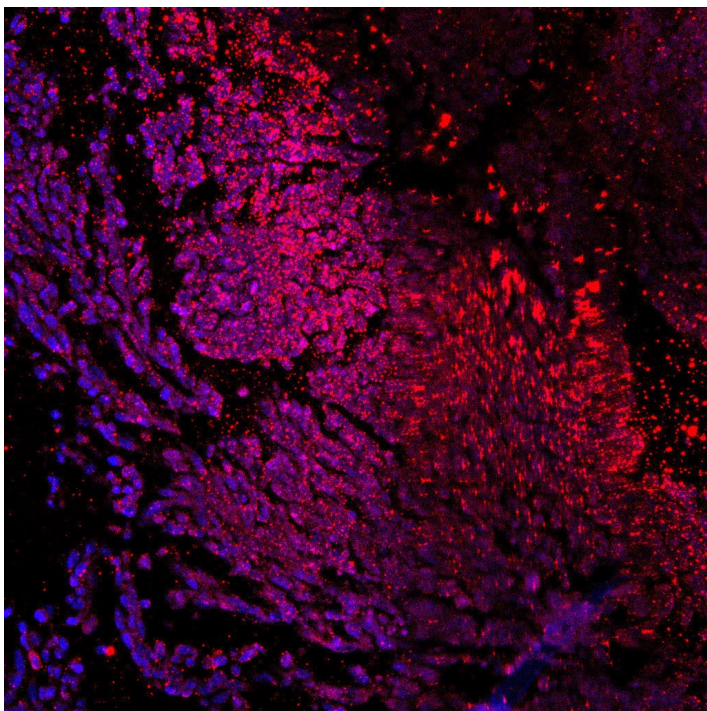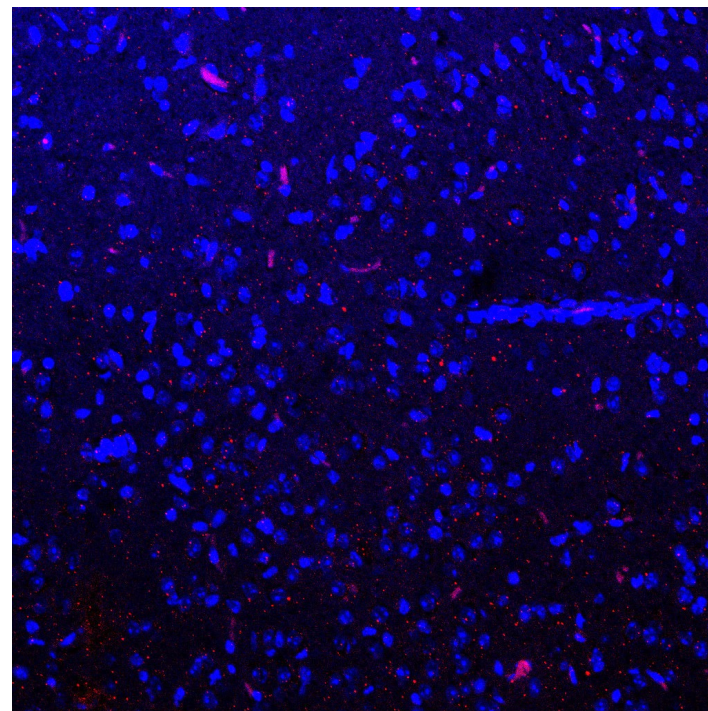

Supplement: Figure 6—figure supplement 1—source data 1. [file elife-78972-fig6-figsupp1-data1.zip › Figure 6-Figure Supplement 1-Source data/Figure 6-Figure Supplement 1-Source Data E/confocal images for panel e.pdf]

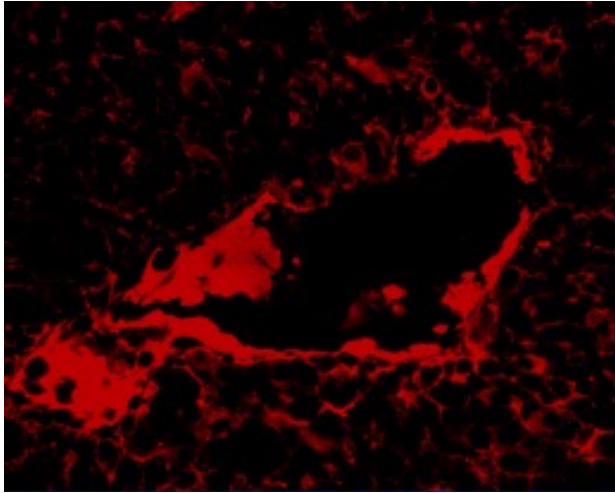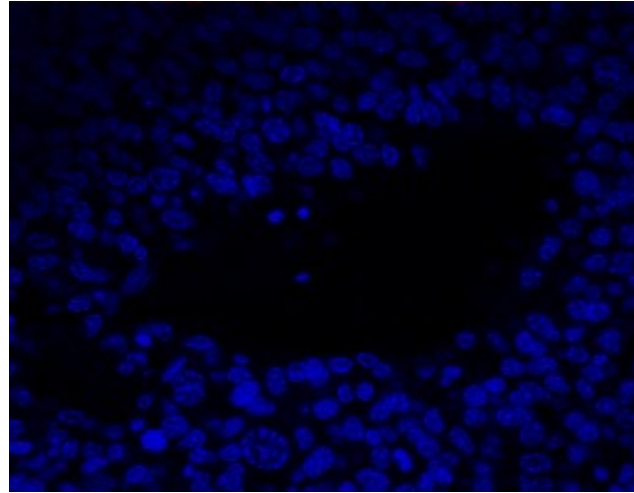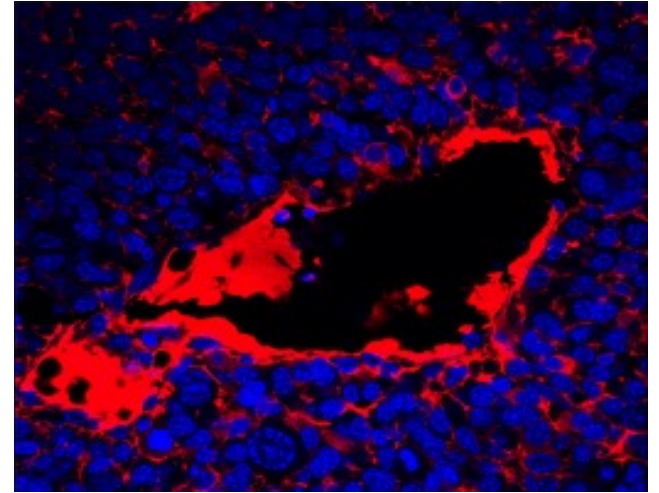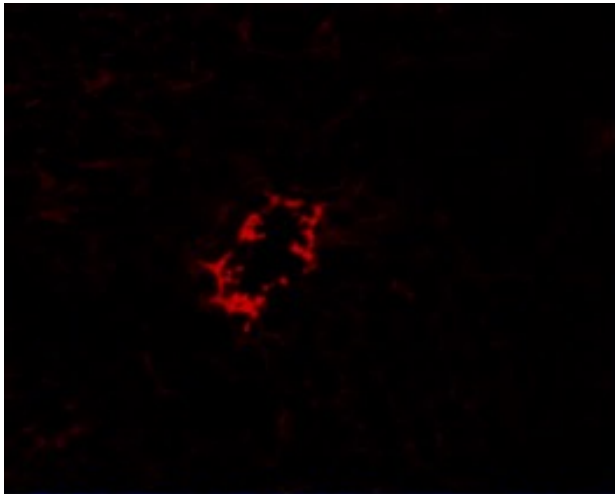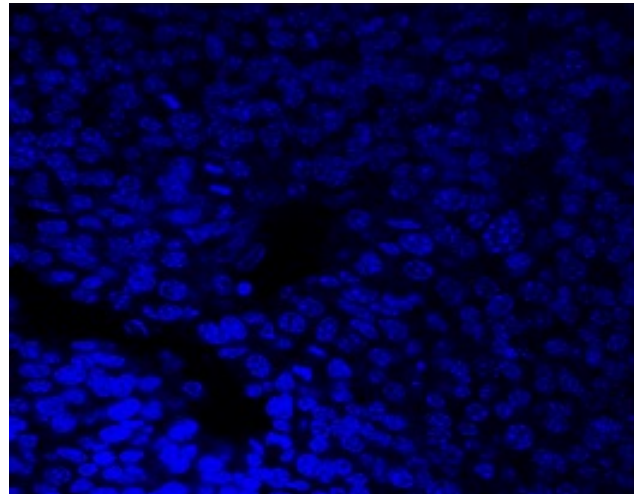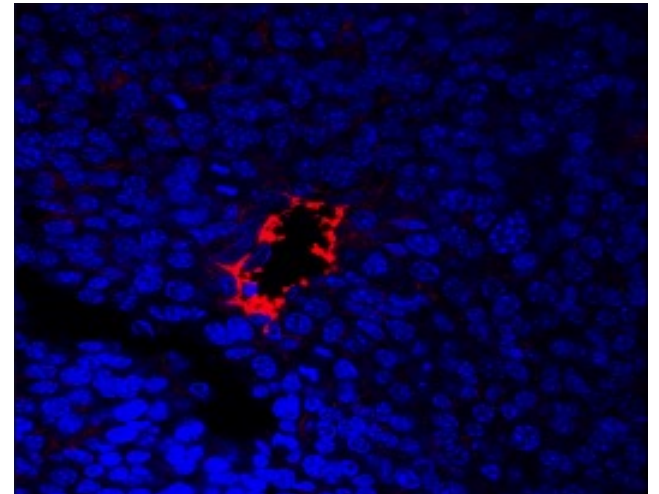

Supplement: Figure 7—source data 1. [file elife-78972-fig7-data1.zip › Figure 7-Source data A/IMAGES FOR PANEL A.pdf]

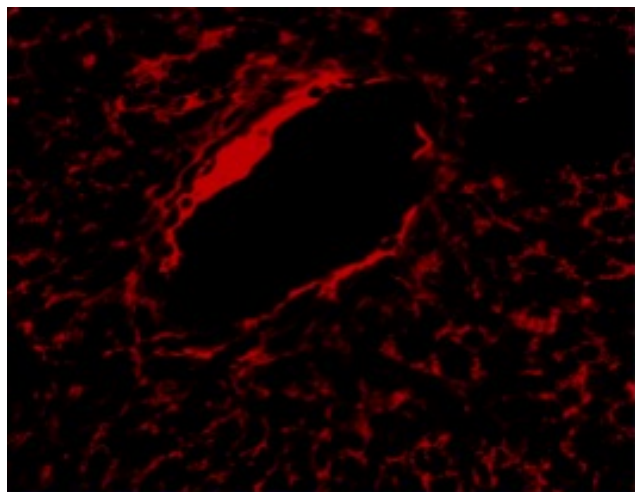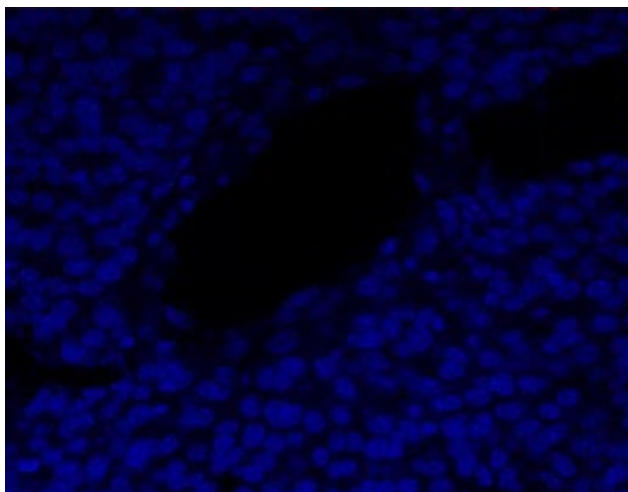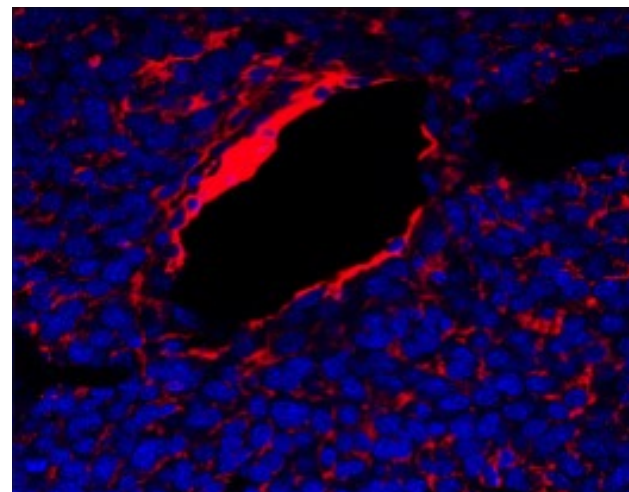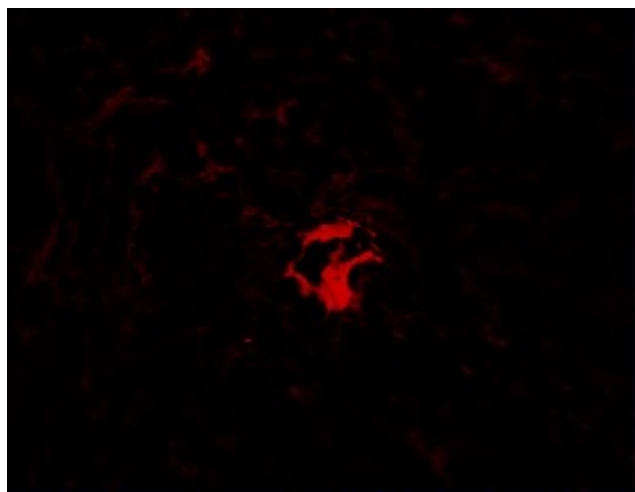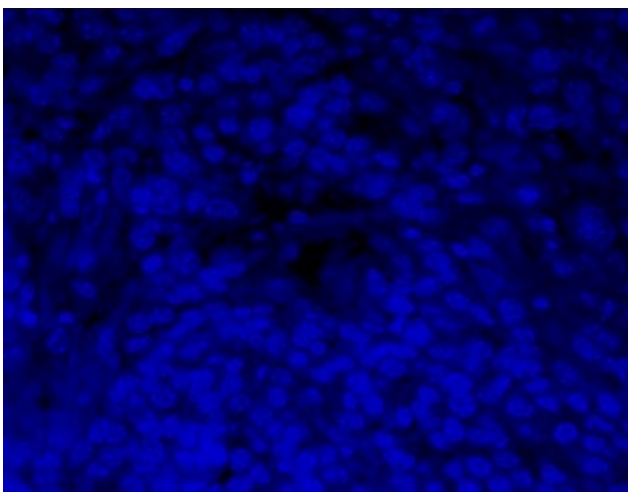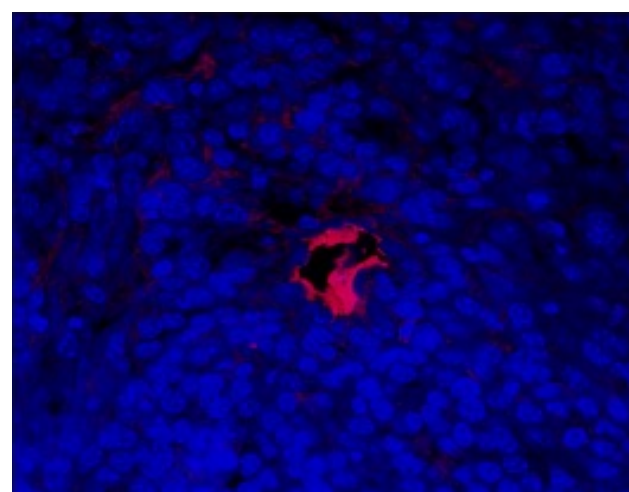

Supplement: Figure 7—source data 3. [file elife-78972-fig7-data3.zip › Figure 7-Source data D/IMAGES FOR PANEL D.pdf]

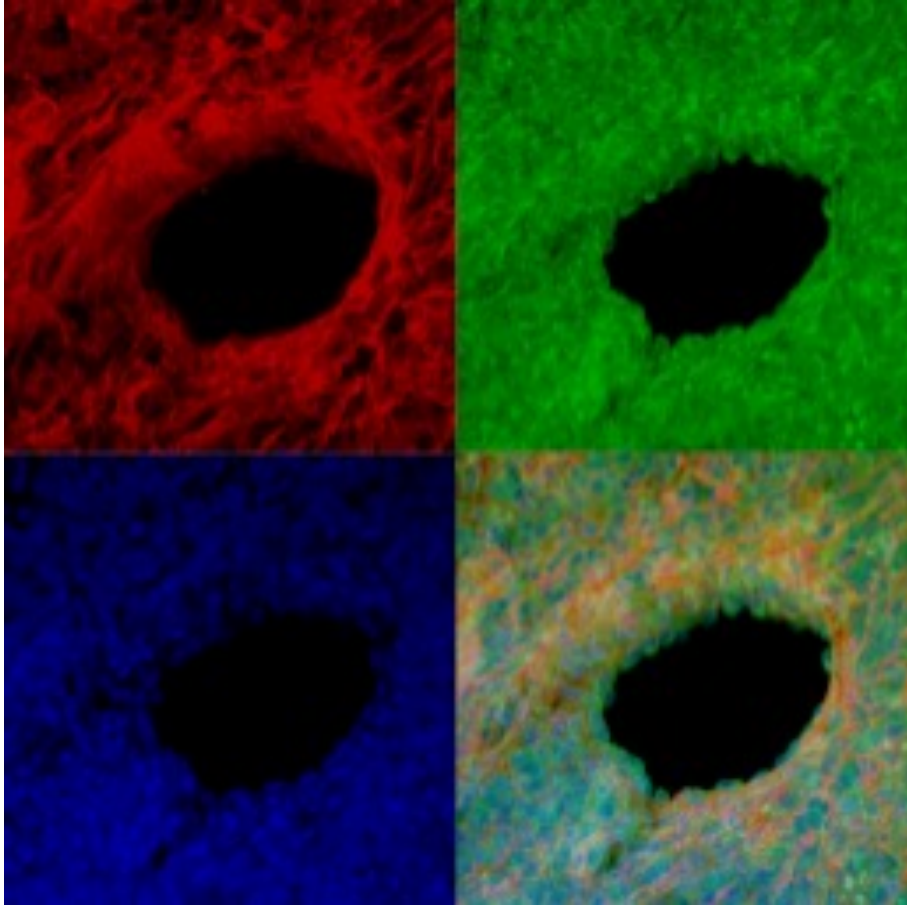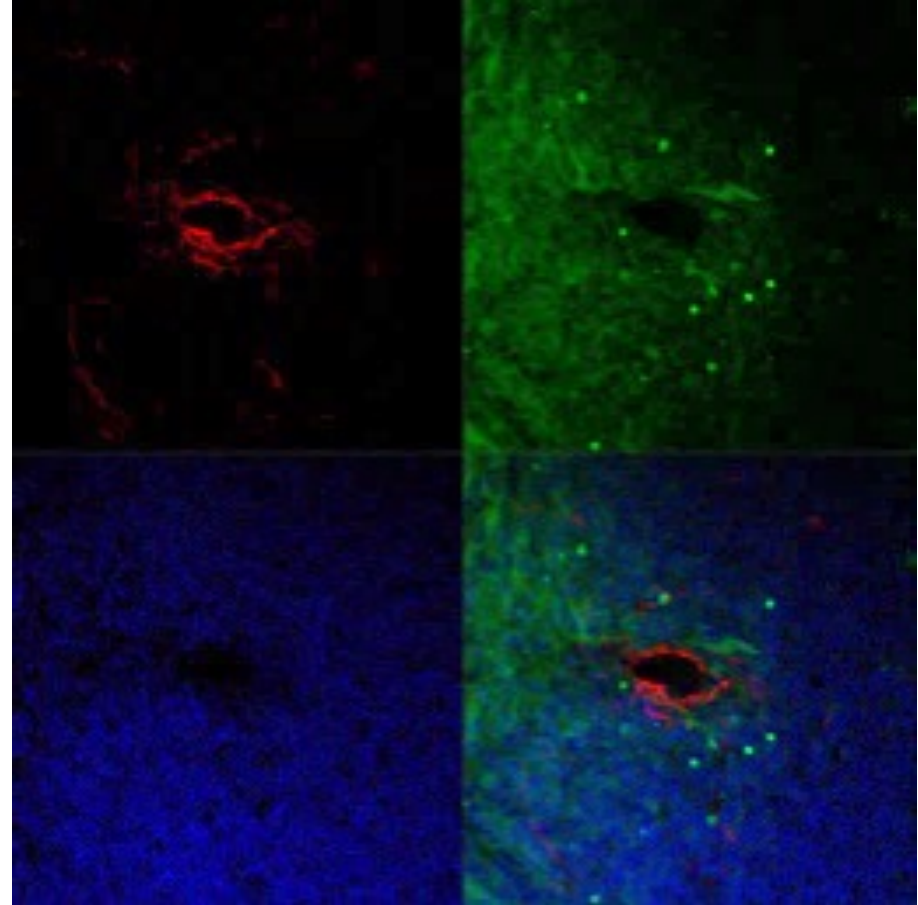

Supplement: Figure 7—source data 5. [file elife-78972-fig7-data5.zip › Figure 7-Source data G/IMAGES FOR PANEL G.pdf]

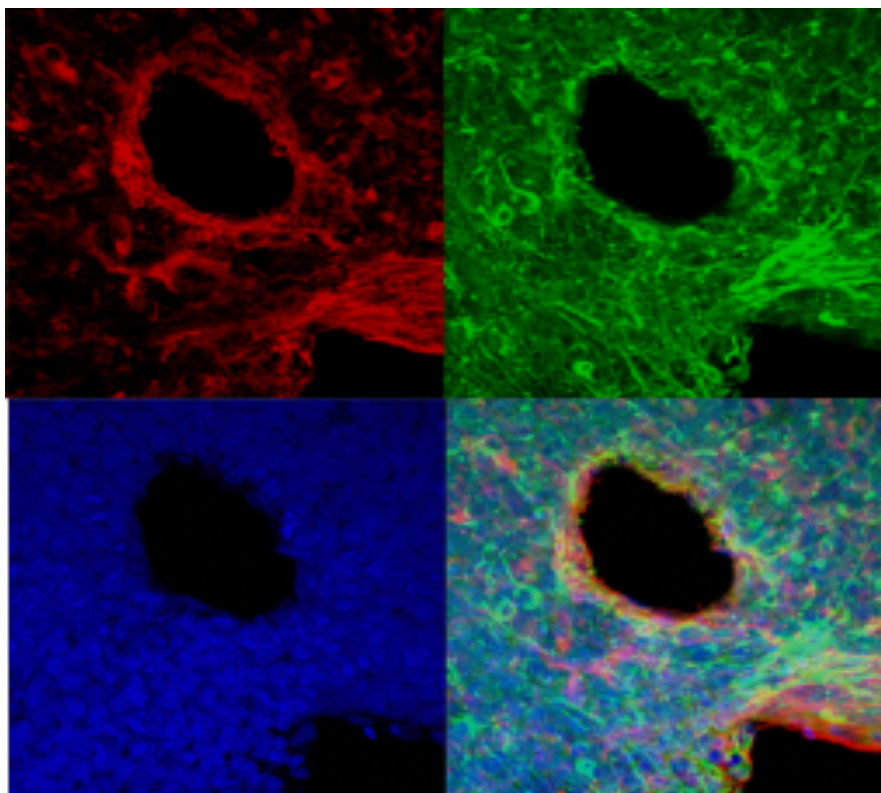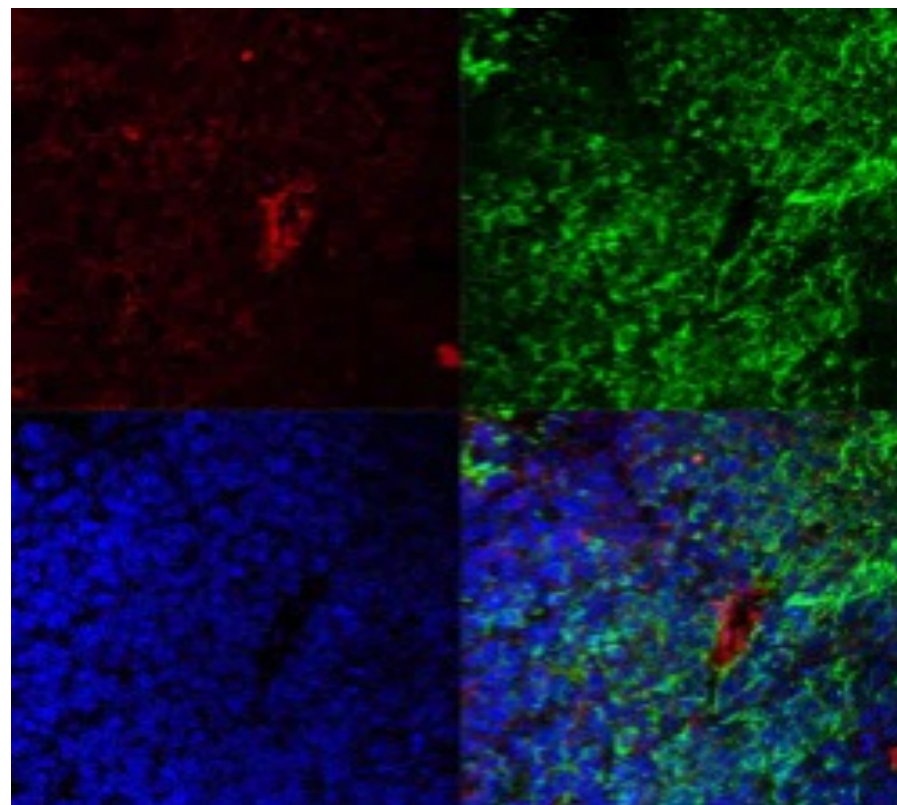

Supplement: Figure 7—source data 6. [file elife-78972-fig7-data6.zip › Figure 7-Source data J/IMAGES FOR PANEL J.pdf]

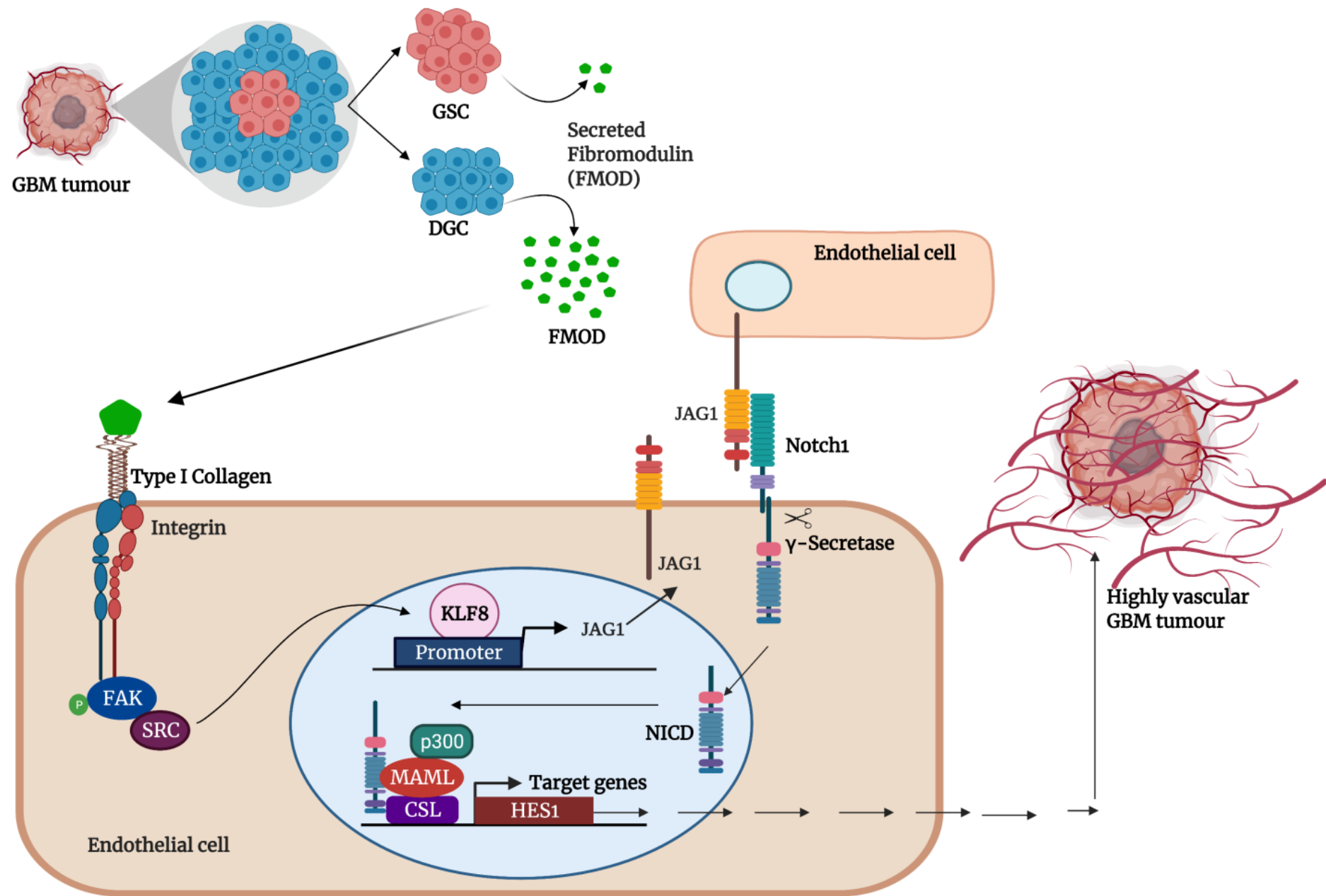

Supplement: Figure 7—source data 7. [file elife-78972-fig7-data7.zip › Figure 7-Source data M/IMAGE FOR PANEL M.pdf]

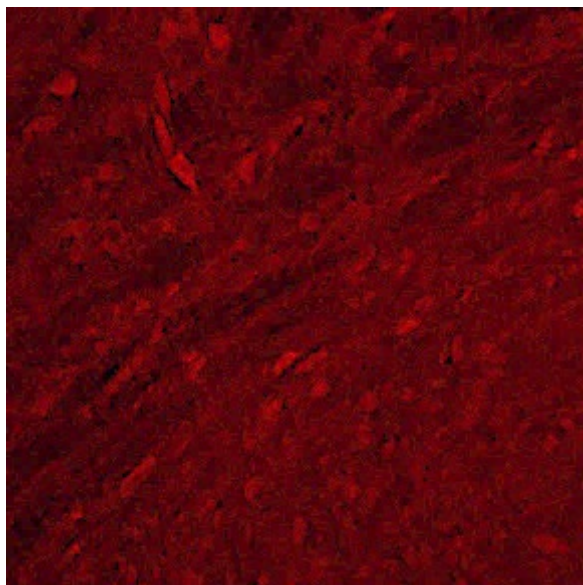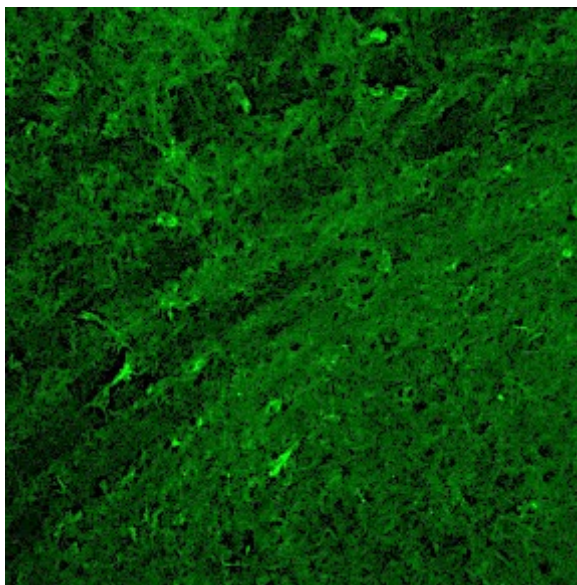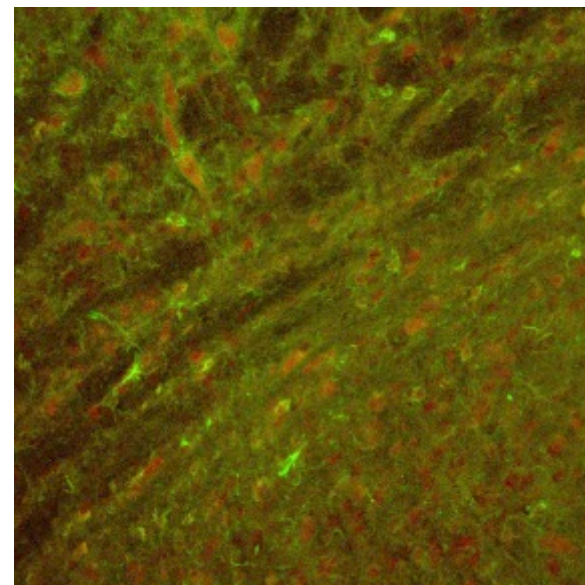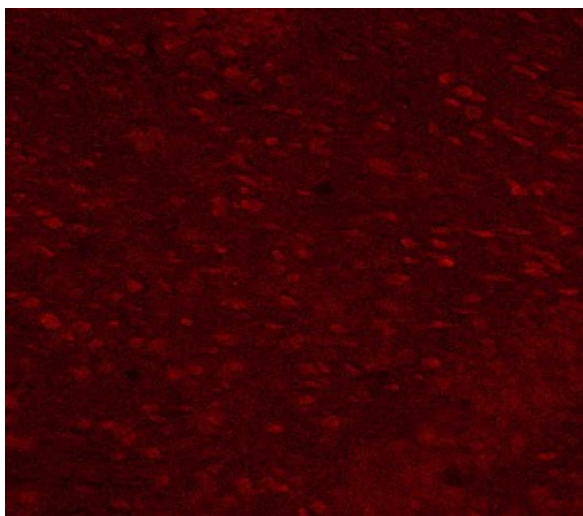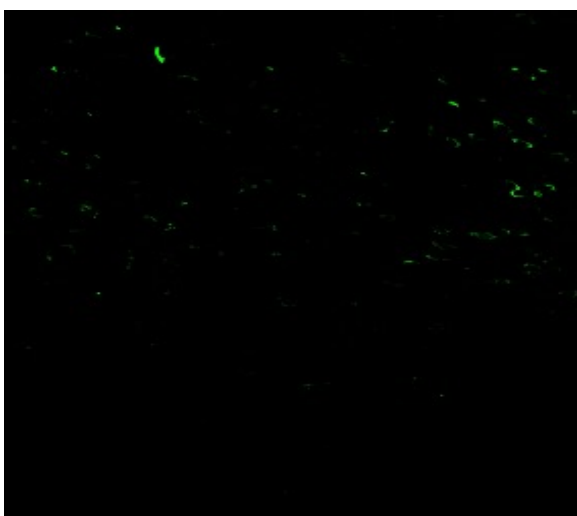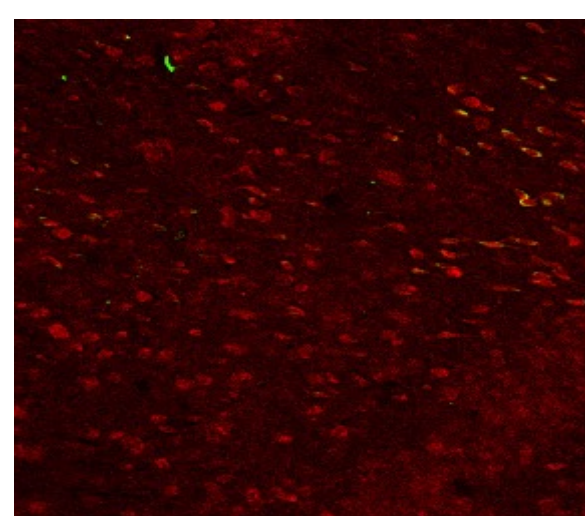

Supplement: Figure 7—figure supplement 1—source data 1. [file elife-78972-fig7-figsupp1-data1.zip › Figure 7-Figure Supplement 1-Source Data/Figure 7-Figure Supplement 1-Source Data A/images for panel a.pdf]

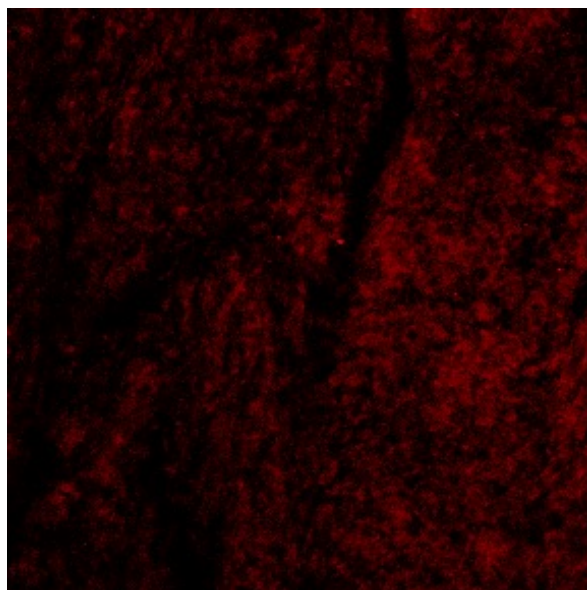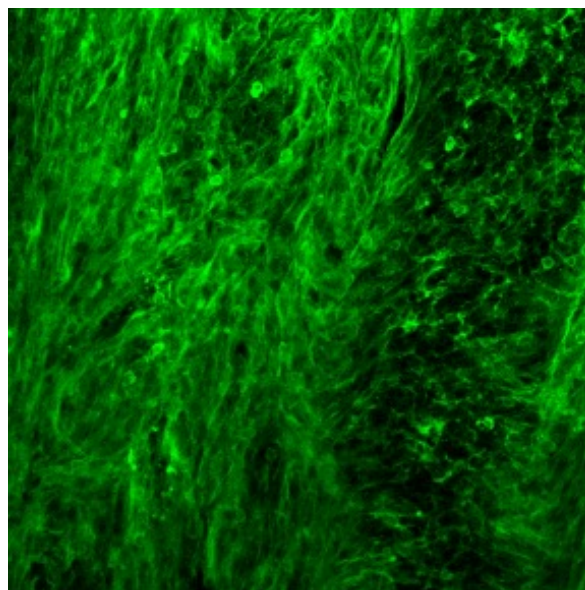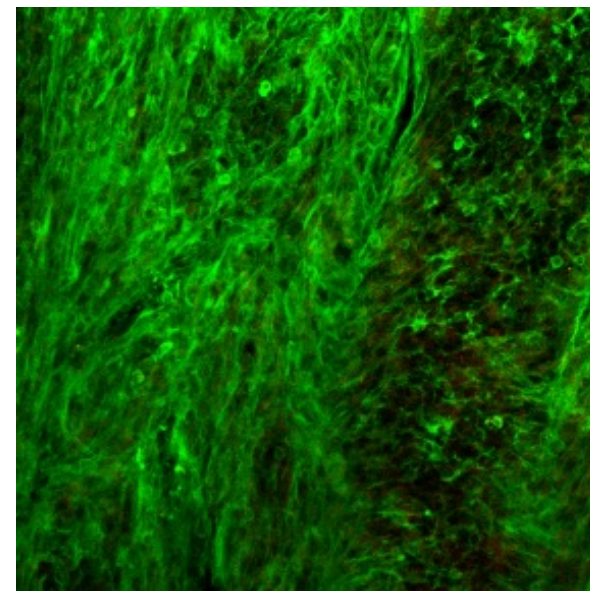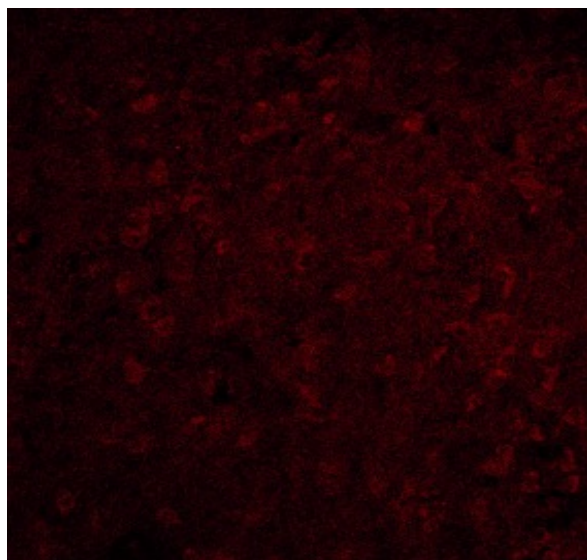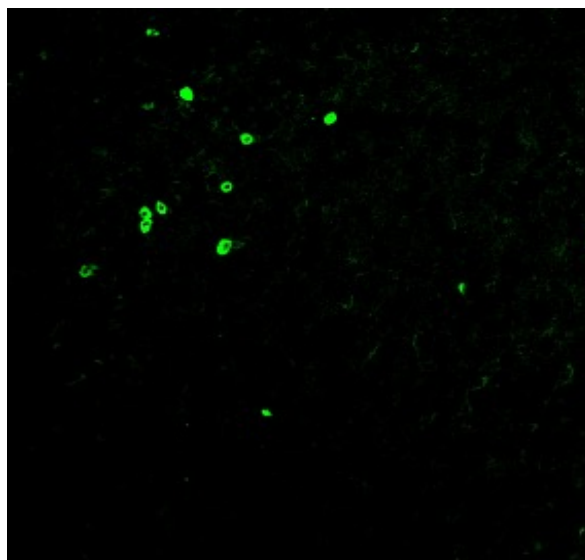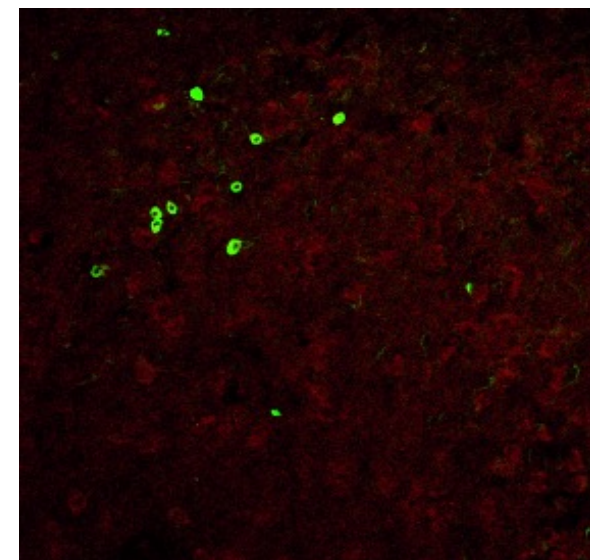

Supplement: Figure 7—figure supplement 1—source data 1. [file elife-78972-fig7-figsupp1-data1.zip › Figure 7-Figure Supplement 1-Source Data/Figure 7-Figure Supplement 1-Source Data D/images for panel d.pdf]

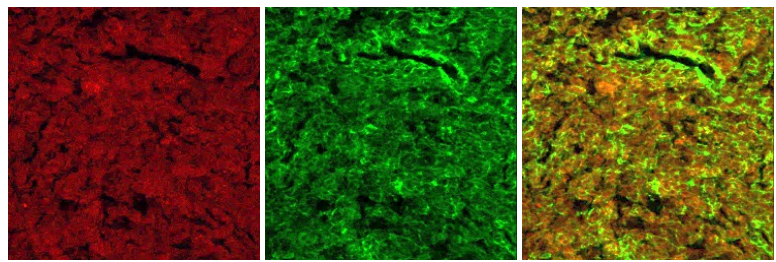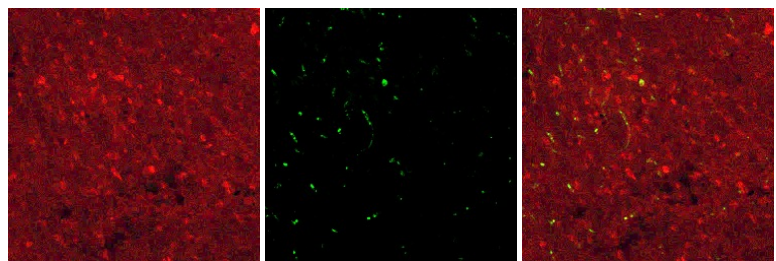

Supplement: Figure 7—figure supplement 2—source data 1. [file elife-78972-fig7-figsupp2-data1.zip › Figure 7-Figure Supplement 2-Source Data/Figure 7-Figure Supplement 2-Source Data A/A.pdf]

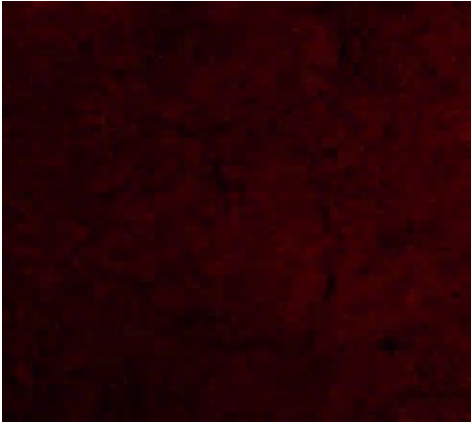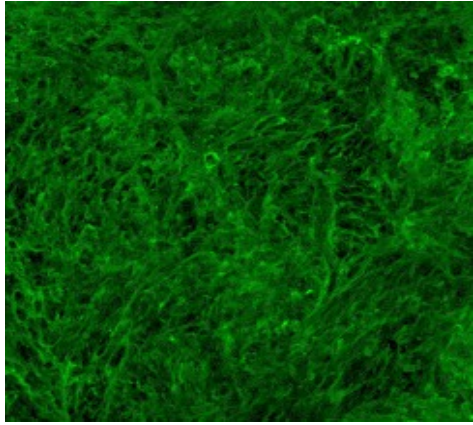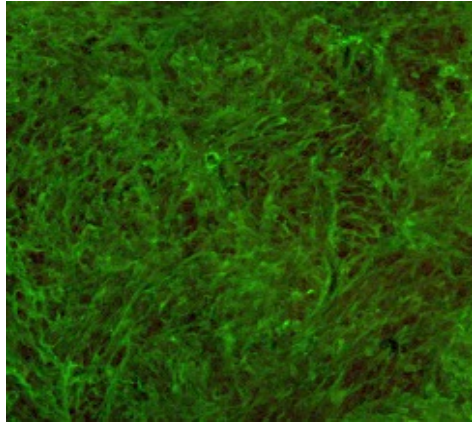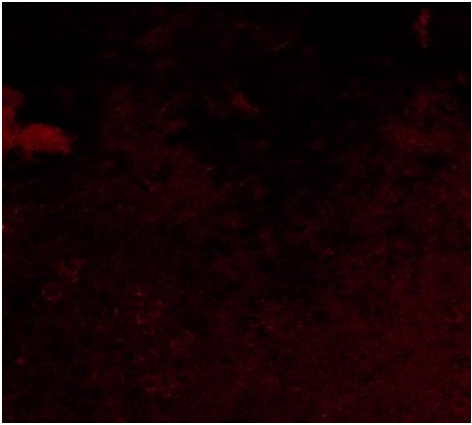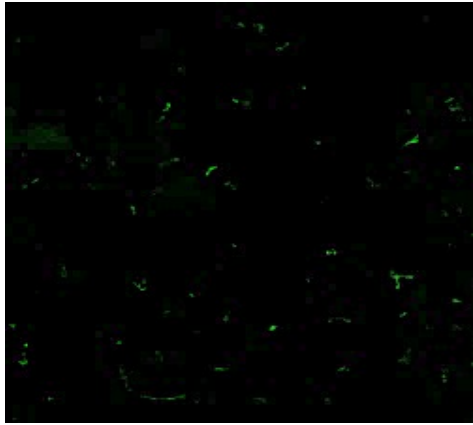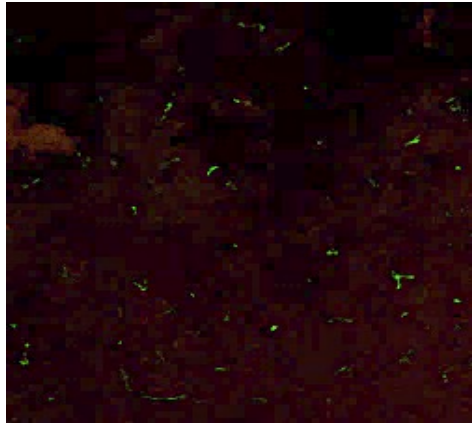

Supplement: Figure 7—figure supplement 2—source data 1. [file elife-78972-fig7-figsupp2-data1.zip › Figure 7-Figure Supplement 2-Source Data/Figure 7-Figure Supplement 2-Source Data D/D.pdf]

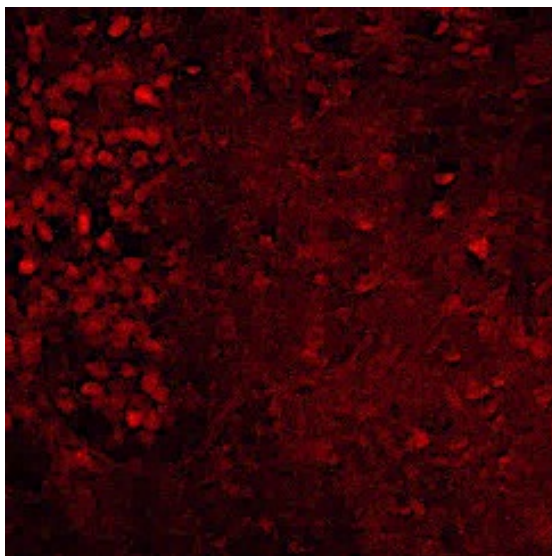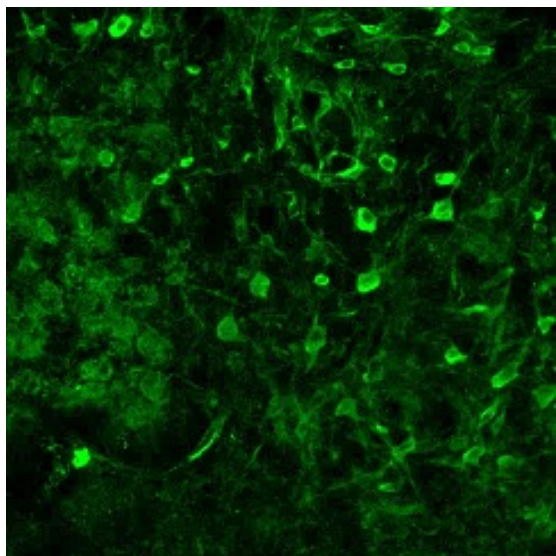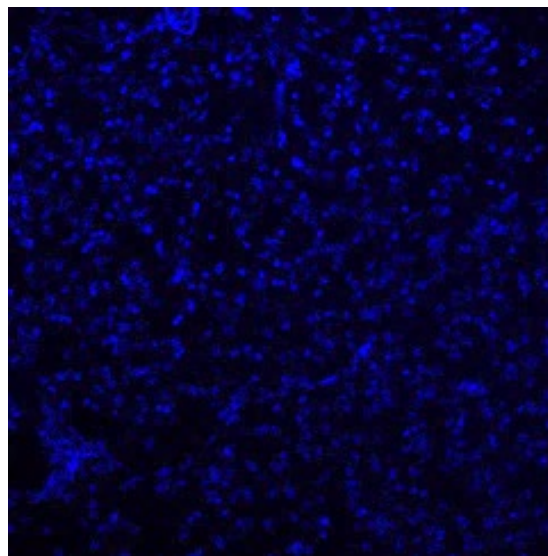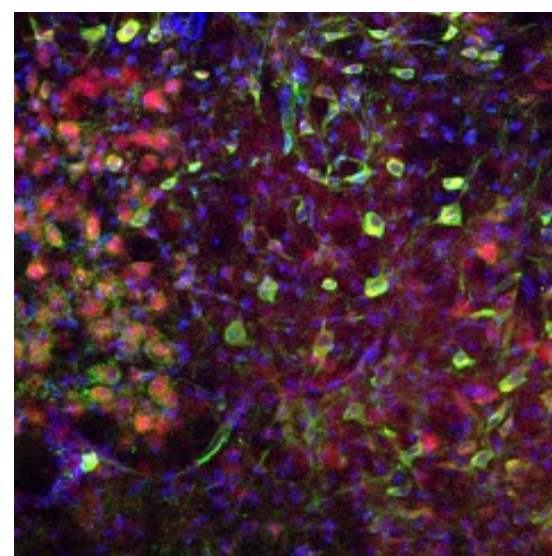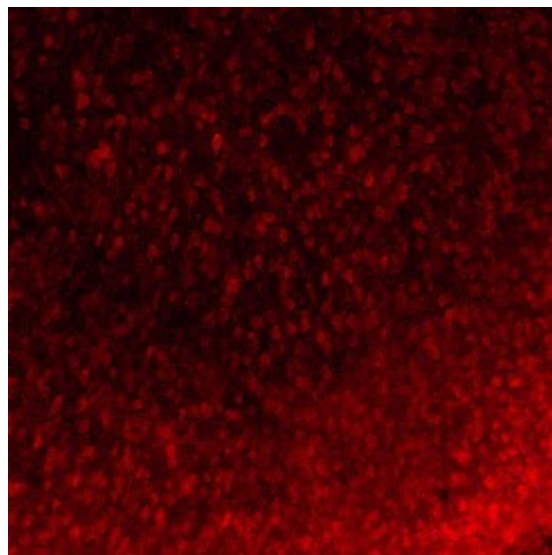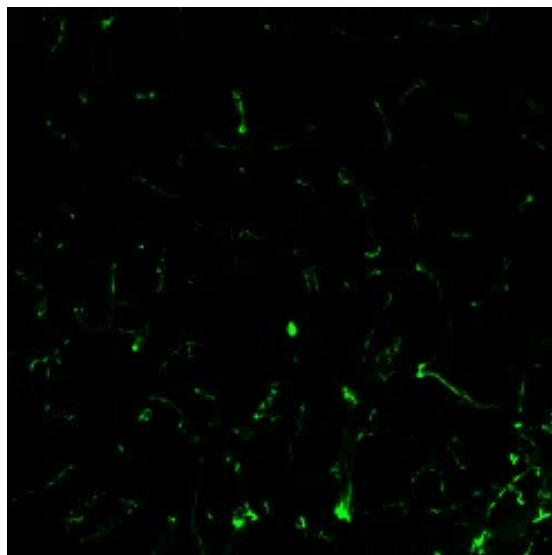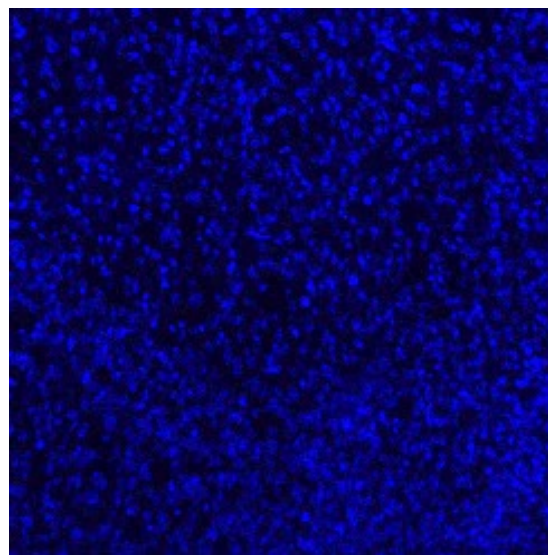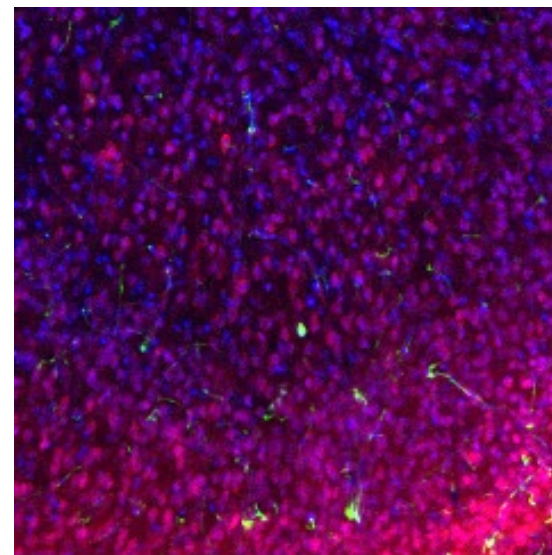

Supplement: Figure 7—figure supplement 3—source data 1. [file elife-78972-fig7-figsupp3-data1.zip › Figure 7-Figure Supplement 3-Source Data/Figure 7-Figure Supplement 3-Source Data A/A.pdf]

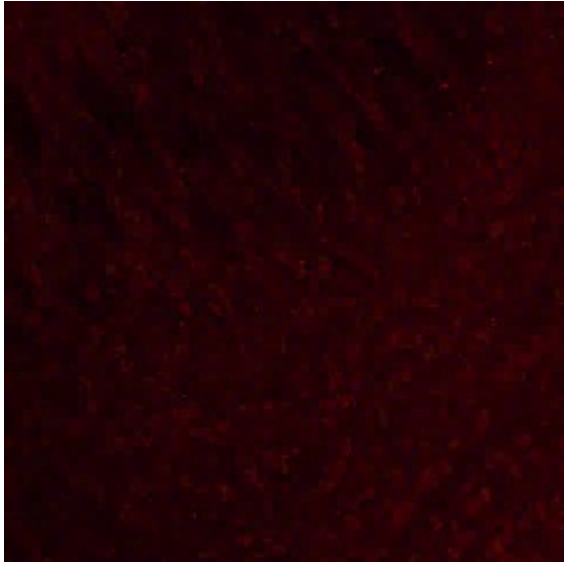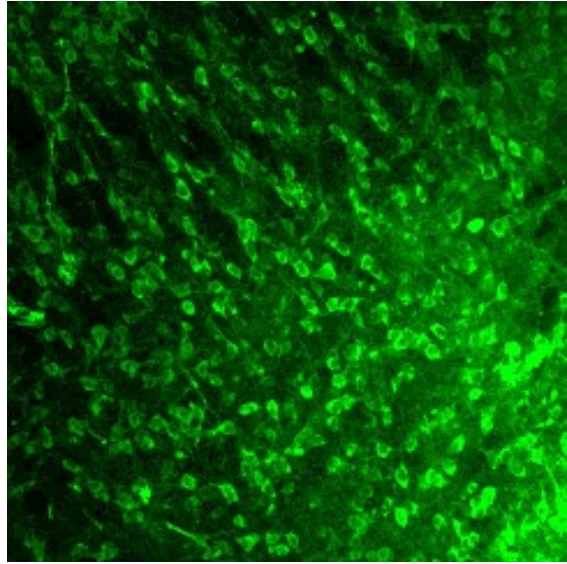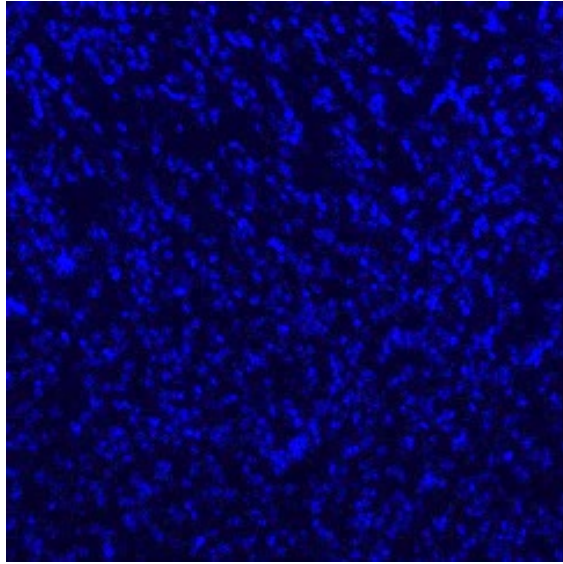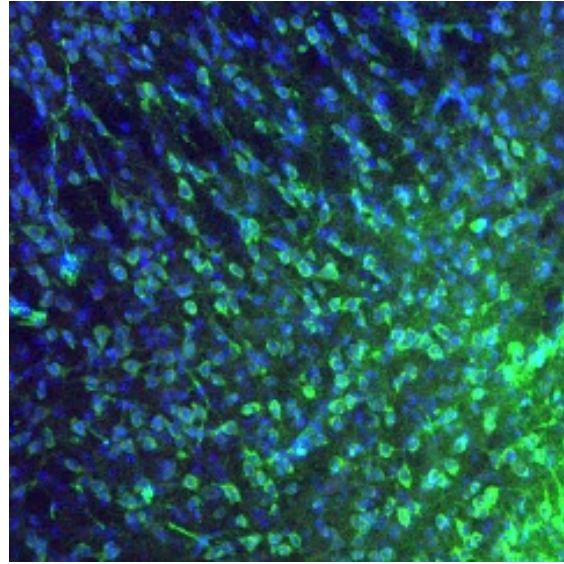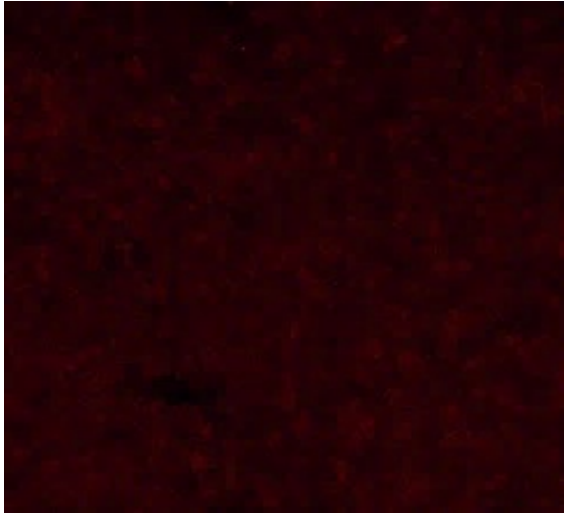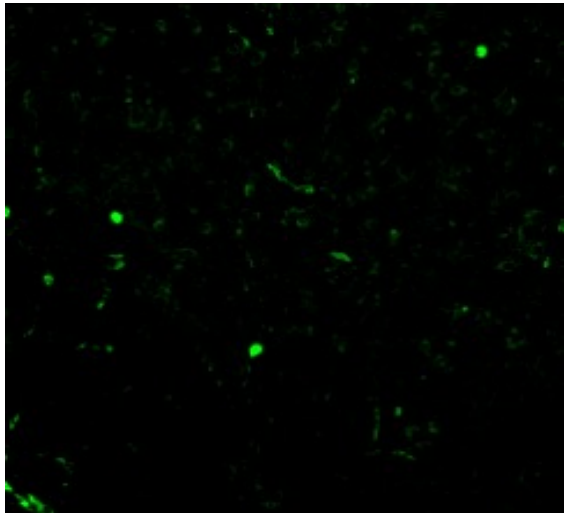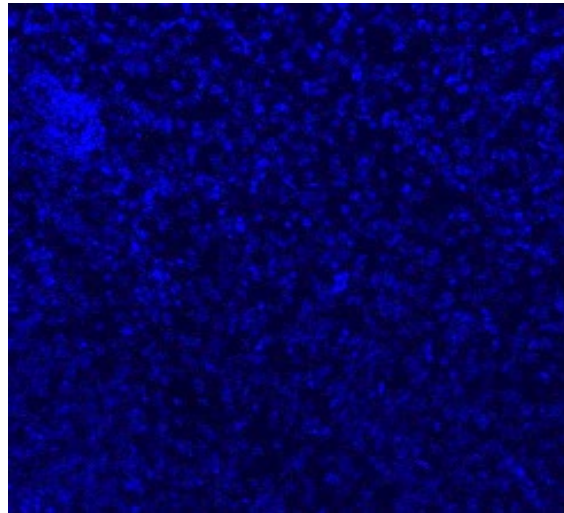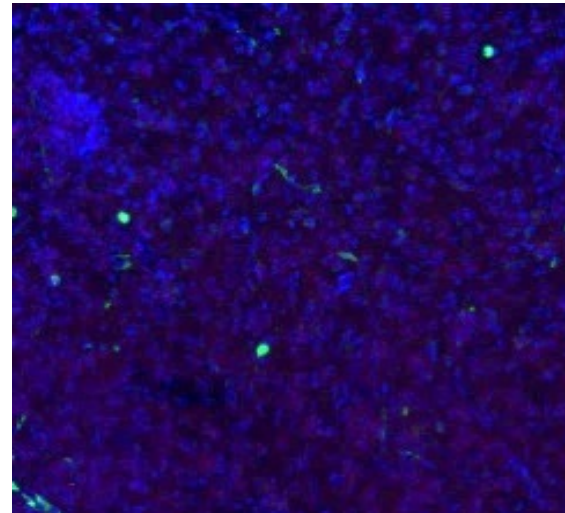

Supplement: Figure 7—figure supplement 3—source data 1. [file elife-78972-fig7-figsupp3-data1.zip › Figure 7-Figure Supplement 3-Source Data/Figure 7-Figure Supplement 3-Source Data D/D.pdf]

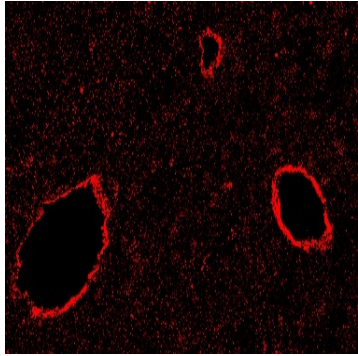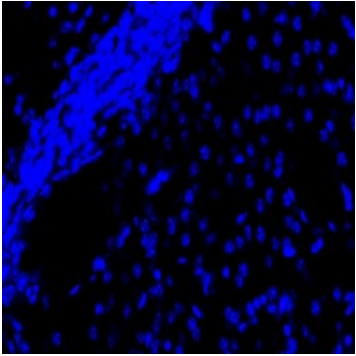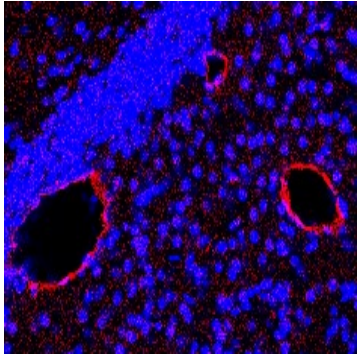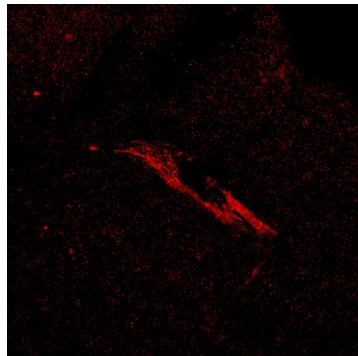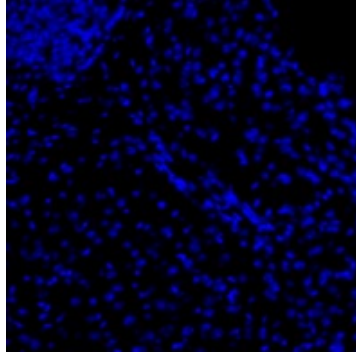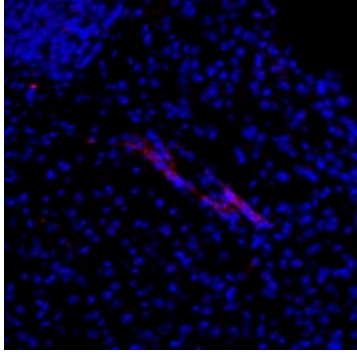

Supplement: Figure 7—figure supplement 4—source data 1. [file elife-78972-fig7-figsupp4-data1.zip › Figure 7-Figure Supplement 4-Source Data/Figure 7-Figure Supplement 4-Source Data A/A.pdf]

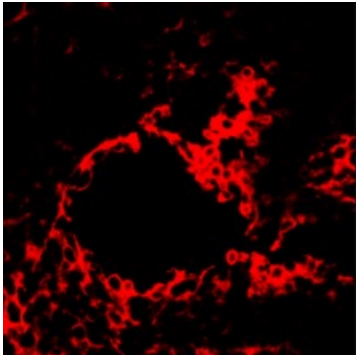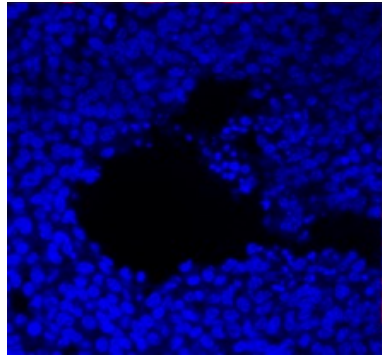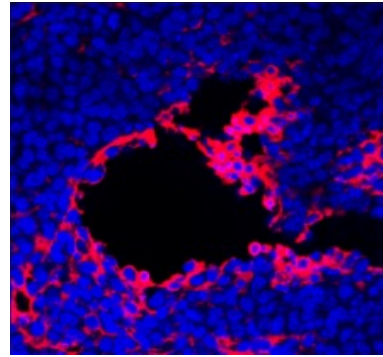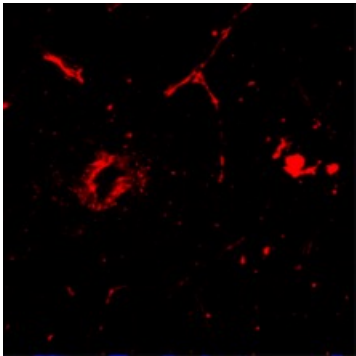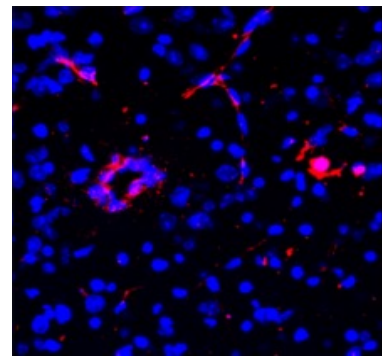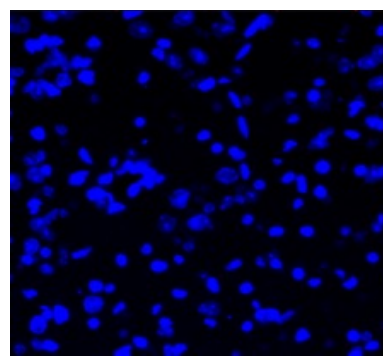

Supplement: Figure 7—figure supplement 4—source data 1. [file elife-78972-fig7-figsupp4-data1.zip › Figure 7-Figure Supplement 4-Source Data/Figure 7-Figure Supplement 4-Source Data D/D.pdf]

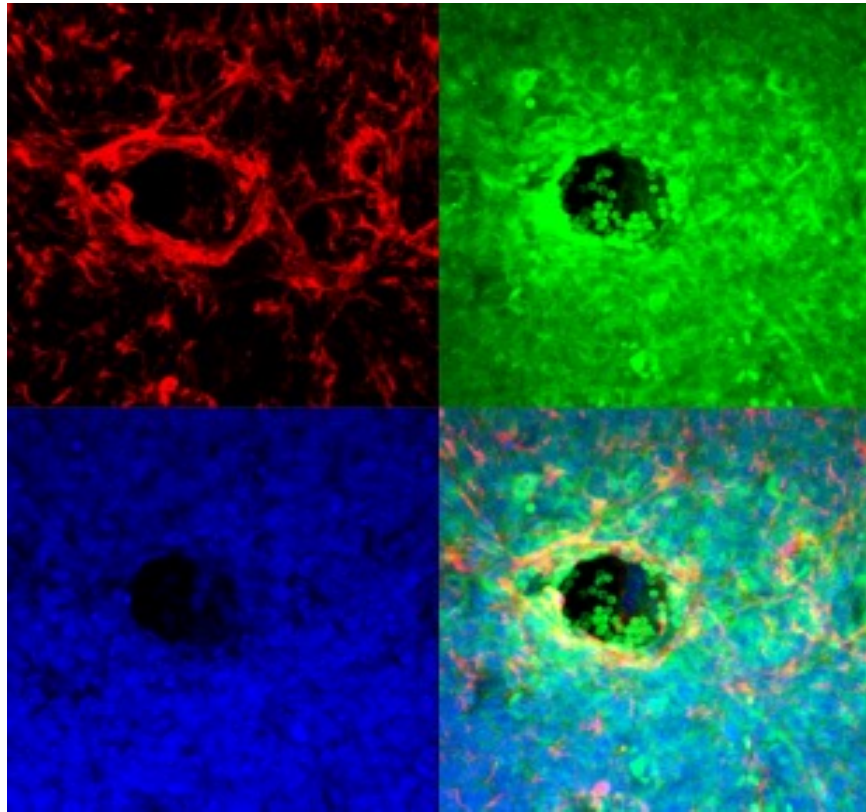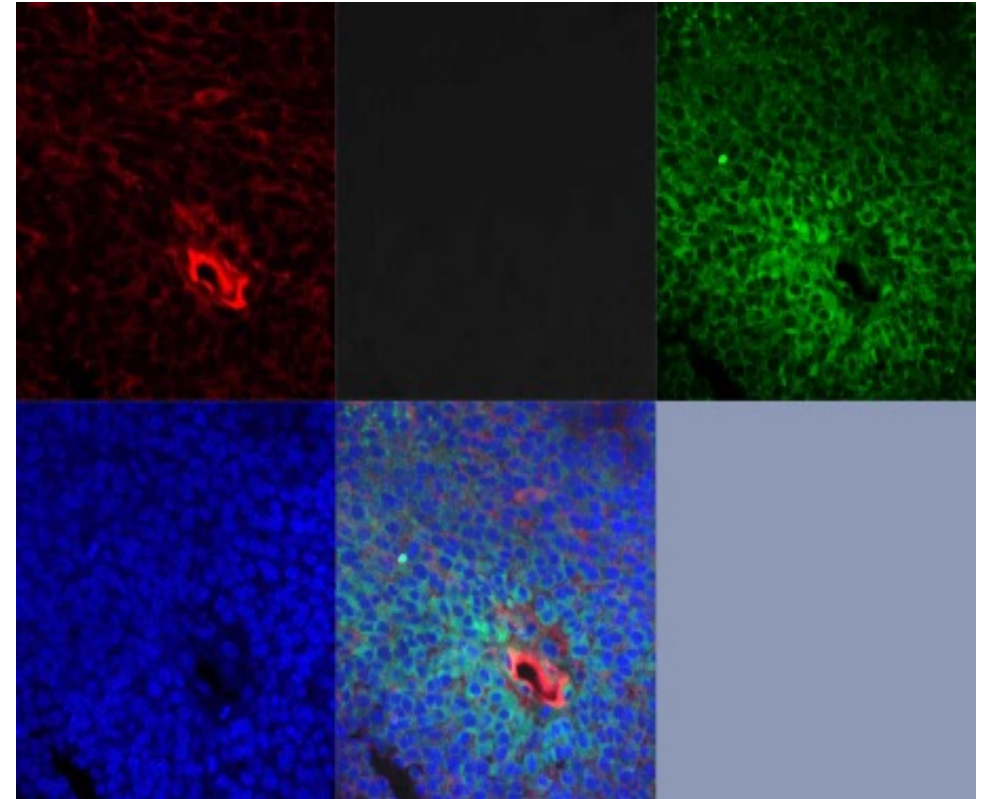

Supplement: Figure 7—figure supplement 4—source data 1. [file elife-78972-fig7-figsupp4-data1.zip › Figure 7-Figure Supplement 4-Source Data/Figure 7-Figure Supplement 4-Source Data G/G.pdf]

FMOD

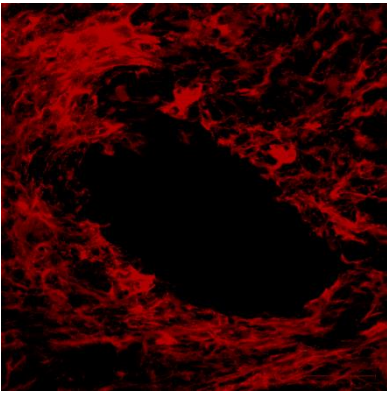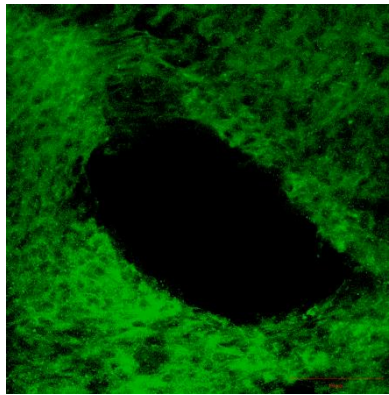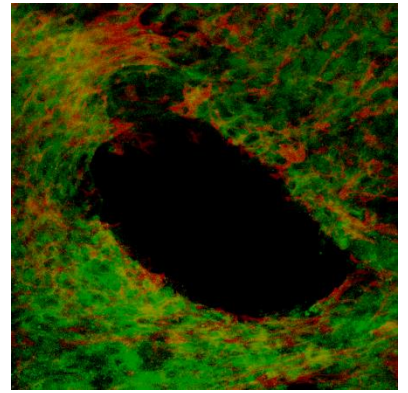

pFAK

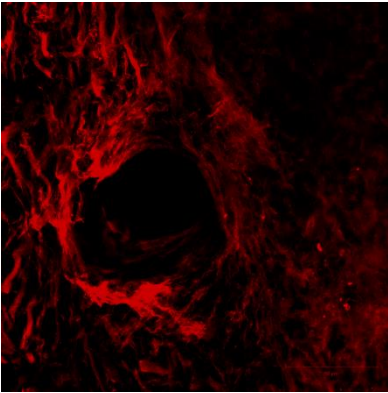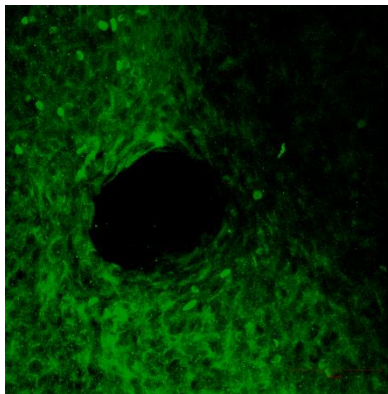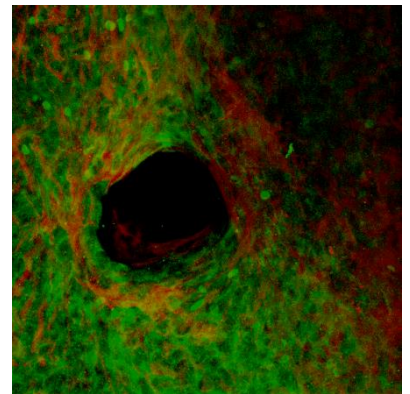

JAG1

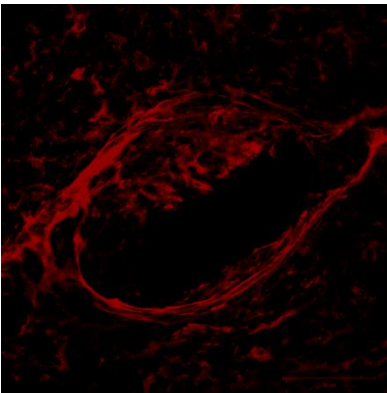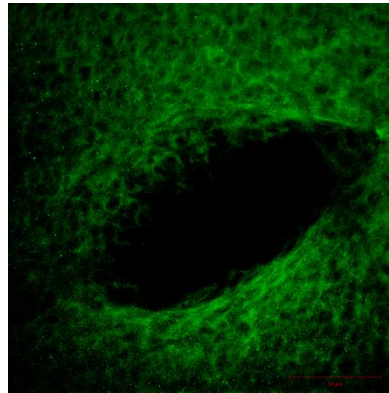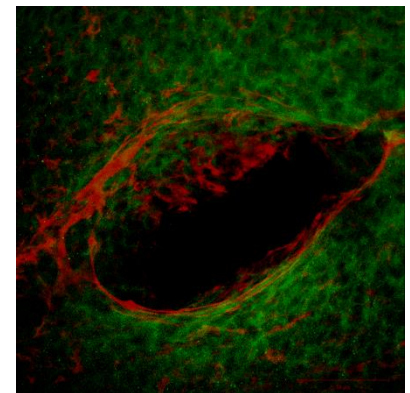

HES1

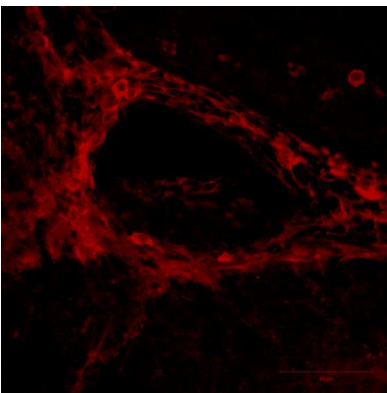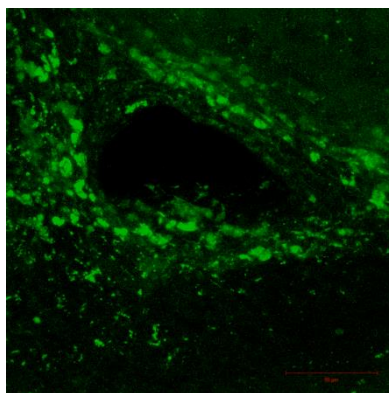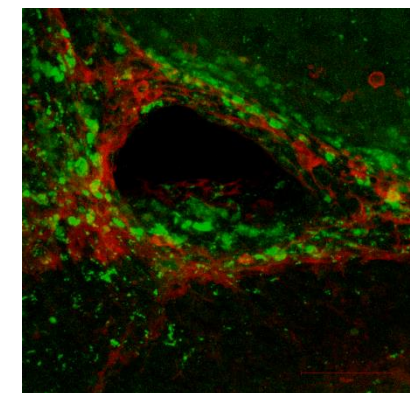

Supplement: Figure 7—figure supplement 5—source data 1. [file elife-78972-fig7-figsupp5-data1.zip › Figure 7-Figure Supplement 5-Source Data/IMAGES FOR PAANEL A.pdf]

FMOD

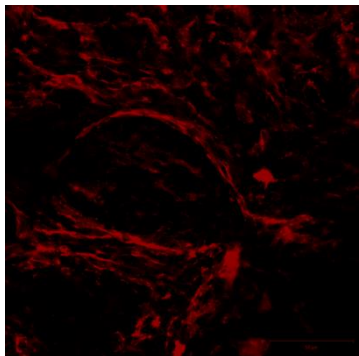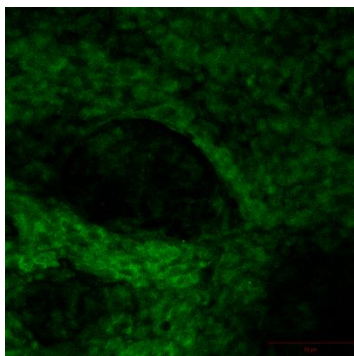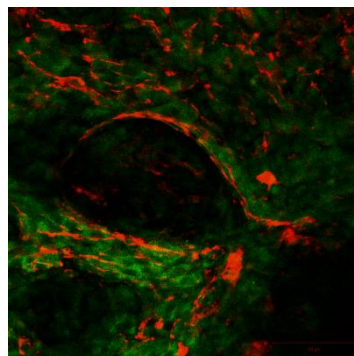

pFAK

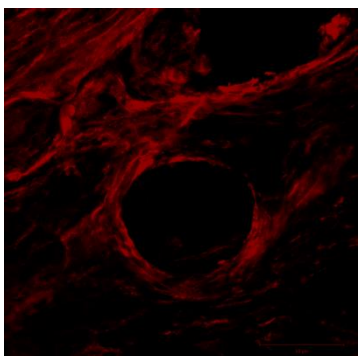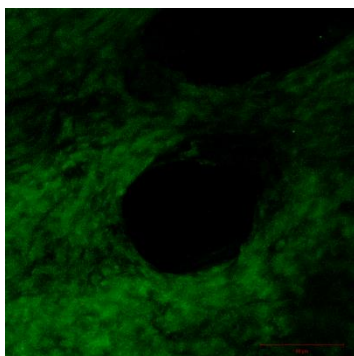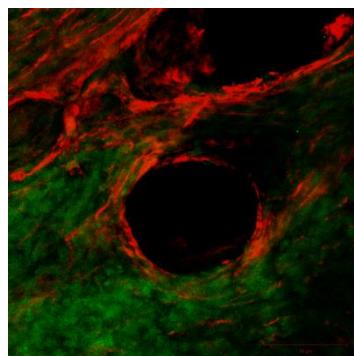

JAG1

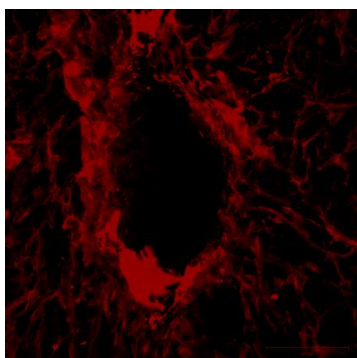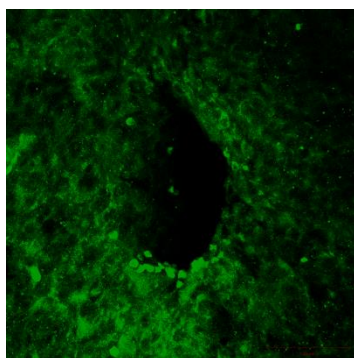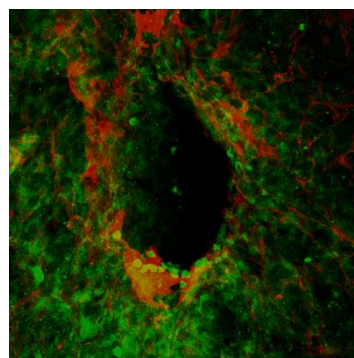

HES1

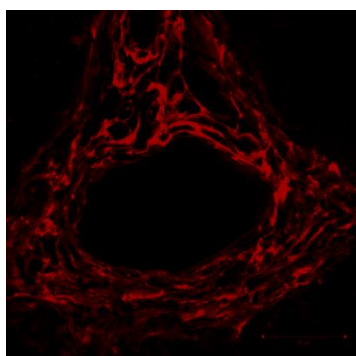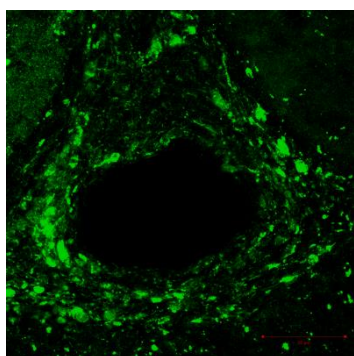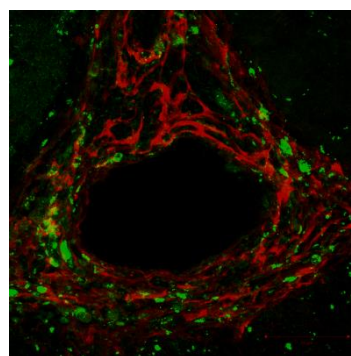

Supplement: Figure 7—figure supplement 5—source data 1. [file elife-78972-fig7-figsupp5-data1.zip › Figure 7-Figure Supplement 5-Source Data/IMAGES FOR PANEL B.pdf]

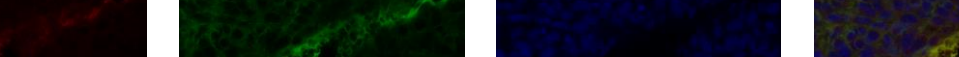

Supplement: Figure 7—figure supplement 5—source data 1. [file elife-78972-fig7-figsupp5-data1.zip › Figure 7-Figure Supplement 5-Source Data/IMAGES FOR PANEL C.pdf]
