# Supplementary material for: Differentiated glioma cell-derived fibromodulin activates integrin-dependent Notch signaling in endothelial cells to promote tumor angiogenesis and growth
Source: eLife. 2022 Jun 1;11:e78972. doi: 10.7554/eLife.78972 (PMC9259034; doi:10.7554/eLife.78972)
Supplement: Figure 2—figure supplement 7—source data 1. [file elife-78972-fig2-figsupp7-data1.zip › Figure 2-Figure Supplement 7-Source Data/BLOTS.pdf]

Supplementary Figure 12

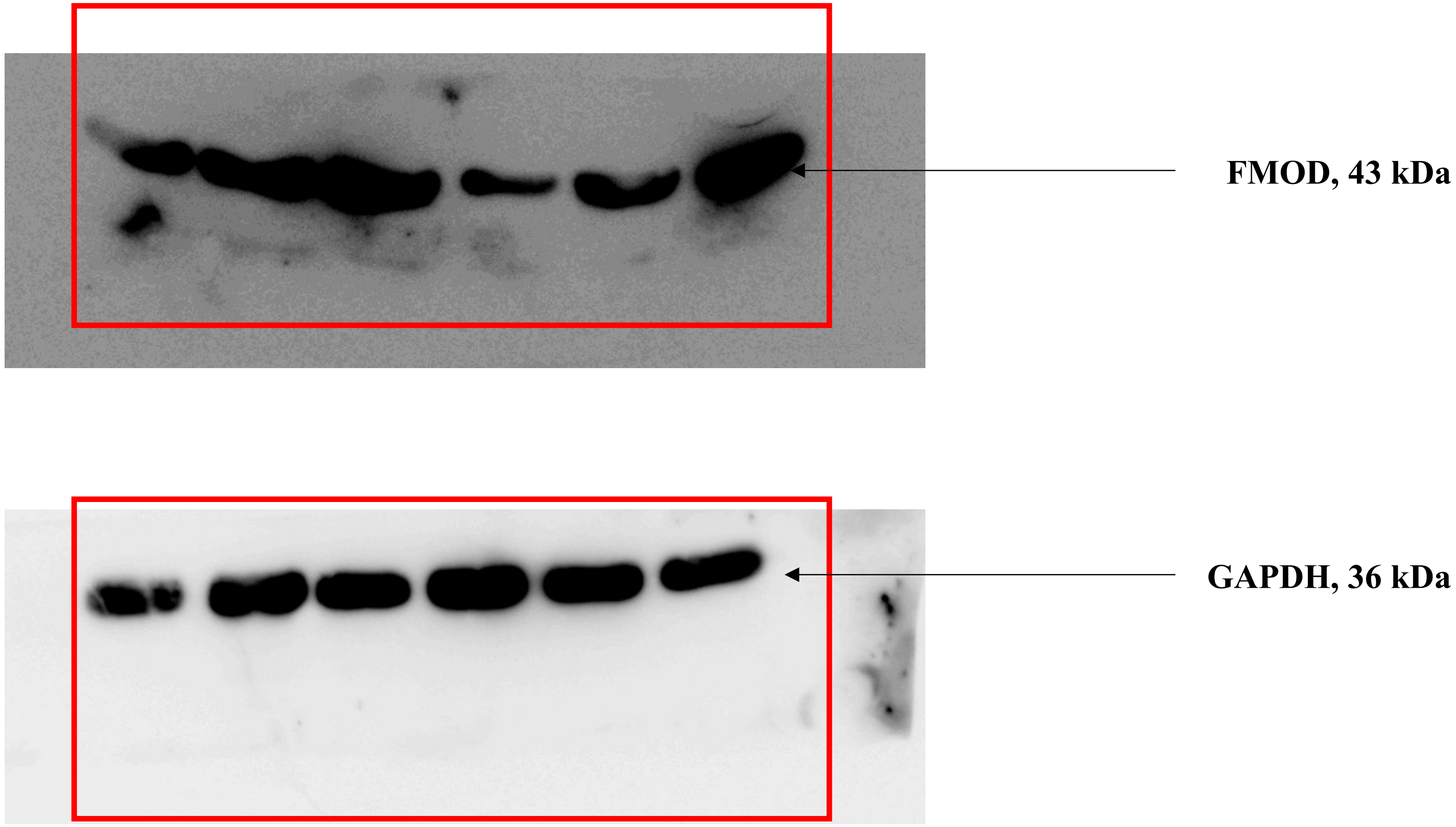

A separate gel was run with less amount of total protein for GAPDH western blot
