# Supplementary material for: Differentiated glioma cell-derived fibromodulin activates integrin-dependent Notch signaling in endothelial cells to promote tumor angiogenesis and growth
Source: eLife. 2022 Jun 1;11:e78972. doi: 10.7554/eLife.78972 (PMC9259034; doi:10.7554/eLife.78972)

Figure 3

3H

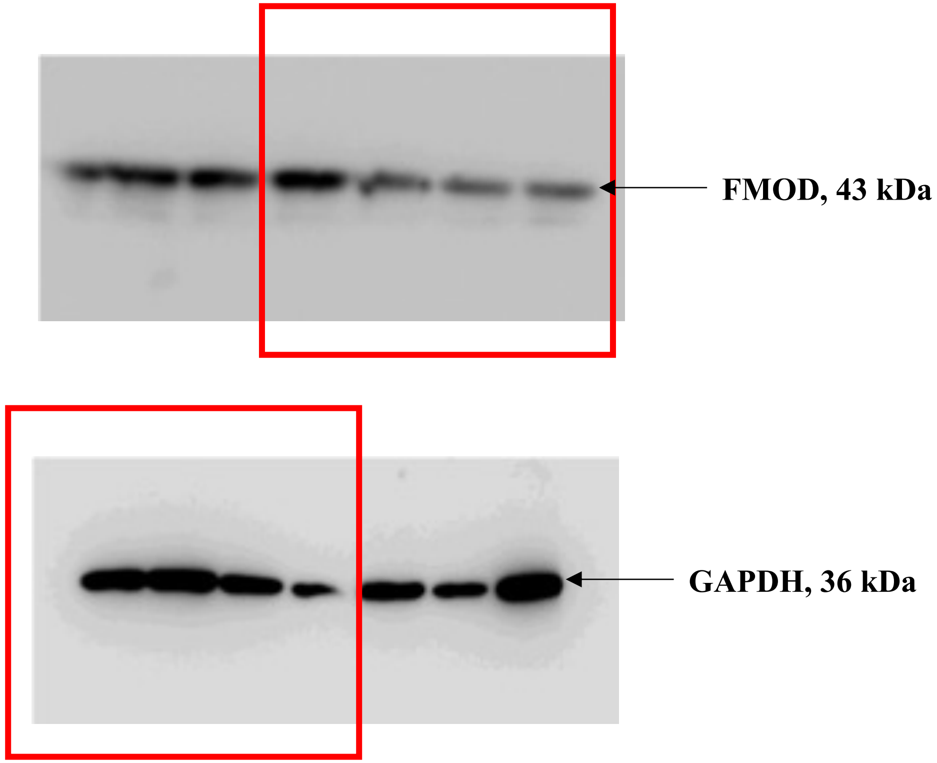

This blot got flipped while developing, the correct order has been put in the main text file, while the raw image has the original version.

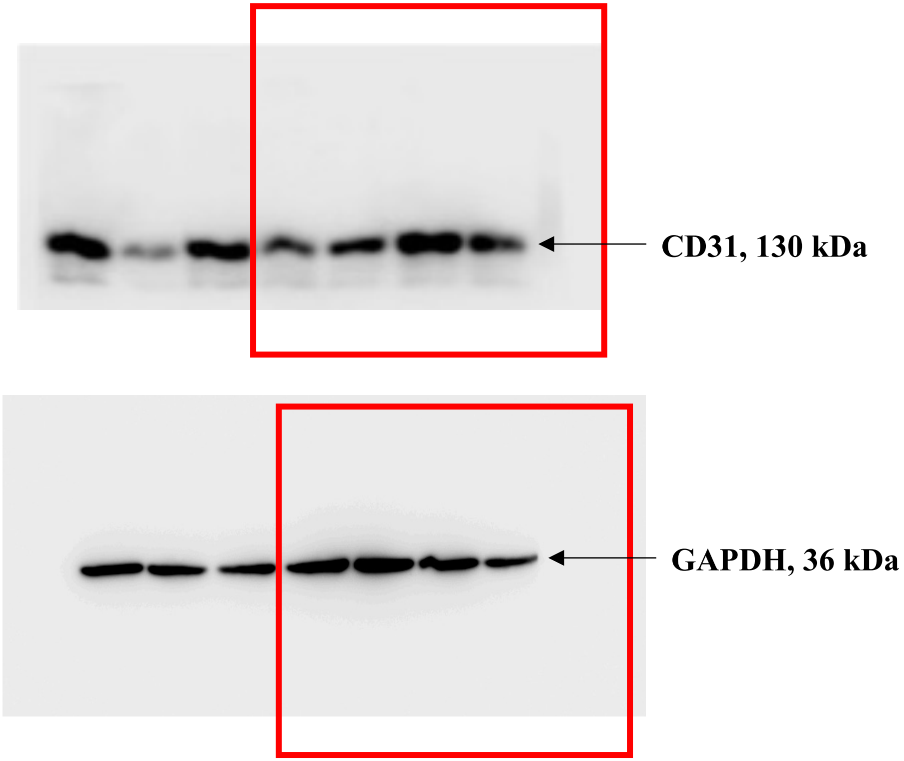

Supplement: Figure 3—source data 8. [file elife-78972-fig3-data8.zip › Figure 3-Source data H/BLOTS FOR PANEL H.pdf]
