# Supplementary material for: Differentiated glioma cell-derived fibromodulin activates integrin-dependent Notch signaling in endothelial cells to promote tumor angiogenesis and growth
Source: eLife. 2022 Jun 1;11:e78972. doi: 10.7554/eLife.78972 (PMC9259034; doi:10.7554/eLife.78972)
Supplement: Figure 4—figure supplement 3—source data 1. [file elife-78972-fig4-figsupp3-data1.zip › Figure 4-Figure Supplement 3-Source Data/Figure 4-Figure Supplement 3-Source Data D,E,F,G,H,I/BLOTS FOR PANELS D,E,F,G,H,I.pdf]

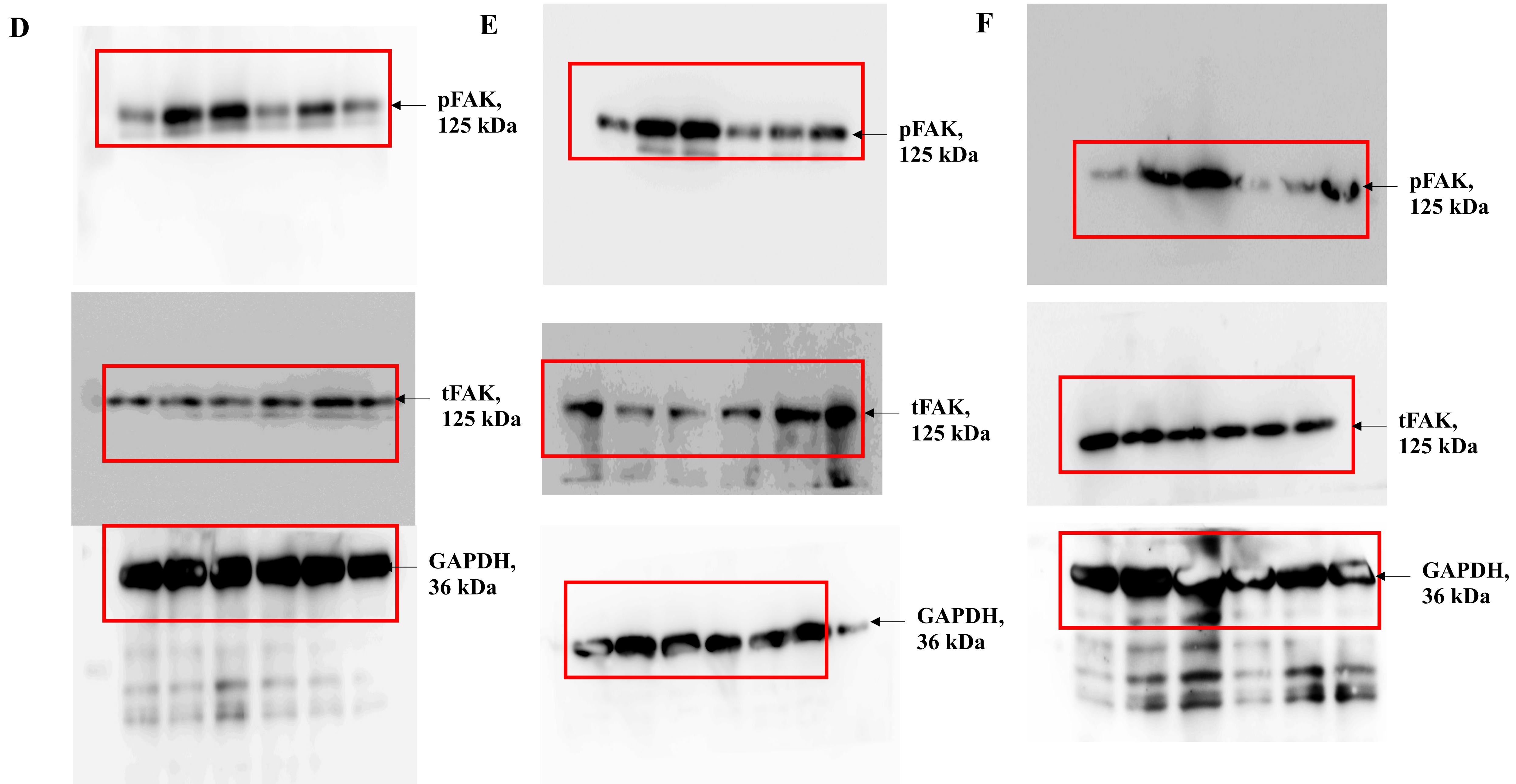
D
pFAK, 125 kDa
tFAK, 125 kDa
GAPDH, 36 kDa
E
pFAK, 125 kDa
tFAK, 125 kDa
GAPDH, 36 kDa
F
pFAK, 125 kDa
tFAK, 125 kDa
GAPDH, 36 kDa

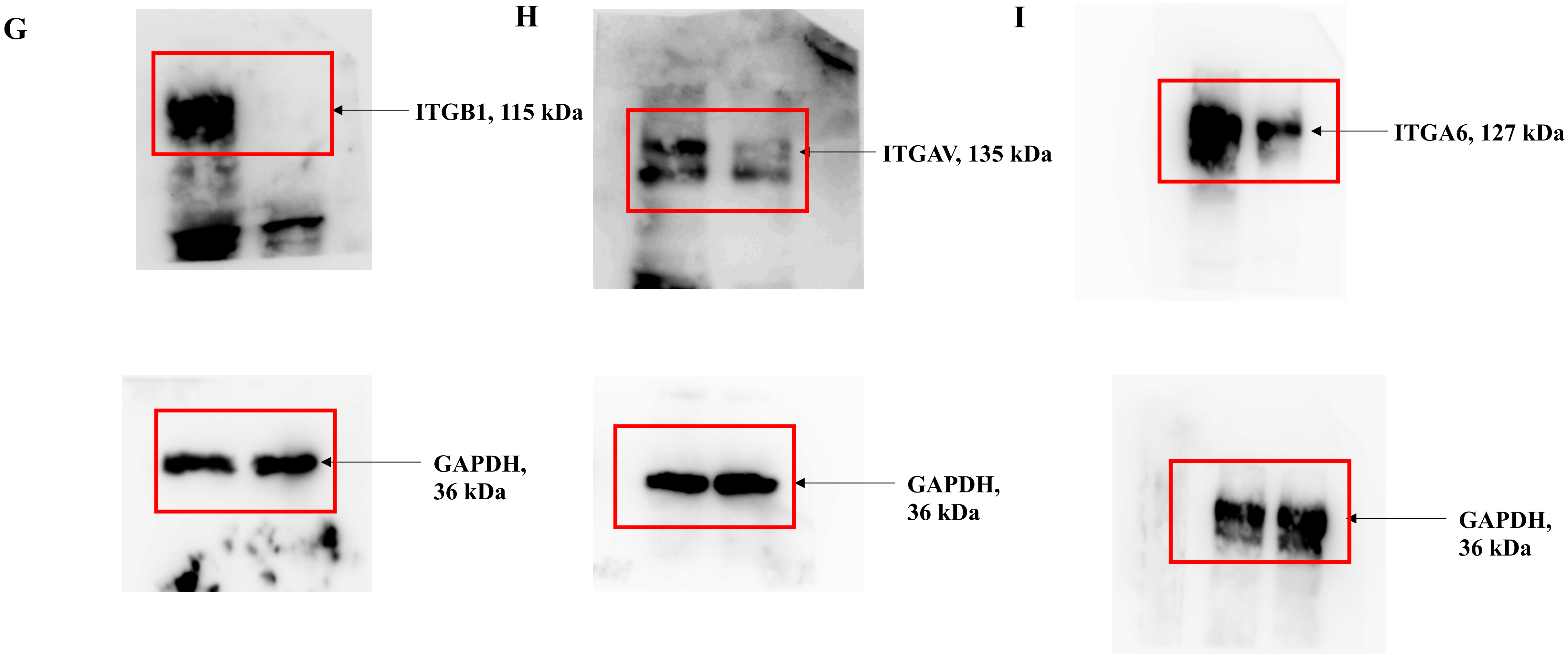
G
ITGB1, 115 kDa
GAPDH, 36 kDa
H
ITGAV, 135 kDa
GAPDH, 36 kDa
I
ITGA6, 127 kDa
GAPDH, 36 kDa
