# Supplementary material for: Differentiated glioma cell-derived fibromodulin activates integrin-dependent Notch signaling in endothelial cells to promote tumor angiogenesis and growth
Source: eLife. 2022 Jun 1;11:e78972. doi: 10.7554/eLife.78972 (PMC9259034; doi:10.7554/eLife.78972)

Figure 1

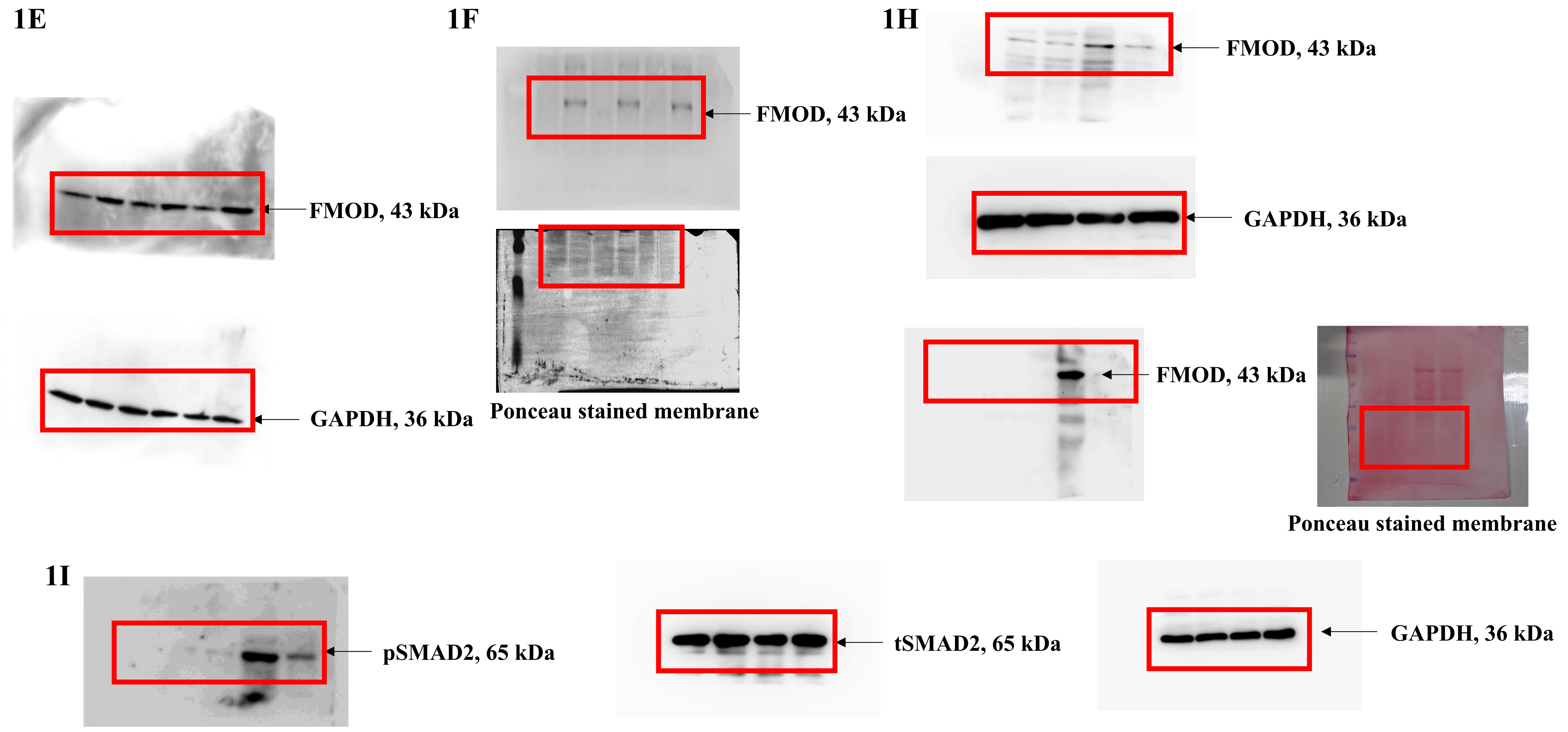

The red box indicates that the marked lanes are from the relevant experiment that has been shown in the manuscript and shown in the figure

Figure 3

3H

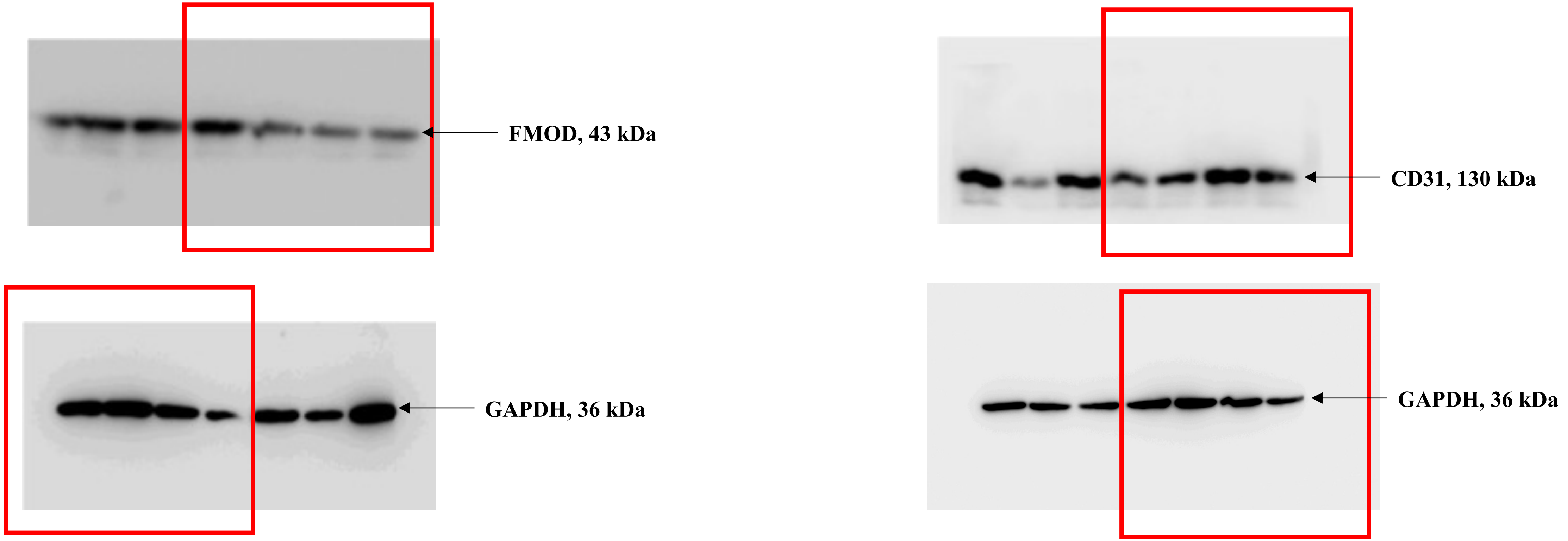

This blot got flipped while developing, the correct order has been put in the main text file, while the raw image has the original version.

Figure 4

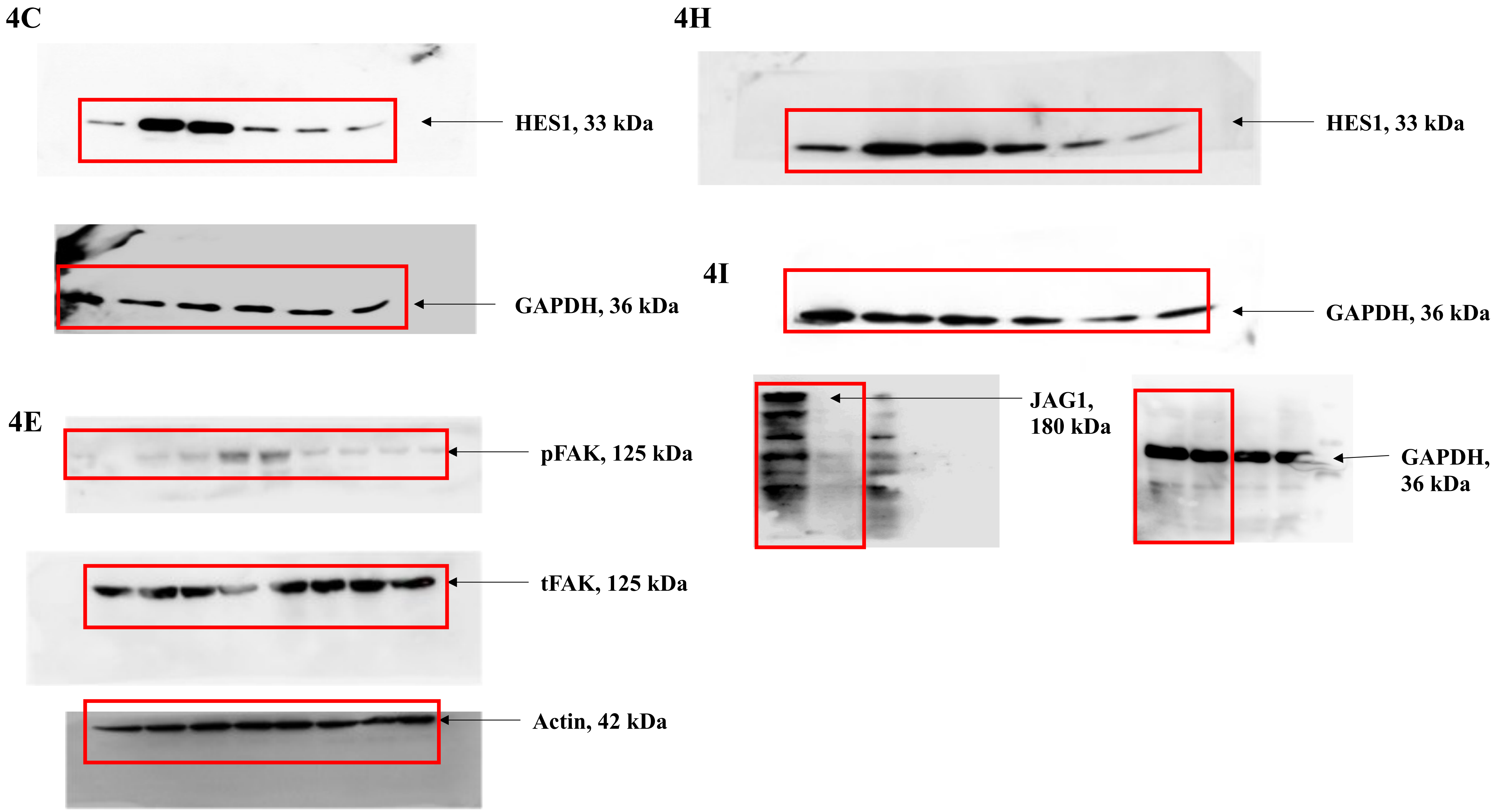
4C
HES1, 33 kDa
GAPDH, 36 kDa
4E
pFAK, 125 kDa
tFAK, 125 kDa
Actin, 42 kDa
4H
HES1, 33 kDa
4I
GAPDH, 36 kDa
JAG1, 180 kDa
GAPDH, 36 kDa

Figure 4

4K

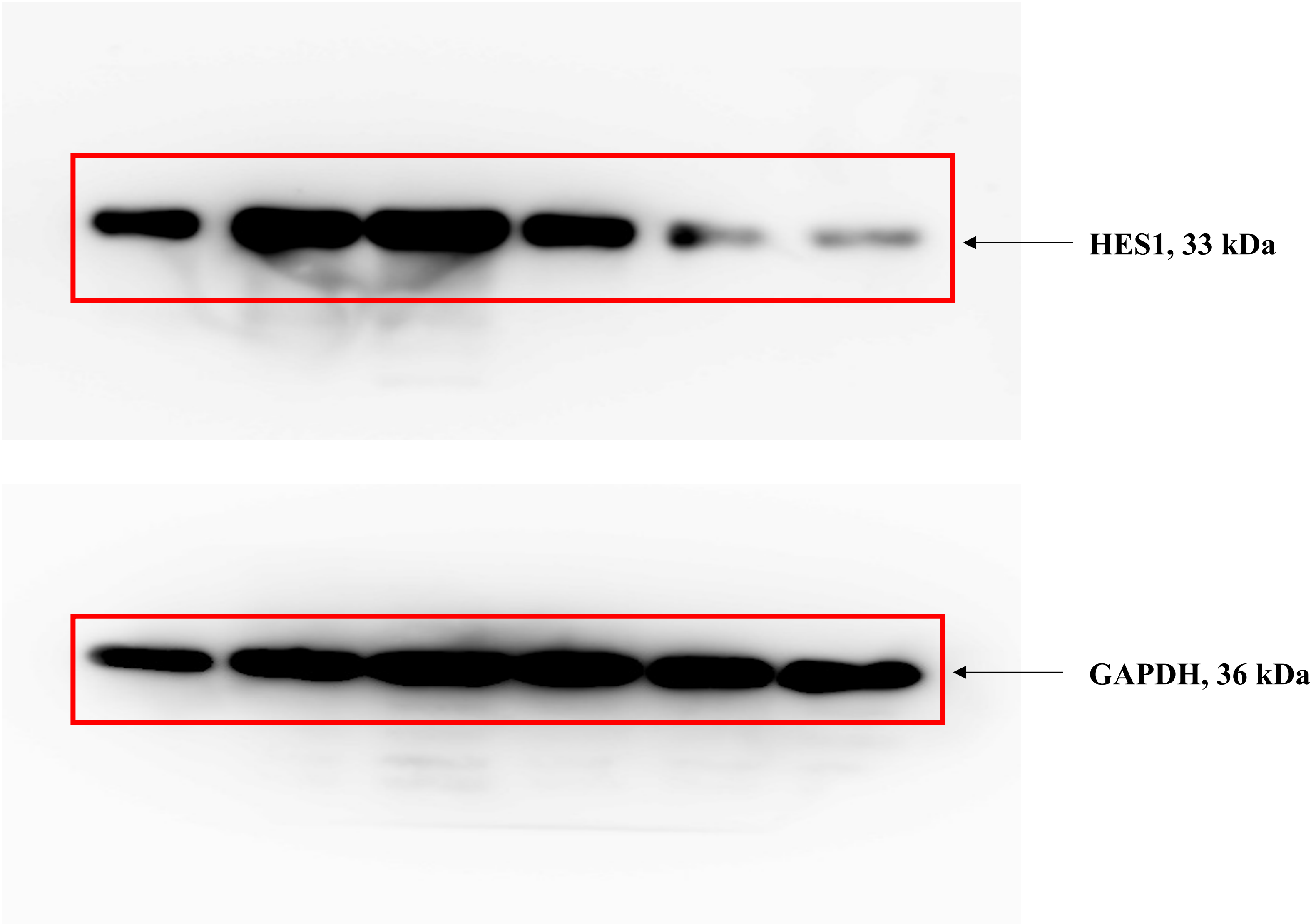

Supplementary Figure 6

6A

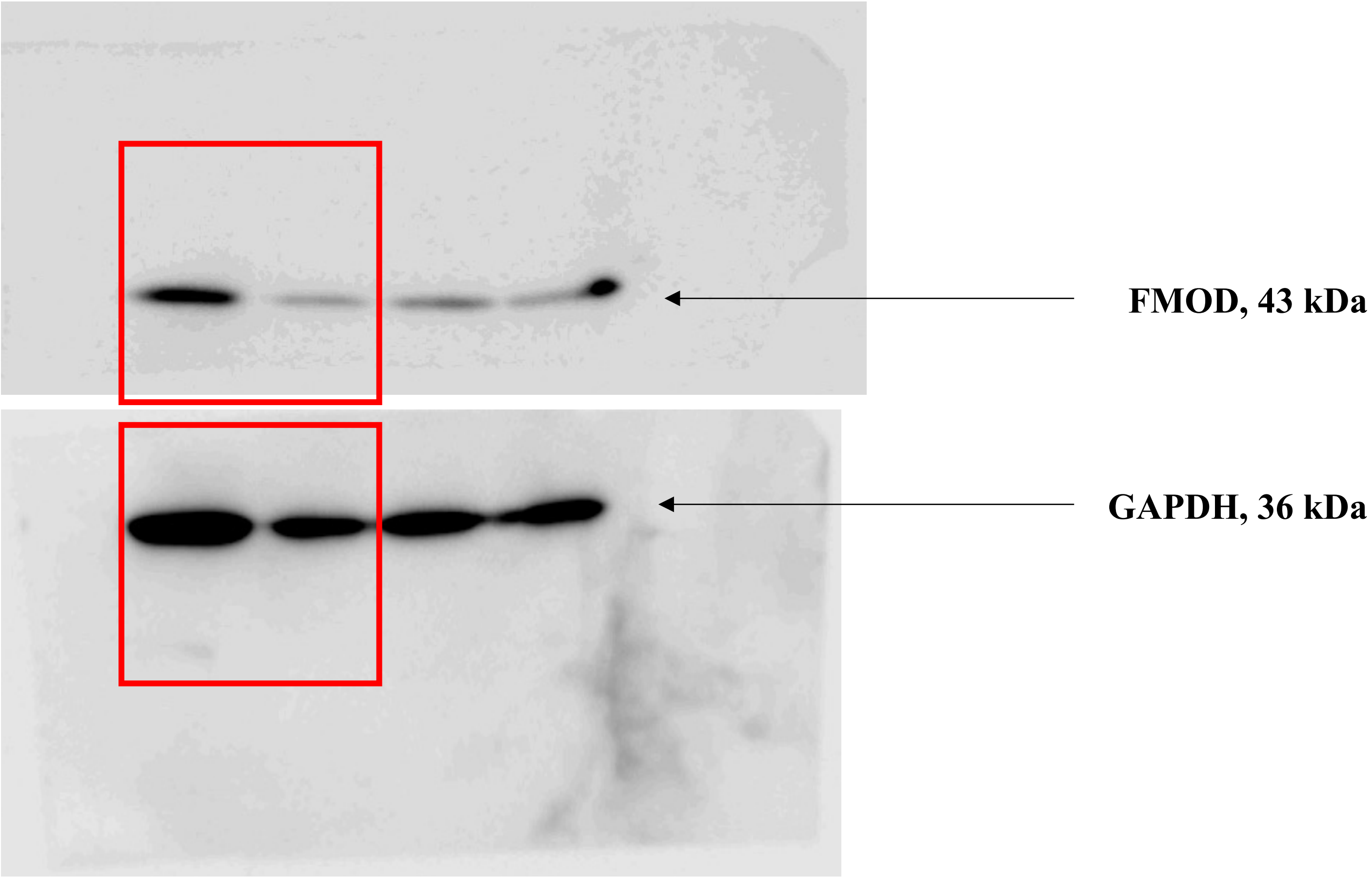

Supplementary Figure 7

7B

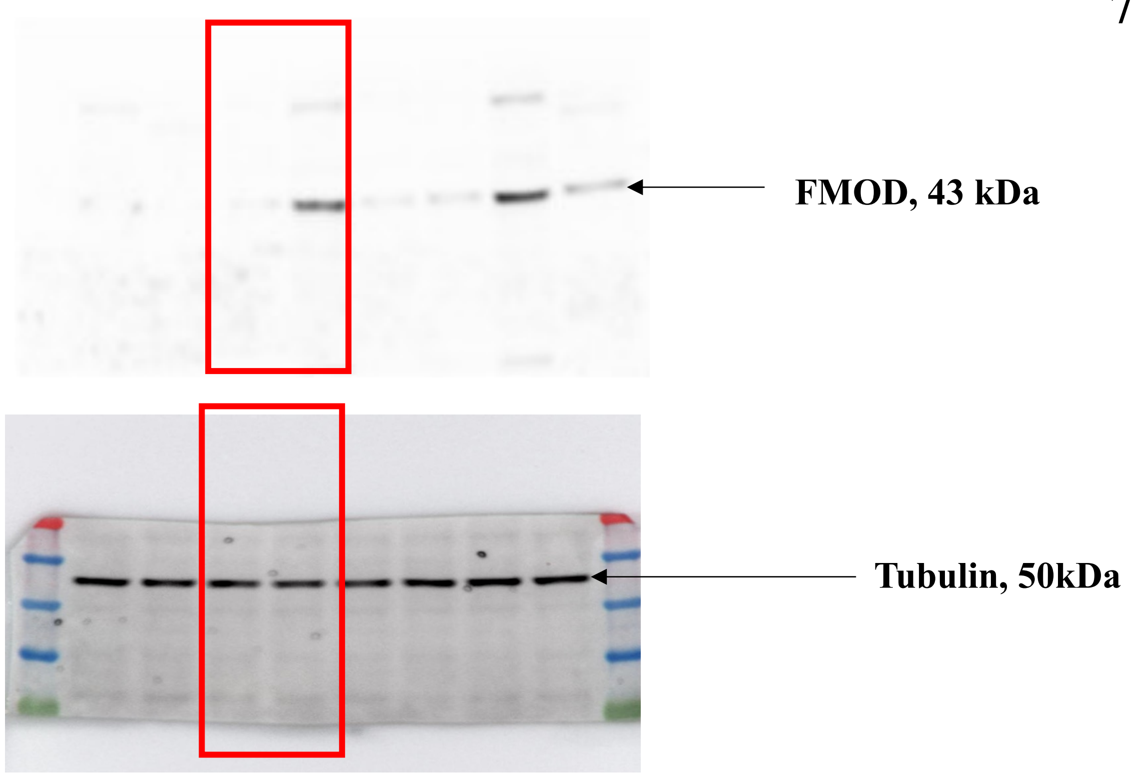

7E

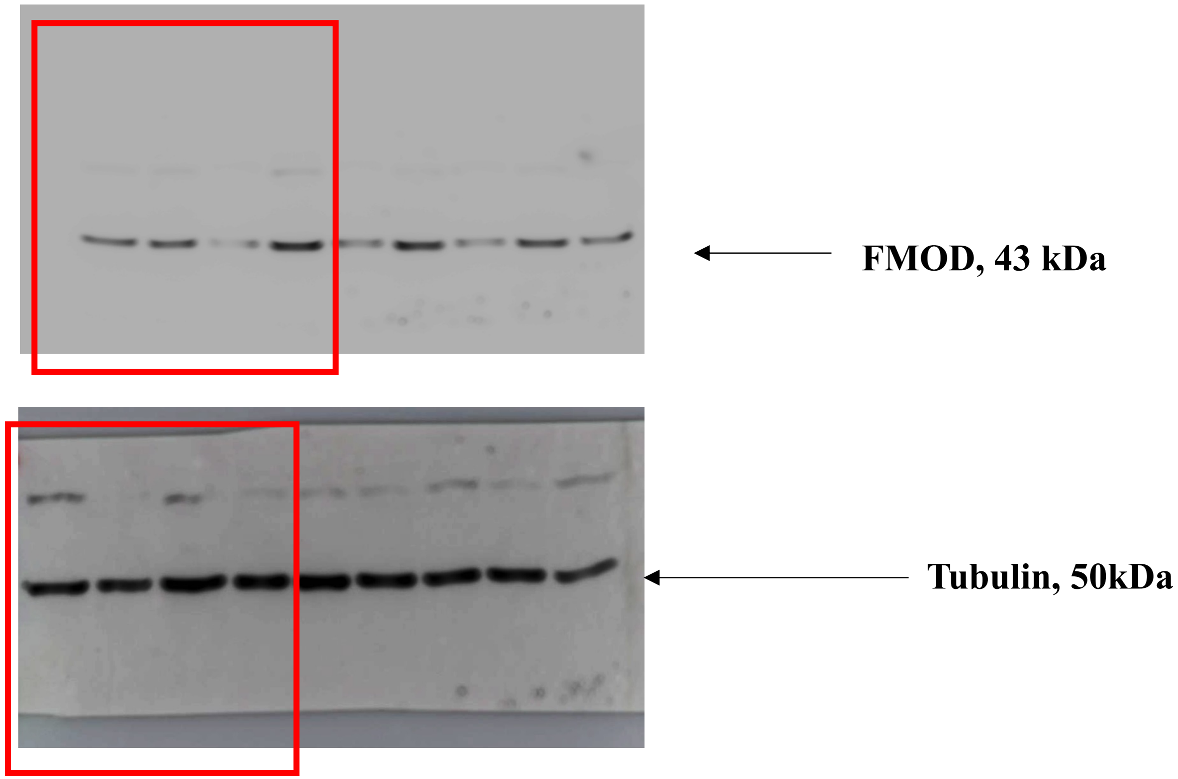

Supplementary Figure 7

7C

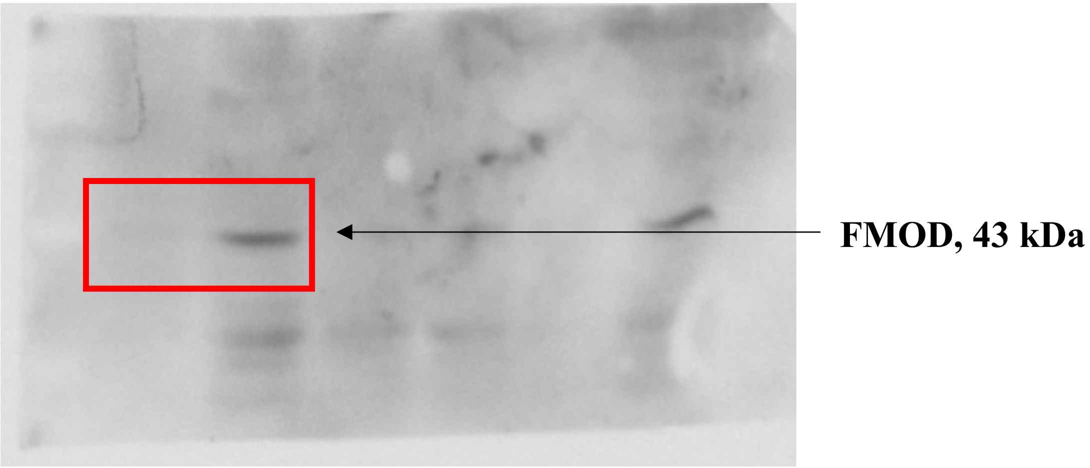

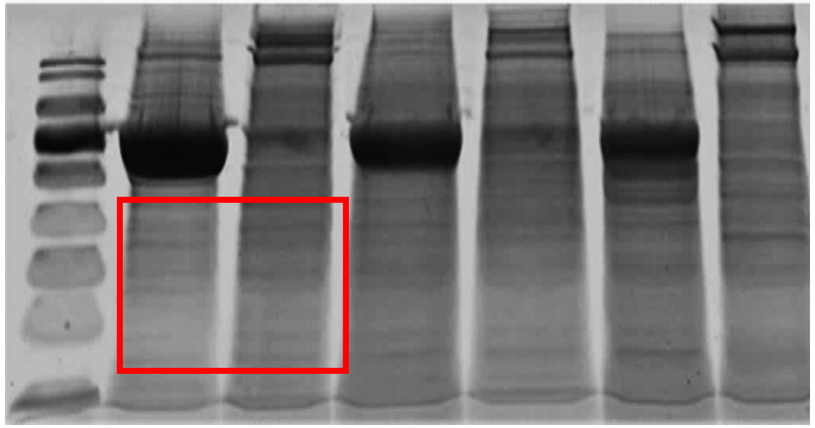

Ponceau stained membrane

Supplementary Figure 10

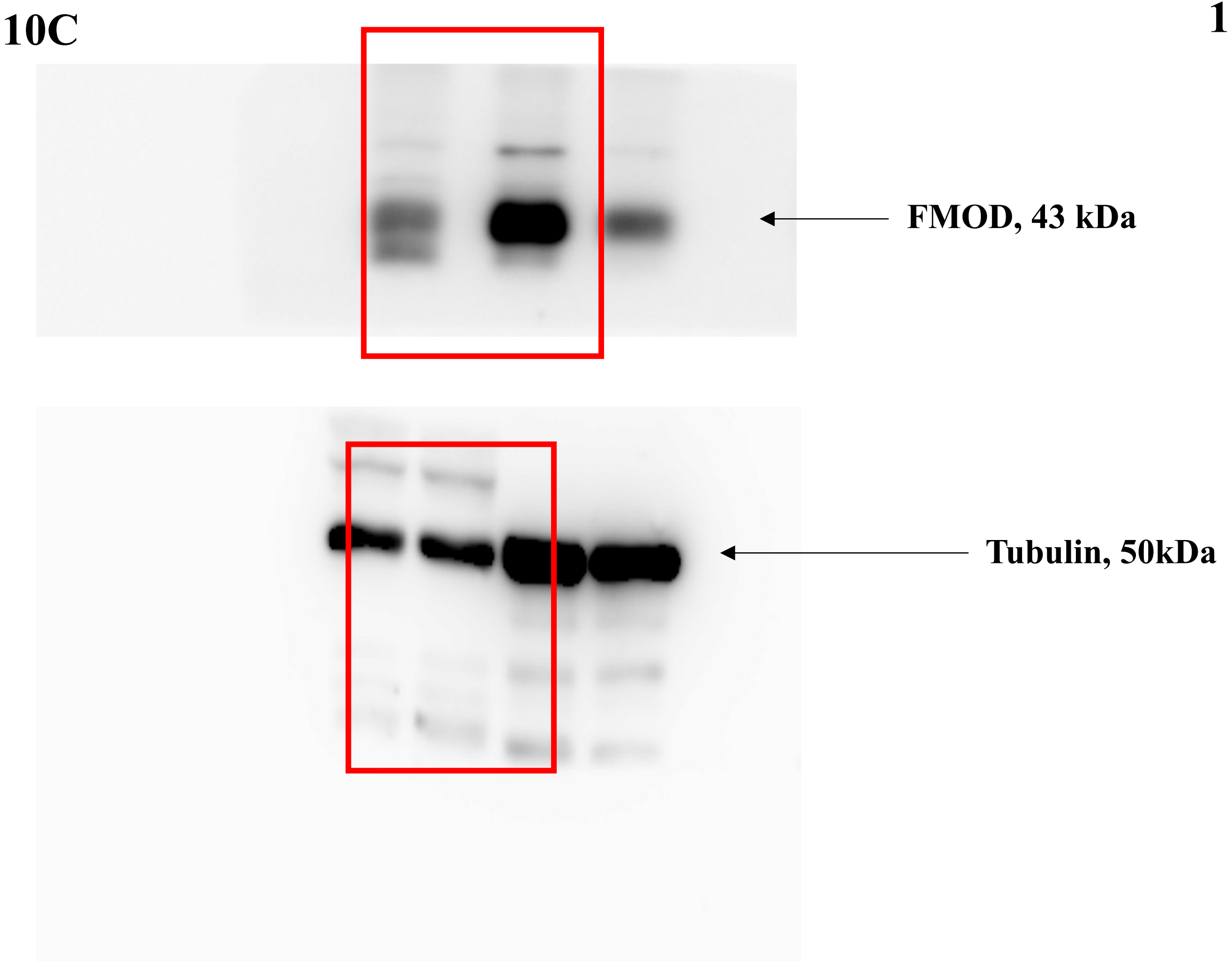

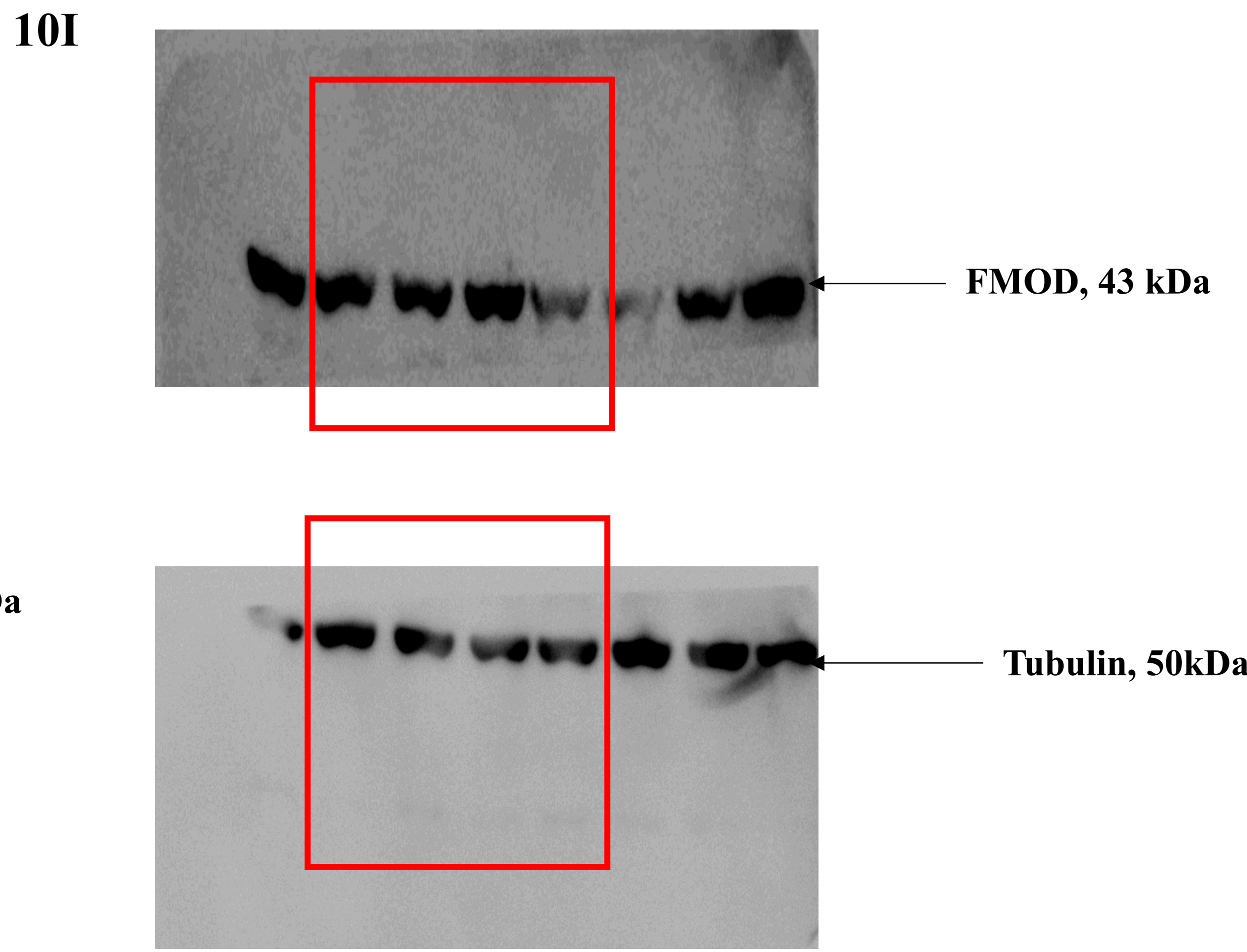

Supplementary Figure 11

Gel pic

11A

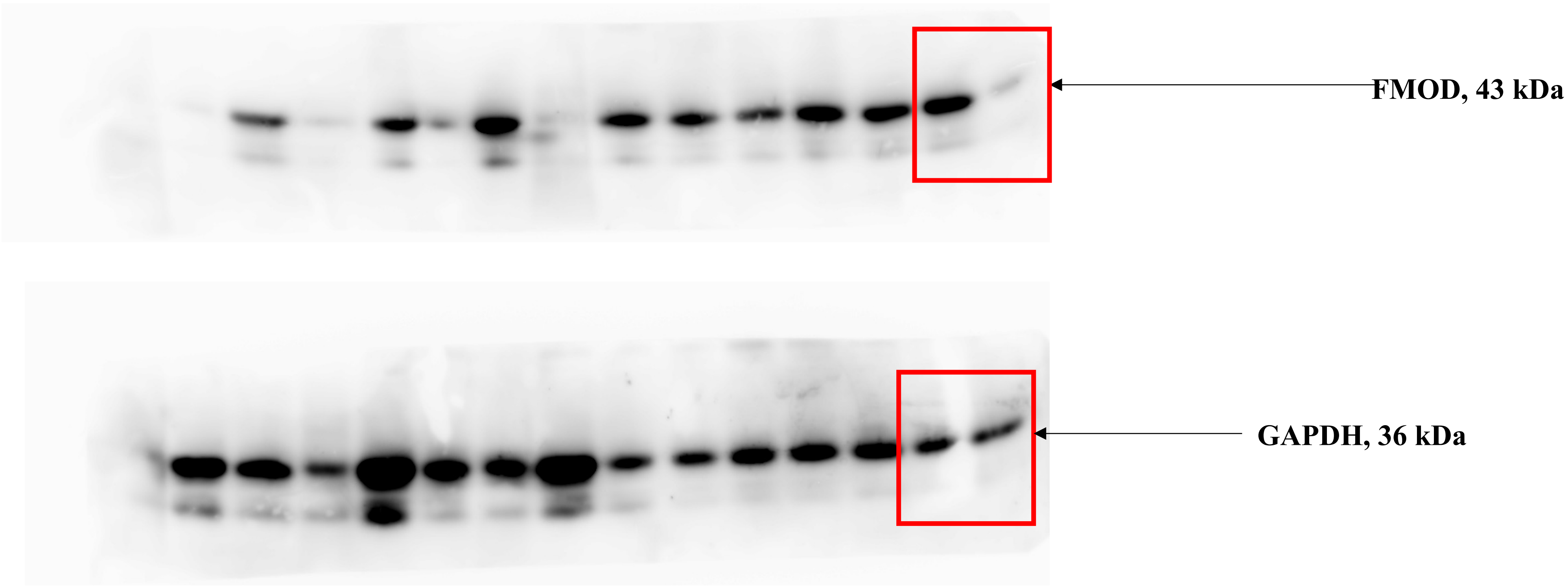

Supplementary Figure 12

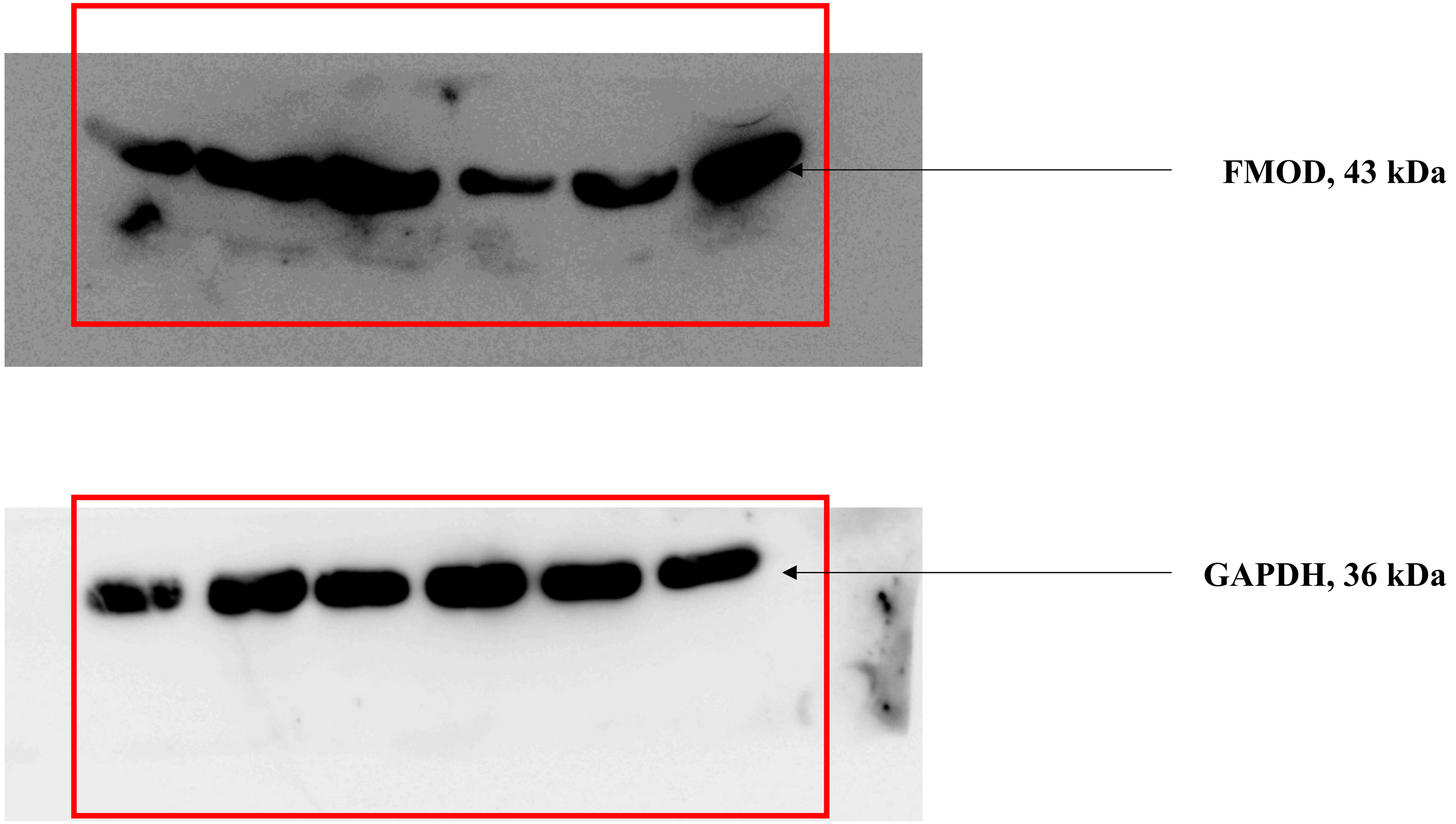

A separate gel was run with less amount of total protein for GAPDH western blot

Supplementary Figure 15

15B

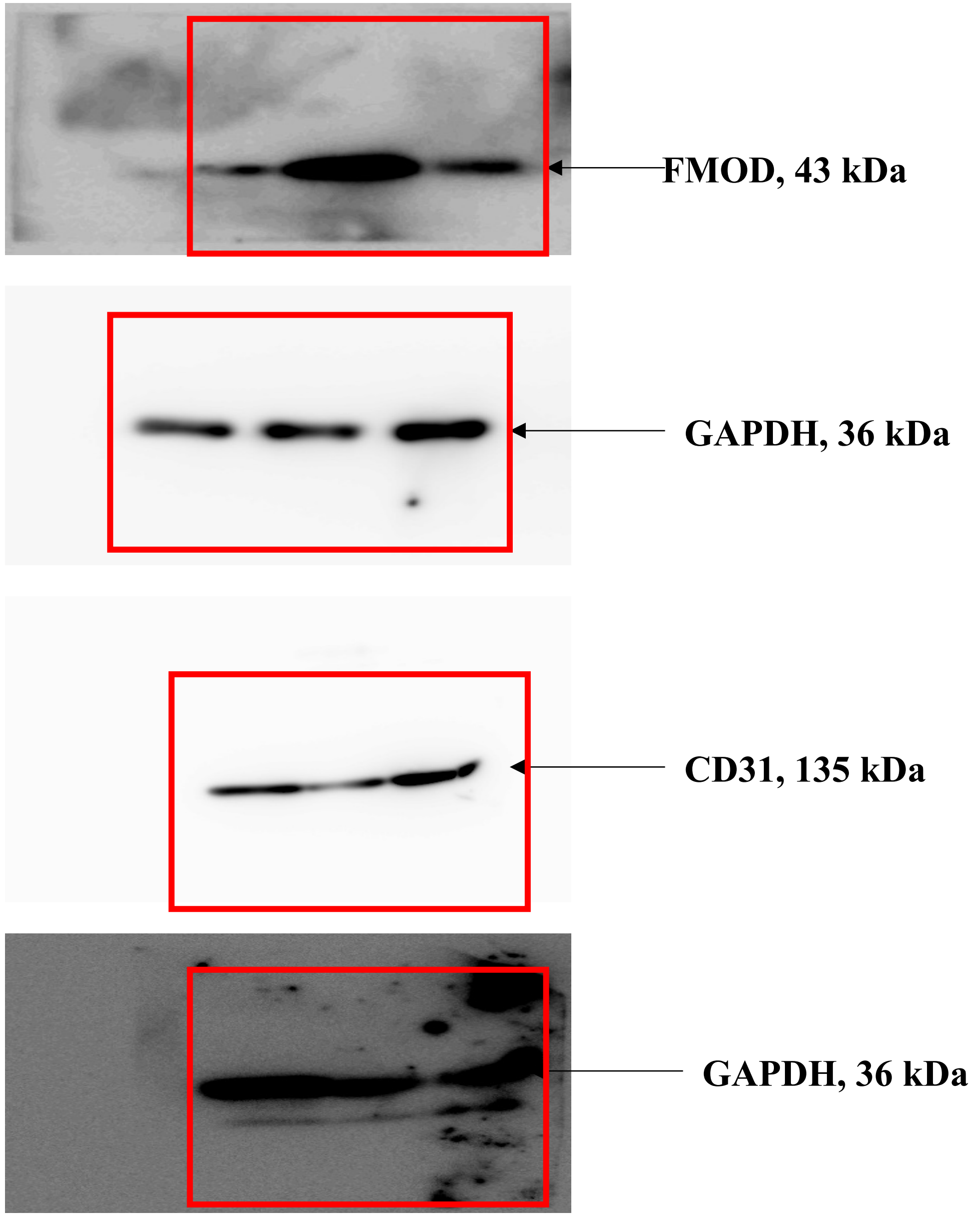

Supplementary Figure 16

16 C

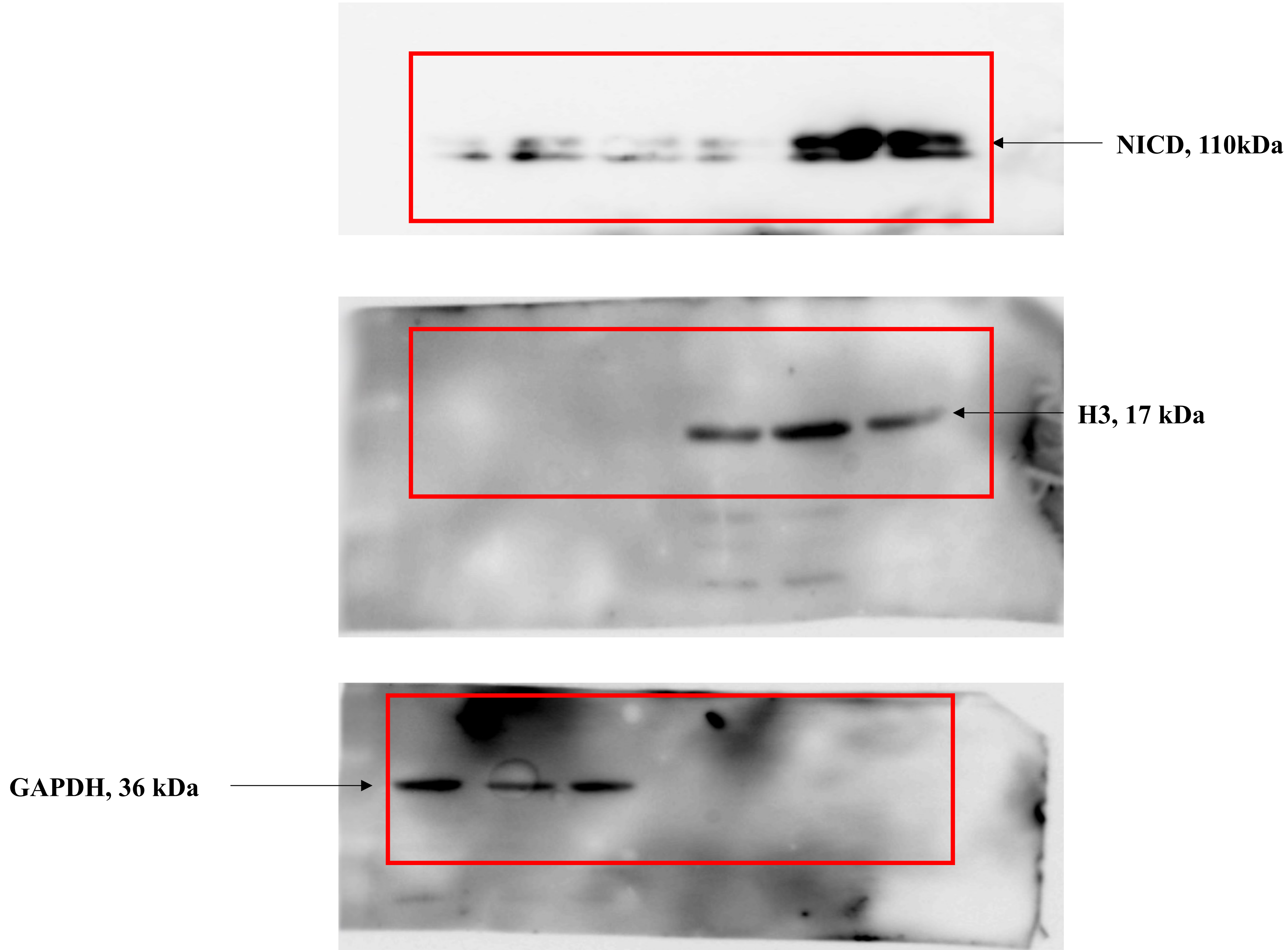

Supplementary Figure 17

17A

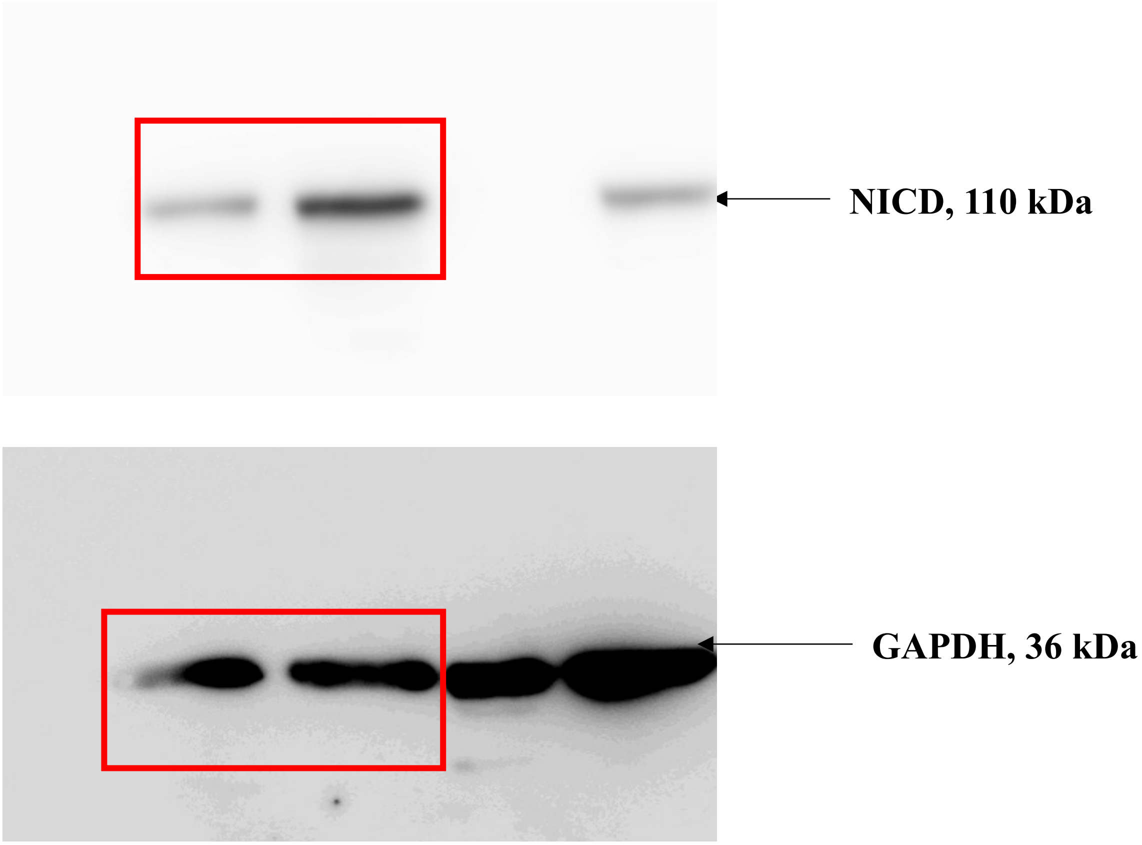

Supplementary Figure 18

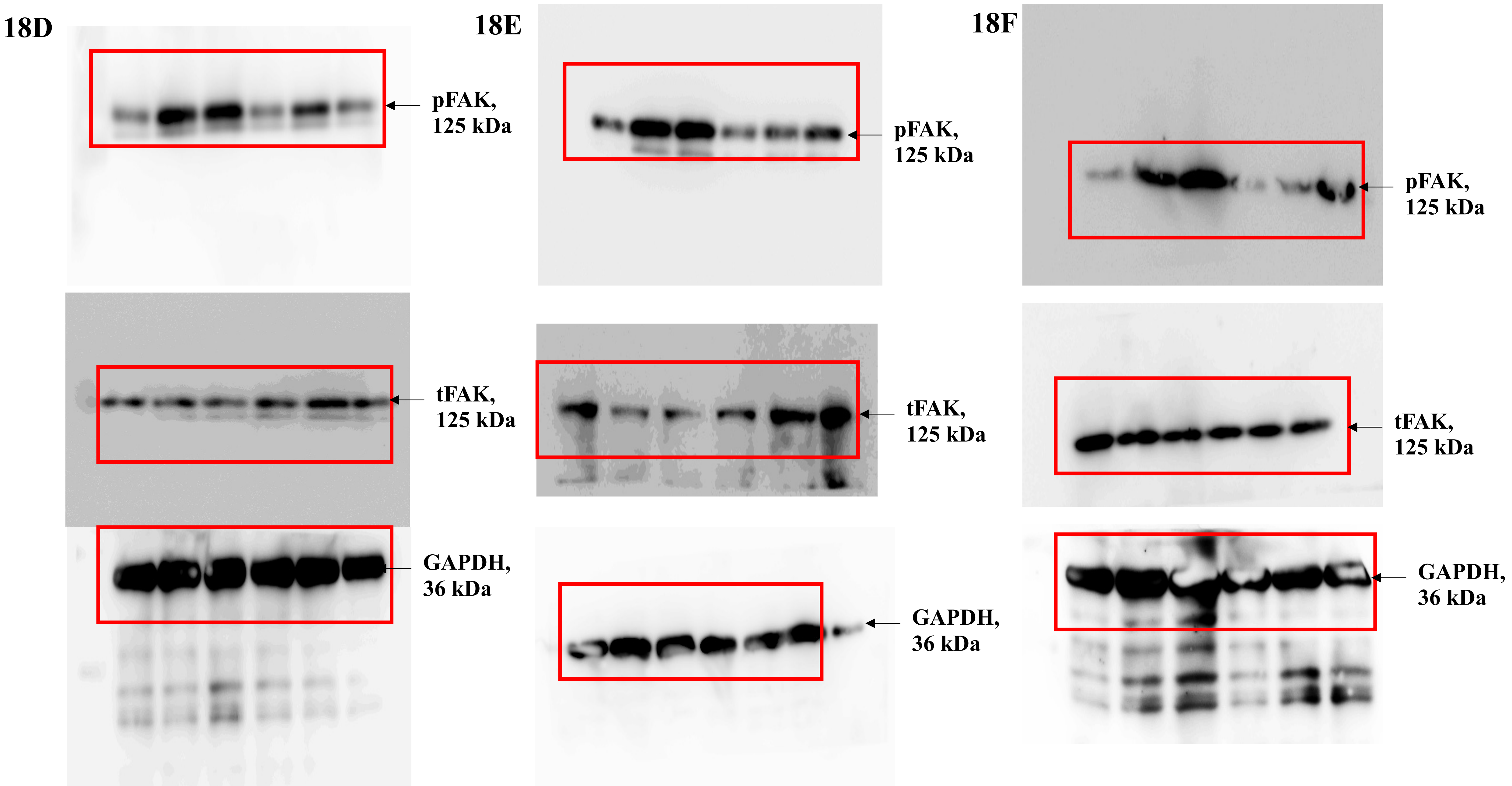

Supplementary Figure 18

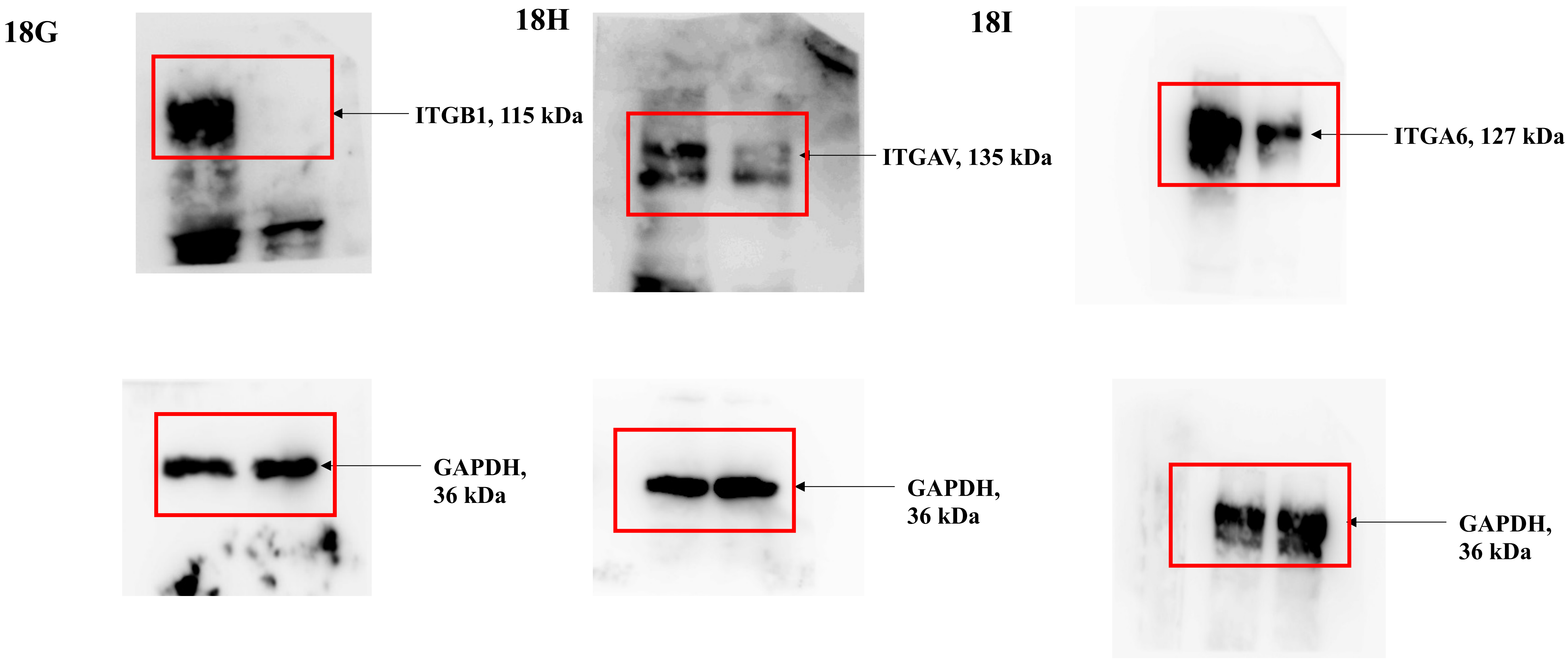

Supplementary Figure 19

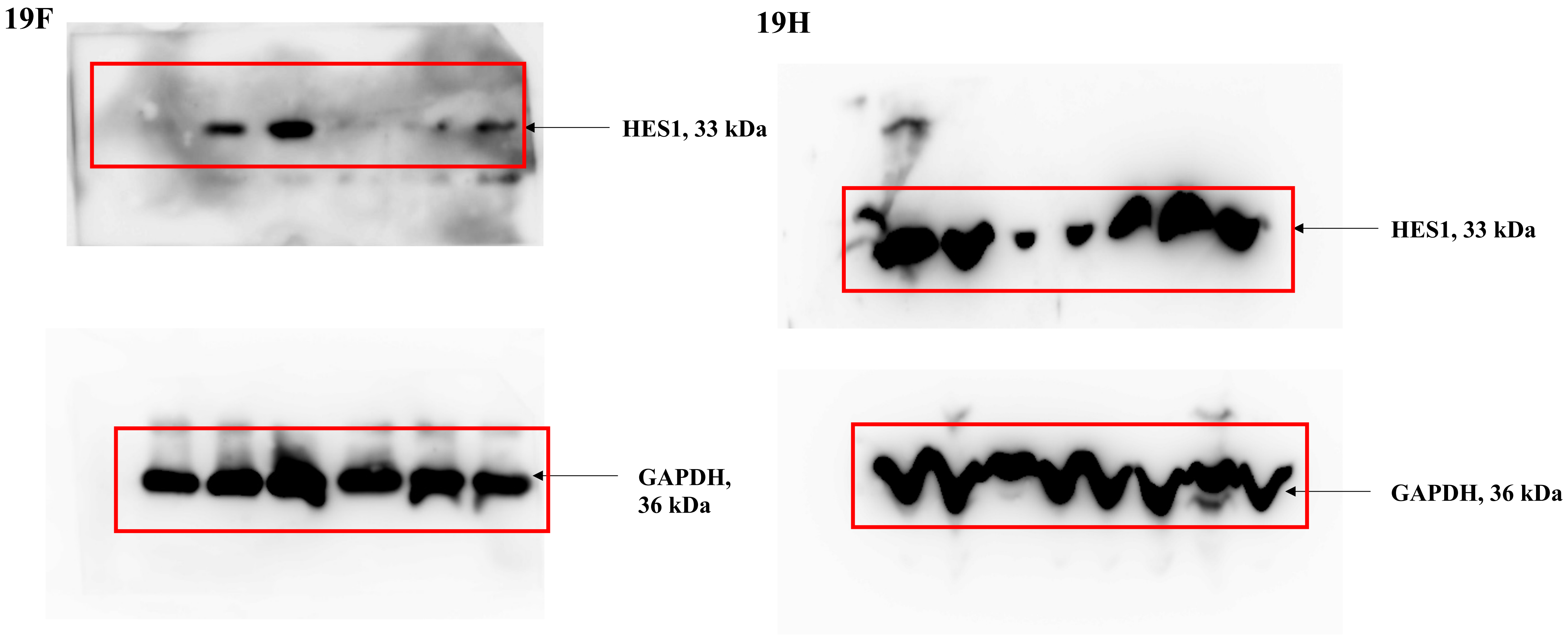

G

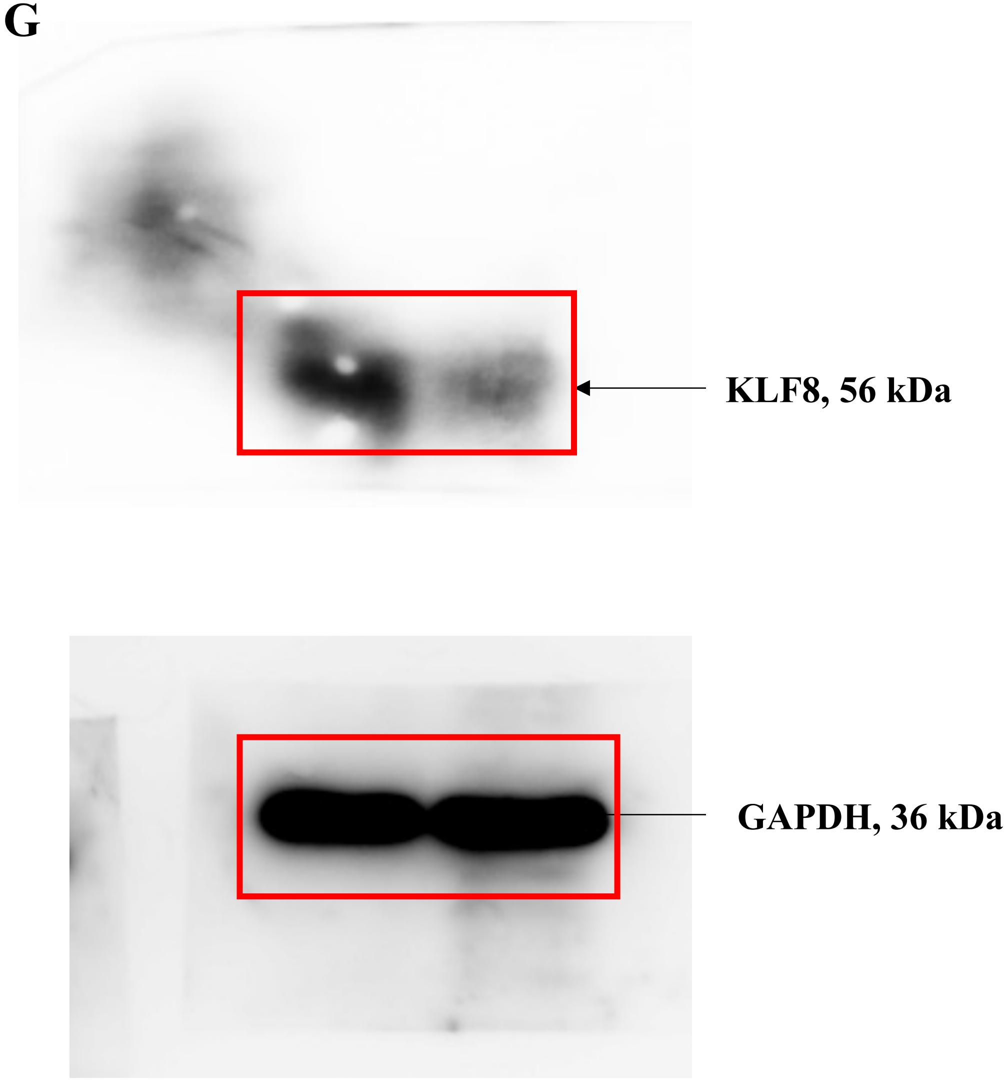

H

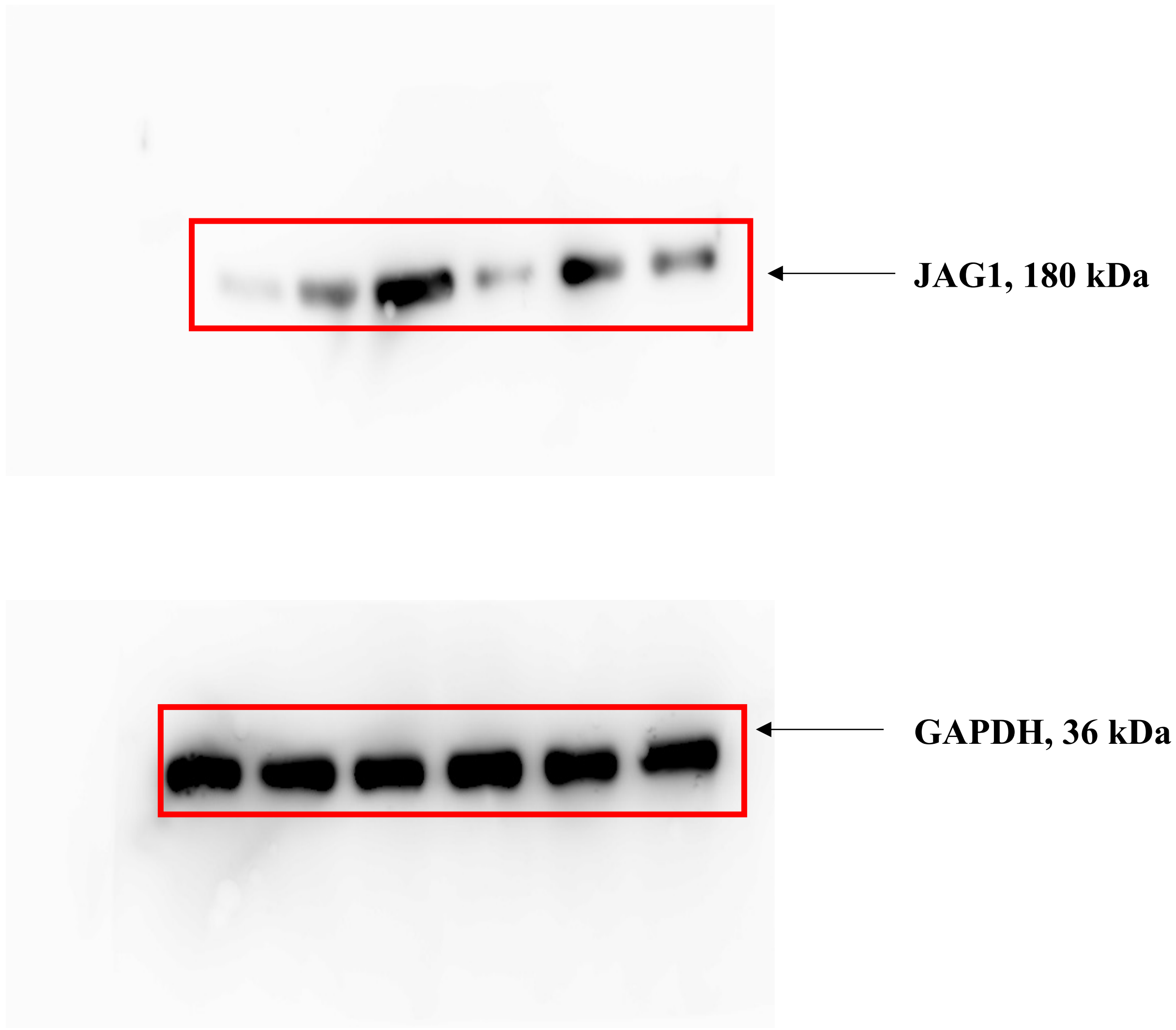

Supplement: Figure 4—figure supplement 5—source data 1. [file elife-78972-fig4-figsupp5-data1.zip › Figure 4-Figure Supplement 5-Source Data/Figure 4-Figure Supplement 5-Source Data G,H/RAW blots-Figures.pdf]
