# Supplementary material for: Differentiated glioma cell-derived fibromodulin activates integrin-dependent Notch signaling in endothelial cells to promote tumor angiogenesis and growth
Source: eLife. 2022 Jun 1;11:e78972. doi: 10.7554/eLife.78972 (PMC9259034; doi:10.7554/eLife.78972)

Figure 1

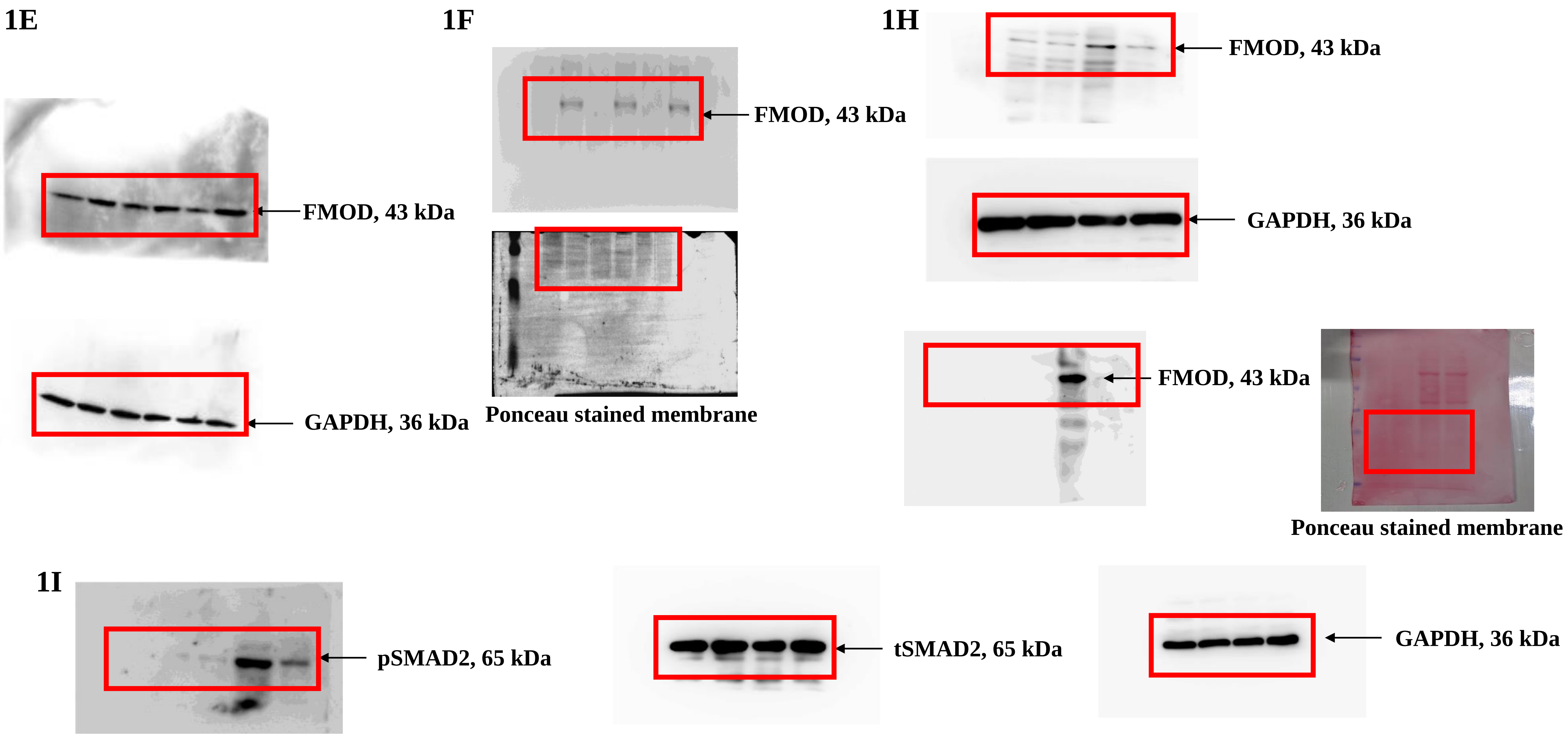

The red box indicates that the marked lanes are from the relevant experiment that has been shown in the manuscript and shown in the figure

Figure 3

3H

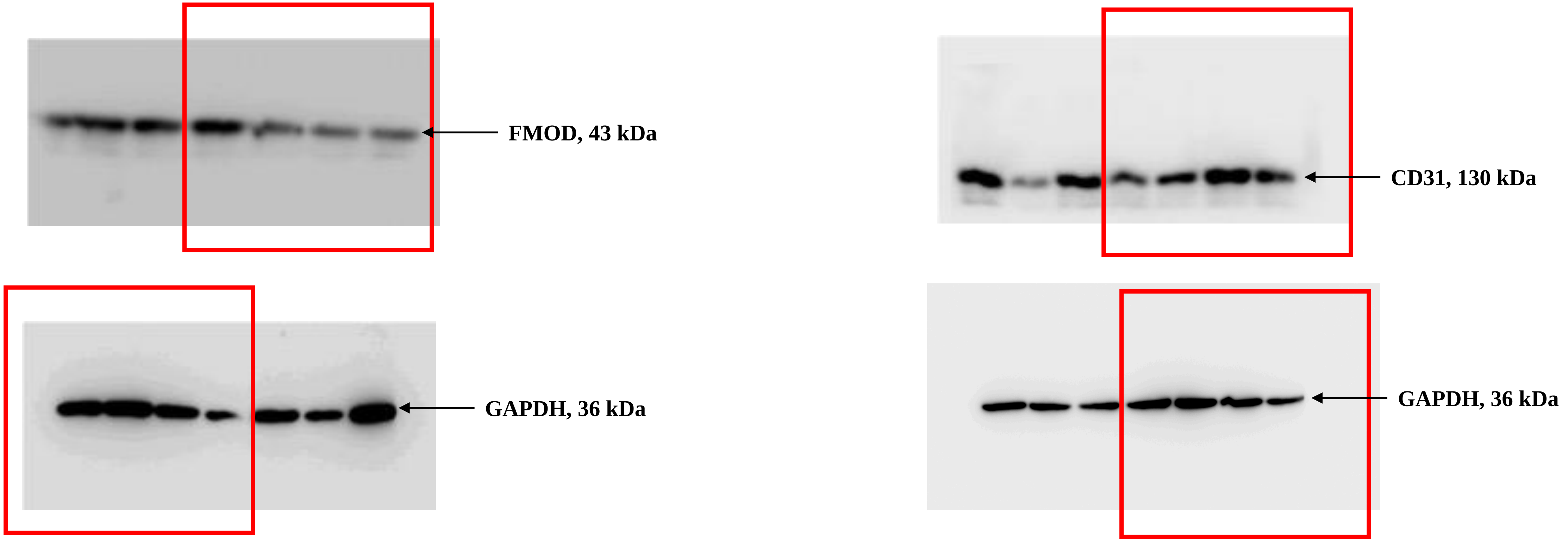

This blot got flipped while developing, the correct order has been put in the main text file, while the raw image has the original version.

Figure 4

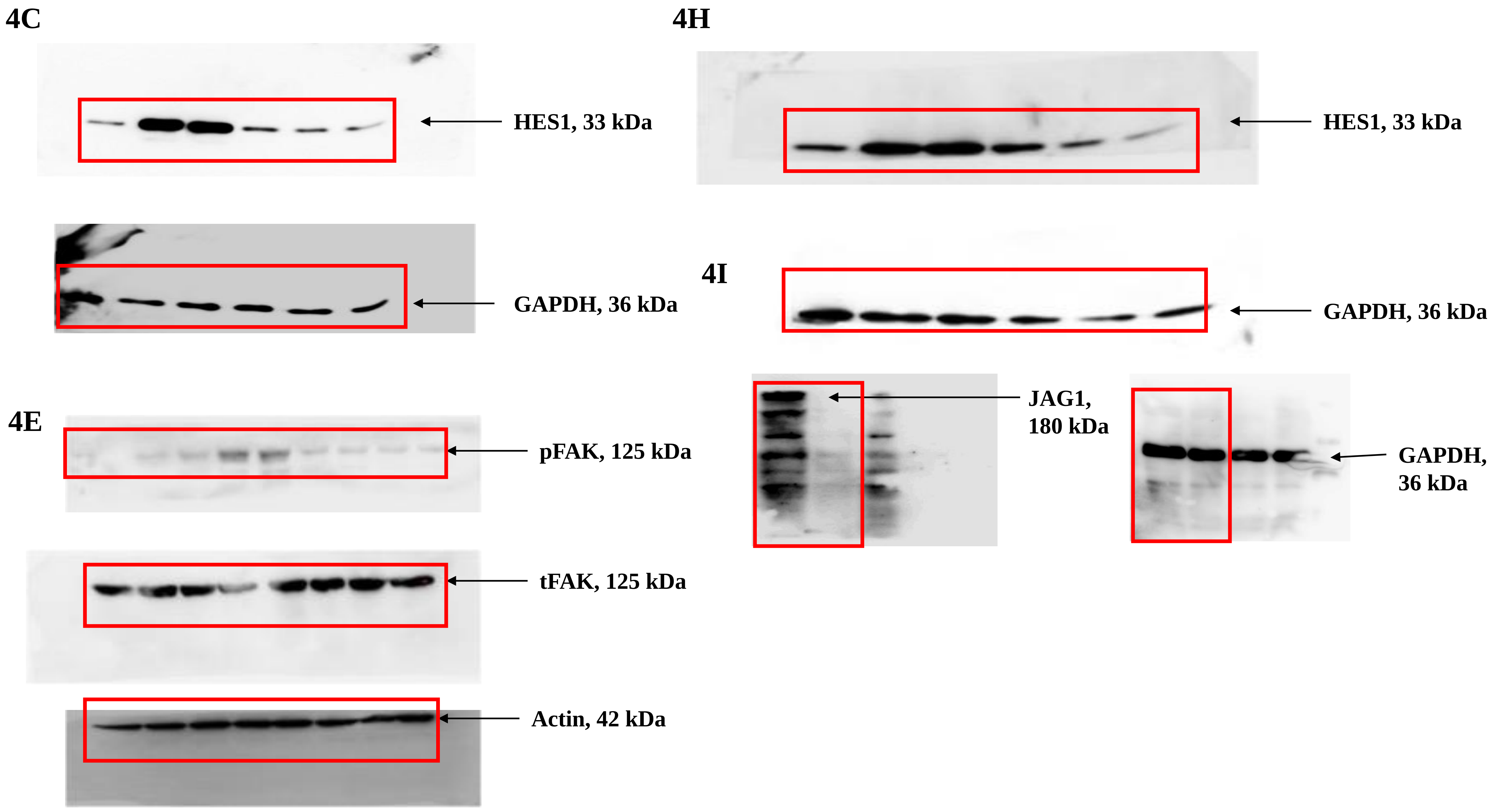

4C
HES1, 33 kDa
GAPDH, 36 kDa
4E
pFAK, 125 kDa
tFAK, 125 kDa
Actin, 42 kDa
4H
HES1, 33 kDa
4I
GAPDH, 36 kDa
JAG1, 180 kDa
GAPDH, 36 kDa

Figure 4

4K

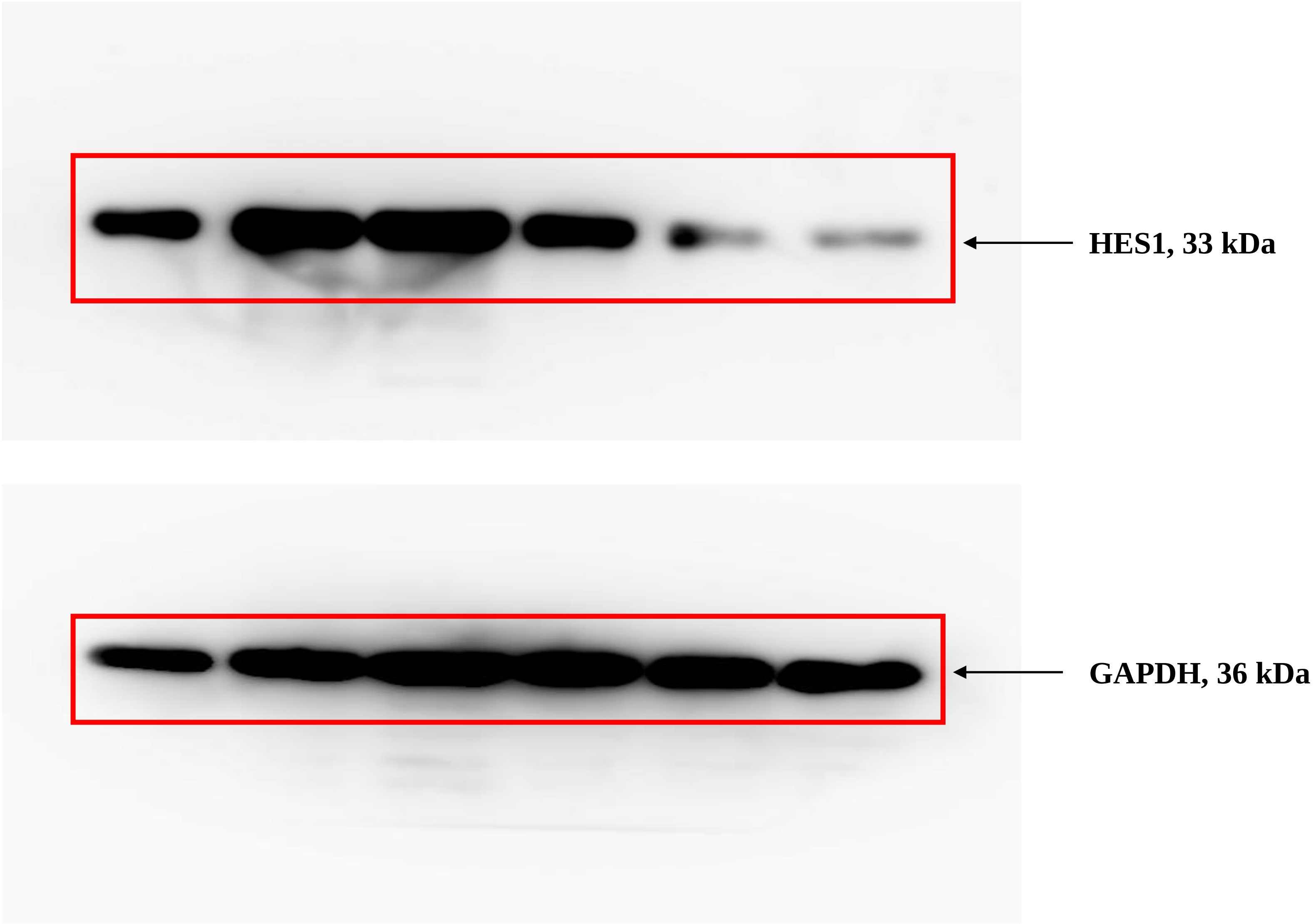

Supplementary Figure 6

6A

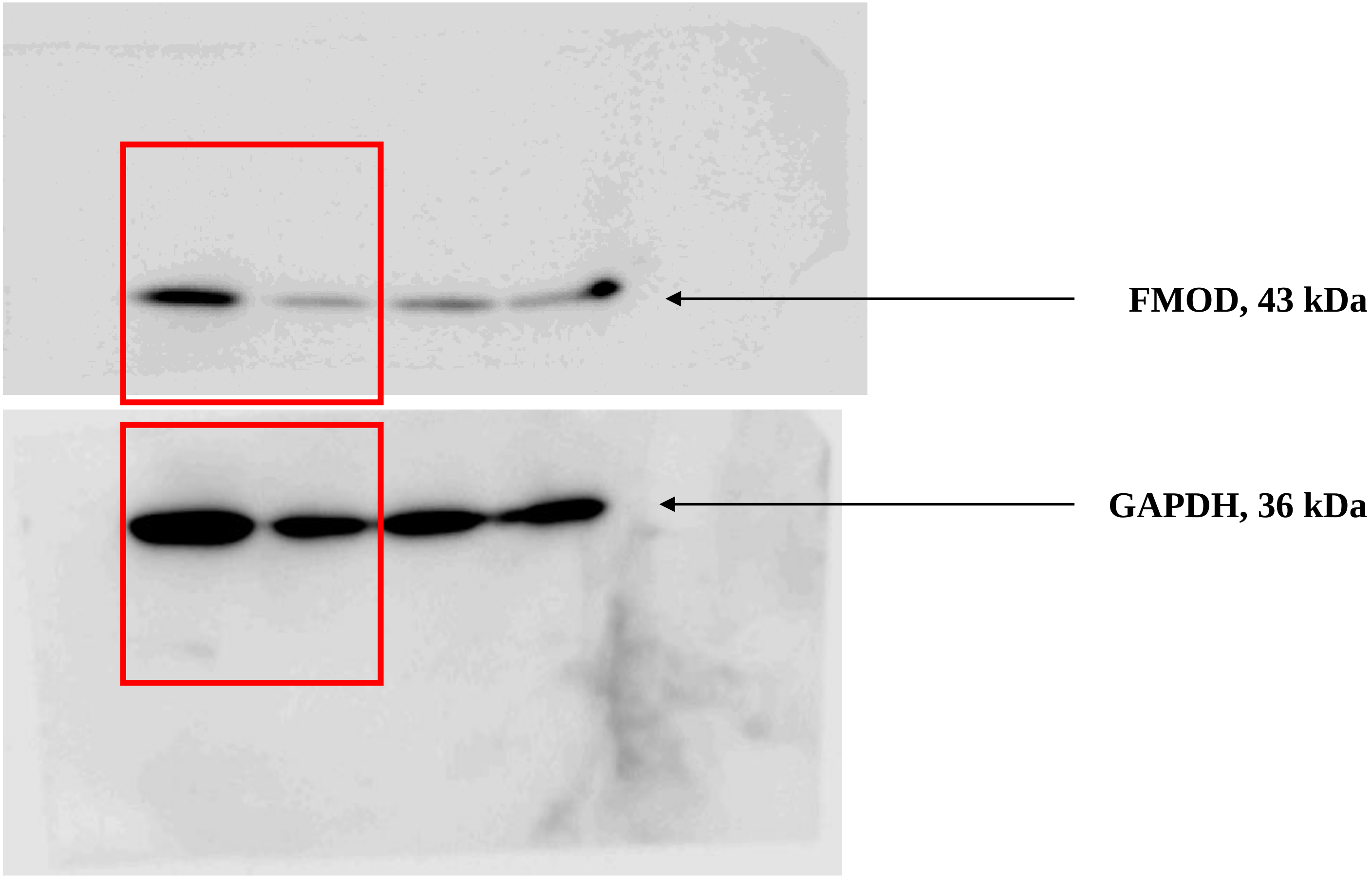

Supplementary Figure 7

7B

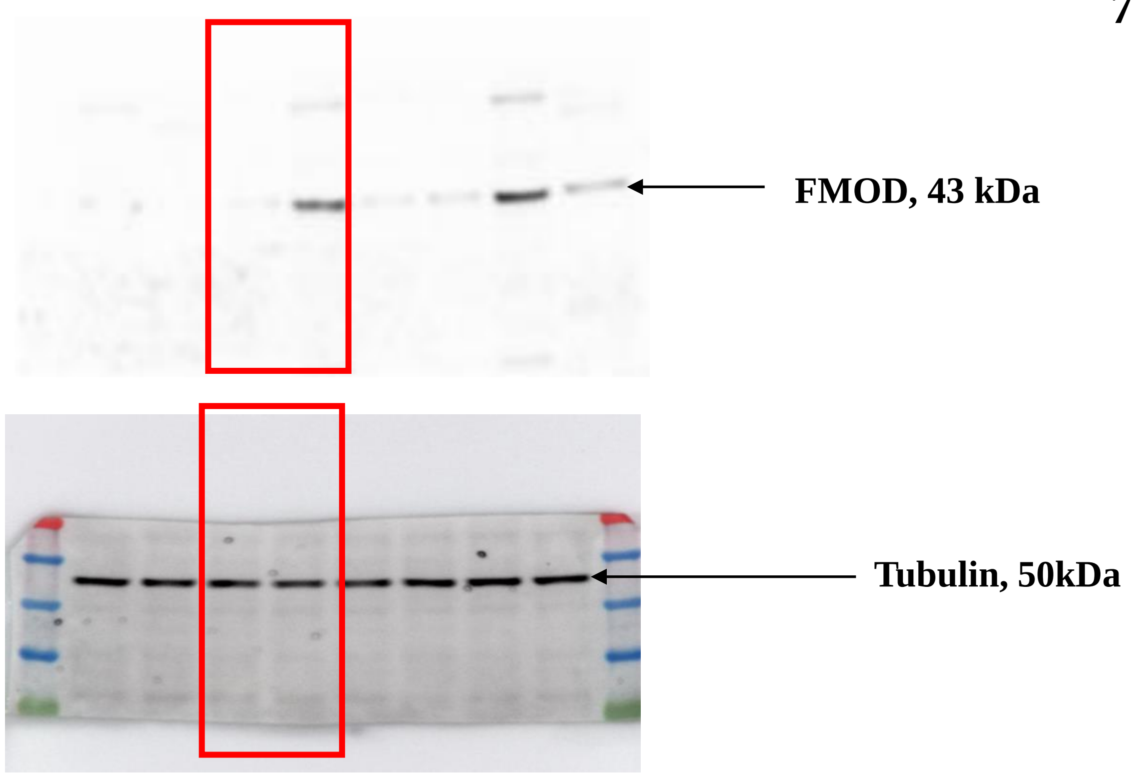

7E

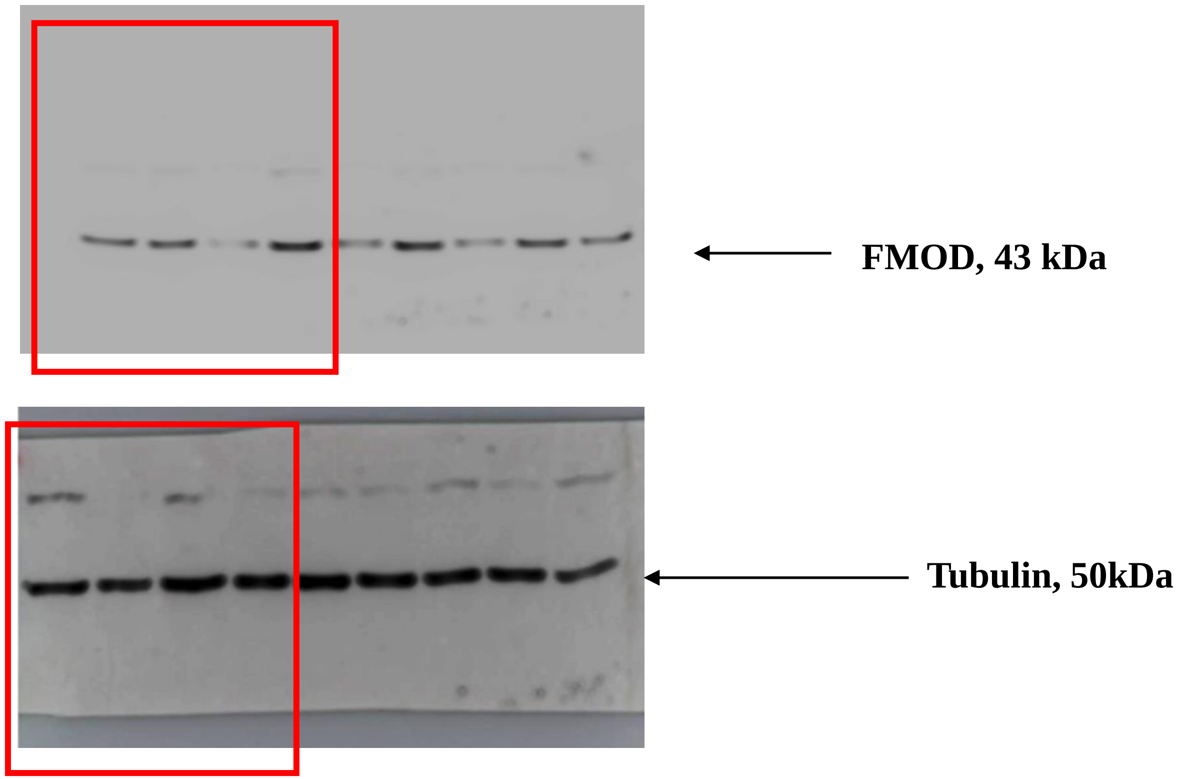

Supplementary Figure 7

7C

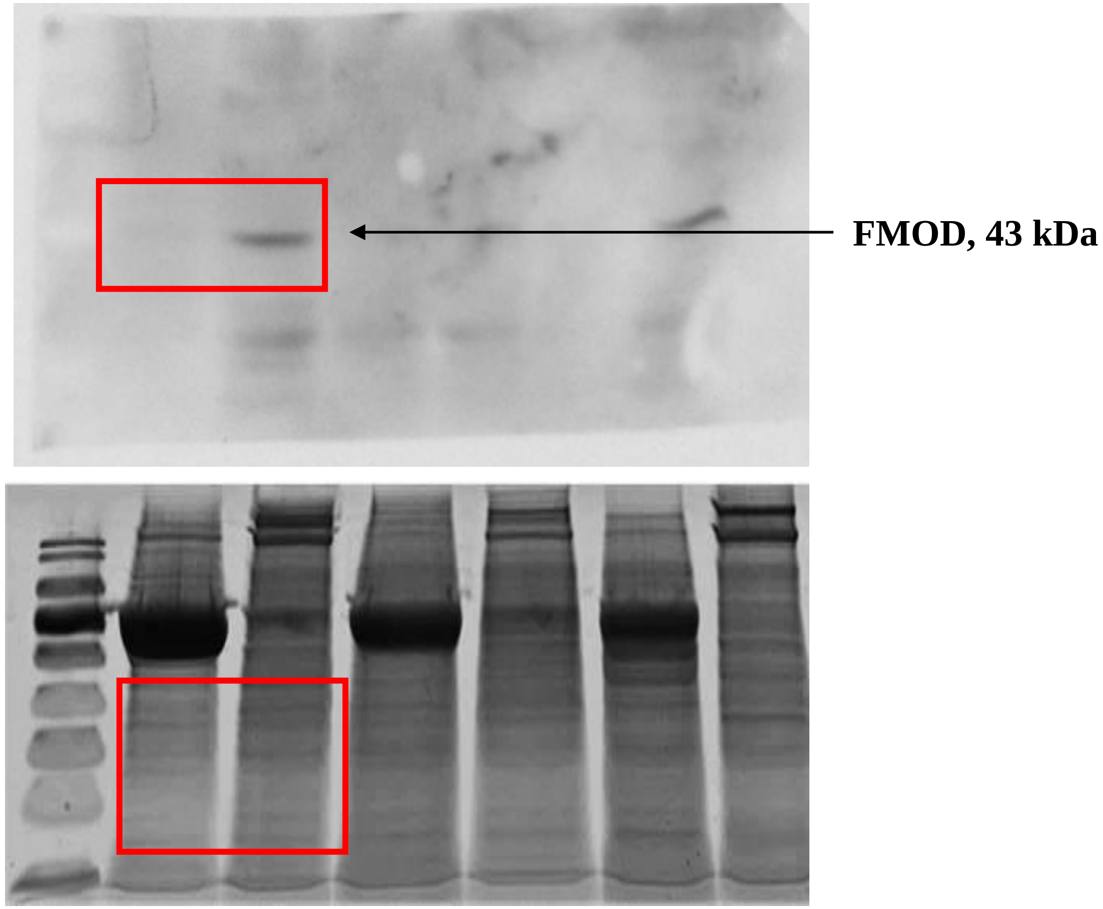

Ponceau stained membrane

Supplementary Figure 10

10C

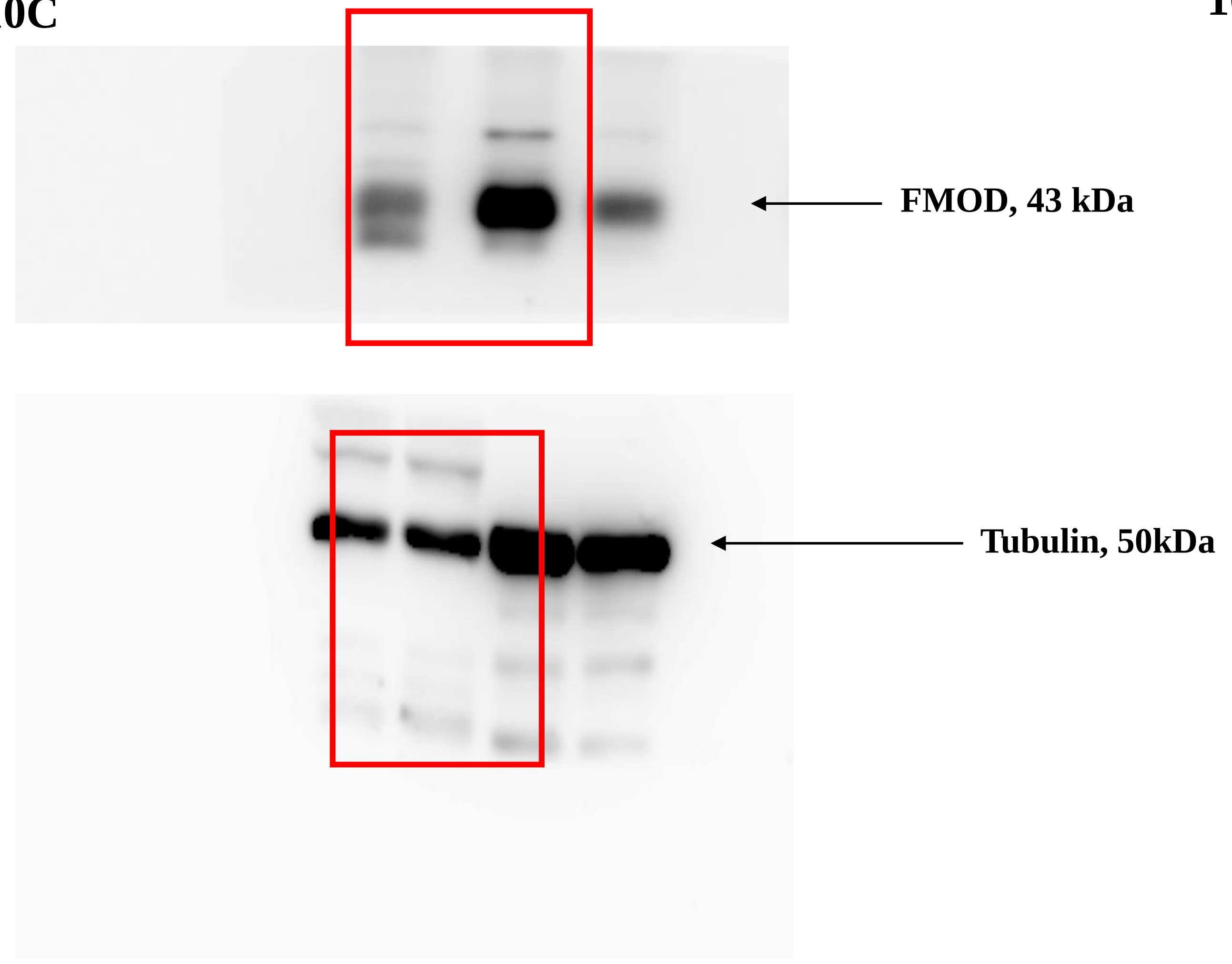

10I

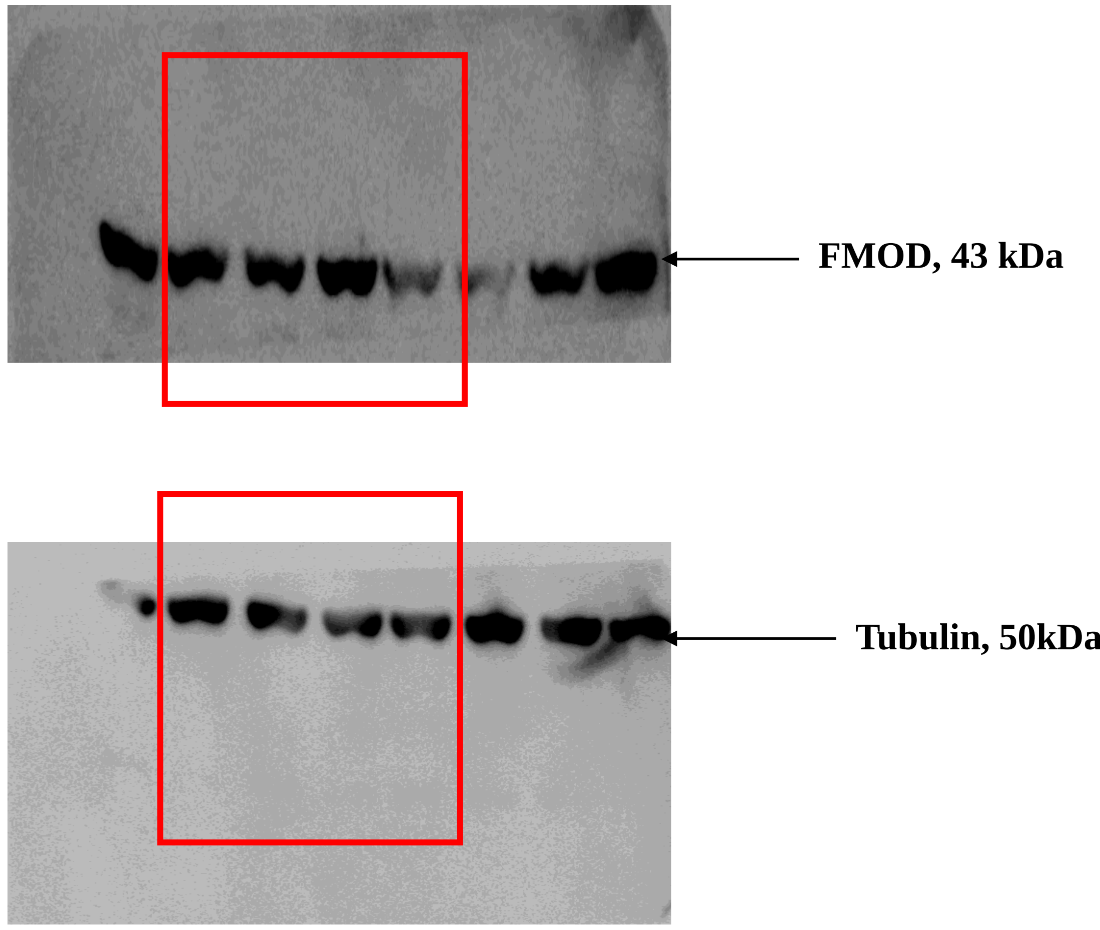

Supplementary Figure 11

Gel pic

11A

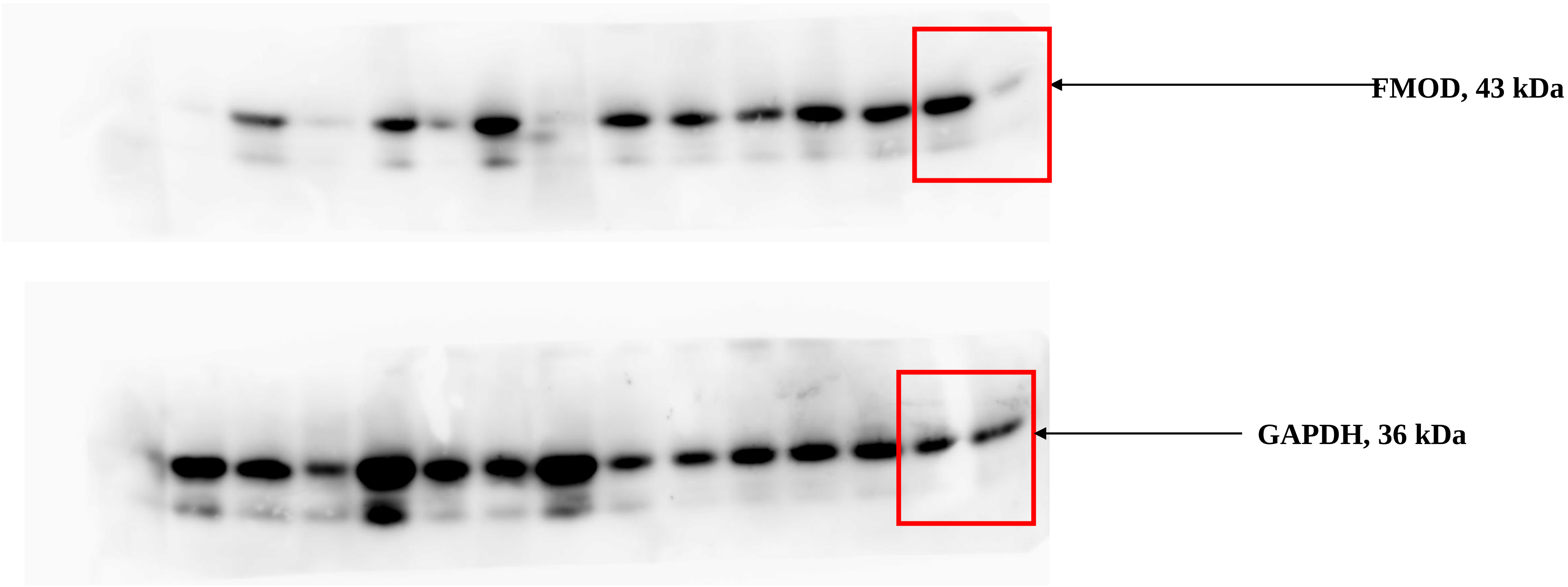

Supplementary Figure 12

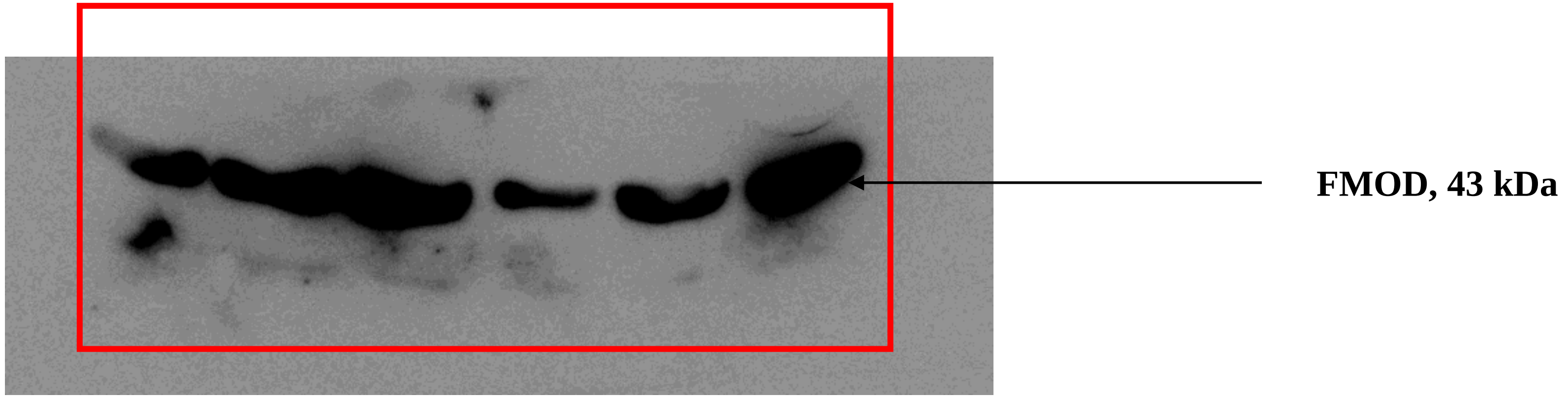

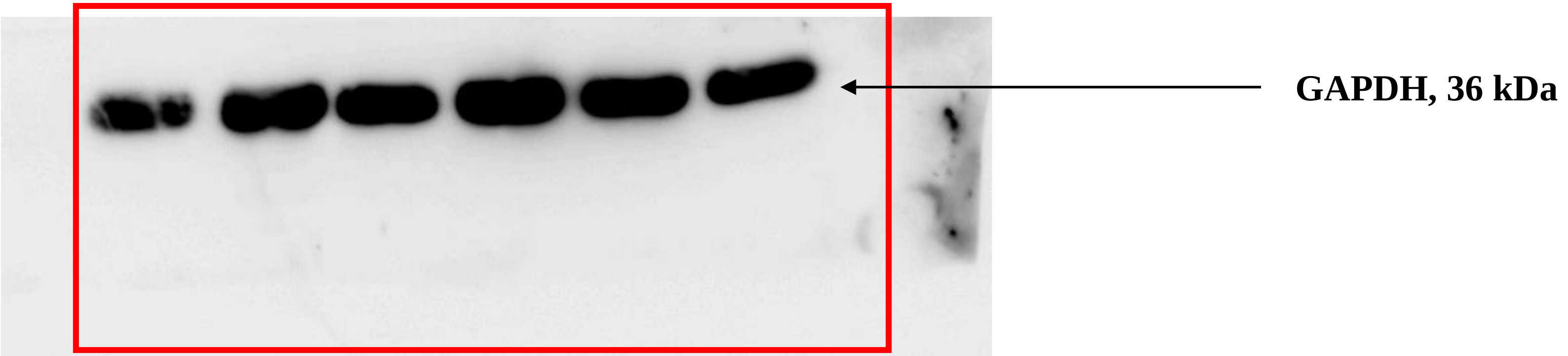

A separate gel was run with less amount of total protein for GAPDH western blot

Supplementary Figure 15

15B

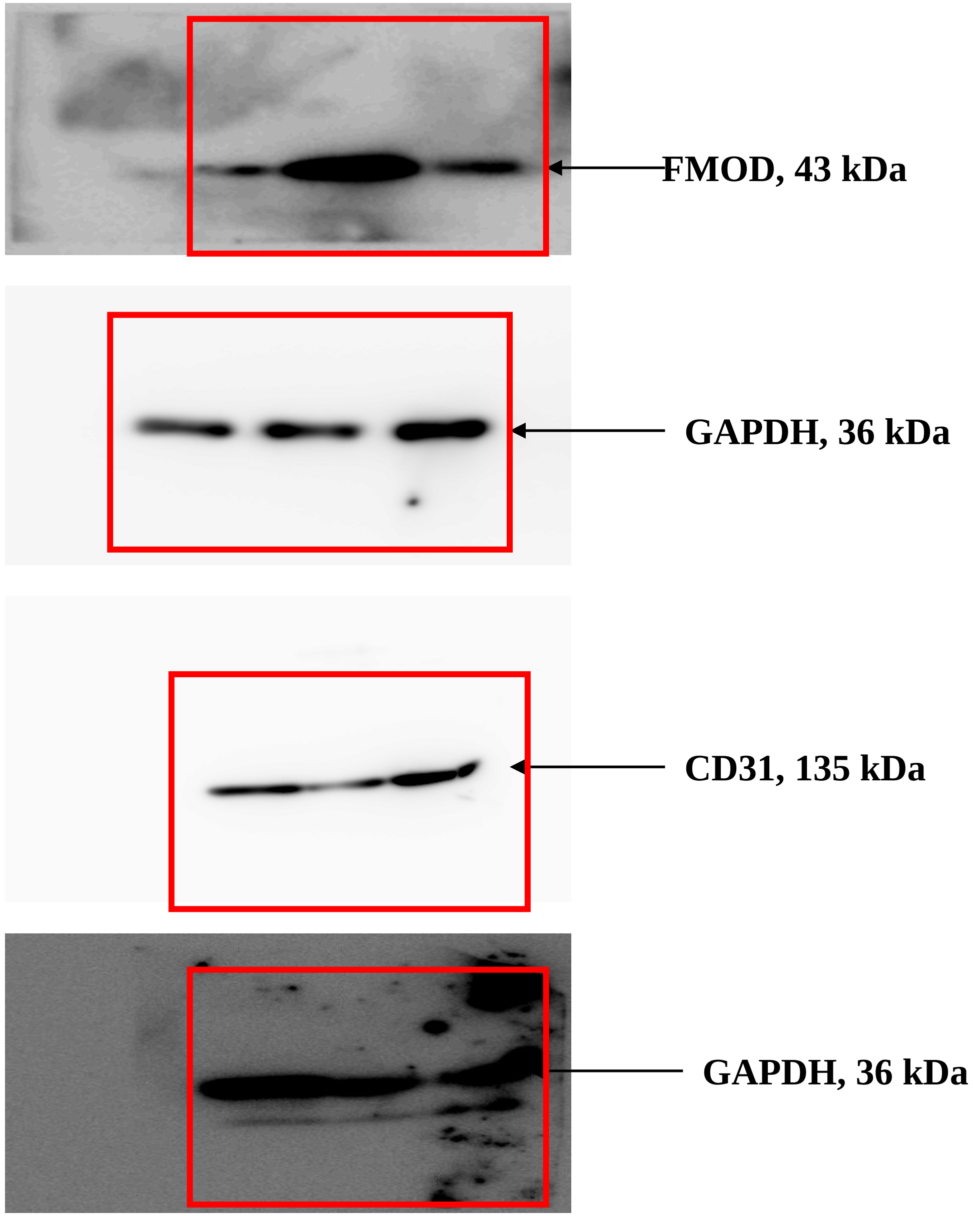

Supplementary Figure 16

16 C

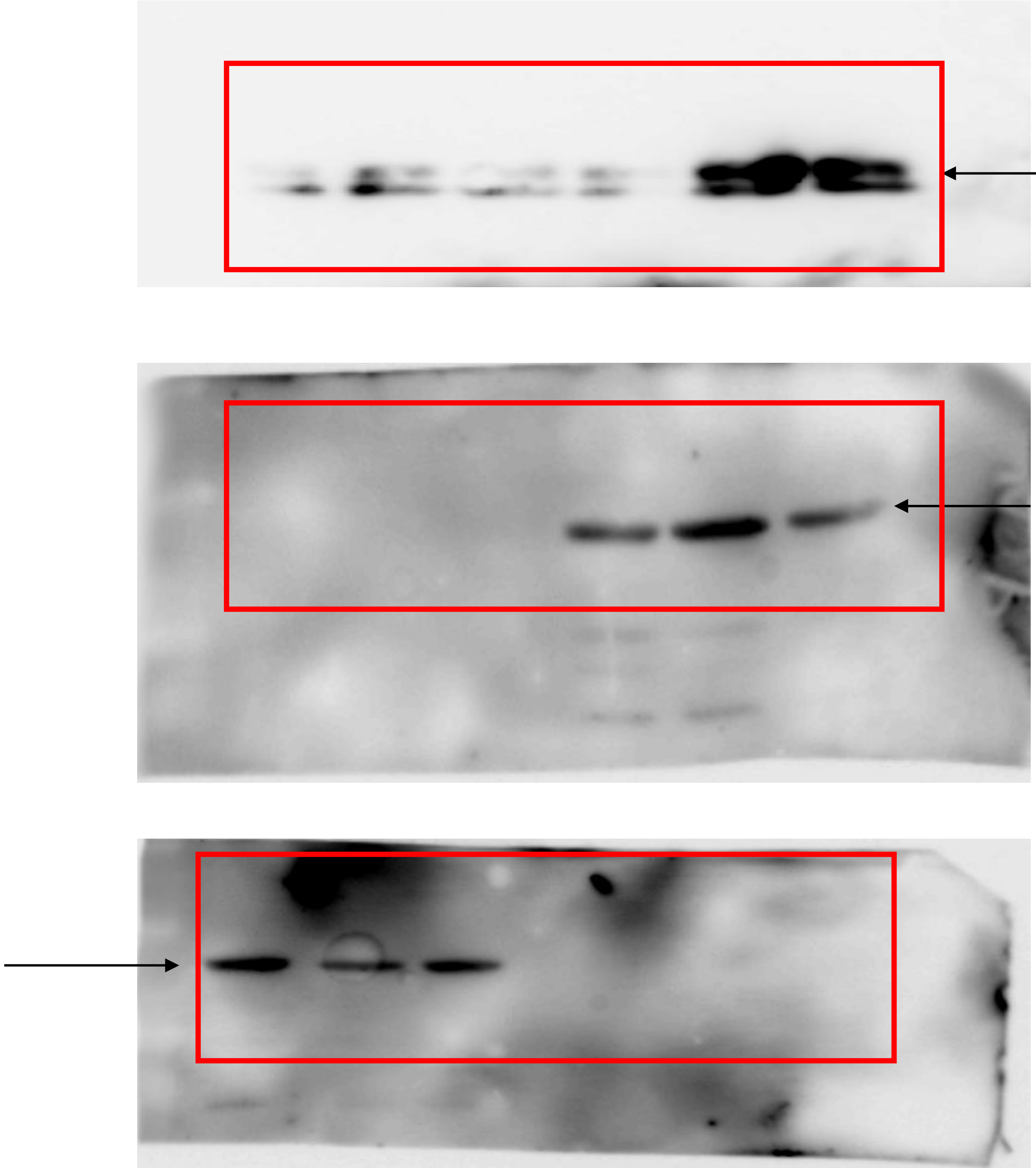

NICD, 110kDa

H3, 17 kDa

GAPDH, 36 kDa

Supplementary Figure 17

17A

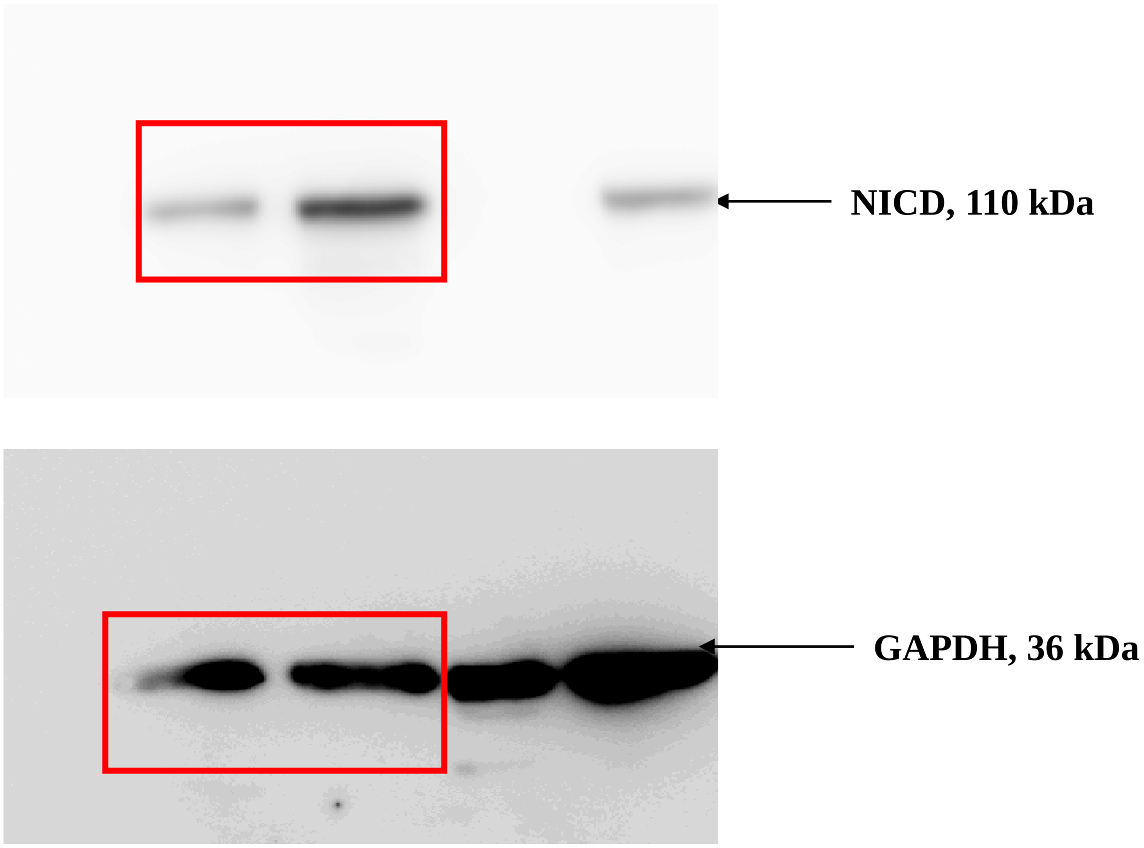

Supplementary Figure 18

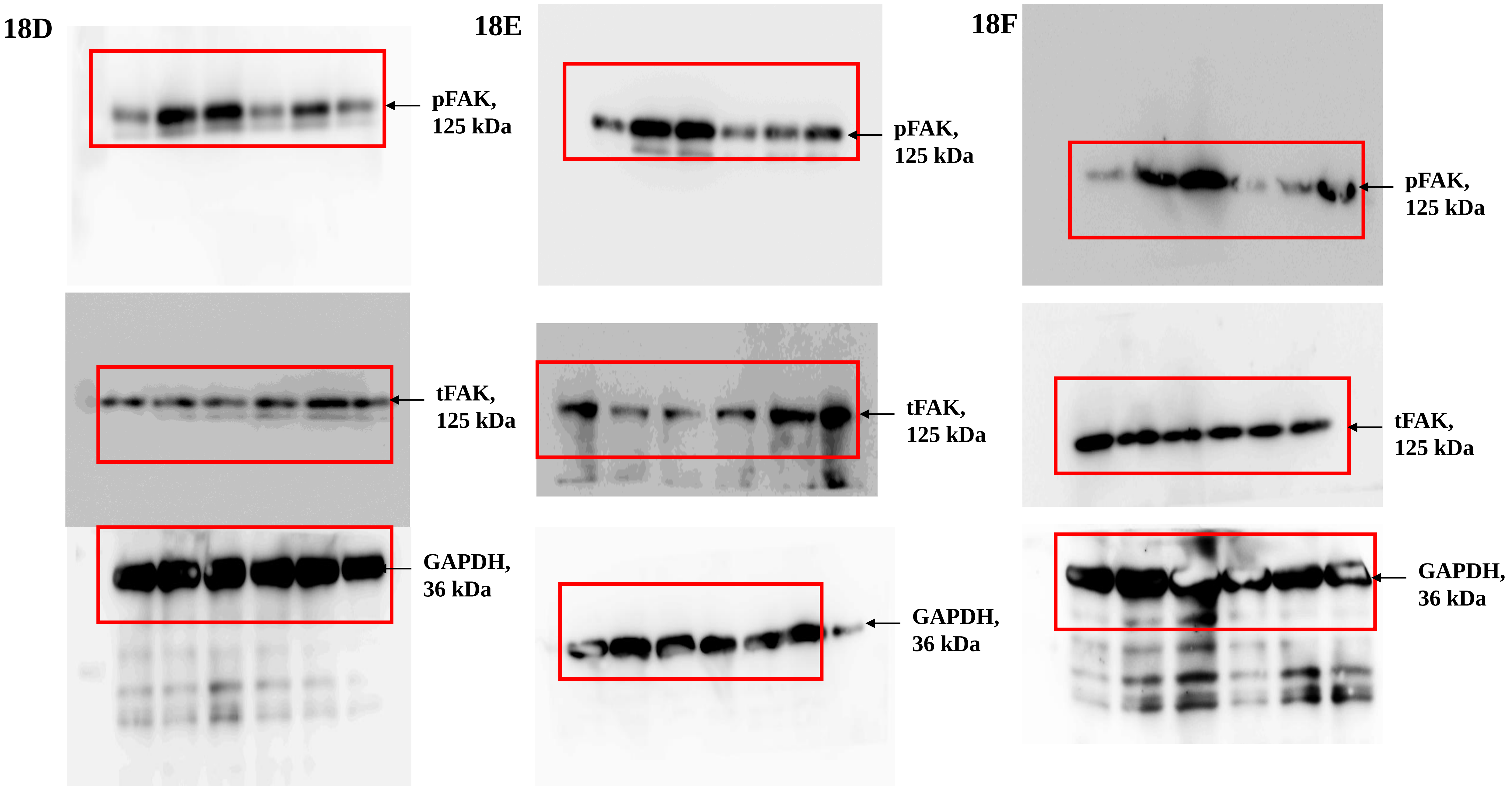

Supplementary Figure 18

18G

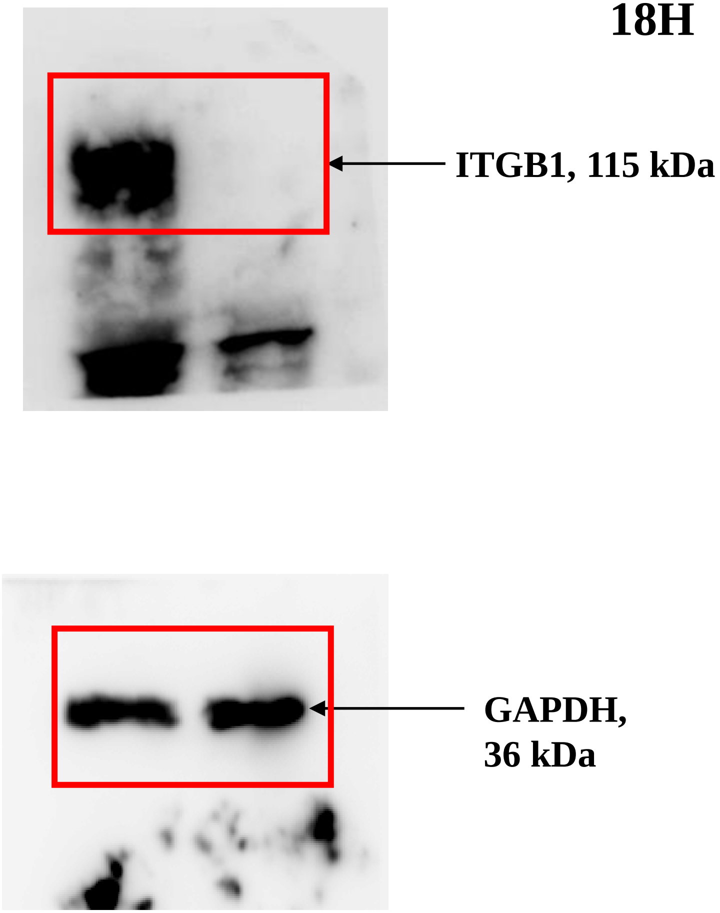

18H

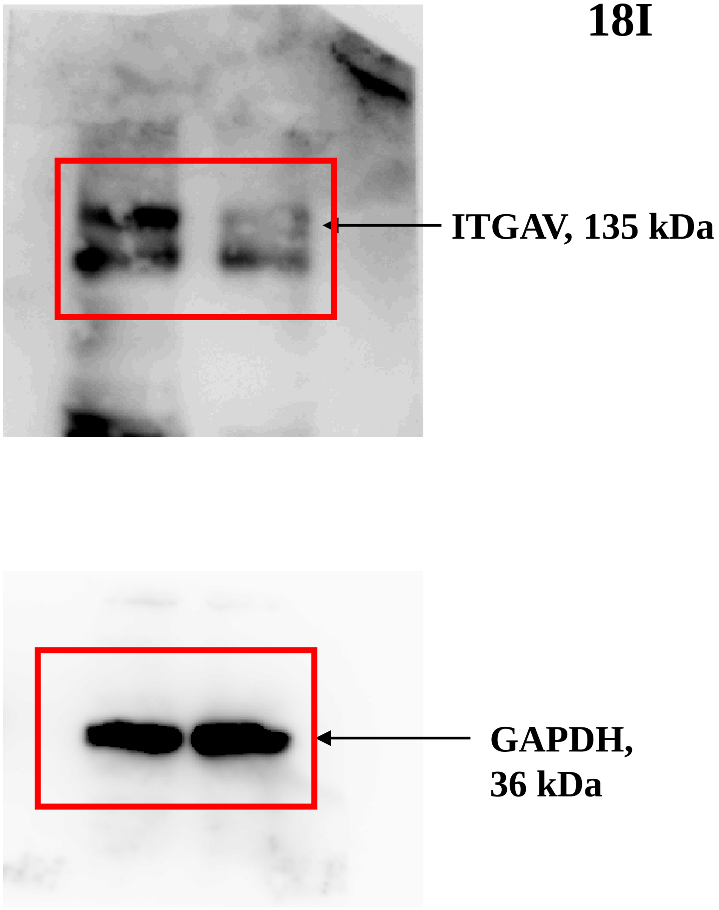

18I

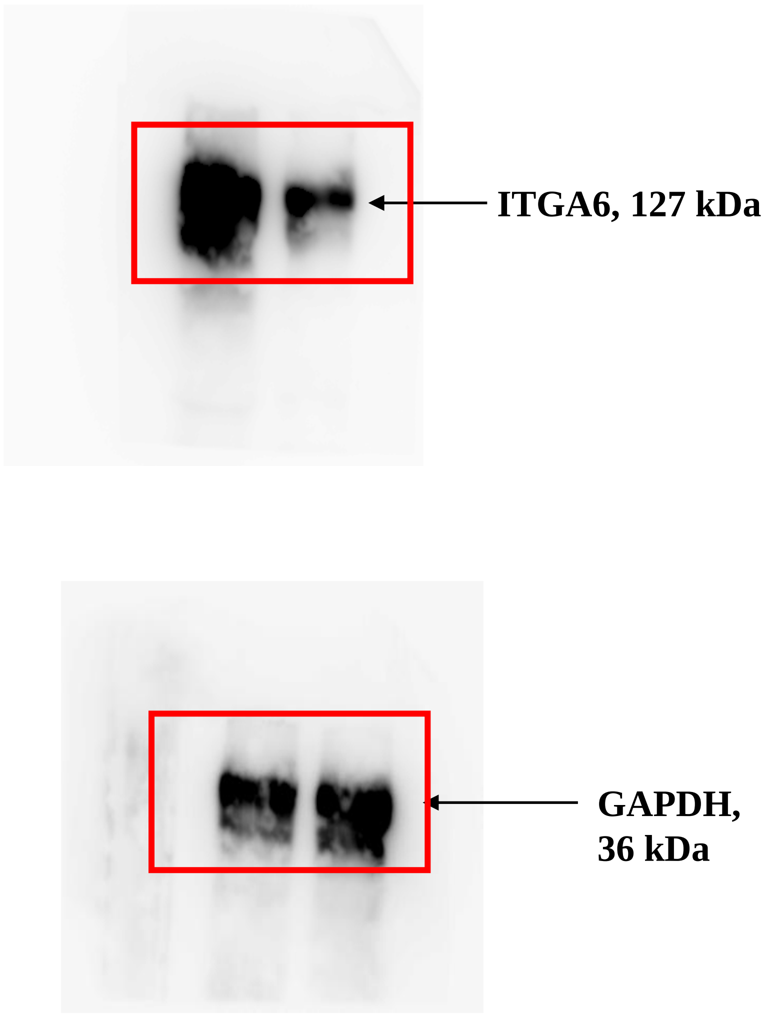

Supplementary Figure 19

19F

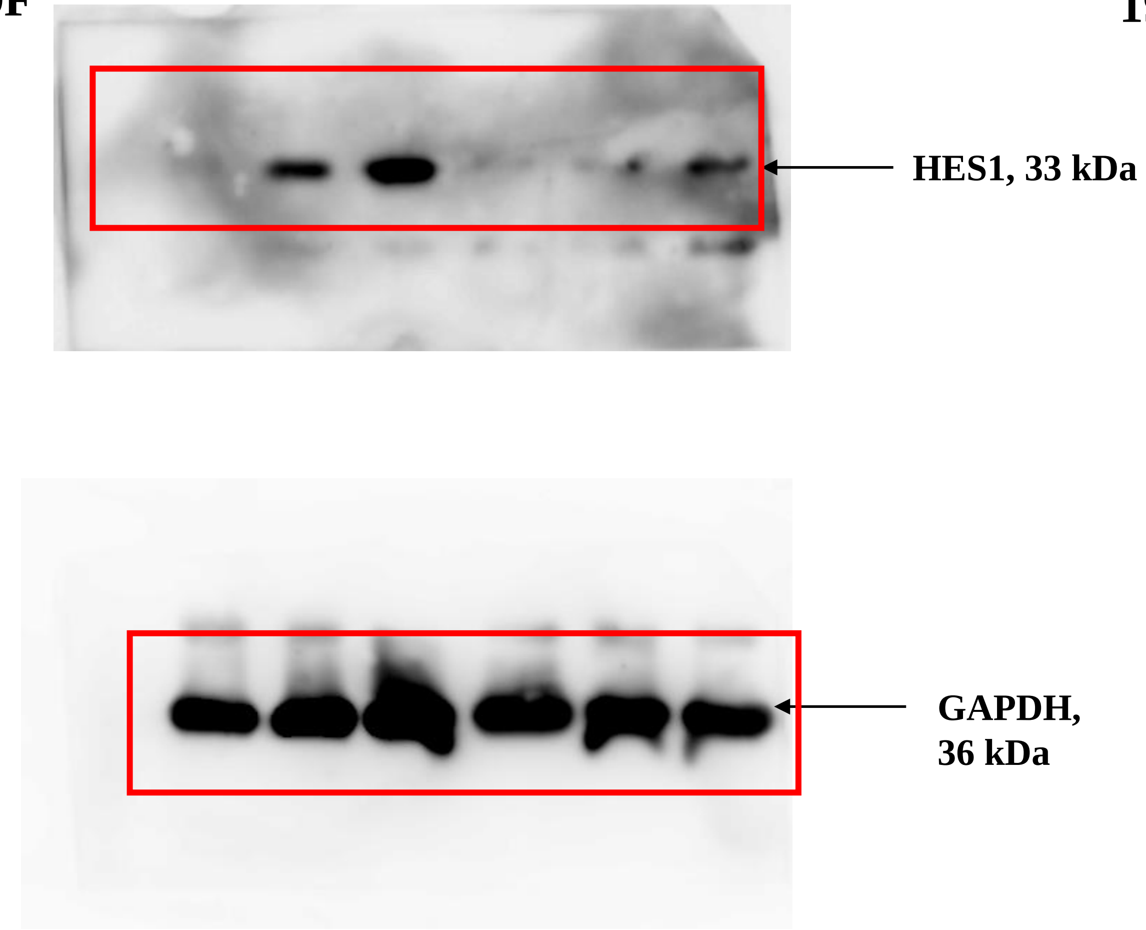

19H

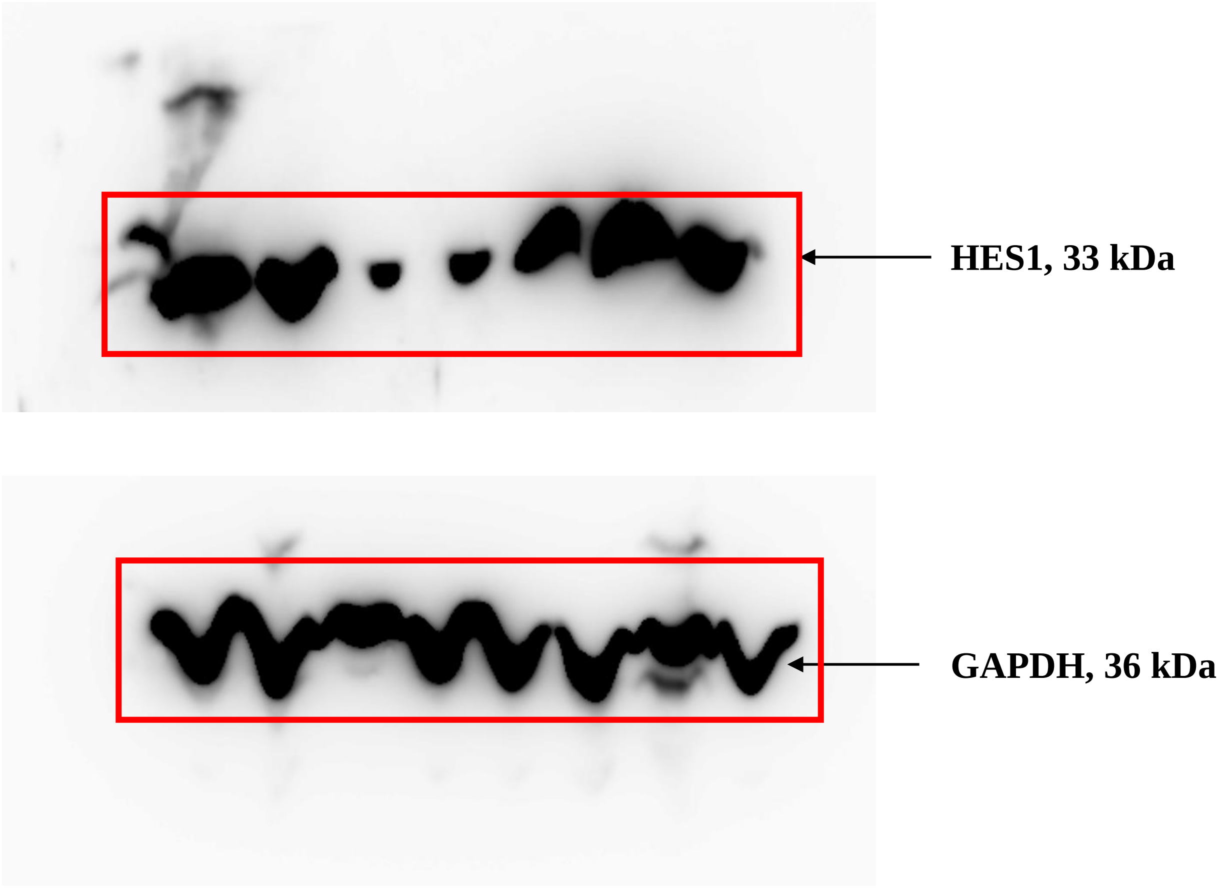

Supplementary Figure 20

20G

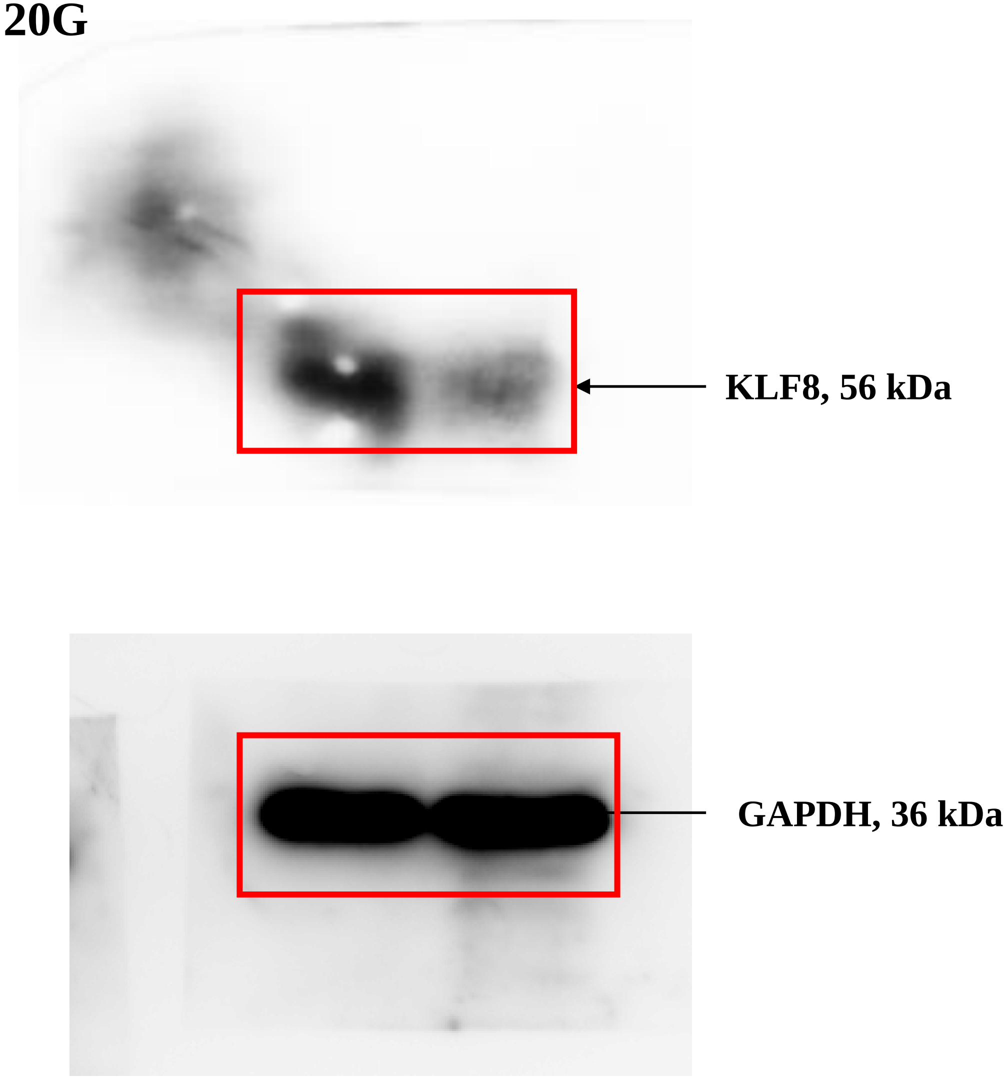

20H

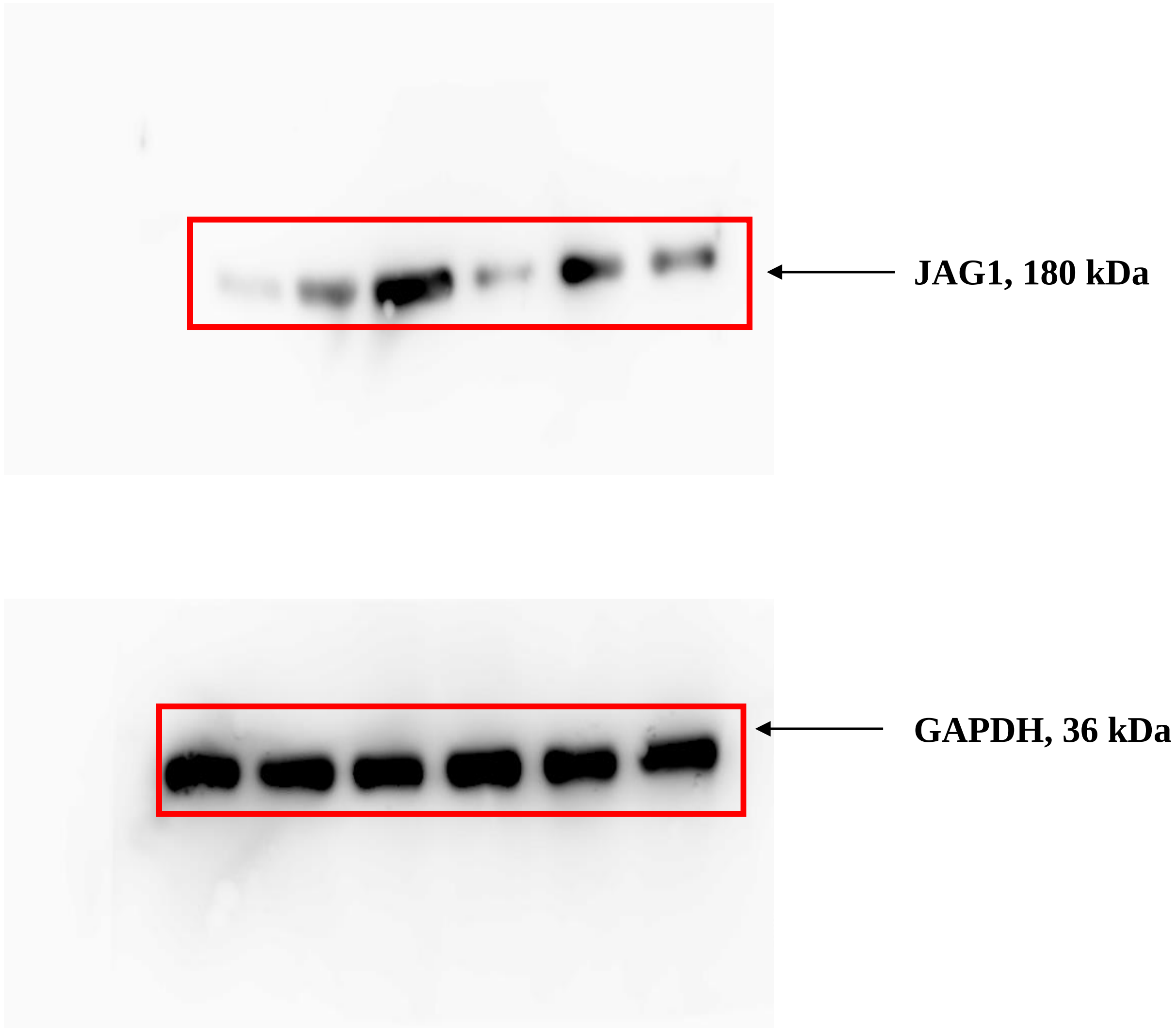

Supplement: Source data 1. [file elife-78972-data1.pdf]
